# Programmable wide-range pH gradients for NMR titrations: application to antibody–drug conjugate linker group modifications†

**DOI:** 10.1039/d5an00406c

**Published:** 2025-05-26

**Authors:** Matthew Wallace, James M. Sharpe, Krzysztof Baj, Michael Ngwube, Jenny Thirlway, Patrick L. Kerigan Higgs, G. Richard Stephenson, Jonathan A. Iggo, Thomas E. Storr, Christopher J. Richards

**Affiliations:** a School of Chemistry, Pharmacy and Pharmacology, University of East Anglia, Norwich Research Park Norwich NR4 7TJ UK matthew.wallace@uea.ac.uk chris.richards@uea.ac.uk; b Department of Chemistry, University of Liverpool Crown Street Liverpool L69 7ZD UK; c Iksuda Therapeutics Ltd., The Biosphere Draymans Way Newcastle Helix Newcastle upon Tyne NE4 5BX UK

## Abstract

We generate pH gradients spanning more than six units in standard NMR tubes and determine all the p*K*_a_ values of polyprotic compounds in single 20 minutes chemical shift imaging (CSI) NMR experiments. The modest demands of our method in terms of sample quantity and preparation time allow it be performed as part of the routine characterisation and optimisation of organic molecules during synthesis campaigns. As proof of concept, we measure the p*K*_a_ values of a family of vinylpyridines employed as antibody drug conjugate linkers. Our analysis reveals a strong correlation between the experimental aqueous p*K*_a_ and the rate of conjugate addition of thiol nucleophiles to the vinyl group, representing a powerful predictive method.

## Abstract 2

Tuneable pH gradients are generated within NMR tubes using buffer components enabling extraction of the p*K*_a_ of polyprotic molecules by chemical shift imaging. Knowledge of p*K*_a_ informs the design of vinylpyridine antibody drug conjugate linkers.

## Graphical abstract

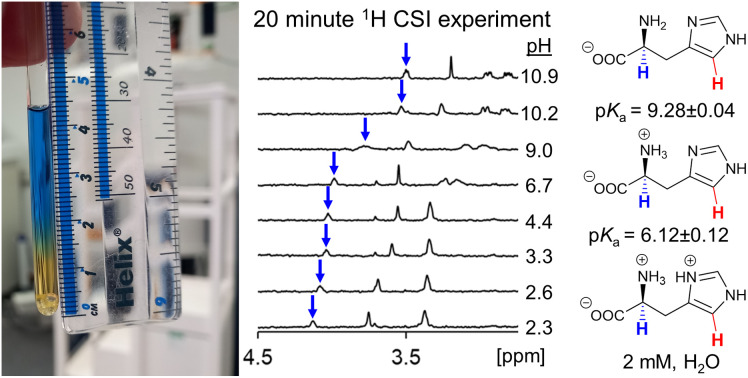

## 1. Introduction

NMR titrations are a highly effective if laborious way to elucidate the pH-responsive behaviour of chemical systems including small molecule pharmaceuticals,^1^ polymers,^2^ proteins^3^ and synthetic supramolecular systems.^4^ As a faster alternative to the conventional titration, we demonstrated that pH gradients could be established in standard NMR sample tubes by placing a solution on top of crystals of an acid. ^1^H NMR spectra were recorded along this gradient using chemical shift imaging (CSI) experiments, thus avoiding the preparation and analysis of separate samples at each pH investigated.^5–7^ Here, we demonstrate how common pH buffer components can be used to generate tuneable, wide-range pH gradients spanning six or more units in standard NMR sample tubes. Adjustment of the buffer composition, guided by simple calculations, allows tuning of the pH gradient to span a wide range, or a narrow range covering acidic or basic pH as desired. Using this approach, we accurately determine all three p*K*_a_ values of histidine in one experiment, the side-chain and amino p*K*_a_ values of tyrosine and lysine, and the side-chain and carboxyl p*K*_a_ values of aspartic acid. Polyprotic molecules are especially difficult to analyse using conventional NMR titrations due to the large number of pH values (points) required.^8–10^ Finally, using substituted 4-vinylpyridines, we demonstrate how our method can be used to optimise the reactivity of families of organic molecules where protonation or deprotonation is a key mechanistic step. We compare our experimental p*K*_a_ values to site specific micro-p*K*_a_ values predicted by machine learning (MolGp*K*_a_^11^ and Epik 6.8^12^) and DFT (Jaguar p*K*_a_^13–15^) methods. For monobasic pyridine derivatives, these computational approaches return predictions in reasonable agreement (<1 unit) with the NMR-derived values. However, significant differences are observed for pyridine derivatives with more than one basic site both in terms of the absolute p*K*_a_ values and relative basicity of the sites. These results highlight the necessity for the experimental determination of the p*K*_a_ for polyprotic compounds during synthesis campaigns. Our method can be applied using automatic NMR equipment and provides a useful addition to the organic chemist's analytical toolbox.

## 2. Materials and methods

### 2.1. Materials

All reagents were purchased from commercial suppliers and used as received. Milli-Q water (18.2 MΩ cm) was used throughout the study. Amino acids were analysed at a concentration of 2 mM. The 4-vinylpyridine derivatives A–M were synthesised in house as described in section S11 of the ESI.† Aqueous solutions of these pyridines for analysis were prepared by weighing sufficient compound into a 5 mL volumetric flask to give a theoretical concentration of 2 mM assuming complete dissolution upon stirring with a PTFE stirrer bar for at least one hour. Analysis was performed on the supernatant solution.

### 2.2. Creation of pH gradients

**Fig. 1:**
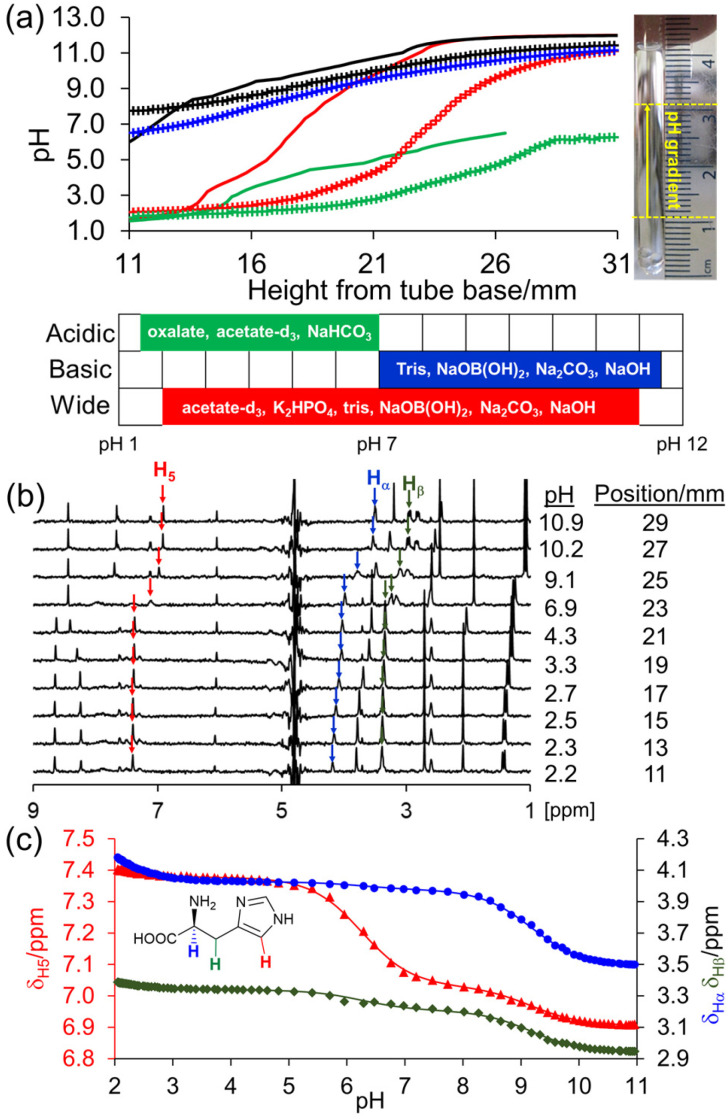
(a) Predicted (solid line) and experimental (cross) pH *versus* position from the base of the NMR tube for determination of the p*K*_a_ values of tyrosine (OH, NH_2_: blue), lysine (NH_2_: black), histidine (NH_2_, imidazole, COOH: red) and aspartic acid (COOH: green). Photograph of NMR sample with NMR-active region indicated as yellow dashed lines. Sketch of pH range accessed with each buffer system. (b) Example ^1^H spectra of histidine extracted from CSI image at pH value and vertical position indicated. (c) Plot of ^1^H chemical shift of α (blue circle), β (green diamond) and H_5_ proton (red triangle) of histidine *versus* pH. The solid lines are fits to eqn (1).

The following indicator compounds were included in the samples to allow determination of the pH between 1 and 12 *via* their ^1^H chemical shifts:^5^ 2 mM sodium acetate, disodium methylphosphonate (MPA), sodium glycinate, methylammonium chloride, sodium dichloroacetate (DCA), 4 mM sodium formate, and 1 mM 2,6-lutidine. Sodium 3-(trimethylsilyl)-1-propanesulfonate (DSS, 0.2 mM) was used as a chemical shift reference for all spectra. The set of indicators used was adjusted depending on the pH range under assessment. To access more basic pH ranges (lysine and tyrosine, Fig. 1a), DCA, formate, acetate and 2,6-lutidine were excluded. Methylammonium chloride was excluded from experiments used to assess more acidic ranges (aspartic acid, Fig. 1a). For analysis of 4-vinylpyridine derivatives, *N*-hydroxysuccinimide (NHS, 2 mM) was substituted for 2,6-lutidine to avoid spectral overlap with the pyridine resonances.

To prepare a pH gradient, crystals of oxalic acid dihydrate were weighed directly into a Wilmad 528-PP 7′′ NMR tube. The nominal masses were within 20% of the stated values. Four 2 mm diameter glass beads were then placed in the tube. Finally, a solution of pH buffers, analyte and indicators was placed on top of the glass beads using a 9′′ NMR Pasteur pipette to a height of 40–45 mm above the absolute base of the NMR tube. The sample was then stood at 22 °C in the autosampler rack of the spectrometer prior to analysis.

### 2.3. NMR analysis and data processing

All experiments were performed on a Bruker 500 MHz Avance III spectrometer equipped with a Bruker Ascend superconducting magnet in 100% H_2_O, without lock, at 298 K to enable direct comparison with published p*K*_a_ values. However, we note that CSI experiments on pH gradients can be performed with lock on a range of solvent systems.^7^^1^H spectra of a sample recorded before and after a CSI experiment confirmed minimal drift (<1 Hz) in the frequency of H_2_O. ^1^H CSI datasets were acquired using a gradient phase encoding sequence with water suppression (section S14.3†). The delay between successive pulses in the selective pulse train was set at 333 μs corresponding to a 3000 Hz separation between the null points. The phase encoding gradient pulse was in the form of a smoothed square and was ramped according to the number of rows collected in the dataset. Amino acids, compounds A–F, K–L: −25 to 25 G cm^−1^ in 128 steps, 8 scans at each increment, giving a theoretical spatial resolution of 0.20 mm along the *z*-axis. Compounds G–J: −18.8 to 18.8 G cm^−1^ in 64 steps, 16 scans, 0.41 mm resolution. Compound M: −5.0 to 5.0 G mm^−1^ in 16 steps, 64 scans, 1.6 mm resolution. The signal acquisition time and relaxation delay were 1 s and 0.26 s, respectively giving a total experimental time of 22 minutes 31 seconds for all experiments. A spoil gradient (1 ms, 27 G cm^−1^) was employed at the end of the acquisition period to destroy any remaining transverse magnetization.

CSI datasets were processed in phase-sensitive mode with 32 K points and an exponential line broadening factor of 3 Hz. Each row was phased, baseline corrected, referenced to DSS (0 ppm) and the chemical shifts of indicators and analytes exported to the spreadsheet accompanying this work using automation scripts written in house (section S14†). Only rows 20–120 (128 point dataset) were used in the analysis to avoid off-coil effects. The pH was determined from the ^1^H chemical shifts of organic indicator molecules and ions (sections S1 and S3†). Fitting of the analyte chemical shifts to eqn (1) was accomplished using the Solver module of Microsoft Excel optimising all variables, with the constraint for histidine that the fitted *δ*_H3_ was less than 1 ppm higher than the lowest experimental chemical shift measured in the data series.

## 3. Results and discussion

### 3.1. Design of pH gradients and data analysis

pH gradients are created by placing a basic solution on top of solid crystals of oxalic acid dihydrate. This acid is particularly convenient owing to its absence of ^1^H NMR resonances, non-deliquescent crystals that enable direct weighing into the NMR sample tube, and high acid strength that enables access to a wide pH range (pH 2–12). The spreadsheet accompanying this work enables selection of the mass of oxalic acid, buffer composition and time to leave the sample following preparation, *t*_opt_, so that the user can create pH gradients spanning any desired range. The theory and calculations underpinning the spreadsheet are described in section S2 of the ESI.† The buffers comprise inorganic phosphate, carbonate and borate species along with the inexpensive deuterated organic buffers, tris(hydroxymethyl-d_3_)amino-d_2_-methane (tris) and acetate-d_3_. Combining these buffers in different proportions permits access to different pH ranges, based on the p*K*_a_ of the protonated buffer species, p*K*_a,H_ (Table 1). The pH of the sample at each position is determined from the ^1^H chemical shifts of organic indicator molecules as described in our previous work and section S3 of the ESI.†^5^

**Table 1:** pH buffer components used in this work

Buffer	p*K*_a,H_^a^	pH range^b^
NaOH	—	>11
Na_2_CO_3_	10	9.2–10.8
NaOB(OH)_2_	9	8.2–10.1
Tris^c^	8	7.2–9.0
K_2_HPO_4_	7	6.1–7.9
NaHCO_3_	6	5.4–7.3
Acetate	4.7	3.8–5.7
Oxalate^2−^	4	2–5^d^

a p*K*_a_ of protonated buffer species.

b pH range over which buffer is between 90% and 10% protonated.

c Tris(hydroxymethyl-d_3_)amino-d_2_-methane.

d pH range accessible using oxalic acid/oxalate alone.

This highly tuneable pH range allows us to determine the p*K*_a_ values of molecules that possess several ionisable groups. Here, we use amino acids bearing ionisable side chains as exemplar systems. To extract the three p*K*_a_ values of histidine in a single experiment, we require a smooth pH gradient spanning approximately pH 2 to pH 11. We choose a buffer system where the p*K*_a,H_ values of the buffers are separated by approximately two units or less: NaOH, Na_2_CO_3_, NaOB(OH)_2_, tris, K_2_HPO_4_ and acetate-d_3_. With 3.7 mg of oxalic acid placed at the bottom of the NMR tube and with 10 mM of each buffer component, the concentration of oxalic acid is predicted to vary from 110 mM to 2 mM from the bottom to the top of the NMR active region of the sample (Fig. 1a), 6.5 hours after preparation, giving a pH gradient spanning the required range. We note that this calculated *t*_opt_ can vary considerably between samples due to differences in the identity and concentration of buffers selected, the pH range targeted, and the mass of acid weighed into the sample tube. However, there is a *ca.* 2-hour window either side of the calculated *t*_opt_ over which acceptable p*K*_a_ values are obtained (section S4†).

Resolution of the two p*K*_a_ values of aspartic acid associated with the COOH groups requires a pH gradient spanning pH 1 to pH 7. We choose buffer components with lower p*K*_a,H_ values (10 mM NaHCO_3_, acetate-d_3_ and 20 mM oxalate), 3.4 mg of oxalic acid and leave the sample seven hours before analysis. Similarly, resolution of the p*K*_a_ values of lysine (NH_2_) and tyrosine (NH_2_, Ar–OH) requires pH gradients spanning pH 6 to pH 12. This alkaline pH range is accomplished using more basic buffer components (0.01 M NaOH, 20 mM Na_2_CO_3_, tris and NaOB(OH)_2_), a smaller mass of oxalic acid (1.3 mg), and leaving the sample 8.5 hours before analysis.

When prepared and analysed by CSI, the pH exhibits a smooth variation over the desired pH ranges, as predicted by our spreadsheet (Fig. 1a). We attribute the discrepancy between the predicted and experimental pH values to the coupled diffusion of acid and base along the NMR tube and the ±0.3 mg uncertainty in the mass of oxalic acid used in the experiments (section S6, ESI†).

The ^1^H NMR chemical shifts of the pH indicator molecules and amino acids were extracted from the CSI datasets using the routines and scripts provided in section S14 of the ESI.† This data was then exported to the spreadsheets accompanying this work and fitted to extract the pH of each row. The p*K*_a_ values of the amino acids were obtained by fitting the observed chemical shift, *δ*_obs_, of each observed site on the molecule to eqn (1):1
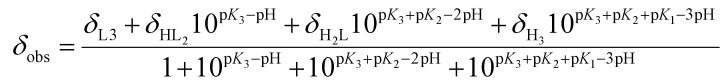
where *δ*_L3_, *δ*_HL2_, *δ*_H2L_ and *δ*_H3_ are the fitted limiting chemical shifts of the fully deprotonated, monoprotonated (p*K*_3_), diprotonated (p*K*_2_) and fully protonated (p*K*_1_) states, respectively. *δ*_L3_, *δ*_HL2_, *δ*_H2L_ and *δ*_H3_ of each observed resonance and the p*K*_a_ values were treated as free variables in the fitting. Only two p*K*_a_ values and three limiting shifts were fitted for lysine, tyrosine and aspartic acid as high pH-resolution was required over the alkaline and acidic pH ranges, respectively (section S4 of the ESI†). The fitted p*K*_a_ values, p*K_n_*, were converted to their thermodynamic values (*I* = 0), p*K*_*n*,0_, using eqn (2):^5,16^2
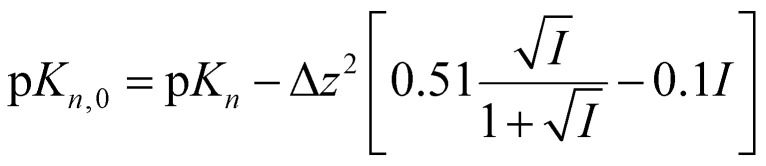
where Δ*z*^2^ is the difference in the square of the overall charge of the molecule between the protonated and deprotonated sides of each protonation equilibrium (*z*^2^_H*n*_–*z*^2^_H(*n*−1)_). The p*K*_*n*,0_ values obtained agree with literature values within experimental uncertainty (Table 2).

**Table 2:** Thermodynamic p*K*_a_ values of amino acids measured using CSI

Amino acid	p*K*_1,0_	p*K*_2,0_	p*K*_3,0_	pH range^a^
Histidine	1.39 ± 0.14 (1.56)	6.12 ± 0.12 (6.00)	9.28 ± 0.04 (9.25)	2.0–11.1
*I* = 0.12 M	Δ*z*^2^ = 3	Δ*z*^2^ = 1	Δ*z*^2^ = −1	
Lysine	—	9.12 ± 0.04 (9.09)	10.90 ± 0.04 (10.90)	7.7–11.4
*I* = 0.13 M		Δ*z*^2^ = 1	Δ*z*^2^ = −1	
Tyrosine	—	9.22 ± 0.08 (9.09)	10.59 ± 0.05 (10.69)	6.5–11.2
*I* = 0.13 M		Δ*z*^2^ = −1	Δ*z*^2^ = −3	
Aspartic acid	2.12 ± 0.08 (1.99^b^)	3.98 ± 0.03 (3.89^b^)	—	1.8–6.3
*I* = 0.10 M	Δ*z*^2^ = 1	Δ*z*^2^ = −1		

a pH range accessed in CSI experiment (Fig. 1a).

b Ref. 17. Uncertainties were calculated as described in section S5 of the ESI.†

Negligible variation in these p*K*_a_ values (≤0.05 units) is observed upon repetition of the CSI experiments (section S6†) while acquisition of CSI images ±2 hours around *t*_opt_ causes <0.1 units variation in the fitted p*K*_a_ values (section S4†). ^1^H spectra are provided in section S7 of the ESI.† We note that the accuracy and reproducibility of CSI experiments are comparable to potentiometric titration and consume less compound (section S3.3).

### 3.2. Application to linker group modification

**Fig. 2:**
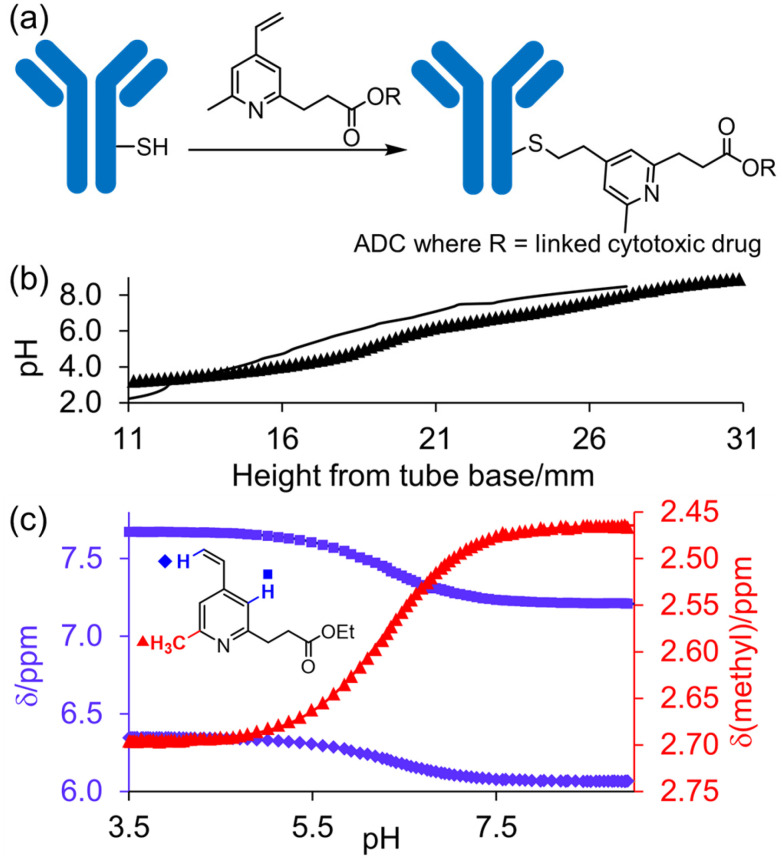
(a) Reaction linking a 4-vinylpyridine derivative to a thiol unmasked antibody. (b) Predicted (solid line) and experimental (cross) pH *versus* position from the base of the NMR tube for determination of p*K*_a_ value of A. (c) Plot of ^1^H chemical shifts of A (2 mM) *versus* pH with fitted p*K*_a,0_ of 6.18 ± 0.09.

The p*K*_a_ value determines the charge and reactivity of a molecule. Nevertheless, the high costs of conventional p*K*_a_ measurements in terms of time, labour and sample quantity can discourage p*K*_a_ determination during molecular design campaigns. Important chemical insights can thus be missed, and time wasted on the synthesis of ineffective compounds. Antibody–drug conjugates (ADCs) are a molecular construct whereby a drug is appended to an antibody for targeted therapeutic treatments, notably oncology.^18^ A reaction is required to attach a linker group to the antibody, with the soft nucleophilic thiol moiety of cysteine being the most targeted functional group due to its chemoselective reactivity with several electrophilic Michael acceptors.^19^ Maleimide is one of the most frequently employed, and is utilised in several clinically approved ADCs, but suffers from deconjugation by retro-Michael reaction.^20^ Selective alkylation of cysteine groups in proteins has been achieved with 2- and 4-vinylpyridines,^21^ and 4-vinylpyridine based PermaLink® is an effective linker unit for ADC development which does not experience reversible addition with thiols (Fig. 2).^22,23^ Related systems developed subsequently include vinylpyrimidines,^24^ divinylpyrimidines (for disulfide rebridging)^25^ and *N*-methylated 2-vinylpyridinium salts.^26^

We sought to synthesise several 4-vinylpyridine derivatives to which can be linked two cytotoxic payloads as a means to improve the drug-antibody ratio (DAR) of a resulting ADC, with this study providing also the opportunity to investigate the relationship between vinylpyridine p*K*_a_ and the rate of a model bioconjugation reaction. Synthesis yielded single-arm compounds A–E, dual-armed compounds F–K containing functionality linked additionally to position 6, and also two heteroatom-substituted compounds L and M for additional reactivity comparison (Table 3).

**Table 3:** p*K*_a_ and rate analysis of substituted 4-vinylpyridines

Entry	R^1^	R^2^	*k*_obs_/M^−1^ min^−1^	p*K*_a,0 NMR_	p*K*_a,0 Epik_	p*K*_a, MolGp*K*_a__	p*K*_a, Jaguar_
A	Me	(CH_2_)_2_CO_2_Et	0.80	6.18 ± 0.09	6.62	5.9	6.40
B		CH_2_CH(CH_2_CO_2_Et)CO_2_Et	0.17	5.69 ± 0.09	6.25	5.5	5.49
C		CH_2_N(CH_2_CO_2_Et)_2_	0.13	5.86 ± 0.07	5.50, 3.91^a^	4.7, 4.6^a^	7.29, 0.95^a^
D		(CH_2_)_2_N(CH_2_CO_2_Et)_2_	0.39	6.69 ± 0.06, 1.4 ± 0.07	6.48, 3.95^a^	6.0, 5.1^a^	7.38, 2.43^a^
E		(CH_2_)_3_N(CH_2_CO_2_Et)_2_	0.75	6.59 ± 0.04, 3.12 ± 0.37	6.87, 4.39^a^	6.3, 5.5^a^	6.75, 3.58^a^
F	(CH_2_)_2_CO_2_Et	(CH_2_)_2_CO_2_Et	0.17	5.36 ± 0.04	5.39	5.2	5.89
G		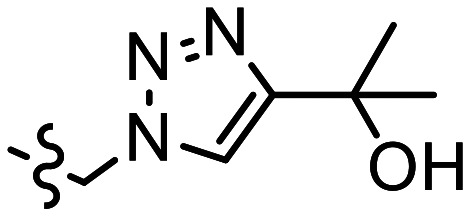	0.0054	2.98 ± 0.05	3.37, 2.84^b^	4.5, 3.0^b^	3.27
H		(CH_2_)NHBoc	0.083	4.74 ± 0.03	4.57	4.7	4.47
I	(CH_2_)NHBoc	(CH_2_)NHBoc	0.087	4.11 ± 0.02	2.55	4.4	5.39
J		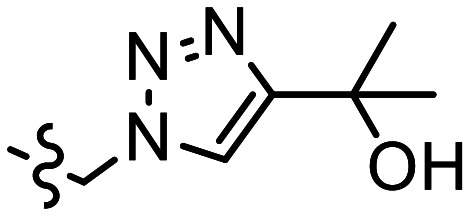	0.0022	2.39 ± 0.02	1.97, 2.84^b^	4.2, 3.2^b^	3.86
K	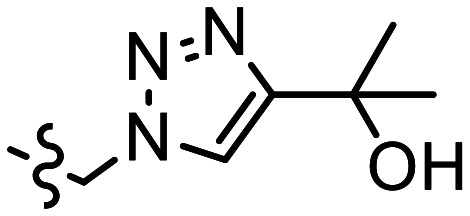	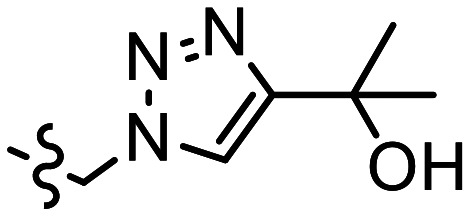	n.d.	0.92 ± 0.15^c^	0.69, 2.84^b^	4.1, 3.1^b^	1.22
L	H	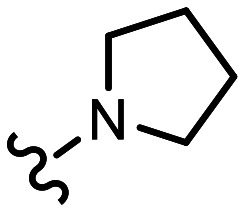	0.084^28^	7.60 ± 0.07	7.35, 7.27^d^	6.7, 5.4^d^	7.60
M		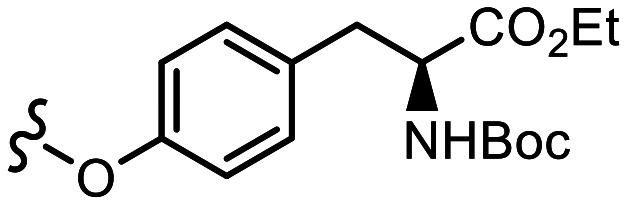	0.0048	2.49 ± 0.49^e^	2.42	3.1	1.62

a p*K*_a,H_ of tertiary amine.

b p*K*_a,H_ of 1,2,3-triazole moiety.

c Additional datapoint measured in homogeneous solution of 0.1 M HCl (Fig. S27†).

d Reported p*K*_a,H_ of pyrrolidine moiety.

e 16 point CSI experiment recorded due to low solubility.


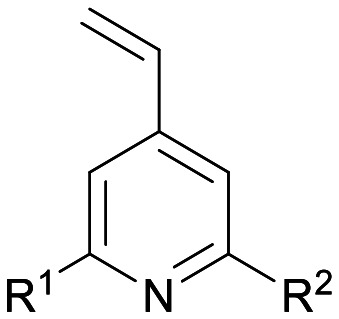
Starting with compound A, we assumed a p*K*_a_ value of approximately 6. We chose a buffer system comprising 20 mM NaHCO_3_, 10 mM tris, K_2_HPO_4_ and acetate. 1.5–2 mg of oxalic acid was predicted to give a gradient between pH 2 and pH 9 with a *t*_opt_ of 8 hours. The p*K*_a_ of twelve other variously substituted 4-vinyl pyridines were then determined. For comparison, micro-p*K*_a_ values were predicted using three computational packages: Jaguar p*K*_a_ (Schrödinger Release 2024-2: Schrödinger, LLC, New York, NY, 2024),^13–15^ Epik version 6.8^12^ and MolGp*K*_a_^11^ (Table 3). The values returned by these calculations were used to guide the selection of the buffer systems for the NMR experiments. For example, the p*K*_a_ of compound J was predicted to be approximately 3 (Table 3). Hence a more acidic buffer system was used relative to A, comprising 20 mM oxalate, 10 mM acetate and NaHCO_3_ and 3 mg of oxalic acid was used. The resulting pH gradient was predicted to span from pH 1.5 to 7 with a *t*_opt_ of 6 hours. Example predicted and experimental gradients for each compound are provided in section S8 of the ESI.†

For compounds D and E additional p*K*_a_ values of 1.4 and 3.1 were obtained that can be associated with the tertiary amine functionality based on the pH-dependence of the chemical shifts of different parts of the molecule (section S9, ESI†). A p*K*_a_ value of 3.3 was determined for the analogous compound PhCH_2_N(CH_2_CO_2_Et)_2_, confirming the low basicity of the tertiary amine (Fig. S30, ESI†). DFT calculations confirmed a higher basicity of the pyridine in compounds C–E (section S10, ESI†). The p*K*_a_ values of the amine sites presented in Table 3 (Jaguar p*K*_a_) were calculated explicitly with the pyridine in the protonated state and are thus closer to the experimentally observed macroscopic values. However, the p*K*_a_ values returned by MolGp*K*_a_^11^ and Epik 6.8^12^ consider the ionisation of each site in isolation (micro-p*K*_a_) without regard for the effect of the ionisation of neighbouring groups. The p*K*_a_ values reported by these methods for the amine sites are thus higher than the second (lower) macroscopic p*K*_a_ observed. That no additional values were obtained for the triazole containing compounds (G, J and K) is consistent with the low basicity of this heterocycle (p*K*_a_ for 1-methyl-1,2,3-triazole 1.25).^27^ However, the p*K*_a_ values of the triazole moiety calculated using Epik and MolGp*K*_a_ are significantly higher, again highlighting the limitations of these predictors when applied to compounds that have more than one protonation site. The 2-pyrrolidinopyridine component of L may be regarded as a single functional group, giving this compound the highest basicity of the compounds studied.

**Scheme 1:**
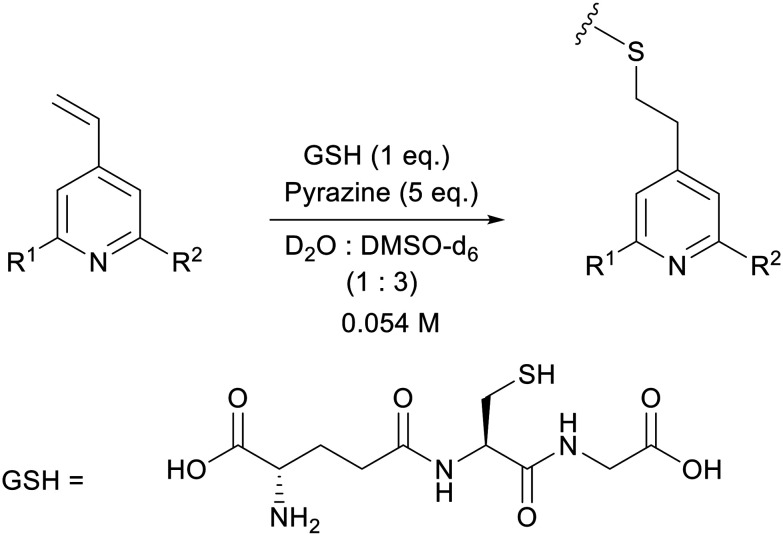
Reaction of 4-vinylpyridines with glutathione (GSH) for the determination of rate as a function of R^1^ and R^2^.

As a model bioconjugation reaction we employed a reaction of each vinylpyridine with glutathione (GSH, Scheme 1). This cysteine containing tripeptide is a non-volatile crystalline solid making it relatively easy to handle, and it has been used previously as a model for reactivity studies on electrophilic covalent inhibitors of cysteine-containing proteins.^28–30^ One equivalent of glutathione was added as a solution in D_2_O (0.22 M) to the vinylpyridine in DMSO-d_6_ (0.073 M), with pyrazine included as an internal standard for integration. The reaction was monitored using ^1^H NMR spectroscopy and the rates of this second order addition^25,26^ are given in Table 3. Synthetic schemes for the preparation of A–M and example kinetic plots are provided in sections S11 and S12 of the ESI,† respectively.

**Fig. 3:**
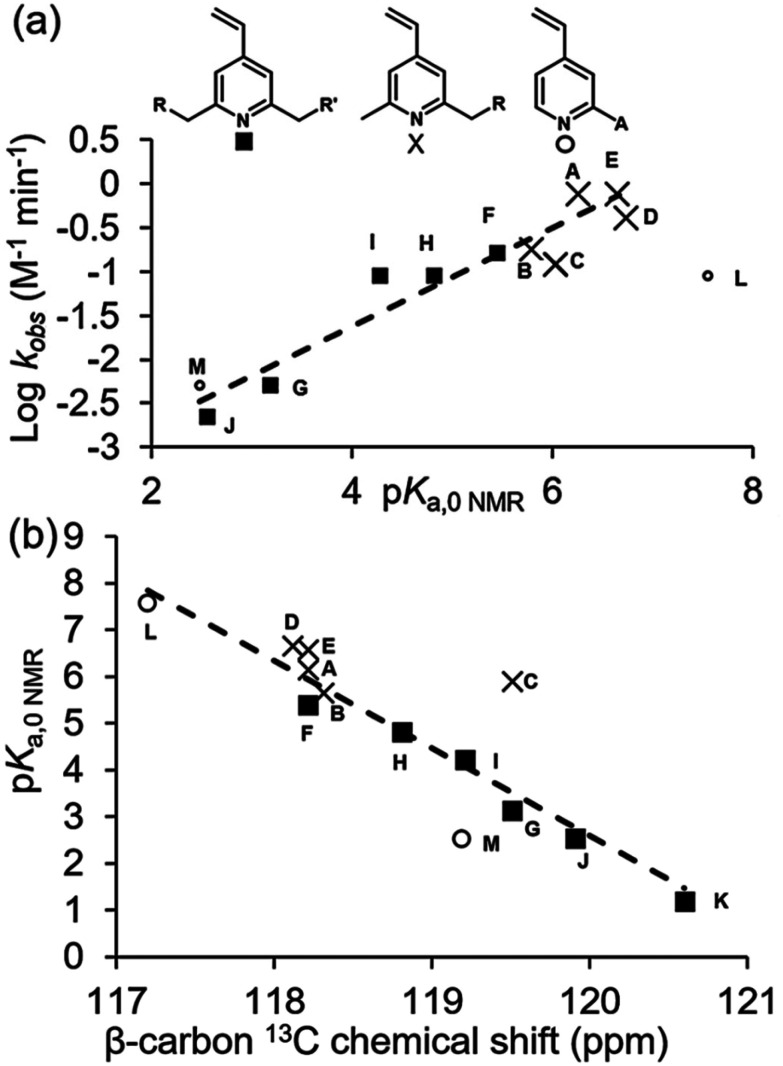
(a) Plot of log *k vs*. p*K*_a,0 NMR_ for 4-vinylpyridine derivatives exhibiting linear correlation. Compound L (Table 3) is plotted separately. (b) Plot of p*K*_a,0 NMR_*versus*^13^C NMR chemical shift (CDCl_3_) of β-carbon of vinyl group.

A plot of the log of rate *versus* the p*K*_a_ value determined by NMR exhibits a strong positive correlation (Fig. 3a) consistent with a lowering of the activation energy barrier and an acceleration of reaction rate when the pyridine nitrogen bears a positive charge, as reported for the reaction of *N*-acetylcysteineamide with quaternized 2-vinylpyridine.^26^ Three different chain lengths were used to attach an iminodiacetate unit to the 2-position [(CH_2_)_*n*_, where *n* = 1–3, entries C–E] and an increase in chain length results in an increase in rate such that with a three-carbon spacer the electronegativity of the nitrogen in E is essentially negated. The other electron-withdrawing functional groups are attached with either a two-carbon (B) or one-carbon spacer (G and H) which significantly diminishes the rate, especially when attached to both positions 2 and 6 (I–K). Similarly, replacement of the methyl group in A with an ester functionality reduces the reactivity (F). The conjugated 2-pyrrolidine nitrogen in L significantly decreases the reactivity relative to A, despite L being the most basic of all the compounds studied as it is now too electron rich to react at a significant rate. The 2-oxygen substituent of M significantly reduces pyridine basicity, shutting down reactivity. Thus, a functionalised alkyl chain is the optimum substituent such that compounds A, D and E are in the ‘Goldilocks zone’ with respect to pyridine basicity and the enhanced electrophilicity of the protonated species.

To confirm that protonation of the pyridines took place under the conditions of the reaction, the ^1^H chemical shift of the methyl resonance of A was measured in D_2_O/DMSO-d_6_ solutions containing 20 mM HCl (A fully protonated, 2.6074 ppm) and in the absence of acidic species (A non-protonated, 2.3548 ppm). Comparison to the chemical shift measured in the presence of GSH (2.3732 ppm) revealed 7 ± 2% of A was protonated in the presence of GSH under the conditions of the reaction.^31^ A full discussion of this calculation, including uncertainty, is provided in section S12 of the ESI.† We note that due to the acidic nature of GSH, an appreciable percentage protonation (>3%) is anticipated for vinylpyridines with p*K*_a,0 NMR_ > 5.3, while the pH is expected to be weakly basic (Fig. S38†). An acceleration of the rate of reaction of *N*-acetylcysteineamide with 2-vinylpyridine has been reported under weakly acidic conditions in H_2_O (pH 5.7) relative to neutral buffer (pH 7.6).^26^ The acidity of GSH therefore aids the reaction under our experimental conditions, with a higher degree of protonation and thus a faster rate of reaction observed for the more basic vinylpyridines.

The correlation between the ^13^C NMR chemical shift of the β-carbon of *para*-substituted styrenes and the Hammett substituent constant (*σ*_p_) has previously been noted.^32^ Accordingly, a correlation is observed between the p*K*_a_ values of A–M and the ^13^C NMR chemical shift of the electrophilic β-vinyl carbon (Fig. 3b). This correlation suggests that ^13^C NMR can be used to predict the p*K*_a_ values of substituted 4-vinylpyirindes and thus estimate their reactivity towards cysteine bioconjugation.

## 4. Conclusions

In conclusion, a rapid and operationally simple method for the p*K*_a_ determination of organic molecules in buffered aqueous media has been developed for a wide range of pHs (*ca.* 2–11). As proof of principle, the thermodynamic p*K*_a_ values of four amino acids were ascertained using this method to obtain values closely matching literature values. As demonstration of application, the p*K*_a_ values of a range of substituted 4-vinylpyridine linker compounds were also determined which revealed a strong correlation between p*K*_a_ and reaction rate. We are also able to rationalise other correlations, such as between observed ^13^C NMR chemical shifts and rates of reactions. Our approach makes experimental measurements of p*K*_a_ accessible and feasible to perform during the routine analysis of newly synthesised organic compounds by NMR.

## Author contributions

MW, TES and CJR conceived the project. MW and MN designed and performed the CSI experiments. JS prepared compounds A–M and performed the kinetic analysis, under the supervision of JT, PLKH, GRS, CJR and TES. KB performed the computational calculations of p*K*_a_ under the supervision of JAI. MW, TES and CJR wrote the manuscript, with suggestions from all other authors.

## Conflicts of interest

There are no conflicts to declare.

## Supplementary Material

AN-150-D5AN00406C-s001

AN-150-D5AN00406C-s002

## Data Availability

Additional data supporting this article (experimental procedures, synthetic details, stacked NMR plots) are available in the ESI.† A spreadsheet for prediction of pH gradients and processing of CSI data is also provided as ESI.† Raw CSI data files will be openly available at: https://research-portal.uea.ac.uk/en/datasets/.
