# Supplementary material for: Programmable wide-range pH gradients for NMR titrations: application to antibody–drug conjugate linker group modifications
Source: Analyst. 2025 May 26;150(13):2872–9. doi: 10.1039/d5an00406c (PMC12128038; doi:10.1039/d5an00406c)
Supplement: AN-150-D5AN00406C-s001 [file AN-150-D5AN00406C-s001.pdf]

Supplementary Information (SI) for Analyst.
This journal is © The Royal Society of Chemistry 2025

Supporting information: Programmable wide-range pH gradients for NMR titrations

Matthew Wallace,[a]* James M. Sharpe, Krzysztof Baj,[b] Michael Ngwube,[a] Jenny Thirlway,[c] Patrick L. K. Higgs,[c] G. Richard Stephenson,[a] Jonathan A. Iggo,[b] Thomas E. Storr[a] and Christopher J. Richards[a]*

[a]School of Chemistry, Pharmacy and Pharmacology, University of East Anglia, Norwich Research Park, Norwich, NR4 7TJ, UK
[b]Department of Chemistry, University of Liverpool, Crown Street, Liverpool, L69 7ZD, UK
[c]Iksuda Therapeutics Ltd., The Biosphere, Draymans Way, Newcastle Helix, Newcastle upon Tyne, NE4 5BX, UK
Corresponding author: matthew.wallace@uea.ac.uk

Contents

S1. Additional experimental details for pK_a determination by NMR 2
S1.1 Limiting chemical shifts of NMR pH indicators and pK_a fitting constraints 2
S1.2 Finding midpoint and effective range of image by analysis of biphasic samples 3
S2. Prediction of pH gradient based on mass of oxalic acid and buffer composition 4
S3. Spreadsheet calculation of pH by ^{1}H NMR, calibration of NHS as indicator and benchmarking against potentiometric titration 9
S3.1 Spreadsheet calculation of pH 9
S3.2 Calibration of NHS as pH indicator 9
S3.3 Benchmarking of CSI method against potentiometric titration 10
S4. Evolution of pH gradient used to determine pK_a of amino acids with time 12
S5. Estimation of uncertainty in fitted pK_a values 14
S6. Reproducibility of pH gradients and pK_a measurements used to determine pK_a of amino acids 15
S7. CSI datasets used to determine pK_a of amino acids (Table 2) 17
S8. Example predicted and experimental pH gradients used to determine pK_a of compounds **A** – **M** 21
S9. CSI datasets used to determine pK_a of compounds **A** – **M** and diethyl benzyliminodiacetate 21
S10. Prediction of pK_a using Jaguar pK_a 29
S11. Methods for preparation of 4-vinylpyridines **A** – **M** and ^{1}H and ^{13}C NMR spectra 31
S12. Example kinetic data for reaction of **F** with glutathione and protonation analysis 146
S13. References 150
S14. Processing routines, scripts and pulse programs 152
14.1 Processing routine for Mnova 14.3.1 152
14.2 Scripts to acquire and process CSI datasets (Bruker Topspin) 157
14.3 Pulse programs for CSI experiments (Bruker Topspin) 171

S1. Additional experimental details for pK_a determination by NMR

S1.1 Limiting chemical shifts of NMR pH indicators and pK_a fitting constraints

Chemical shifts of the limiting protonated (δ_H) and deprotonated (δ_L) forms of the indicators are provided in Table S1.

Table S1. pK_a, limiting chemical shifts and ionic strength correction parameters for NMR pH indicators used in this study

**Indicator**	**p$K_{a,0}$**	**δ_L/ppm[a]**	**δ_H/ppm[a]**	**Δz^2**
DCA	1.35	6.0480	6.3118	-1
$MPAH^-$	2.38	1.2819	1.5106	-1
Formate	3.75	8.4414	8.2669	-1
Acetate	4.76	1.9060	2.0830	-1
2,6-lutidine	6.75	2.4563	2.7074	1
NHS[b]	6.11	2.6386	2.7812	-1
MPA^{2-}	7.99	1.0711	1.2819	-3
Glycinate	9.78	3.1754	3.5494	-1
Methylamine	10.66	2.2901	2.5942	1

[a]Referenced to DSS (0 ppm). [b]*N*-hydroxysuccinimide, Section S3. All other pK_a values and limiting chemical shifts are taken from Reference 51.

The pH is determined for each indicator using Equation S1:

$$\mathrm{pH} = \mathrm{p}K_{a,0} + \Delta z^2\left[0.51\frac{\sqrt{\mathrm{I}}}{1+\sqrt{\mathrm{I}}} - 0.1\mathrm{I}\right] + \log_{10}\left(\frac{\delta_H - \delta_{obs}}{\delta_{obs} - \delta_L}\right) \quad \text{S1}$$

where I is the ionic strength of the solution.

S1.2 Finding midpoint and effective range of image by analysis of biphasic samples

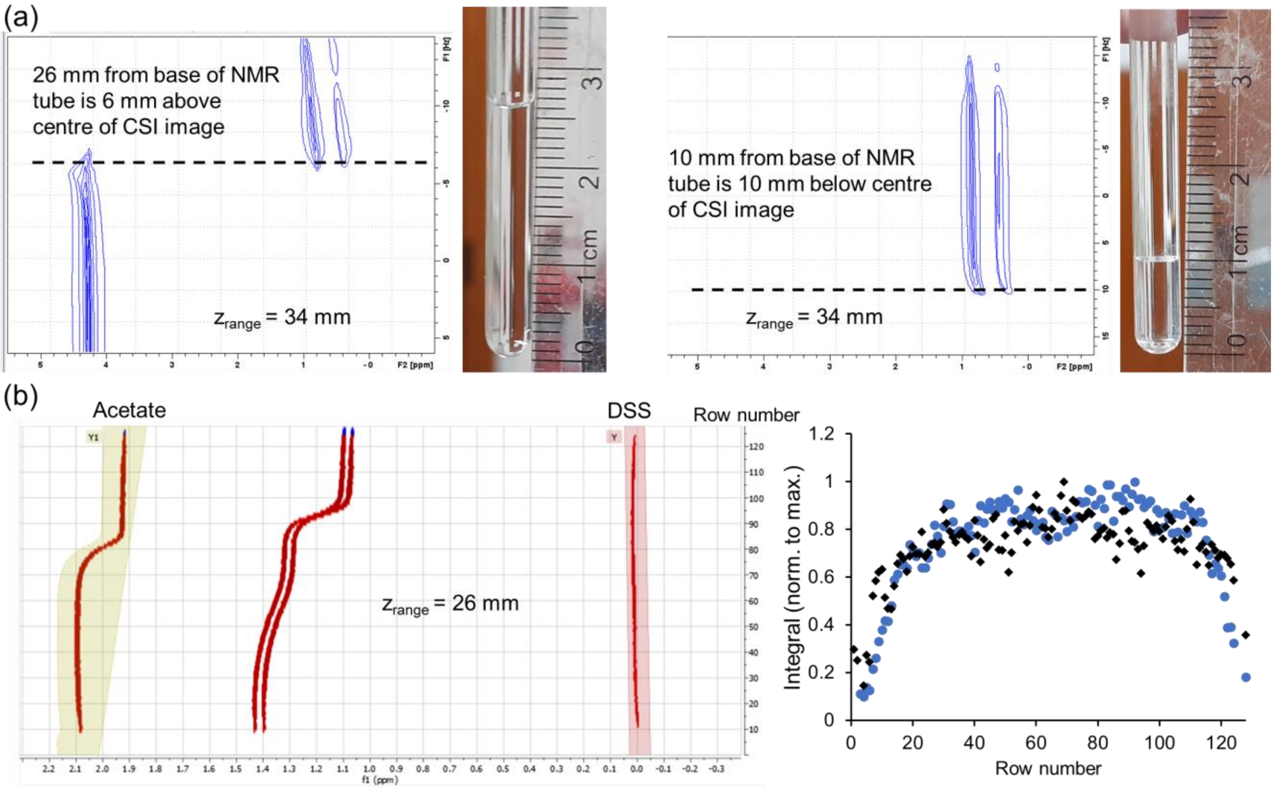

Figure S1. (a) Measurement of position of centre of CSI image through analysis of biphasic sample comprising mineral oil layered on top of 0.5 M sodium acetate solution (H_2O). Vertical scale on CSI plots is 1 Hz/mm. The meniscus is taken as the position on the CSI plot where the mineral oil signals (δ < 2 ppm) finishes. Centre of CSI image is 20 mm from tube base. These experiments were recorded off lock using a spin-echo sequence without water suppression (Section S14.2) with a 10 μs pulse (π/2). The vertical window of the CSI experiment (cnst0) was set to 3.4 cm. The phase encoding gradient pulse (200 μs) was a smoothed square and ramped from -24.8 to 24.8 G/cm in 128 steps, giving a theoretical resolution of 0.27 mm. (b) Example CSI dataset used to determine pK_a. 2D overview plot (left) and integral of acetate (blue circle) and DSS (black diamond) normalised to largest absolute integral measured in series. Useable spectra for pK_a determination are obtained between rows 20 and 120, with cnst0 set to 2.6. The vertical size of the CSI image is greater than the range of NMR signal to avoid folding artefacts.[54]

S2. Prediction of pH gradient based on mass of oxalic acid and buffer composition

Assuming fast dissolution of the oxalic acid, the concentration, C, at any vertical position, Z, along the tube at time, t, after preparation is given by Equation S2.[51]

$$C_Z = \frac{m}{\pi r^2 Mr\sqrt{\pi D t}} \exp\left[{-(Z-h)^2}/{4Dt}\right] \quad \text{S2}$$

D is the diffusion coefficient of oxalic acid, $1.2\text{x}10^{-9}$ m^2s^{-1},[51] m the mass of oxalic acid, r the tube radius, Mr the molecular mass of oxalic acid and h the thickness of the tube bottom and oxalic acid layer (2 mm). The NMR-active window of our sample on our spectrometer extends from 11 mm to 31 mm from the absolute base of the 5 mm NMR tube (Figure 1a). These limits were determined by analysis of biphasic samples (Section S1.2). To design a pH gradient, the user inputs trial concentrations of oxalic acid at 25 mm (Z_{top}) and 11 mm (Z_{bottom}) from the base of the tube and the spreadsheet will predict the resultant pH gradient based on a trial composition of the buffer system. The time, t_{opt}, required for this gradient to develop is calculated using Equation S3:[51]

$$t_{opt} = \frac{(Z_{bottom}-h)^2 - (Z_{top}-h)^2}{4D\ln\left(\frac{C_{top}}{C_{bottom}}\right)} \quad \text{S3}$$

where C_{top} and C_{bottom} are the concentrations of oxalic acid at Z_{top} and Z_{bottom}, respectively, after diffusion up the NMR tube. The user adjusts C_{top}, C_{bottom} and the buffer composition until the predicted pH gradient spans the desired range. We advise setting the lower limit of the pH gradient to a value significantly below the expected pK_a of the analyte. For example, for lysine and tyrosine (pK_a > 9) the lower limit of the predicted pH gradient is set <6 and reproducible pK_a values are obtained (Section S6). For simplicity, we assume that the buffer species remain at fixed concentration throughout the sample. A sample is prepared according to m and the buffer composition. The sample is then placed in an automatic sample changer and a CSI experiment performed at $t_{opt}\pm2$ hours after preparation. The spreadsheet also predicts the ionic strength, I, of the solution. The buffers used in this work and their pH ranges are provided in Table 1.

Oxalic acid appears as a diprotic strong acid to buffer components possessing p$K_{a,H}$ values greater than the 2nd pK_a value of oxalic acid (4.0). Once a buffer has been completely neutralised by oxalic acid, it can no longer control the pH and the pH is controlled by buffers with lower p$K_{a,H}$ values. To calculate the pH in a vertical slice along the NMR tube, C_z is calculated using Equation S2 and the oxalic acid allocated to buffers in order of decreasing p$K_{a,H}$ value. The pH is then calculated based upon the buffer which is only partially protonated – the active buffer. However, the oxalic acid will also be consumed slightly by buffers with p$K_{a,H}$ values below that of the active buffer. The lower buffer is defined as the buffer component with the highest p$K_{a,H}$ value that is below that of the active buffer. The fraction of acid reallocated to the lower buffer, f_L, is calculated using Equation S4:

$$f_L = \frac{\frac{2L}{A}10^{(pK_{a,H\,L}-pK_{a,H\,A})}}{1+\frac{2L}{A}10^{(pK_{a,H\,L}-pK_{a,H\,A})}} \quad \text{S4}$$

where L and A are the total concentrations of lower and active buffer respectively. The factor 2 accounts for the common case when the active buffer is 50% protonated and there is one unit difference in pK_a between the lower and active; if L = A the lower buffer would be 10% protonated. The fraction of acid allocated to the active buffer is given by 1-f_L. A pH is calculated for both buffers separately using Equation S5 and the overall pH of the solution is reported as the arithmetic mean of the pH calculated from the two indicators:

$$\mathrm{pH} = \mathrm{pK_{a,H}} + \log_{10}\left(\frac{\mathrm{B} - 2\mathrm{C_{Buff}}}{2\mathrm{C_{Buff}}}\right) \qquad \text{S5}$$

where B is the total concentration of the buffer (B = A or L depending on assignment of buffer species) and C_{Buff} the concentration of oxalic acid allocated to it. Equation S5 is used to calculate the pH for all buffers other than oxalate.

As the pK_a of the buffer approaches the 2nd pK_a of oxalic acid (4.0), the fraction of the concentration of a buffer which acts as a strong base towards oxalic acid is reduced. The effective concentration of a buffer, B_{Eff}, is given by Equation S6:

$$\mathrm{B_{Eff}} = \mathrm{B}\frac{10^{(\mathrm{pK_{a,H}}-4)}}{1 + 10^{(\mathrm{pK_{a,H}}-4)}} \qquad \text{S6}$$

where the p$K_{a,H}$ value is that of the buffer. When the pH approaches 4.0, the oxalate generated by the higher buffers as well as any added to the sample begins to act as a buffer. If the $pK_{a,H}$ of the active buffer is < 4, the fraction of oxalic acid diffusant reallocated to oxalate from the active buffer is calculated using Equation S7:

$$\mathrm{f_L} = \frac{\frac{2\mathrm{Ox_{Buff}} + \sum \mathrm{B_{Eff}}}{\mathrm{A}}10^{(4-\mathrm{pK_{a,H}})}}{1 + \frac{2\mathrm{Ox_{Buff}} + \sum \mathrm{B_{Eff}}}{\mathrm{A}}10^{(4-\mathrm{pK_{a,H}})}} \qquad \text{S7}$$

where Ox_{Buff} is additional oxalate which has been added as a buffer and ΣB_{Eff} is the sum of the effective concentrations of all buffers with p$K_{a,H}$ values greater than 4.0. Once the other buffers have been fully allocated, oxalate itself becomes the active buffer. A pH is calculated based upon the ratio of oxalate and monohydrogen oxalate in the sample (Equation S8):

$$\mathrm{pH} = 4 + \log_{10}\left(\frac{\frac{\sum \mathrm{B_{Eff}}}{2} + \mathrm{Ox_{Buff}} - \mathrm{C_{Buff}}}{2\mathrm{C_{Buff}}}\right) \qquad \text{S8}$$

C_{Buff} is the concentration of oxalic acid diffusant allocated to oxalate buffer. This Equation is used until the reported pH falls to 3.

Below pH 3, the following procedure is adopted: Firstly, the total concentration, T, of oxalic acid at the position with the lowest pH, but with pH > 3, is calculated using Equation S2. This yields the total concentration of monohydrogen oxalate at lower positions down the tube (pH < 3), generated by the reaction of oxalic acid with the pH buffers. The pH at positions further down the tube is then calculated from C_Z at these lower positions using Equation S9:

$$\mathrm{pH} = -\log_{10}\left(\frac{-(\mathrm{T} + 10^{-1.2}) + \sqrt{(\mathrm{T} + 10^{-1.2})^2 + 4(\mathrm{C_Z} - \mathrm{T})10^{-1.2}}}{2}\right) \qquad \text{S9}$$

Equation S9 is equivalent to the calculation of the pH of a solution when monohydrogen oxalate (of concentration T) is mixed with oxalic acid (of concentration C_z - T), with the pK_a of monohydrogen oxalate assumed as 1.2. The pH calculation based upon Equations S4-S8 is most accurate when the pH is close to the p$K_{a,H}$ of a buffer. In the attached spreadsheet, calculated pH values greater than 3 are automatically rejected where they lie more than 0.5 units away from the p$K_{a,H}$ of the active buffer. Below pH 3, the pH is calculated using Equation S9.

When the active buffer is hydroxide, the free concentration of hydroxide must be corrected for buffer components with very weakly acidic protons in their deprotonated (basic) forms (K_2HPO_4 and $NaOB(OH)_2$ in this work). If the concentration of these buffer components is less than the total concentration of hydroxide, the pH is calculated using the following procedure: Firstly, an approximate pH, pH′, is calculated based on the total concentration of oxalic acid and sodium hydroxide present:

$$\mathrm{pH'} = -\log_{10}\left(\frac{10^{-\mathrm{pK_w}}}{[\mathrm{NaOH}]_\mathrm{t} - 2\mathrm{C_{Buff}}}\right) \qquad \text{S10}$$

where pK_w = 14, C_{Buff} is the concentration of oxalic acid assigned to hydroxide using the procedure described above and $[NaOH]_t$ the total amount of sodium hydroxide added. The concentration of deprotonated buffer, [D], (PO_3^{3-} or $B(OH)O_2^{2-}$) is then calculated from Equation S11:

$$[\mathrm{D}] = -\log_{10}\left(\frac{10^{\mathrm{pH'}-\mathrm{pK_{a,upper}}}}{1 + 10^{\mathrm{pH'}-\mathrm{pK_{a,upper}}}}\right) \qquad \text{S11}$$

where p$K_{a,upper}$ is the pK_a of the weakly acidic proton. The pH of the sample is then calculated from Equation S12:

$$\mathrm{pH} = -\log_{10}\left(\frac{10^{-\mathrm{pK_w}}}{[\mathrm{NaOH}]_\mathrm{t} - 2\mathrm{C_{Buff}} - \sum[\mathrm{D}]}\right) \qquad \text{S12}$$

where the summation is carried out over all buffer components with weakly acidic protons.

If the concentration of buffer components with weakly acidic protons is greater than the total concentration of hydroxide added to the sample, the free concentration of hydroxide, $[OH^-]_f$, is calculated from Equation S13:

$$[\mathrm{OH^-}]_\mathrm{f} = \frac{-10^{-\mathrm{pK_{a,av}}}\left(\mathrm{B_{up,t}} - [\mathrm{NaOH}]_\mathrm{t} + 2\mathrm{C_{Buff}} + \mathrm{K_W}\right) + \sqrt{10^{-2\mathrm{pK_{a,av}}}\left(\mathrm{B_{up,t}} - [\mathrm{NaOH}]_\mathrm{t} + 2\mathrm{C_{Buff}} + \mathrm{K_W}\right)^2 + 4\mathrm{K_W}\left([\mathrm{NaOH}]_\mathrm{t} - 2\mathrm{C_{Buff}}\right)10^{-\mathrm{pK_{a,av}}}}}{2\left(10^{-\mathrm{pK_{a,av}}}\right)} \qquad \text{S13}$$

where C_{buff} is the concentration of oxalic acid diffusant assigned to hydroxide and p$K_{a,av}$ is the average pK_a of the weakly acidic protons of the buffer components calculated using Equation S14:

$$\mathrm{pK_{a,av}} = \frac{\sum_{i=1}^{n} \mathrm{BpK_{a,upper}}}{\mathrm{B_{up,t}}} \qquad \text{S14}$$

where $B_{up,t}$ is the total concentration of buffer components with weakly acidic protons and the summation is carried over all these species. The pH is then calculated using Equation S15, provided

the approximate pH estimated using Equation S10 is less than 11. Otherwise, the pH is reported as the higher of the pH values calculated using Equation S10 and S15.

$$pH = -\log_{10}\left(10^{-pK_w}/[OH^-]_f\right) \qquad S15$$

The ionic strength along the gradient is calculated by summing the contribution of each ionic species:

The contributions to the overall ionic strength from OH^-, I_{OH}, and H^+, I_H, are calculated using Equation S16 and S17:

$$I_{OH} = 0.5x10^{pH-pK_w} \qquad S16$$

$$I_H = 0.5x10^{-pH} \qquad S17$$

The contribution from buffer species, I_B, with charge, Z_B, in their deprotonated state are calculated using Equation S18:

$$I_B = 0.5Z_B{}^2B\frac{10^{(pH-pK_{a,H})}}{1+10^{(pH-pK_{a,H})}} \qquad S18$$

The contribution from oxalate, I_{ox}, is calculated using Equation S19:

$$I_{ox} = 0.5(C_Z + Ox_{Buff})\left[\frac{2+0.5\times10^{(4-pH)}}{1+10^{(4+1.2-2pH)}+10^{(4-pH)}}\right] \qquad S19$$

If it is assumed that only monovalent counterions are present in the sample at concentration C_c, the overall ionic strength is computed using Equation S20:

$$I = I_{OH} + I_H + I_B + I_{ox} + 0.5C_c \qquad S20$$

where C_c is calculated using Equation S21:

$$C_c = \sum B\sqrt{z_L{}^2} \qquad S21$$

where z_L is the charge of the buffer species in its non-protonated form, as added during preparation of the sample in the absence of oxalic acid. The summation is carried out over all buffer components added to the sample.

Figure S2 compares the pH and ionic strength calculated using the spreadsheet for a range of buffer systems with the pH and ionic strength calculated explicitly using the CurTiPot package.[50] Good agreement (< 0.5 units) is generally obtained for all buffer systems. This level of accuracy is adequate for prediction of the pH ranges accessible using different buffer systems.

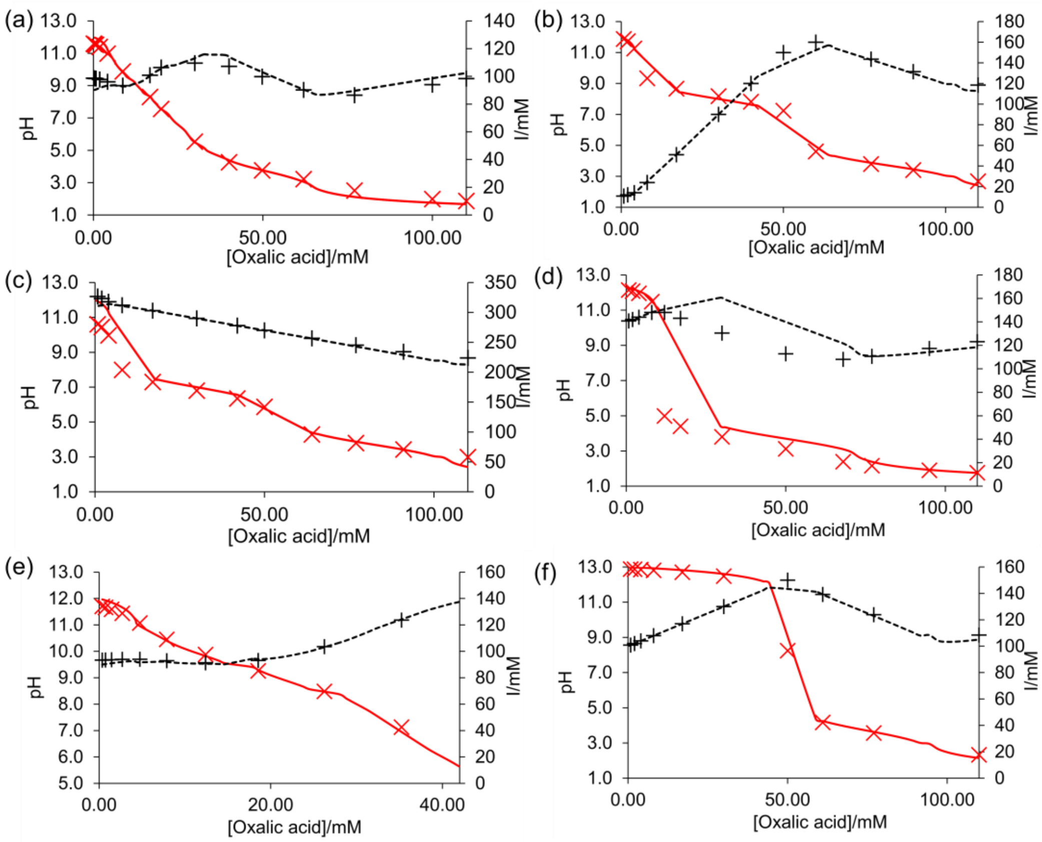

Figure S2. Plots of pH (red) and ionic strength (black) versus concentration of oxalic acid calculated using the spreadsheet accompanying this work (line) and using the CurTiPot[50] package (cross) in different buffer systems: (a) 0.01 M each of NaOH, Na_2CO_3, $NaOB(OH)_2$, tris, K_2HPO_4 and acetate; (b) 0.01 M NaOH and 0.1 M tris; (c) 0.01 M NaOH and 0.1 M K_2HPO_4; (d) 0.02 M NaOH and 0.04 M Na_2Oxalate; (e) 0.01 M NaOH, 0.02 M each of Na_2CO_3, $NaOB(OH)_2$ and tris; (f) 0.1 M NaOH.

S3. Spreadsheet calculation of pH by ^{1}H NMR, calibration of NHS as indicator and benchmarking against potentiometric titration

S3.1 Spreadsheet calculation of pH

The spreadsheet accompanying this work calculates the pH based on the ^{1}H chemical shifts of all indicators. Indicators are excluded from the calculation of pH if δ_{obs} is within 0.005 ppm of δ_H or δ_L, or outside of these values (Table S1). An apparent pH is calculated from each remaining indicator using Equation S1. The sensitivity, S, of an indicator is defined as the first derivative of the indicator chemical shift with respect to pH. S can be obtained from Equation S1 as:[51]

$$S = (\ln 10)\left[\frac{(\delta_L - \delta_{obs})(\delta_{obs} - \delta_H)}{\delta_H - \delta_L}\right] \qquad \text{S22}$$

To determine the pH of the row of the CSI dataset, the indicators are grouped in pairs: methylamine/glycinate, glycinate/MPA^{2-}, MPA^{2-}/2,6-lutidine (or MPA^{2-}/NHS for analysis of 4-vinylpyridine derivatives), 2,6-lutidine (or NHS)/acetate, acetate/formate, formate/$MPAH^{-}$ and $MPAH^{-}$/DCA. If a smaller set of indicators is used, indicators should still be grouped into pairs and arranged in sequential columns in the spreadsheet. The pH is calculated from the pair with the highest combined sensitivity. The pH is obtained as the average pH reported by each indicator in the pair, weighted by S. This procedure is performed automatically by the spreadsheet accompanying this work. The parameters of the NMR indicators used in this work are provided in Table S1. A single representative ionic strength is used for all rows of the CSI dataset, taken as the average of the maximum and minimum ionic strength along the gradient calculated from the buffer composition (Figure S2), plus the ionic strength of the indicators (20 mM for all datasets). We note that if I ≈ 0.1 M, negligible variation in $pK_{a,0}$ is obtained with a ±0.03 M variation in ionic strength (Figure S3).

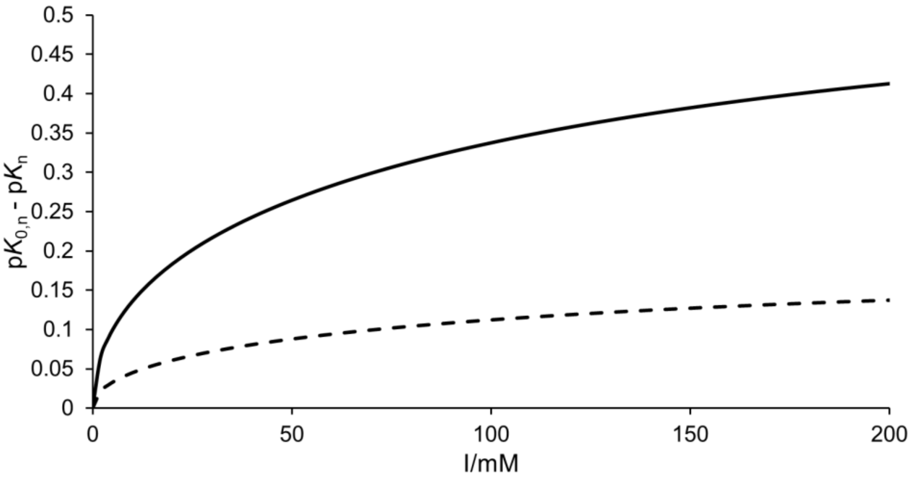

Figure S3. Plot of $pK_{0,n} - pK_n$ versus I for Δz^2 = -1 (dashed) and -3 (solid line), calculated using Equation 2.

S3.2 Calibration of NHS as pH indicator

To determine the pK_a, δ_H and δ_L of NHS (Table S1), a sample was prepared containing 10 mM each of $NaOB(OH)_2$, tris, K_2HPO_4, $NaHCO_3$ and acetate-d_3 as buffers. 2 mM MPA, glycinate, acetate and NHS were included to measure the pH. The sample also contained

2,4,6-trimethylpyridine (1 mM). This solution was layered on top of 1.3 mg oxalic acid and stood for 8 bours before analysis by CSI. The pH was determined from the ^{1}H chemical shifts of acetate, MPA and glycine. Fitting to Equation 1, δ_H and δ_L were obtained as given in Table S1, p$K_{a,0}$ was obtained as 6.11 in good agreement with a literature value of 6.0.[52] Fits are shown on Figure S4.

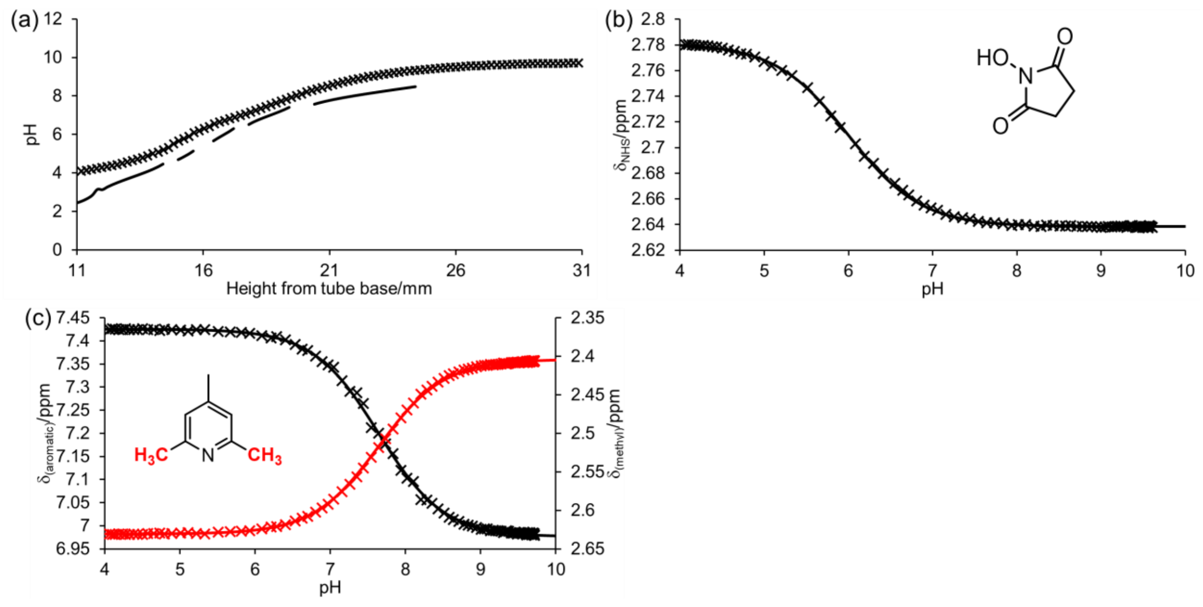

Figure S4. (a) Measured (cross) and predicted (line) pH gradient used to determine pK_a value of NHS and 2,4,6-trimethylpyridine. (b) Plot of ^{1}H chemical shift of NHS versus pH (cross) and fit to Equation 1 (solid line). (c) Plot of ^{1}H chemical shift of (2,6) methyl groups (red) and aromatic protons (black) of 2,4,6-trimethylpyridine and fit (solid line) versus pH.

S3.3 Benchmarking of CSI method against potentiometric titration

The p$K_{a,0}$ of 2,4,6-trimethylpyridine was obtained as 7.51±0.04 by CSI (Figure S4). Literature p$K_{a,0}$ values of this compound determined by potentiometric titration are provided in Table S2 and span the range 7.47 to 7.29, where ionic strength is reported, corresponding to a spread of 0.18 units. The p$K_{a,0}$ values determined by CSI are reproducible to < 0.1 units (Figure S10) and agree with literature values to within 0.2 units (Table 2), suggesting that the CSI method has an accuracy and reproducibility comparable to potentiometric titration. Based on the reported experimental details for potentiometric titrations of 2,4,6-trimethylpyridine where available, the mass of compound consumed in a single potentiometric titration is > 2 mg which contrasts with the < 0.1 mg consumed in the CSI experiment where 700 μL of sample was analysed at a concentration of 1 mM.

Table S2. Comparison of pK_a values of 2,4,6-trimethylpyridine determined by NMR (this work) and potentiometric titration (other references), p$K_{a,0}$ calculated using Equation 2, and calculated mass of 2,4,6-trimethylpyridine consumed based on reported experimental details.

Reference	p$K_{a\ reported}$ at (I/M)	p$K_{a,0}$ (Equation 2)	Mass 2,4,6-trimethylpyridine used/mg
This work	7.62±0.04 (I = 0.1 M)	7.51±0.04	0.1
76	7.45±0.05 (0.005 M)	7.42±0.05	15
77	7.40 (0.1 M)	7.29	No details given
78	7.39 (0.01-0.014 M)	7.35	> 2.4 (assuming > 2 mL titration volume)
53	7.59 (I not reported, no details of titration given)	-	-

S4. Evolution of pH gradient used to determine pK_a of amino acids with time

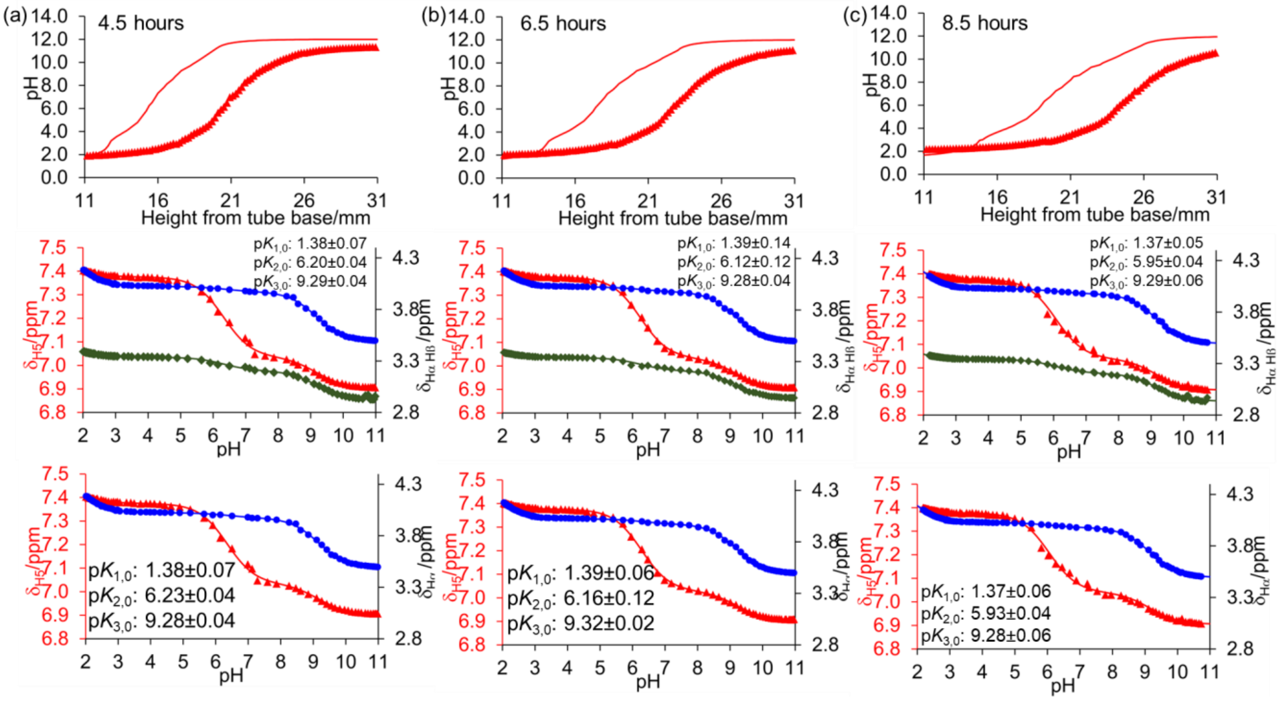

Figure S5. Evolution of Histidine sample of Figure 1b,c with time (t_{opt} = 6.5 hours). Top plots: predicted (solid line) and experimental (red triangle) pH gradient (top). Middle plots: α (blue circle), H_5 proton (red triangle) and downfield β chemical shifts of histidine versus pH. Fits to Equation 1 are solid lines. (a) 4.5 hours after preparation, (b) 6.5 hours (as plotted Figure 1a) and (c) 8.5 hours after preparation. Lower plots: β resonance excluded from fits.

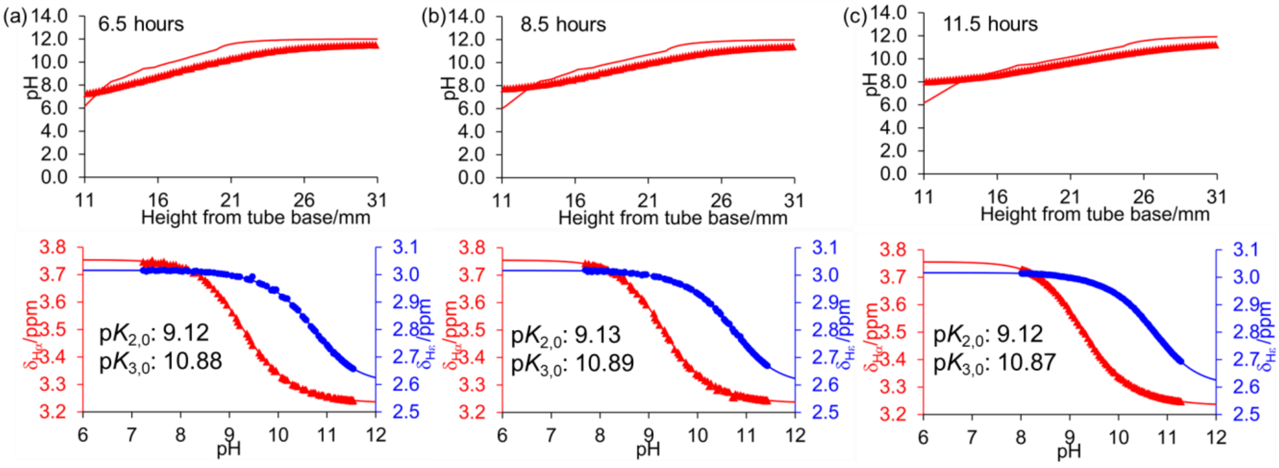

Figure S6. Evolution of lysine sample with time (t_{opt} = 8.5 hours). Top plots: predicted (solid line) and experimental (red triangle) pH gradient (top). Lower plots: α (red triangle) and H_e proton (blue circle) chemical shifts versus pH. Fits to Equation 1 are solid lines. (a) 6.5 hours after preparation, (b) 8.5 hours and (c) 11.5 hours after preparation.

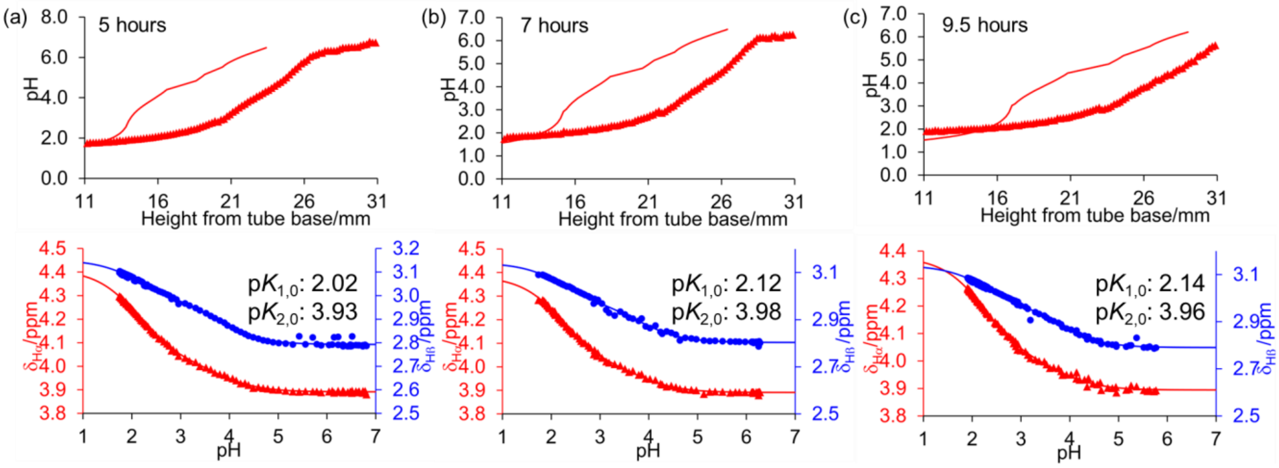

Figure S7. Evolution of aspartic acid sample with time (t_{opt} = 7 hours). Top plots: predicted (solid line) and experimental (red triangle) pH gradient (top). Lower plots: α (red triangle) and β proton (blue circle) chemical shifts versus pH. Fits to Equation 1 are solid lines. (a) 5 hours after preparation, (b) 7 hours and (c) 9.5 hours after preparation.

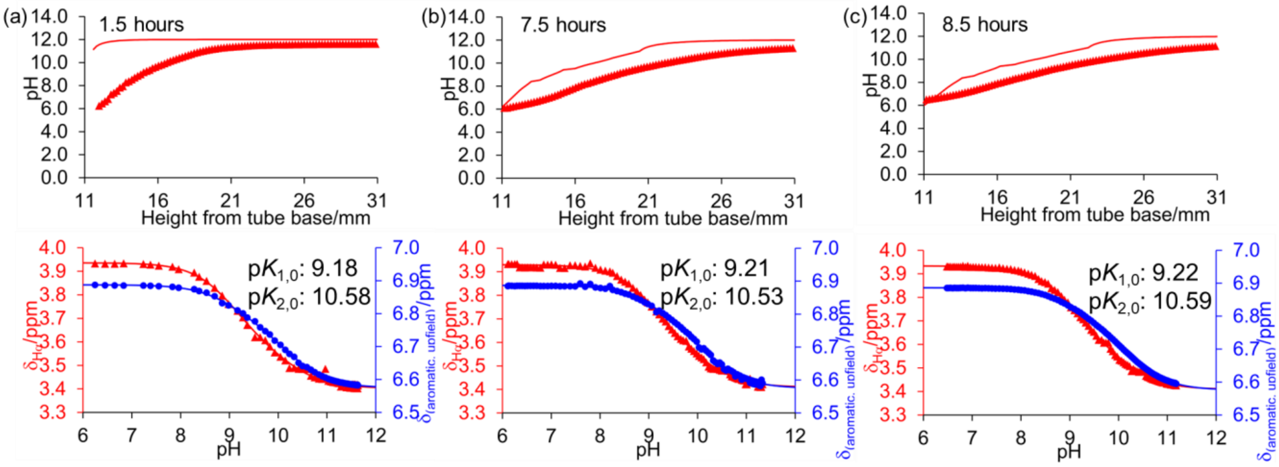

Figure S8. Evolution of tyrosine sample with time (t_{opt} = 8.5 hours). Top plots: predicted (solid line) and experimental (red triangle) pH gradient (top). Lower plots: α (red triangle) and upfield aromatic resonance (blue circle) chemical shifts versus pH. Fits to Equation 1 are solid lines. (a) 1.5 hours after preparation, (b) 7.5 hours and (c) 8.5 hours after preparation.

S5. Estimation of uncertainty in fitted pK_a values

The maximum and minimum ionic strength, I_{max} and I_{min}, are calculated from the max/min values of I predicted by the spreadsheet based on the concentration of oxalic acid and the composition of the buffers (Section S2). An additional 0.02 M ionic strength is added for all samples to represent the pH indicators and analyte. The uncertainty in the pK_a value of each indicator arising from the variation in ionic strength, Δ_{pKa}, is computed from their p$K_{a,0}$ values using Equation S23:

$$\Delta_{pKa} = |\Delta z^2| \left[0.51 \frac{\sqrt{I_{max}}}{1+\sqrt{I_{max}}} - 0.51 \frac{\sqrt{I_{min}}}{1+\sqrt{I_{min}}} + 0.1(I_{min} - I_{max})\right] \quad S23$$

A propagation of uncertainty analysis of Equation S1 yields the overall uncertainty in the pH reported by an indicator:[6]

$$\Delta_{pH} = \sqrt{{\Delta_{pK_a}}^2 + \left(\frac{\Delta_{\delta_H}}{2.303(\delta_{obs} - \delta_H)}\right)^2 + \left(\frac{\Delta_{\delta_L}}{2.303(\delta_L - \delta_{obs})}\right)^2 + \left(\frac{\Delta_{\delta_{obs}}(\delta_L - \delta_H)}{2.303(\delta_{obs} - \delta_H)(\delta_L - \delta_{obs})}\right)^2} \quad S24$$

where $\Delta_{\delta H}$, $\Delta_{\delta L}$ and $\Delta_{\delta obs}$ are the uncertainty in the limiting (δ_H and δ_L) and measured chemical shift of the indicator, all taken as 0.005 ppm in this work. Δ_{pH} is computed for each indicator in the pair used to calculate the pH and a weighted average taken according to S. The overall Δ_{pH} is computed using the same procedure used to calculate pH (Section S3). To estimate uncertainties in the fitted pK_a values of the analyte, the overall Δ_{pH} at each row of the CSI datset is multiplied by a random number between -1 and 1 and added to the experimental pH to generate three sets of pseudo experimental pH values. The experimental chemical shifts of the analytes are then fitted using the pseudo-experimental pH values to generate three sets of p$K_{n,0}$ values. The uncertainty of the fitted pK_a values (Tables 2 and 3) is given as the difference between the maxmium and minimum fitted values.

S6. Reproducibility of pH gradients and pK_a measurements used to determine pK_a of amino acids

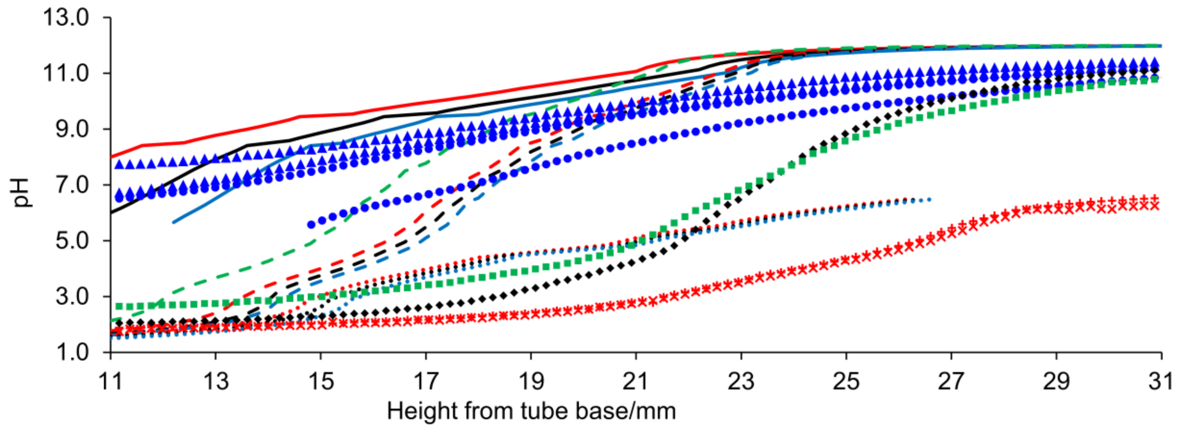

Figure S9. Plot of predicted pH versus height from base of NMR tube with different masses of oxalic acid (lines) to highlight effect of variability in mass weighed out: Lysine/tyrosine (solid line; 0.01 M NaOH, 20 mM Na_2CO_3, tris and $NaOB(OH)_2$; 1.0 mg (red), 1.3 mg (black) and 1.6 mg (blue) oxalic acid dihydrate, 8.5 hours since preparation). Histidine (dashed line, 0.01 M NaOH, Na_2CO_3, $NaOB(OH)_2$, tris, K_2HPO_4 and acetate-d_3; 3.4 mg (red), 3.7 mg (black), 4.0 mg (blue) oxalic acid dihydrate, 6.5 hours: 2.6 mg oxalic acid dihydrate (green), 6 hours). Aspartic acid (dotted line, 0.01 M $NaHCO_3$, 0.01 M acetate-d_3 and 0.02 M oxalate; 3.1 mg (red), 3.4 mg (black), 3.7 mg (blue) oxalic acid dihydrate, 6.6 hours). Experimental pH gradients including repeats: Lysine (blue triangle), tyrosine (blue circle), aspartic acid (red cross), histidine (3.7 mg oxalic acid, black diamond, 6.5 hours; 2.6 mg oxalic acid, green square, 6 hours).

The uncertainty in p$K_{1,0}$ for histidine is observed to be much higgher with a pH range of 2.6 to 10.9 when only 2.6 mg oxalic is used (Figure S10,a) while exclusion of the β-resonance from the fitting does not change the fitted pK_a within experimental uncertainty (Figure S6), suggesting that two resonances are adequate to extract pK_a values of these amino acids. For aspartic acid, inclusion of a datapoint at pH 0.8 measured in 0.2 M HCl (pH 0.9) does not significantly improve the accuracy of the fitted p$K_{1,0}$ value (Figure S11) demonstrating that the pH range afforded in a single CSI experiment is adequate even for this highly acidic compound.

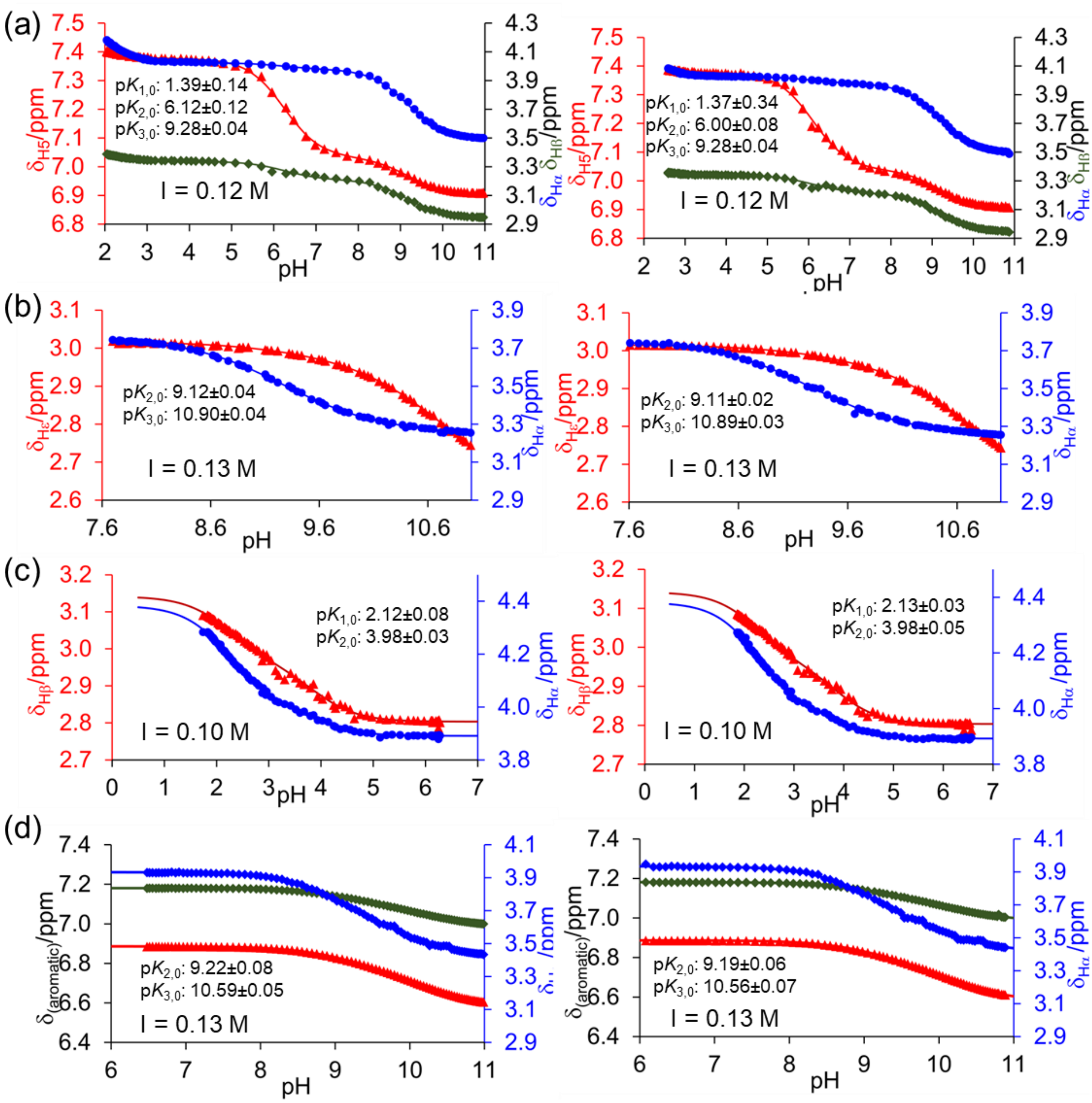

Figure S10. Plots of δ_{obs} versus pH to determine pK_a values of amino acids (two repeats). The solid lines are fits to Equation 1. Samples are same as plotted on Figure S9. (a) Histidine α (blue circle), downfield β resonance (green diamond) and H_5 (imidazole) proton (red triangle). Left: 3.7 mg oxalic acid, Right: 2.6 mg oxalic acid. (b) Lysine α (blue circle), ε (red triangle). (c) Aspartic acid α (blue circle) and downfield β (red triangle). (d) Tyrosine α (blue circle), upfield aromatic (red triangle) and downfield aromatic (green diamond).

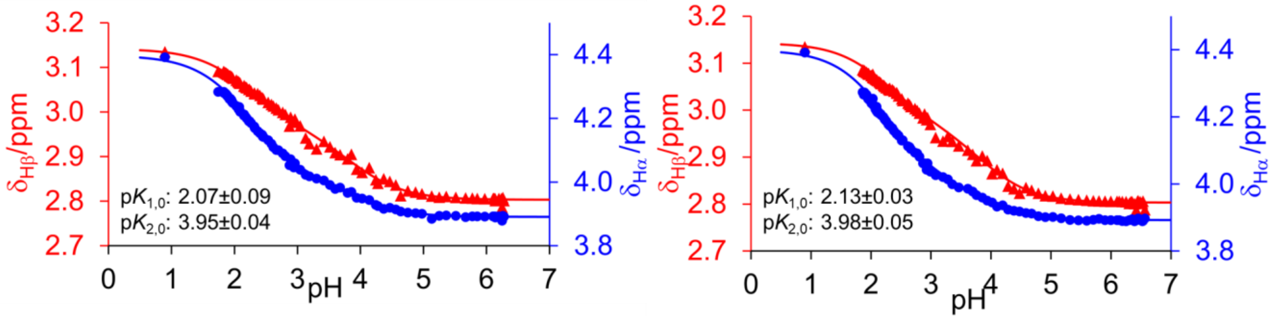

Figure S11. Plot of aspartic acid α (blue circle) and downfield β (red triangle) when point measured in 0.2 M HCl (pH 0.9) is included in fit. Datasets are same as Figure S10,c.

S7. CSI datasets used to determine pK_a of amino acids (Table 2)

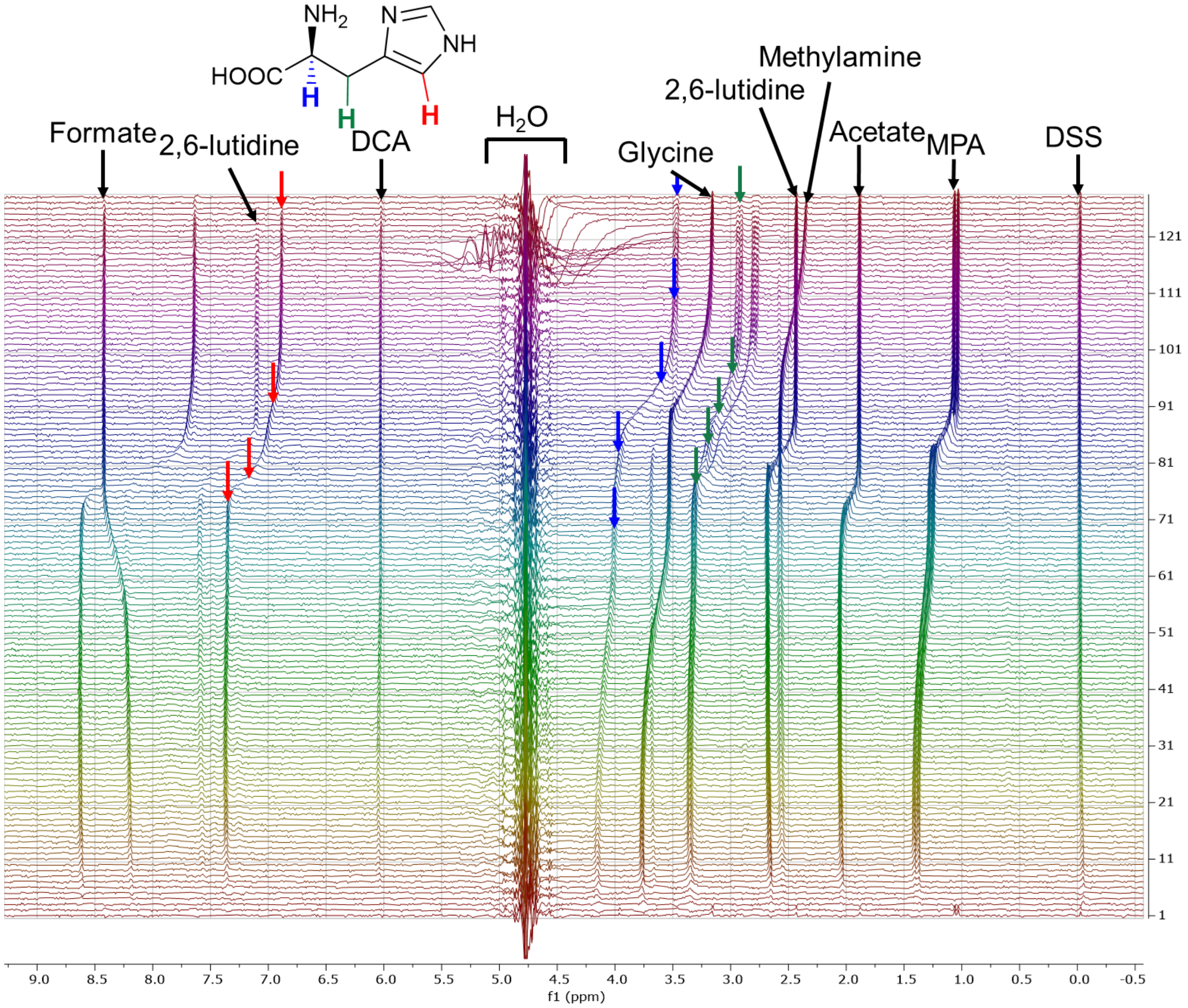

Figure S12. ^1H spectra of CSI dataset used to extract pK_a values of histidine. Resonances of indicators (black) and observed resonances of histidine (Figure 1) indicated with arrows.

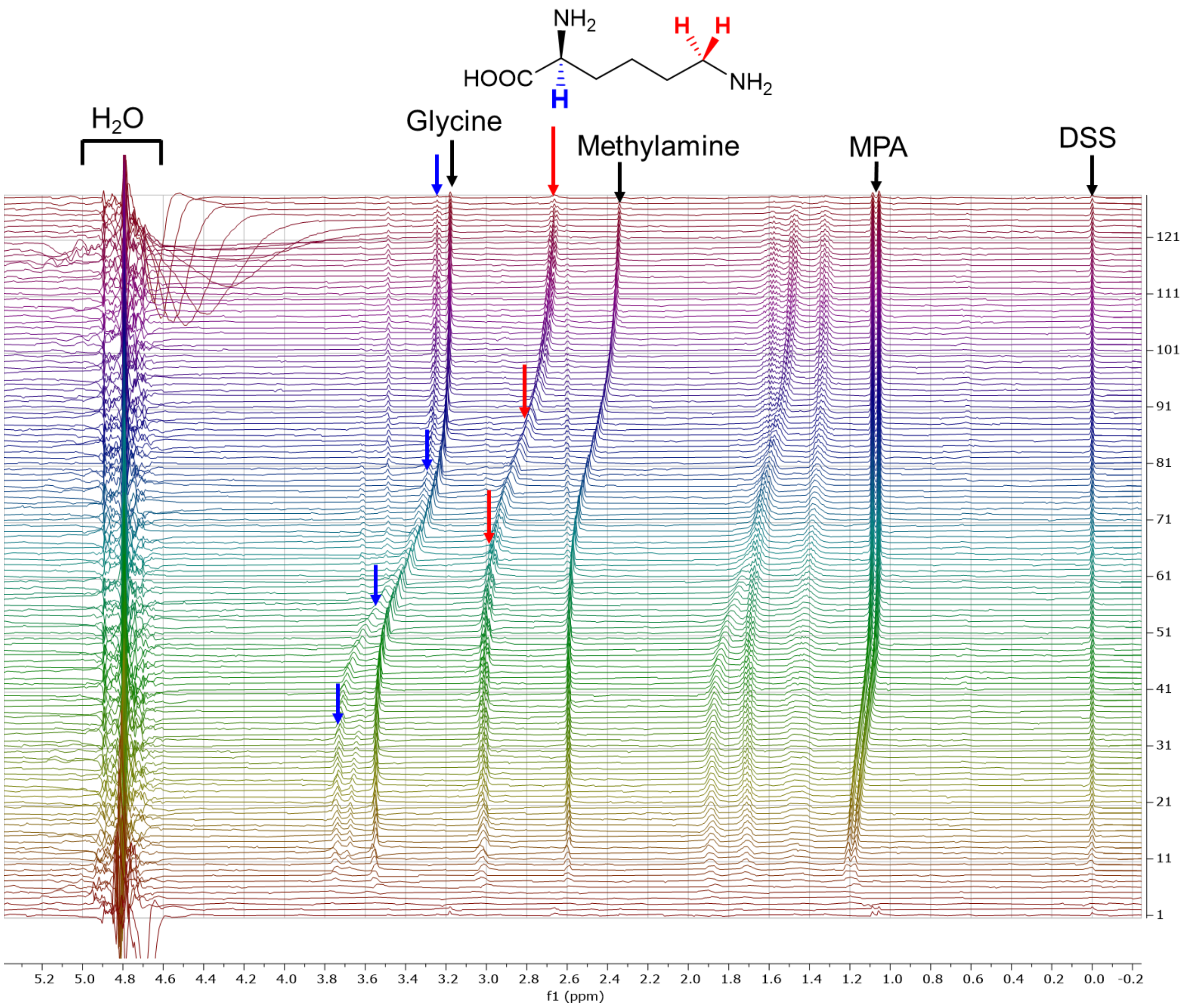

Figure S13. ^{1}H spectra of CSI dataset used to extract pK_a values of lysine. Resonances of indicators (black) and observed resonances of lysine indicated with arrows.

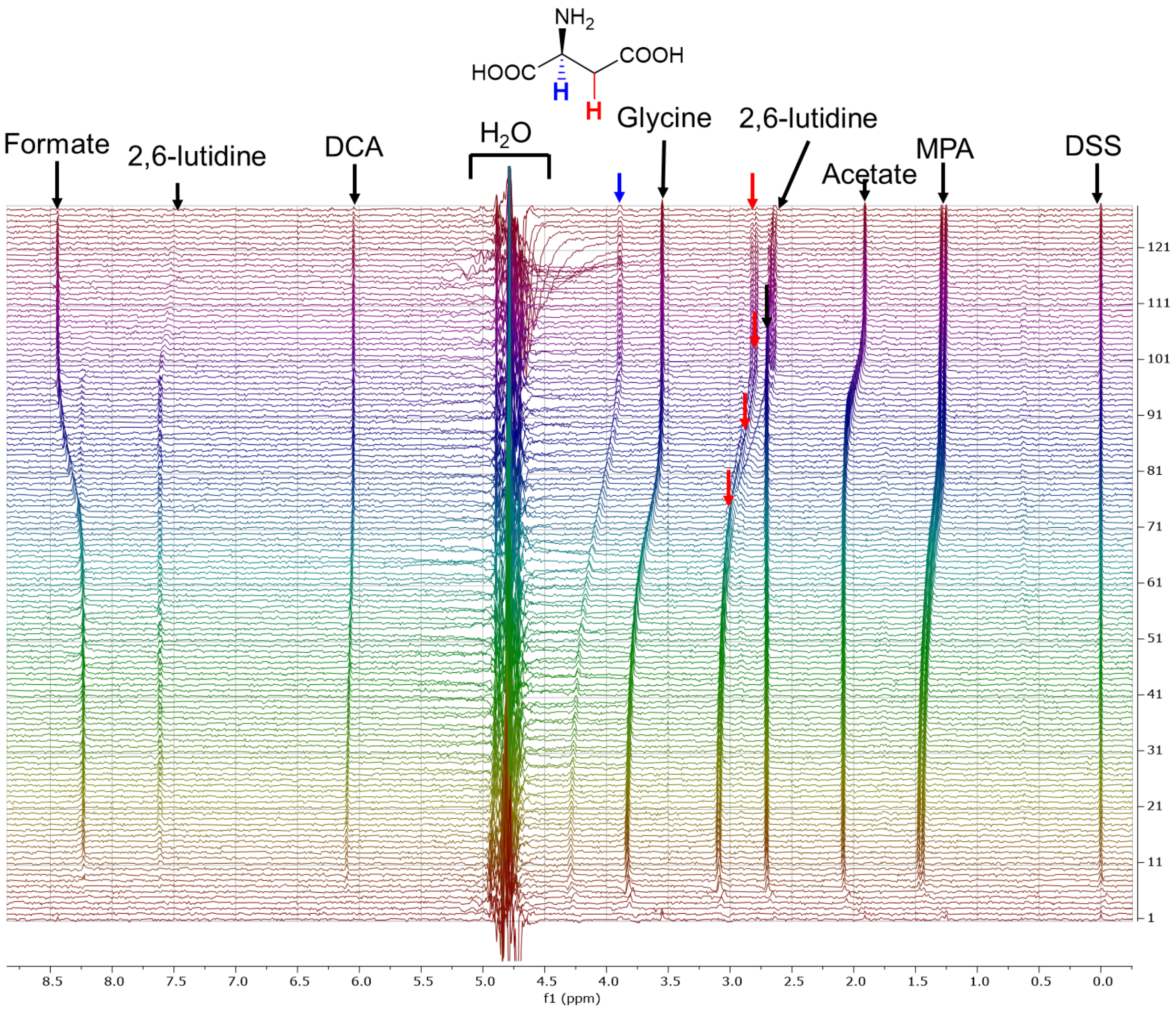

Figure S14. ^{1}H spectra of CSI dataset used to extract pK_a values of aspartic acid. Resonances of indicators (black) and observed resonances of aspartic acid indicated with arrows.

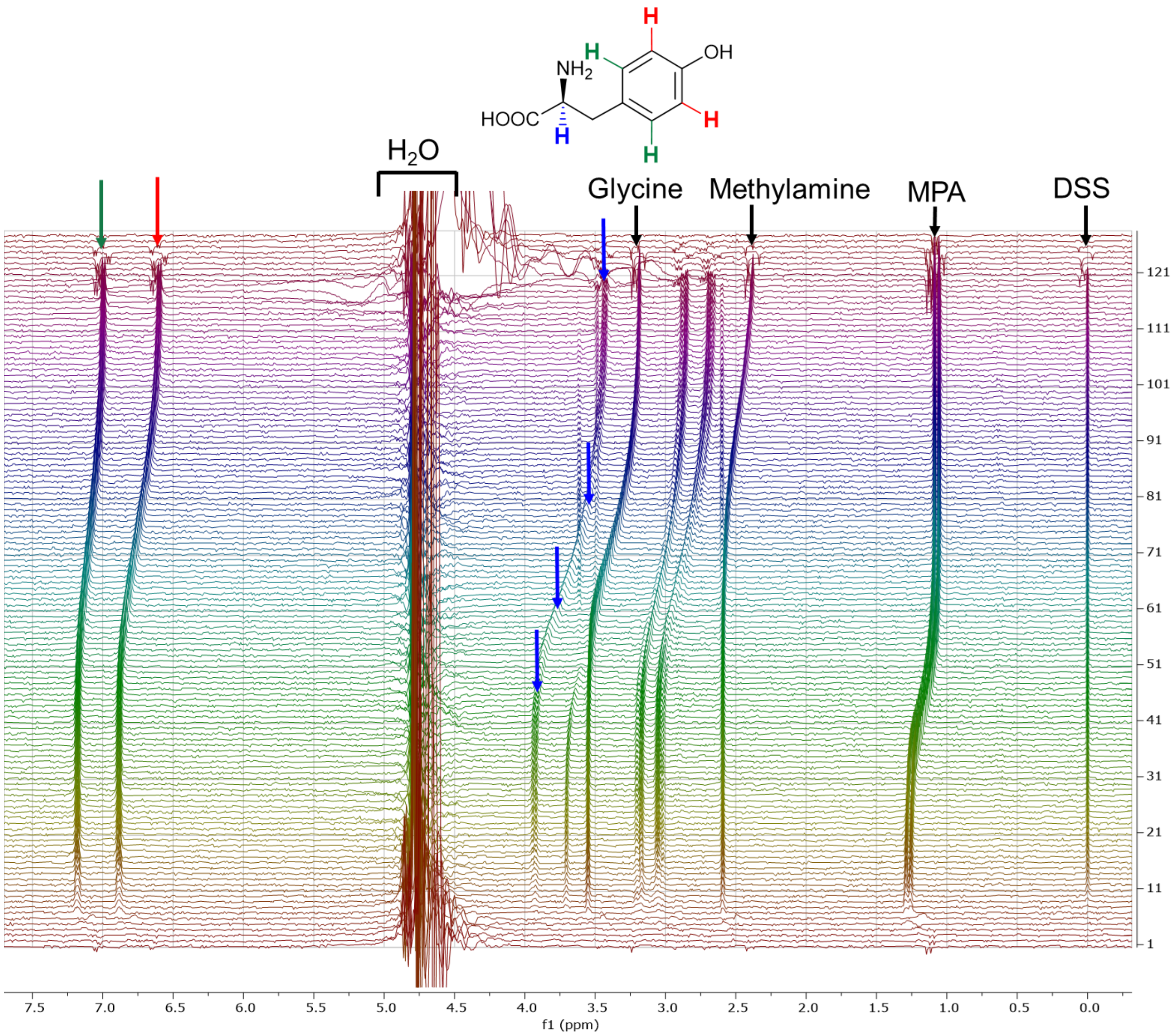

Figure S15. ^{1}H spectra of CSI dataset used to extract pK_a values of tyrosine. Resonances of indicators (black) and observed resonances of tyrosine indicated with arrows.

S8. Example predicted and experimental pH gradients used to determine pK_a of compounds A – M

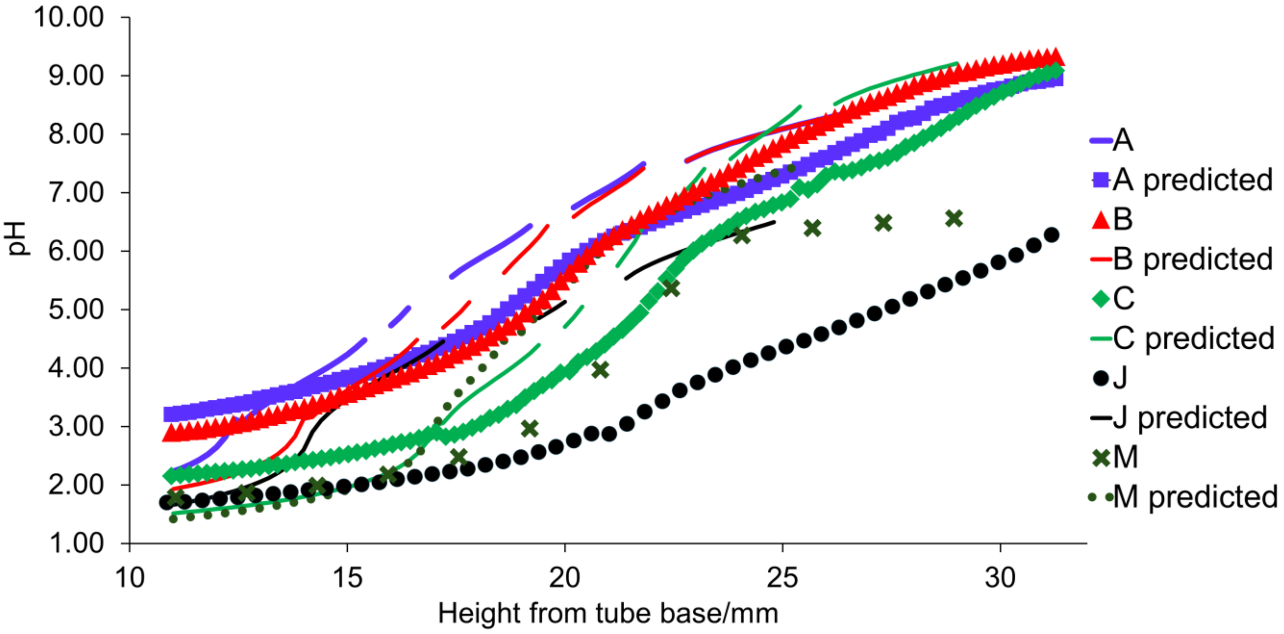

Figure S16. Example plots of predicted (line) versus experimentally observed (symbol) pH gradients used to determine pK_a values of **A – M**.

S9. CSI datasets used to determine pK_a of compounds A – M and diethyl benzyliminodiacetate

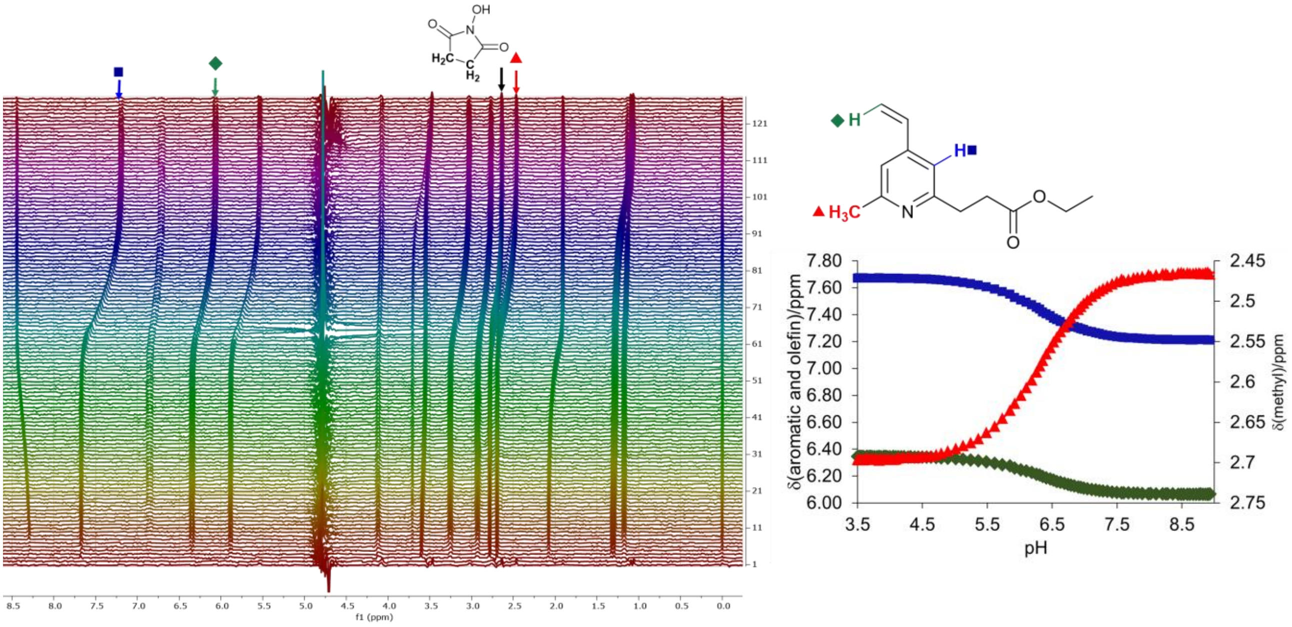

Figure S17. Stacked ^{1}H spectra from CSI dataset used to determine pK_a of **A**. Fitted resonances of **A** are indicated, along with resonance of NHS.

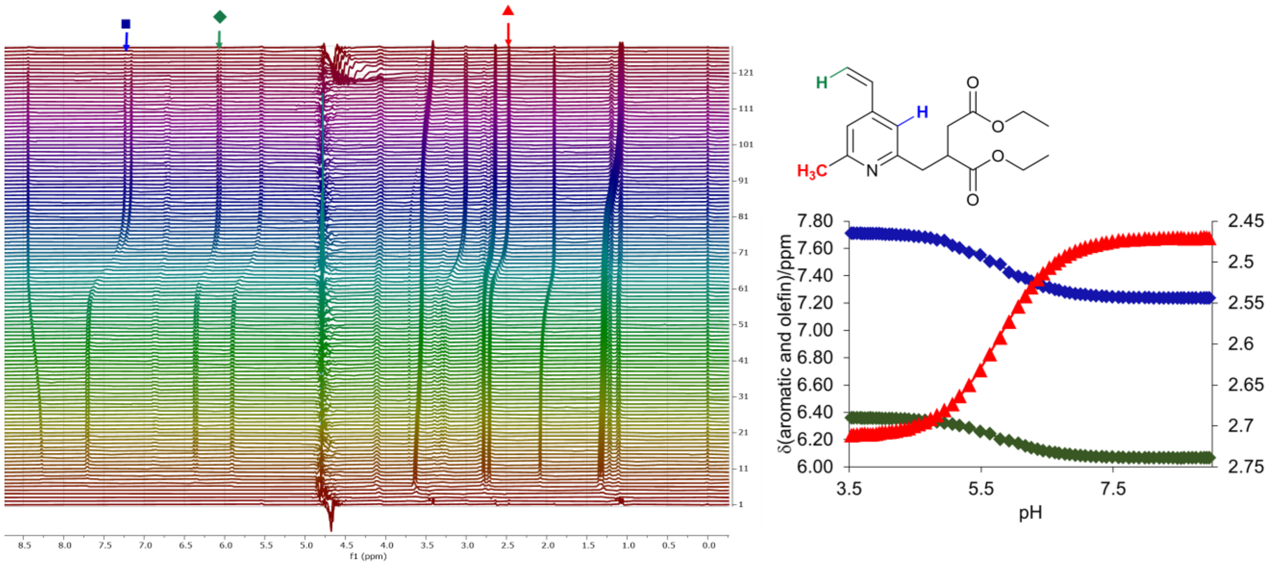

Figure S18. Stacked ^1H spectra from CSI dataset used to determine pK_a of **B**. Fitted resonances of **B** are indicated.

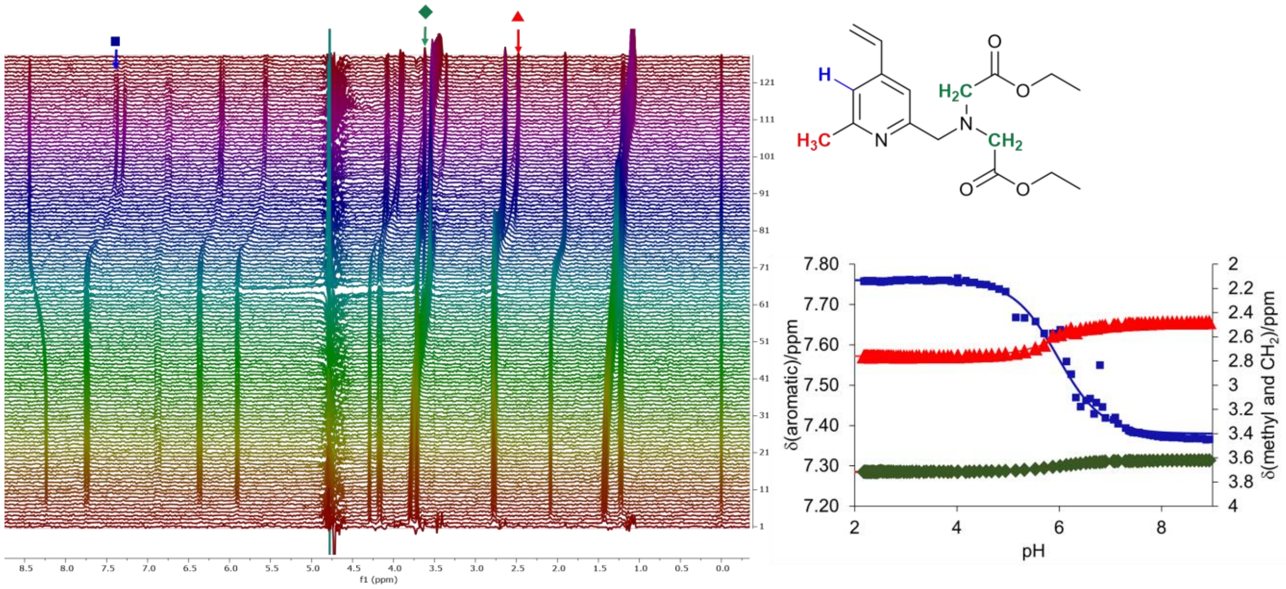

Figure S19. Stacked ^1H spectra from CSI dataset used to determine pK_a of **C**. Fitted resonances of **C** are indicated.

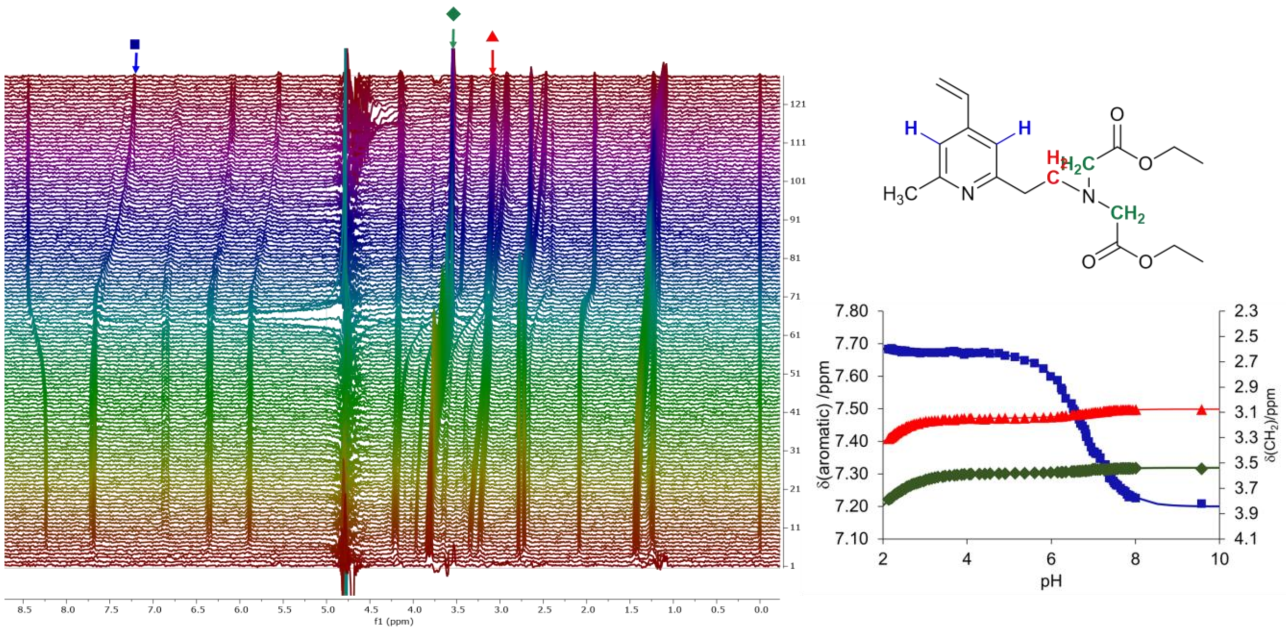

Figure S20. Stacked **¹**H spectra from CSI dataset used to determine pK_a of **D**. Fitted resonances of **D** are indicated. ¹H chemical shifts measured in 1D spectrum immediately after preparation (pH > 9) were included in the fitting.

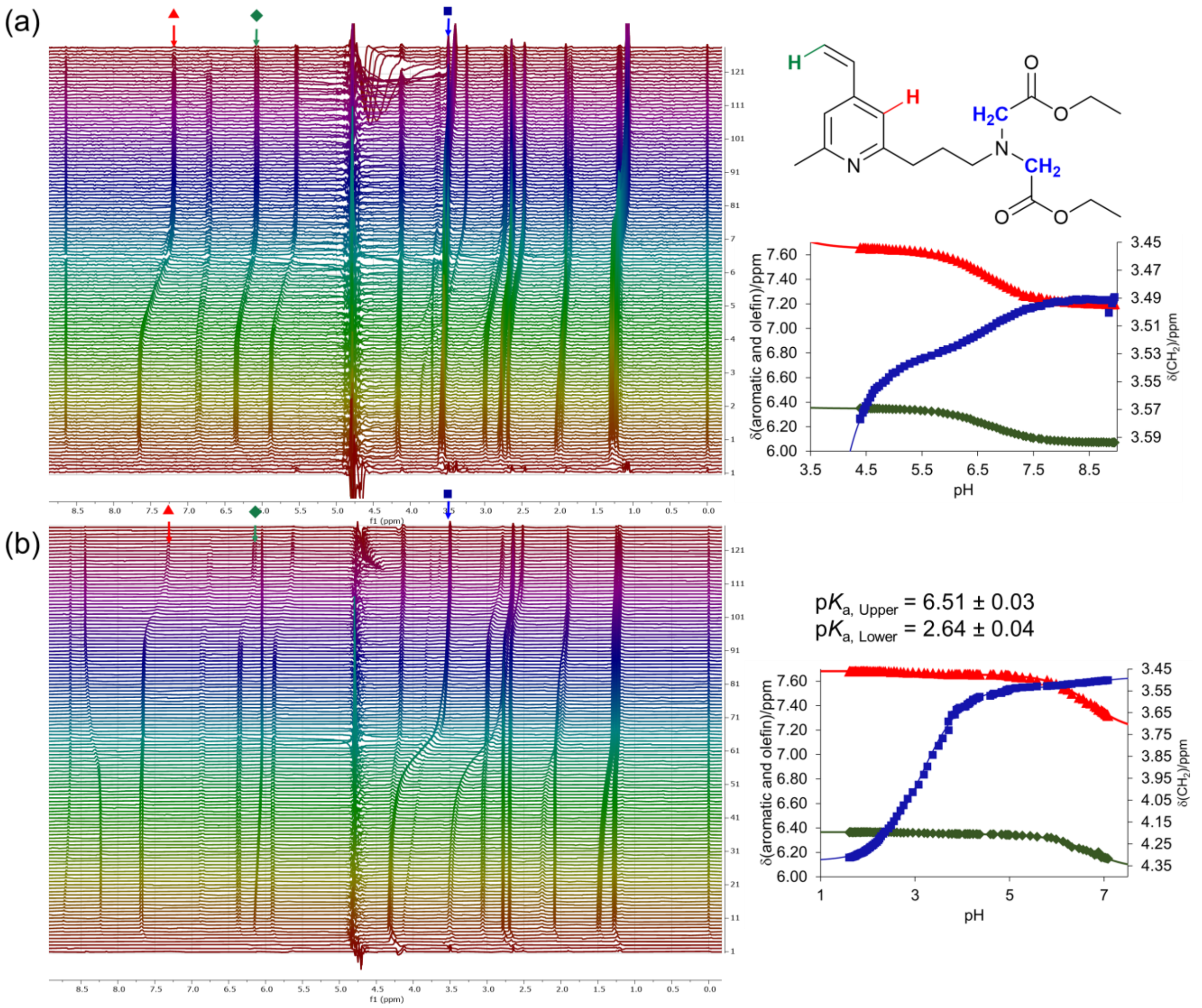

Figure S21. Stacked ^{1}H spectra from CSI dataset used to determine pK_a of **E**. Fitted resonances of **E** are indicated. (a) pH gradient to determine pK_a of pyridinium and tertiary amine (Table 3). (b) Acidic pH gradient to confirm low basicity of tertiary amine.

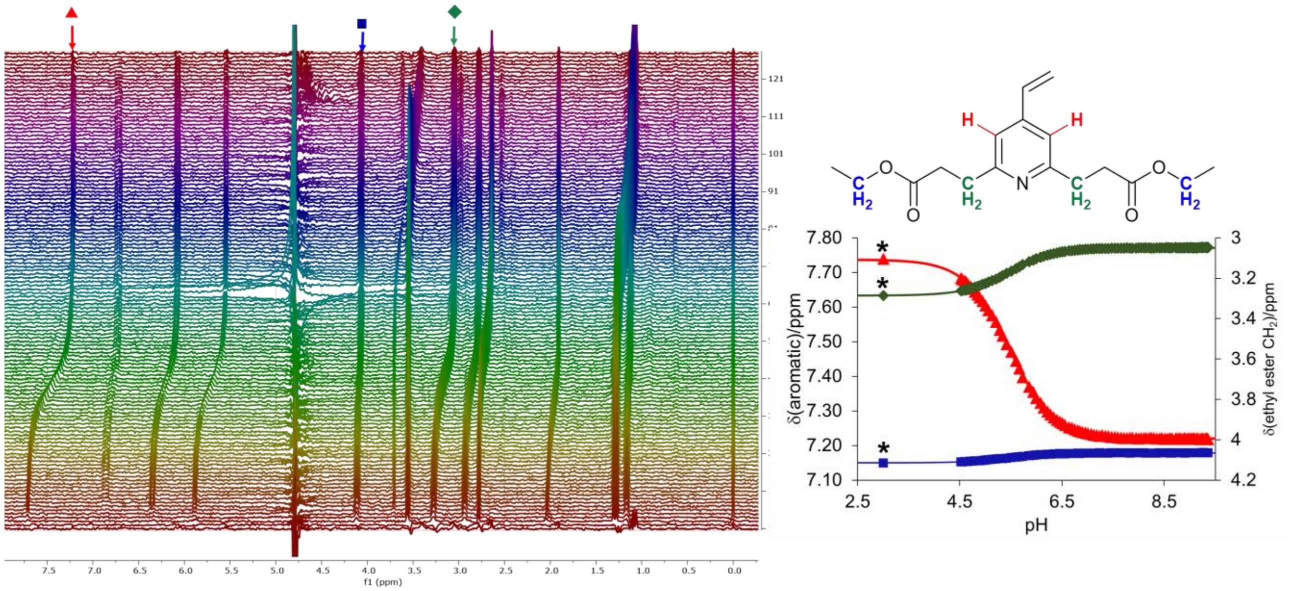

Figure S22. Stacked ^{1}H spectra from CSI dataset used to determine pK_a of **F**. Fitted resonances of **F** are indicated. The additional point marked * was measured in a homogeneous solution containing 1,5 mM **F**, 10 mM HCl, 4 mM formate, 2 mM acetate, 4 mM DCA, 2 mM MPA, 0.2 mM DSS. However, this point was not included in the fitting to Equation 1, confirming the validity of extrapolation using such a large number of points.

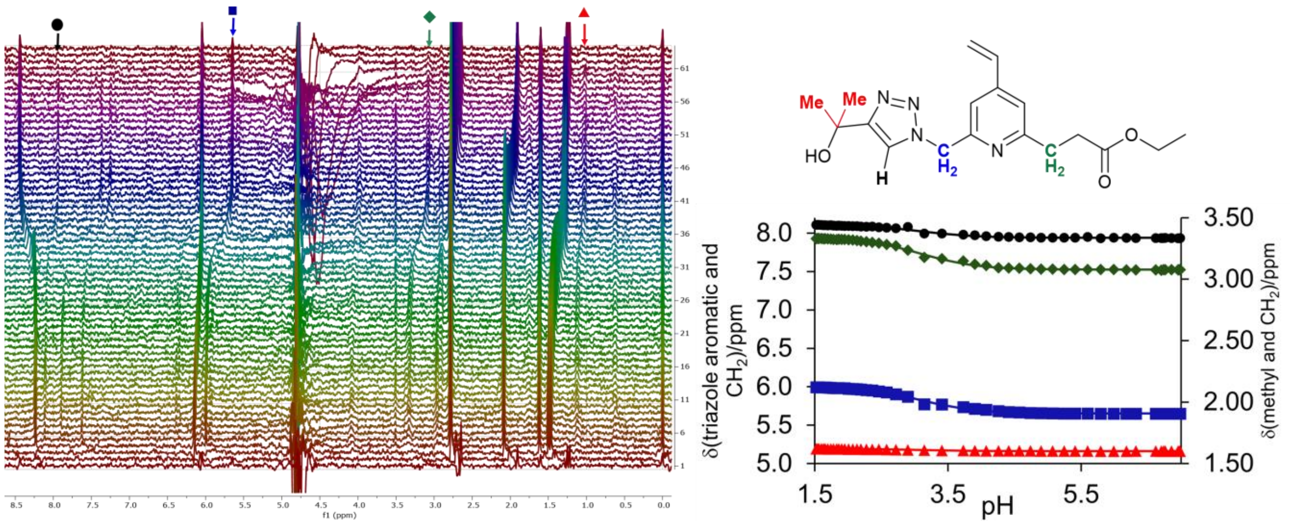

Figure S23. Stacked ^{1}H spectra from CSI dataset used to determine pK_a of **G**. Fitted resonances are indicated.

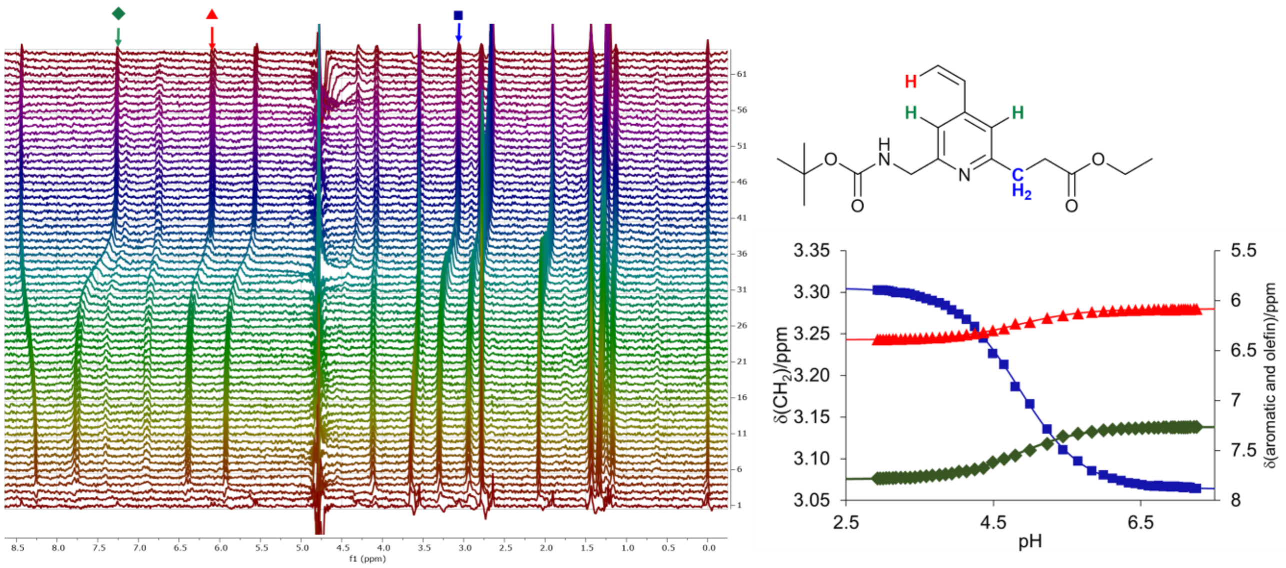

Figure S24. Stacked ^{1}H spectra from CSI dataset used to determine pK_a of **H**. Fitted resonances are indicated.

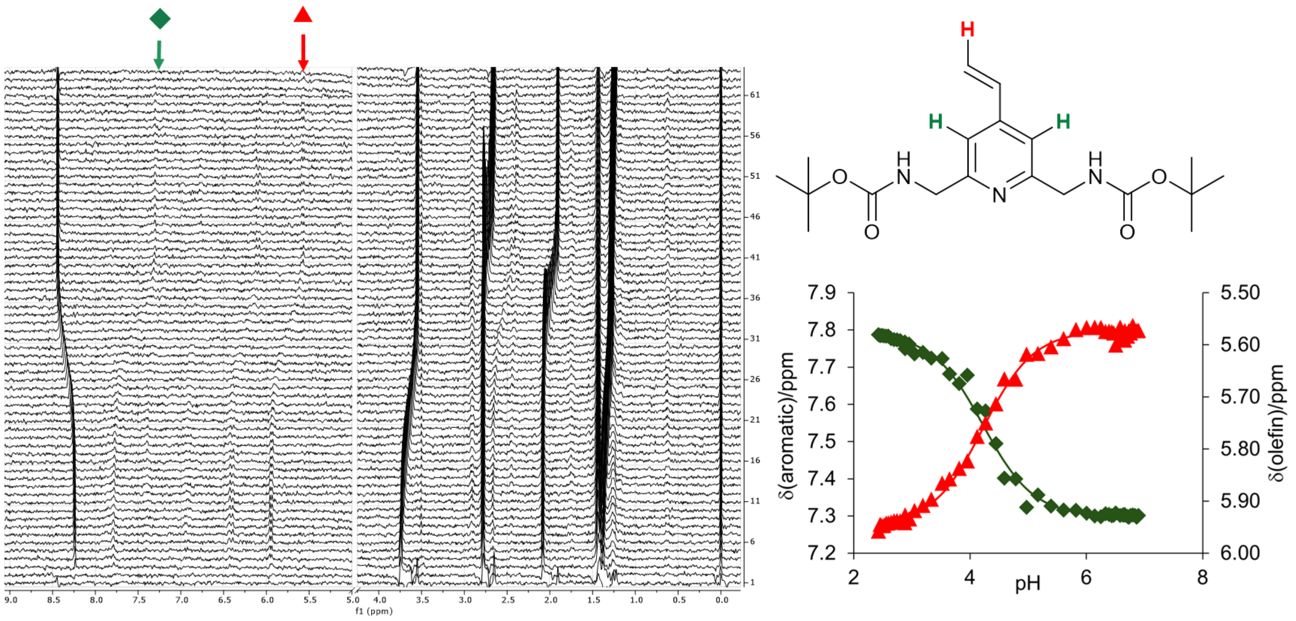

Figure S25. Stacked ^{1}H spectra from CSI dataset used to determine pK_a of **I**. Fitted resonances are indicated.

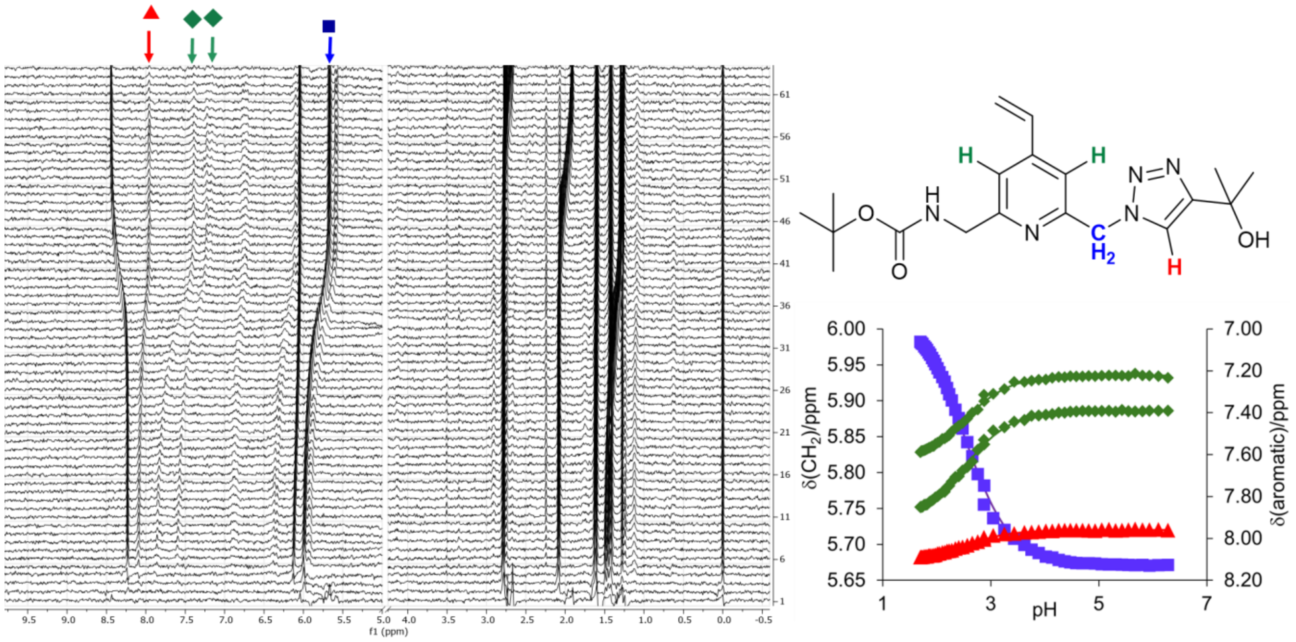

Figure S26. Stacked ^1H spectra from CSI dataset used to determine pK_a of **J**. Fitted resonances are indicated.

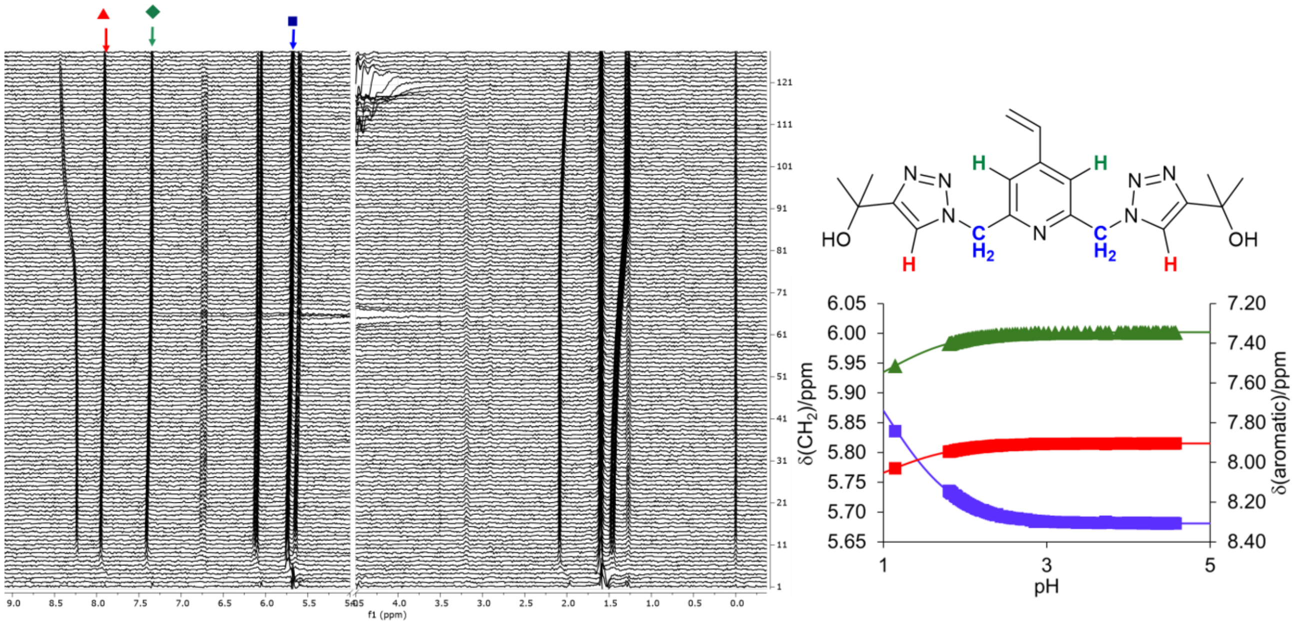

Figure S27. Stacked ^1H spectra from CSI dataset used to determine pK_a of **K**. Fitted resonances are indicated. The isolated data point at very acidic pH was measured in a separate homogeneous sample containing all components of the CSI sample, but with 0.1 M HCl in place of the oxalic acid.

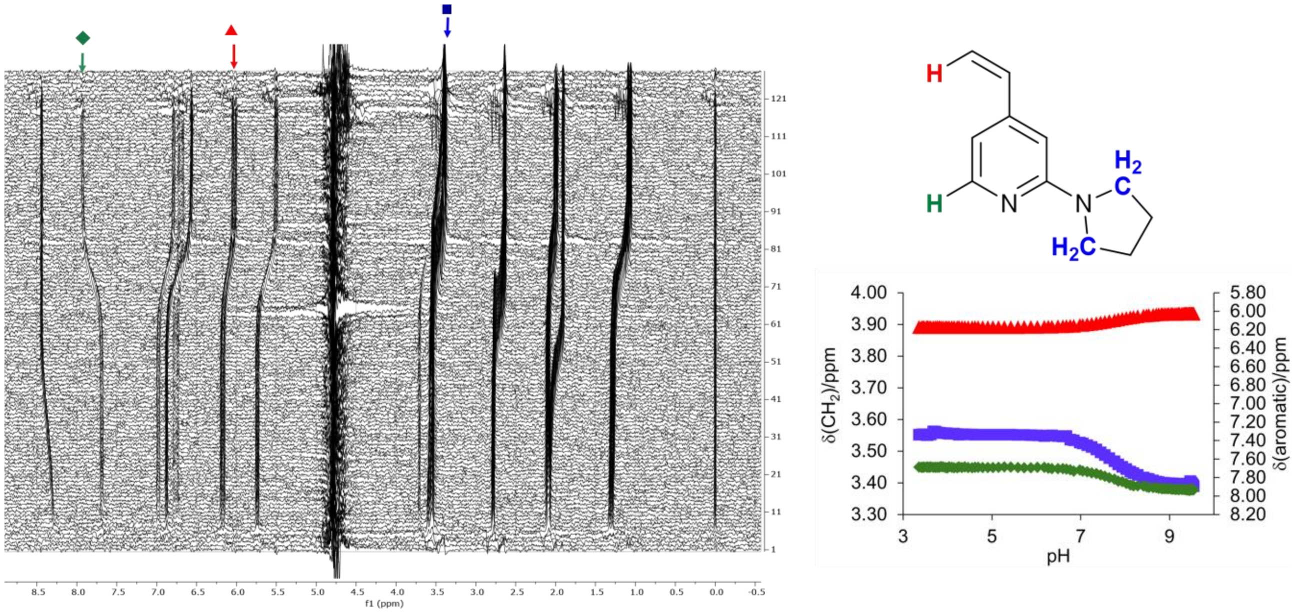

Figure S28. Stacked ^{1}H spectra from CSI dataset used to determine pK_a of **L**. Fitted resonances are indicated.

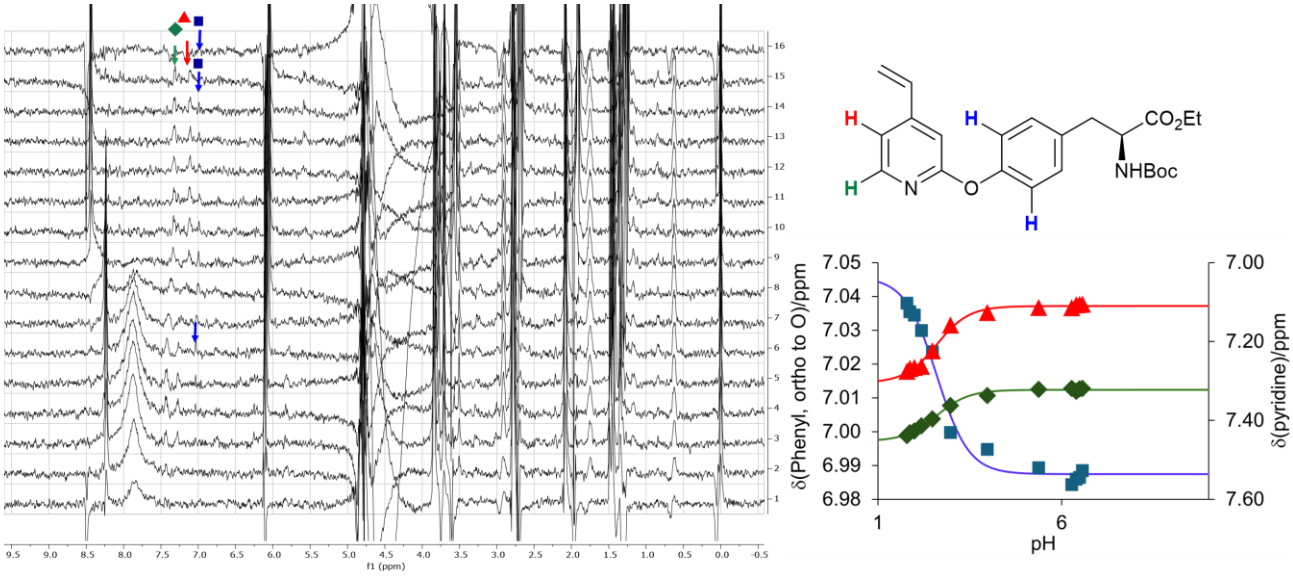

Figure S29. Stacked ^{1}H spectra from CSI dataset used to determine pK_a of **M**. Fitted resonances are indicated.

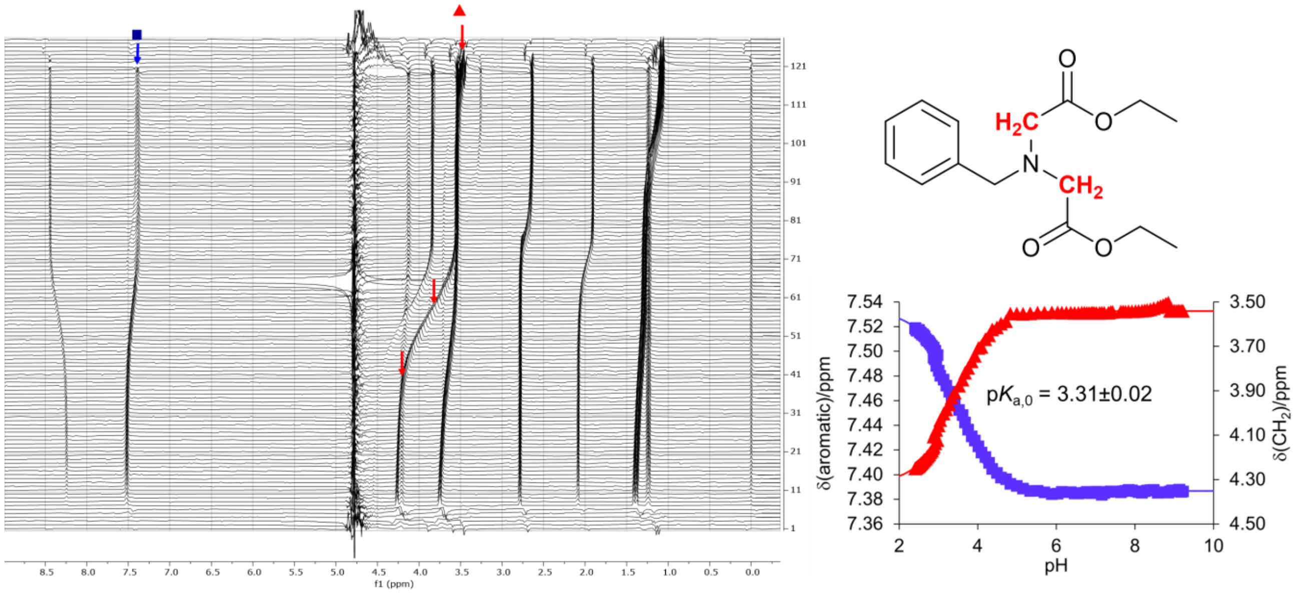

Figure S30. Stacked ^{1}H spectra from CSI dataset used to determine pK_a of diethyl benzyliminodiacetate. Fitted resonances are indicated.

S10. Prediction of pK_a using Jaguar pK_a

The pK_a values in Table 3 were predicted using Schrödinger Release 2024-2 (Jaguar pKa, Schrödinger, LLC, New York, NY, 2024).[56-58] Jaguar pKa performed a conformational search for the five least energetic geometries in the gas phase. This was done with an energy window of 12 kcal/mol for protonated and deprotonated species, using a force field-based MacroModel.[59-60] These were then used as a starting point for a subsequent Density Function Theory (DFT) geometry optimisation using B3LYP/6-31G*. Accurate single-point energies were calculated using B3LYP/cc-pVTZ(+). Pseudospectral methods were turned off. The solvation-free energy of the protonated and deprotonated species was calculated using empirical parameterisation.

Entry molecules for the Jaguar should represent a relevant protonation state. Therefore, for compounds 3-4, a neutral molecule was calculated for both pyridine and tertiary amine moiety to establish the most basic region. In all cases, pyridine was proved to be more basic (Figures S31-33). A molecule with protonated pyridine was used to calculate the pK_a of tertiary amine sites on **C - E**.

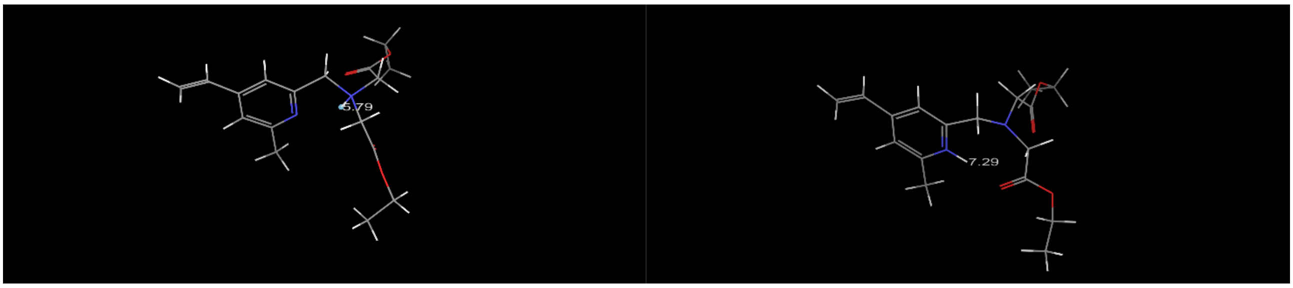

Figure S31. Predictions of pK_a for compound **C**: Protonated tertiary amine with pyridine neutral (left), and protonated pyridine with amine site neutral (right).

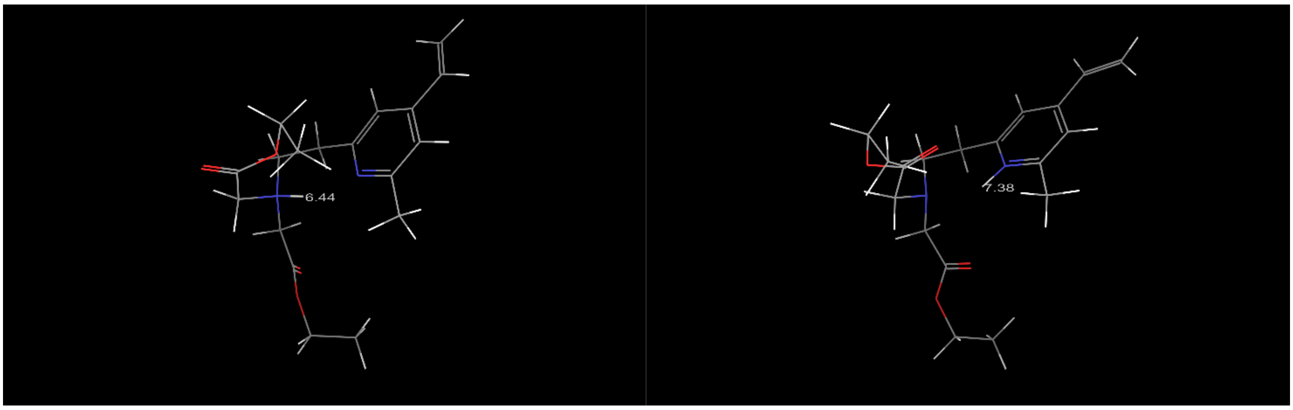

Figure S32. Predictions of pK_a for compound **D**: Protonated tertiary amine with pyridine neutral (left), and protonated pyridine with amine site neutral (right).

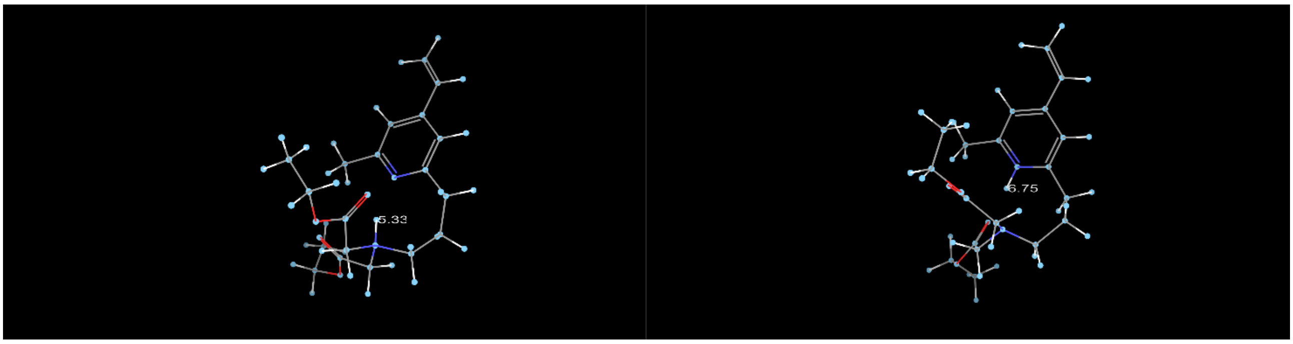

Figure S33. Predictions of pK_a for compound **E**: Protonated tertiary amine with pyridine neutral (left), and protonated pyridine with amine site neutral (right).

S11. Methods for preparation of 4-vinylpyridines A – M and ^{1}H and ^{13}C NMR spectra

Two synthetic approaches were employed for the generation of single-arm compounds 4-vinylpyridine derivatives, or dual-armed compounds with functionality linked not only to position 2, but also position 6. The first approach employed 4-bromo-2-(bromomethyl)-6-methylpyridine **S3** as a common starting material (Scheme S1). Substitution with enolate or amine nucleophiles gave intermediates **S6-S8** which all underwent clean palladium-catalysed cross-coupling with potassium vinyltrifluoroborate to generate 4-vinylpyridines **A-C**. This method of introducing the 4-vinyl group in the final step was found to be equally applicable to all the derivatives synthesised in this study. Ester functionalities serve for linker group introduction. To increase the length of the single carbon-spacer of **C** between pyridine and the iminodiacetate group, the two-carbon analogue **D** was synthesised from **S3** by use of cyanide as the nucleophile, and after conversion to the two-carbon electrophile **S12**, incorporation of diethyl iminodiacetate as before. The three-carbon analogue **E** was accessed by reductive amination with diethyl iminodiacetate on aldehyde **S15**.

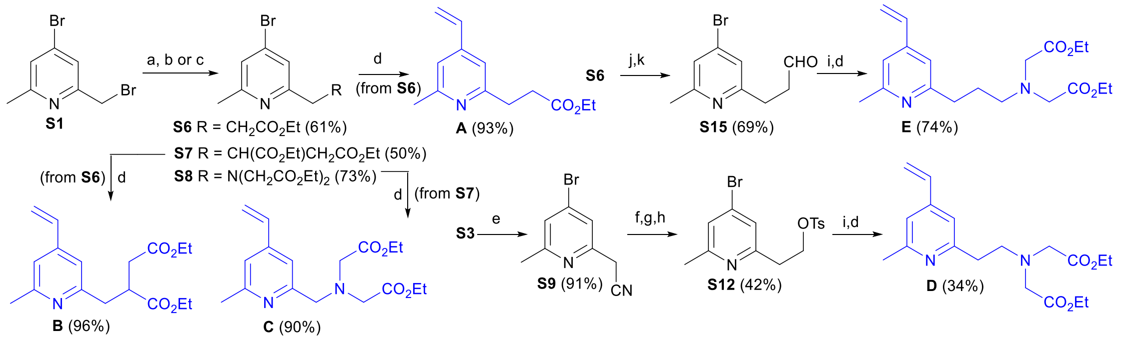

Scheme S1. Synthesis of single-arm 4-vinylpyridine derivatives **A**-**E**. Methods (see ESI for full details): a) LDA (1 eq.), EtOAc (1 eq.), THF, -78 °C to RT. b) LDA (1 eq.) diethyl succinate (1 eq.), -78 °C to RT. c) Diethyl iminodiacetate (2 eq.), K_2CO_3 (3 eq.), DMF, 75 °C. d) Potassium vinyltrifluoroborate (1 eq.), NEt_3 (3 eq.), (dppf)$PdCl_2$ (5 mol%), 2 : 1 PhMe/*i*-PrOH, 90 °C. e) TMSCN (1.5 eq.), TBAF (1.5 eq.), MeCN, Δ. f) MeOH, H_2SO_4, Δ (99%). g) $LiAlH_4$ (2 eq.), THF, 0 °C, then H_2O (57%). h) TsCl (1.2 eq.), KOH (1.5 eq.), THF, 0 °C to RT (74%). i) Diethyl iminodiacetate (3 eq.), CH_2Cl_2, Δ (44%). j) $LiAlH_4$ (2 eq.), THF, 0 °C, then H_2O (88%). k) DMSO (2.6 eq.), $(COCl)_2$ (1.3 eq.), NEt_3 (4 eq.), CH_2Cl_2, -78 °C to 0 °C (78%). l) Diethyl iminodiacetate, $NaBH(OAc)_3$ (2 eq.), AcOH (2 eq.), DCE, RT (76%).

Synthesis of the dual-armed compounds started with 4-bromo-2,6-bis(bromomethyl)pyridine **S17** (Scheme S2). Double substitution with the enolate derived from ethyl acetate by deprotonation with LDA, followed as before by palladium-catalysed vinylation, gave **F** the duel-armed ethyl ester equivalent of **A**. Alternatively, mono-substitution to give **S19** and a second substitution with azide, was

followed using **S20** in either a copper-catalysed alkyne 'click' addition, or azide reduction and Boc protection, leading to differentially substituted derivatives **G** and **H** respectively. Three further dual-armed derivatives containing either two (Boc protected) amino groups (**I**), an amino/functionalised triazole (**J**), or a bis-functionalised triazole (**K**) were obtained similarly from the common bis-azide intermediate **11**. Finally, two 2-heteroatom substituted derivatives **L** and **M** were synthesised by reaction of pyrrolidine or a protected (*S*)-tyrosine with 4-iodo-pyridine-*N*-oxide and phosphonium salt PyBroP,[75] followed as before by palladium-catalysed vinylation.

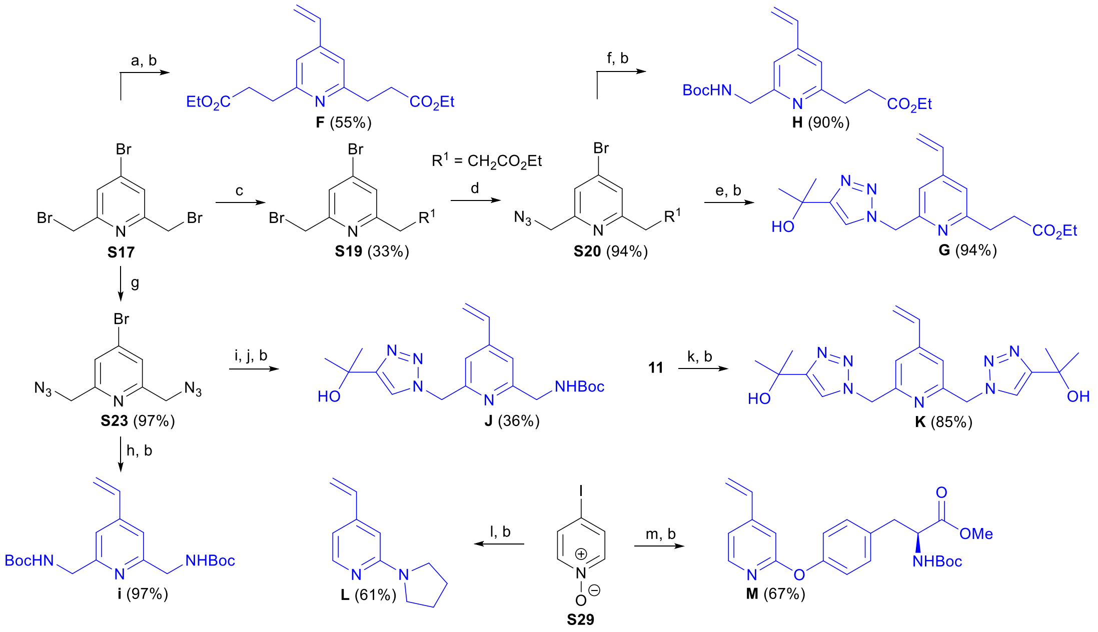

Scheme S2. Synthesis of dual-armed 4-vinylpyridine derivatives **F**-**K** and 2-heteroatom substituted derivatives **L** and **M**. Methods (see ESI for full details): a) LDA (2 eq.), EtOAc (2 eq.), THF, -78 °C to RT (56%). b) Potassium vinyltrifluoroborate (1 eq.), NEt_3 (3 eq.), (dppf)$PdCl_2$ (5 mol%), 2 : 1 PhMe/*i*-PrOH, 90 °C. (c) a) LDA (2 eq.), EtOAc (2 eq.), THF, -78 °C to RT (check this?). (d) NaN_3 (1.5 eq.), DMSO, RT. (e) Methyl-3-butyn-2-ol (1.5 eq.), CuI (10 mol%), THF (96%). (f) PPh_3 (1.5 eq.), THF, RT then H_2O, $NaHCO_3$ (11 eq.), Boc_2O (1.5 eq.), RT (94%). (g) NaN_3 (3 eq.), DMSO, RT. (h) PPh_3 (2.2 eq.), THF, RT then H_2O, $NaHCO_3$ (2.3 eq.), Boc_2O (2.5 eq.), RT (98%). (i) PPh_3 (1 eq.), THF/H_2O, RT then $NaHCO_3$ (8 eq.), Boc_2O (1.5 eq.), RT (59%). (j) Methyl-3-butyn-2-ol (1.1 eq.), CuI (10 mol%), THF (62%). (k) Methyl-3-butyn-2-ol (3 eq.), CuI (19 mol%), THF (92%). (l) Pyrrolidine (1.3 eq.), PyBroP (1.3 eq.), NEt_3/CH_2Cl_2 (73%). (m) (*S*)-Methyl (*t*-butoxycarbonyl) tyrosinate (1.3 eq.), PyBroP (1.3 eq.), NEt_3/CH_2Cl_2 (92%).

Synthesis of 4-bromo-6-methyl-2-pyridinemethanol S2[61]

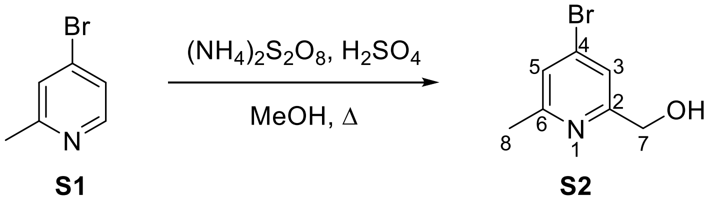

To a 250 mL round-bottom flask was added 4-bromo-2-methylpyridine **S1** (2.5 g, 14.5 mmol), methanol (63 mL), and sulphuric acid (0.13 mL). The mixture was heated to reflux, followed by the addition of ammonium peroxydisulphite (3.32 g) in water (6 mL). After 2 h, the mixture was allowed to cool, and the volatiles were removed *in vacuo*. The remainder was neutralised with slow addition of $NaHCO_3$ sat. soln. (approx. 50 mL), and extracted with ethyl acetate (3 × 100 mL). The organics were washed with brine (50 mL), dried over Na_2SO_4, filtered, and the volatiles removed *in vacuo*. The mixture was purified by flash column chromatography using 30% ethyl acetate in hexanes to yield **S2** as a colourless solid (1.05 g, 36%); Mp 98 – 101 °C; R_f 0.35 (30% ethyl acetate in hexanes); HRMS (ES) $[M+H]^+$ $C_7H_8Br^{79}NO+H^+$, calc. 201.9868, obs. 201.9860; v_{max}/cm^{-1} 3204 (OH), 1567 (C-C stretch), 1422 (C-H), 818 (C-H); ^{1}H-NMR (500 MHz, $CDCl_3$) δ 7.29 (1H, s, 5-CH), 7.28 (1H, s, 3-CH), 4.71 (2H, s, 7-CH_2), 3.57 (1H, s, OH), 2.55 (3H, s, 8-CH_3); ^{13}C-NMR (126 MHz, $CDCl_3$) δ 160.9 (8-C), 158.8 (7-C), 133.9 (4-C), 125.0 (3-C), 121.1 (5-C), 63.9 (7-C), 23.8 (8-C).

Synthesis of 4-bromo-2-(bromomethyl)-6-methylpyridine S3[62]

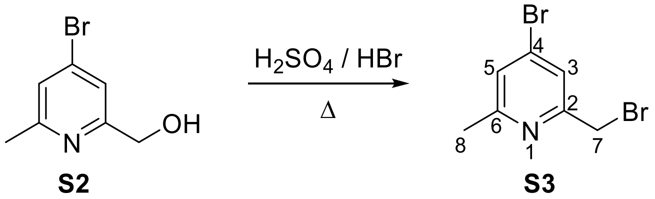

A 50 mL round-bottom flask was charged with 4-bromo-6-methyl-2-pyridinemethanol **S2**(1.05 g, 5.20 mmol). The Flask was cooled in an ice bath and H_2SO_4/HBr (20mL, v/v = 4:6) was added slowly. The flask was then fitted with a reflux condenser and heated to reflux. After 4 h, the reaction vessel was allowed to cool to room temperature and the mixture poured into ice and neutralised with addition of solid sodium bicarbonate. The crude product was extracted with dichloromethane (3 × 30 mL), dried over $MgSO_4$, and filtered. The solvent was removed *in vacuo,* to give a brown solid, and purified by flash column chromatography

using dichloromethane to yield **1** as a colourless solid (1.13 g, 83%). R_f 0.42 (dichloromethane). Characterisation data matches that of **S3** given below.

Synthesis of 4-bromo-2-(bromomethyl)-6-methylpyridine S3, 4-bromo-2-(dibromomethyl)-6-methylpyridine S5, 4-bromo-2,6-bis(bromomethyl)pyridine S17

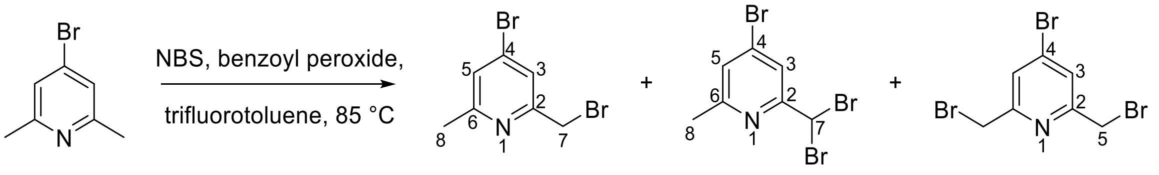

A 250 mL 2-neck round-bottom flask, fitted with a reflux condenser, was charged with 4-bromo-2,6-dimethylpyridine **S4** (2.00 g, 10.7 mmol), *N*-bromosuccinimide (2.10 g, 11.8 mmol), and benzoyl peroxide (34 mg, 0.10 mmol). The flask was purged with argon. To the solids was added α,α,α-trifluorotoluene (100 mL) and the reaction vessel heated to 85 °C. Upon reaching temperature the reaction turned to a yellow solution. After 7.5 h a further 0.01 eq. of benzoyl peroxide (34 mg, 0.10 mmol) was added. After a total of 23 h the heat was removed. After allowing the reaction to cool to room temperature the reaction was diluted with dichloromethane (100 mL), washed with water (2 × 50 mL) and brine (50 mL), dried over $MgSO_4$ and filtered. The solvent was removed *in vacuo,* to give an orange oil, and purified by flash column chromatography using 10% ethyl acetate in hexanes to yield:

S3, a colourless solid (1.05 g, 37%). Mp 131 – 135 °C; HRMS (ES) $[M+H]^+$ $C_7H_7Br^{79}{}_2N+H^+$, calc. 265.9003, obs. 265.8997; v_{max}/cm^{-1} 2967 (C-H stretch), 1563 (C-C stretch), 645 (C-Br stretch); ^1H-NMR (500 MHz, $CDCl_3$) δ 7.43 (1H, d, *J* = 1.1 Hz, 3-CH), 7.26 (1H, d, *J* = 1.1 Hz, 5-CH), 4.46 (2H, s, 7-CH_2), 2.53 (3H, s, 8-CH_3). ^{13}C-NMR (126 MHz, $CDCl_3$) δ 159.9 (6-C), 157.5 (2-C), 133.8 (4-C), 126.0 (3-C), 124.0 (5-C), 33.0 (7-C), 24.3 (8-C).

S5, a colourless solid (295 mg, 8%). Mp 94 – 98 °C; HRMS (ES) $[M+H]^+$ $C_7H_6Br^{79}{}_3N+H^+$, calc. 343.8108, obs. 343.8098; v_{max}/cm^{-1} 2937 (C-H stretch), 1570 (C-C stretch), 673, 656 (C-Br stretch); ^1H-NMR (500 MHz, $CDCl_3$) δ 7.79 (1H, s, 3-CH), 7.28 (1H, s, 5-CH), 6.54 (1H, s, 7-CH), 2.53 (3H, s, 8-CH_3). ^{13}C-NMR (101 MHz, $CDCl_3$) δ 159.5 (6-C), 158.9 (2-C), 134.0 (4-C), 127.2 (5-C), 122.7 (3-C), 40.6 (7-C), 24.2 (8-C).

S17, a colourless solid (441 mg, 12%). Mp 131 – 135 °C; HRMS (ES) [M+H]$^+$ $C_7H_6Br^{79}{}_3N+H^+$, calc. 343.8108, obs. 343.8101; v_{max}/cm^{-1} 2922 (C-H stretch), 1561 (C-C stretch), 605 (C-Br stretch); ^{1}H-NMR (500 MHz, $CDCl_3$) δ 7.55 (2H, s, 3-CH), 4.48 (4H, s, 5-CH_2). ^{13}C-NMR (101 MHz, $CDCl_3$) δ 158.0 (2-C), 134.4 (4-C), 126.6 (3-C), 32.5 (5-C).

Synthesis of ethyl 3-(4-bromo-6-methylpyridin-2-yl)propanoate S6

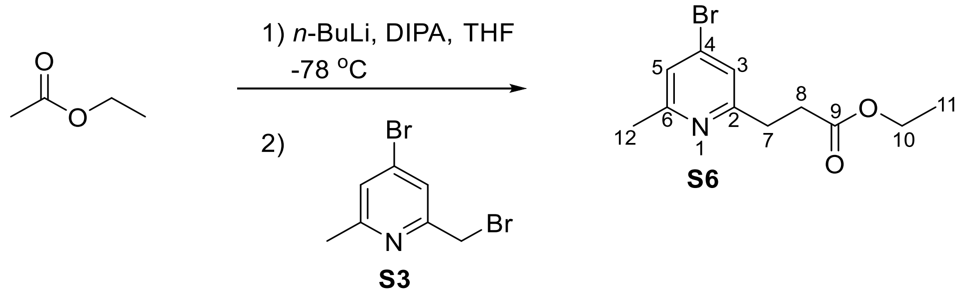

THF (10 mL) and diisopropylamine (650 μL, 4.74 mmol) were added to a 50 mL round-bottom flask and cooled to -78 °C. *n*-BuLi (2.5 M, 1.86 mL, 4.65 mmol) was added and the reaction was stirred for 1 h. Ethyl acetate (450 μL, 4.65 mmol) was added in THF (0.9 mL), dropwise, to the reaction mixture and stirred for 1 h. Next, 4-bromo-2-(dibromomethyl)-6-methylpyridine **S3** (1.23 g, 4.65 mmol) was added in THF (3.0 mL), in one portion. The reaction mixture was allowed to reach room temperature over 16h. The reaction mixture was reduced *in vacuo* and purified by flash column chromatography using 10% ethyl acetate in hexanes to yield **S6** as a colourless oil (768.3 mg, 61%). R_f 0.15 (10% ethyl acetate in hexanes); HRMS (ES) [M+H]$^+$ $C_{11}H_{14}Br^{79}NO_2+H^+$, calc. 272.0286, obs. 272.0309; v_{max}/cm^{-1} 2979 (C-H stretch), 1731 (CO), 1563 (C-C stretch); ^{1}H-NMR (500 MHz, $CDCl_3$) δ 7.15 (1H, dd, J = 1.7 Hz, 3-CH), 7.14 (1H, dd, J = 1.7 Hz, 5-CH), 4.10 (2H, q, J = 7.1 Hz, 10-CH_2), 3.01 (2H, t, J = 7.5 Hz, 7-CH_2), 2.72 (2H, t, J = 7.5 Hz, 8-CH_2), 2.45 (3H, s, 12-CH_3), 1.20 (3H, t, J = 7.1 Hz, 11-CH_3); ^{13}C-NMR (126 MHz, $CDCl_3$) δ 172.8 (9-C), 160.9 (2-C), 159.3 (6-C), 133.2 (4-C), 124.2 (5-C), 123.3 (3-C), 60.5 (10-C), 33.5 (8-C), 32.7 (7-C), 24.3 (12-C), 14.3 (11-C).

Synthesis of diethyl 2-[(4-bromo-6-methylpyridin-2-yl)methyl]butanedioate S7

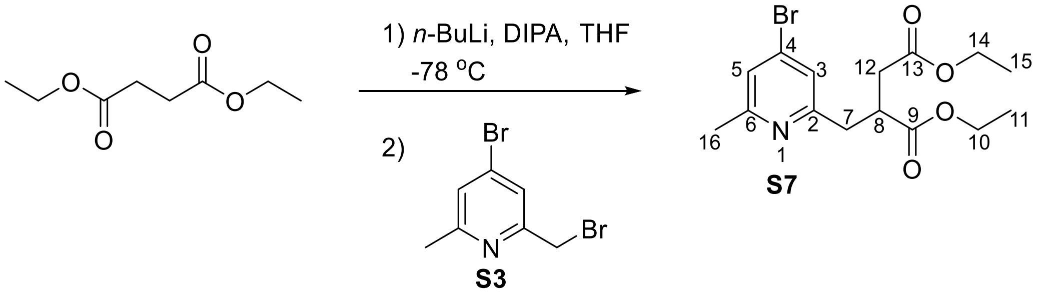

THF (0.86 mL) and diisopropylamine (55 µL, 0.38 mmol) were added to a 5 mL microwave vial and cooled to -78 °C. *n*-BuLi (2.5 M, 150 µL, 0.38 mmol) was added and the reaction was stirred for 1 h. Diethyl succinate (63 µL, 0.38 mmol) was added in THF (0.1 mL), dropwise, to the reaction mixture and stirred for 1 h. Next, 4-bromo-2-(bromomethyl)-6-methylpyridine **S3** (100 mg, 0.38 mmol) was added in THF (0.2 mL), in one portion. The reaction mixture was allowed to reach room temperature over 16 h. The reaction mixture was reduced *in vacuo* and purified by flash column chromatography using 60% diethyl ether in hexanes to yield **S7** as a colourless oil (68.5 mg, 50%). R_f 0.23 (60% diethyl ether in hexanes); HRMS (ES) $[M+H]^+$ $C_{15}H_{20}Br^{79}NO_4+H^+$, calc. 358.0654, obs. 358.0634; ν_{max}/cm^{-1} 2978 (C-H stretch), 1729 (CO), 1564 (C-C stretch), 1174 (C-O stretch); ^{1}H-NMR (500 MHz, $CDCl_3$) δ 7.17 (1H, d, *J* = 1.7 Hz, 3-CH), 7.13 (1H, d, *J* = 1.7 Hz, 5-CH), 4.15 – 4.07 (4H, m, 10-CH_2, 14-CH_2), 3.38 – 3.29 (1H, m, 8-CH), 3.11 (1H, dd, *J* = 14.0, 6.9 Hz, 7-CH), 2.91 (1H, dd, *J* = 14.0, 7.5 Hz, 7-CH), 2.69 (1H, dd, *J* = 16.7, 8.8 Hz, 12-CH), 2.51 – 2.45 (4H, m, 12-CH, 16-CH_3), 1.23 (3H, t, *J* = 7.1 Hz, 11-CH_3), 1.19 (3H, t, *J* = 7.1 Hz, 15-CH_3); ^{13}C-NMR (126 MHz, $CDCl_3$) δ 174.1 (9-C), 171.8 (13-C), 159.6 (6-C), 159.4 (2-C), 133.2 (4-C), 124.5 (3-C), 123.9 (5-C), 60.9 (10-C), 60.8 (14-C), 41.3 (8-C), 39.3 (7-C), 35.6 (12-C), 24.3 (16-C), 14.3 (11-C), 14.2 (15-C).

Synthesis of diethyl 2,2'-(((4-bromo-6-methylpyridin-2-yl)methyl)azanediyl)diacetate S8[63]

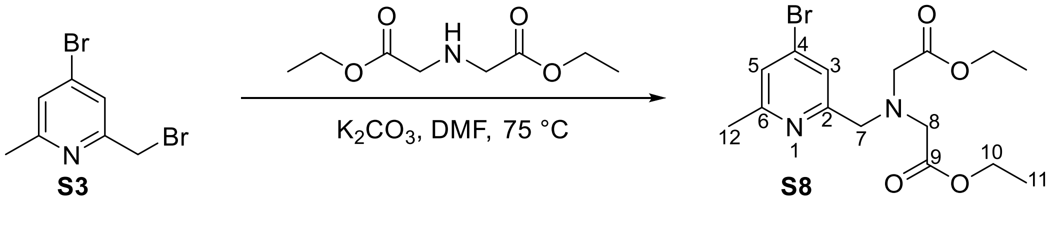

A 30 mL microwave vial was charged with diethyl iminodiacetate (9.9 mL, 5.5 mmol). Dimethylformamide (7.7 mL) was added, followed by potassium carbonate (1.14 g, 8.2 mmol). 4-Bromo-2-(bromomethyl)-6-methylpyridine **S3** (730 mg, 2.8 mmol) was added in dimethylformamide (7.3 mL). The reaction vessel was sealed and heated to 75 °C for 6 h. The reaction was allowed to cool and water (10 mL) was added. The mixture was taken up in diethyl ether (100 mL) and washed with water (5 × 20 mL). The organic layer was dried over Na_2SO_4, filtered, and the solvent removed. The mixture was purified by flash column chromatography using 70% diethyl ether in hexanes to yield **S8** as a yellow oil (750 mg, 73%). R_f 0.43 (70% diethyl ether in hexanes); HRMS (ES) $[M+H]^+$ $C_{15}H_{21}Br^{79}N_2O_4+H^+$, calc. 373.0763, obs. 373.0746; v_{max}/cm^{-1} 2926 (C-H stretch), 1736 (CO), 1566 (C-C stretch), 1192 (C-O stretch); ^{1}H-NMR (500 MHz, $CDCl_3$) δ 7.66 (1H, s, 5-CH), 7.22 (1H, s, 3-CH), 4.16 (4H, q, *J* = 7.2 Hz, 9-CH_2), 4.02 (2H, s, 7-CH_2), 3.59 (4H, s, 8-CH_2), 2.50 (3H, s, 12-CH_3), 1.26 (6H, t, *J* = 7.2 Hz, 11-CH_3); ^{13}C-NMR (126 MHz, $CDCl_3$) δ 171.2 (9-C), 160.2 (2-C), 159.0 (6-C), 134.3 (4-C), 125.2 (3-C), 123.4 (5-C), 60.8 (10-C), 59.6 (7-C), 55.2 (8-C), 24.1 (12-C), 14.4 (11-C).

Synthesis of 2-(4-bromo-6-methylpyridin-2-yl)acetonitrile S9[64a-b]

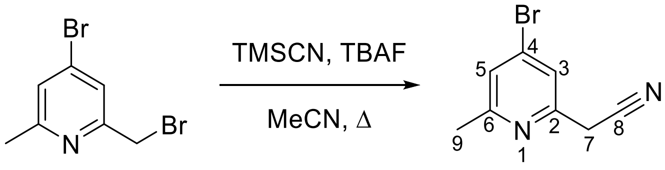

S3 **S9**

4-Bromo-2-(bromomethyl)-6-methylpyridine **S3** (500 mg, 1.89 mmol), acetonitrile (19 mL), trimethylsilyl cyanide (0.35 mL, 2.80 mmol), and TBAF (2.85 mL, 2.85 mmol) were added to a 50 mL round-bottom flask. The reaction was heated to reflux for 15 min. The reaction was allowed to cool, and ammonium hydroxide was added. The reaction mixture was extracted with ethyl acetate (3 × 30 mL). The organics were dried over $MgSO_4$, filtered, and reduced *in vacuo*. The crude reaction mixture was purified by flash column chromatography using 20% ethyl acetate in hexanes to yield **S9** as an off-white solid (362 mg, 91%). Mp 73 – 77 °C; R_f 0.12 (20% ethyl acetate in hexanes); R_f 0.55 (20% ethyl acetate in dichloromethane); HRMS (ES) $[M+H]^+$ $C_8H_7Br^{79}N_2+H^+$, calc. 210.9871, obs. 210.9865; v_{max}/cm^{-1} 3073, 2901 (C-H stretch), 2245 (CN stretch), 1568 (C-C stretch), 1395, 839, 824; ^{1}H-NMR (400 MHz,

CDCl3) δ 7.43 (1H, s, 3-CH), 7.31 (1H, s, 5-CH), 3.86 (2H, s, 7-CH_2), 2.52 (3H, s, 9-CH_3); ^{13}C-NMR (101 MHz, $CDCl_3$) δ 160.4 (6-C), 151.1 (2-C), 134.2 (4-C), 126.0 (5-C), 122.7 (3-C), 116.7 (8-C), 26.4 (7-C), 24.2 (9-C).

Synthesis of methyl 2-(4-bromo-6-methylpyridin-2-yl)acetate S10[65]

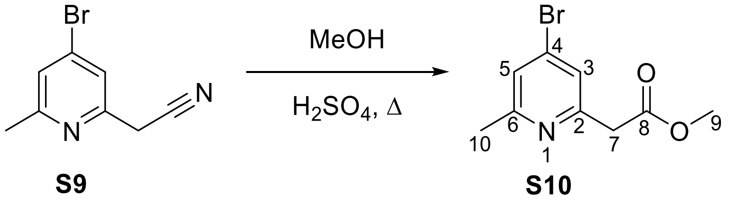

A 50 mL round-bottom flask was charged with 2-(4-bromo-6-methylpyridin-2-yl)acetonitrile **S9** (1.18 g, 5.59 mmol) and methanol (11.8 mL). Sulphuric acid (3.7 mL) was added dropwise. The reaction was heated to reflux overnight. Upon completion, the reaction mixture was added to saturate sodium bicarbonate solution, and extracted with dichloromethane (3 × 30 mL). The organics were dried over $MgSO_4$, filtered, and reduced *in vacuo* to yield **S10** as a colourless oil (1.35 g, 99%). R_f 0.41 (20% ethyl acetate in dichloromethane) HRMS (ES) $[M+H]^+$ $C_9H_{10}Br^{79}NO_2+H^+$, calc. 243.9973, obs. 243.9880; v_{max}/cm^{-1} 2952, 2927 (C-H stretch), 1736 (CO), 1562 (C-C stretch), 1431, 1272, 1198, 1163 (C-O stretch); ^{1}H-NMR (500 MHz, $CDCl_3$) δ 7.31 (1H, d, *J* = 1.1 Hz, 3-CH), 7.26 (1H, d, *J* = 1.1 Hz, 5-CH), 3.80 (2H, s, 7-CH_2), 3.73 (3H, s, 9-CH_3), 2.52 (3H, s, 10-CH_3); ^{13}C-NMR (126 MHz, $CDCl_3$) δ 170.7 (8-C), 159.6 (6-C), 154.9 (2-C), 133.4 (4-C), 125.0 (5-C), 124.2 (3-C), 52.3 (7-C), 43.4 (9-C), 24.3 (10-C).

Synthesis of 2-(4-bromo-6-methylpyridin-2-yl)ethan-1-ol S11 (72) and 2-(6-methylpyridin-2-yl)ethan-1-ol S11a[66]

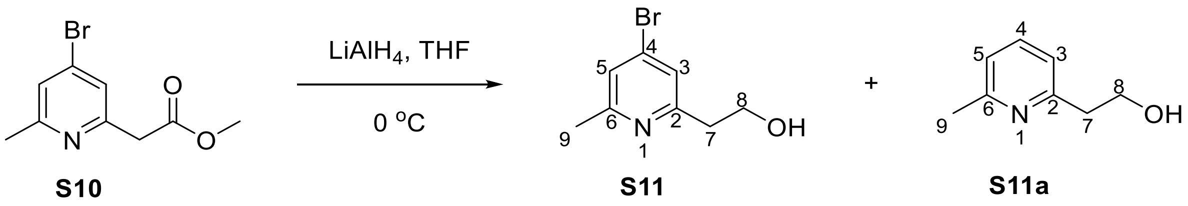

A 250 mL round-bottom flask was charged with 2-(4-bromo-6-methylpyridin-2-yl)acetate **S10** (1.35 g, 5.53 mmol) in THF (55 mL). The vessel was cooled to 0 °C, and lithium aluminium

hydride (2.4 M, 4.6 mL, 11.0 mmol) in THF was added dropwise, and stirred for 5 min. Diethyl ether (100 mL) was added, followed by water (2 mL) dropwise. The reaction mixture was then reduced *in vacuo*, taken up in ethyl acetate (100 mL) and washed with water (3 × 30 mL). The organics were dried over Na_2SO_4, filtered and the solvent removed. The mixture was purified by flash column chromatography using ethyl acetate to yield:

S11, a colourless oil (680.9 mg, 57%). R_f 0.19 (ethyl acetate); HRMS (ES) $[M+H]^+$ $C_8H_{10}Br^{79}NO+H^+$, calc. 216.0024, obs. 216.0016; v_{max}/cm^{-1} 3261 (OH), 2922, 2869 (C-H stretch), 1566 (C-C stretch), 1048 (C-O stretch); ^{1}H-NMR (500 MHz, $CDCl_3$) δ 7.19 (1H, s, 3-CH), 7.15 (1H, s, 5-CH), 4.35 (1H, s, OH), 3.96 (2H, t, *J* = 5.5 Hz, 8-CH_2), 2.92 (1H, t, *J* = 5.5 Hz, 7-CH_2), 2.48 (3H, s, 9-CH_3); ^{13}C-NMR (126 MHz, $CDCl_3$) δ 161.4 (2-C), 159.1 (6-C), 133.6 (4-C), 124.4 (3-C), 123.7 (5-C), 61.6 (8-C), 38.9 (7-C), 24.2 (9-C).

S11a, a colourless oil (92 mg, 12%). R_f 0.12 (ethyl acetate); HRMS (ES) $[M+H]^+$ $C_8H_{11}NO+H^+$, calc. 137.0841, obs. 137.0796; v_{max}/cm^{-1} 3283 (OH), 2926 (C-H stretch), 1595, 1577, 1458 (C-C stretch), 1044 (C-O stretch); ^{1}H-NMR (500 MHz, $CDCl_3$) δ 7.50 (1H, t, *J* = 7.7 Hz, 4-CH), 7.00 (1H, d, *J* = 7.7 Hz, 3-CH), 6.94 (1H, d, *J* = 7.7 Hz, 5-CH), 4.81 (1H, s, OH), 3.99 (2H, t, *J* = 5.3 Hz, 8-CH_2), 2.96 (2H, t, *J* = 5.3 Hz, 7-CH_2), 2.51 (s, 3H); ^{13}C-NMR (126 MHz, $CDCl_3$) δ 160.3 (2-C), 157.7 (6-C), 137.1 (4-C), 121.1 (3-C), 120.3 (5-C), 62.1 (8-C), 38.7 (7-C), 24.5 (9-C).

Synthesis of 2-(4-bromo-6-methylpyridin-2-yl)ethyl 4-methylbenzenesulfonate S12[67]

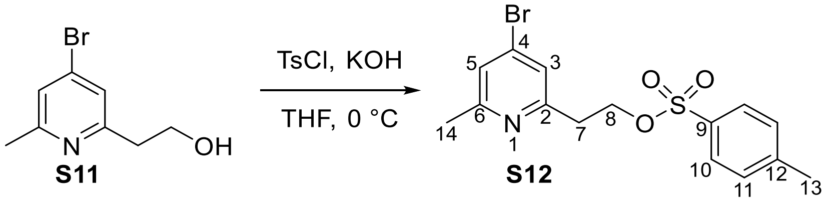

A 30 mL microwave vial was charged with 2-(4-bromo-6-methylpyridin-2-yl)ethan-1-ol **S11** (600 mg, 2.78 mmol) and THF (14.5 mL), and cooled to 0 °C. Freshly ground potassium hydroxide (235 mg, 4.19 mmol) was added, and the reaction was stirred for 10 min. *p*-Toluenesulphonly chloride (690 mg, 3.26 mmol) was added, and the reaction was allowed to warm to room temperature overnight. Upon completion, the reaction mixture was poured into ethyl acetate (50 mL), and washed with water (3 × 10 mL) and brine (10 mL). The organics were dried over Na_2SO_4, filtered, and reduced *in vacuo*. The resultant material was

purified by flash column chromatography using 30% ethyl acetate in hexanes to yield **S12** as a colourless solid (758.2 mg, 74%). Mp 88 – 92 °C; R_f 0.15 (30% ethyl acetate in hexanes); HRMS (ES) $[M+H]^+$ $C_{15}H_{16}NO_3S+H^+$, calc. 370.0113, obs. 370.0123; v_{max}/cm^{-1} 2923 (C-H stretch), 1564, 1355 (S=O stretch), 1173 (C-O stretch); ^{1}H-NMR (500 MHz, $CDCl_3$) δ 7.68 – 7.64 (2H, m, 10-CH), 7.29 – 7.25 (2H, m, 11-CH), 7.14 (1H, d, *J* = 1.2 Hz, 3-CH), 7.06 (1H, d, *J* = 1.2 Hz, 5-CH), 4.40 (2H, t, *J* = 6.4 Hz, 8-CH_2), 3.01 (2H, t, *J* = 6.4 Hz, 7-CH_2), 2.43 (3H, s, 14-CH_3), 2.38 (3H, s, 13-CH_3); ^{13}C-NMR (126 MHz, $CDCl_3$) δ 159.6 (6-C), 157.4 (2-C), 144.8 (12-C), 133.3 (4-C), 133.0 (9-C), 129.9 (11-C), 128.0 (10-C), 124.7 (3-C), 124.1 (5-C), 69.3 (8-C), 37.2 (7-C), 24.2 (13-C), 21.8 (14-C).

Synthesis of diethyl 2,2'-((2-(4-bromo-6-methylpyridin-2-yl)ethyl)azanediyl)diacetate S13[68]

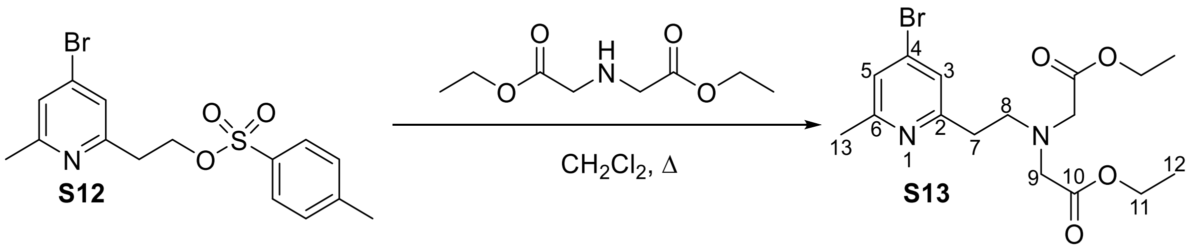

To a 30 mL microwave vial was added 2-(4-bromo-6-methylpyridin-2-yl)ethyl 4-methylbenzenesulfonate **S12** (760 mg, 2.05 mmol), dichloromethane (20 mL), and diethyl iminodiacetate (1.1 mL, 6.1 mmol). The mixture was heated to reflux for 48 h. Upon completion, the reaction mixture was poured into saturated sodium carbonate solution (20 mL). The organic phase was separated, and the aqueous phase was extracted with ethyl acetate (3 × 30 mL). The organics were combined, dried over Na_2SO_4, filtered, and the solvent removed *in vacuo*. The mixture was purified by flash column chromatography 50% ethyl acetate in hexanes to yield **S13** as a colourless oil (348.9 mg, 44%). R_f 0.25 (50% ethyl acetate in hexanes); HRMS (ES) $[M+H]^+$ $C_{16}H_{23}Br^{79}N_2O_4+H^+$, calc. 387.0919, obs. 387.0899; v_{max}/cm^{-1} 2971 (C-H stretch), 1737 (CO), 1563 (C-C stretch), 1184 (CO); ^{1}H-NMR (500 MHz, $CDCl_3$) δ 7.21 (1H, d, *J* = 1.3 Hz, 3-CH), 7.15 (1H, d, *J* = 1.3 Hz, 5-CH), 4.15 (4H, q, *J* = 7.2 Hz, 11-CH_2), 3.56 (4H, s, 9-CH_2), 3.10 (2H, dd, *J* = 8.6, 6.6 Hz, 8-CH_2), 2.90 (2H, dd, *J* = 8.6, 6.6 Hz, 7-CH_2), 2.47 (3H, s, 13-CH_3), 1.25 (6H, t, *J* = 7.2 Hz, 12-CH_3); ^{13}C-NMR (126 MHz, $CDCl_3$) δ 171.4 (10-C), 160.8 (6-C), 159.3 (2-C), 133.2 (4-C), 124.1 (5-C), 123.6 (3-C), 60.7 (11-C), 55.4 (9-C), 54.4 (8-C), 37.1 (7-C), 24.3 (13-C), 14.4 (12-C).

Synthesis of 3-(4-bromo-6-methylpyridin-2-yl)propan-1-ol S14 and 3-(6-methylpyridin-2-yl)propan-1-ol S14a [66]

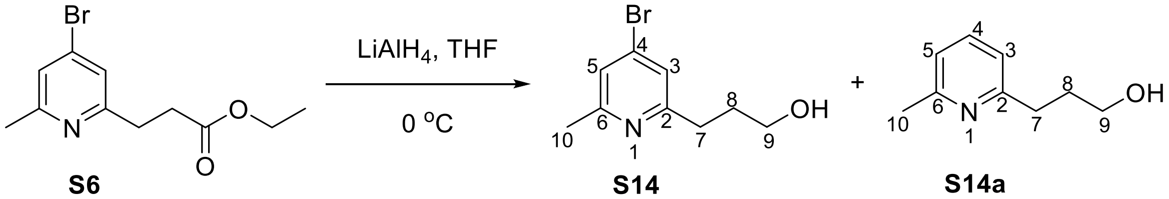

S6 **S14** **S14a**

A 5 mL microwave vial was charged with ethyl 3-(4-bromo-6-methylpyridin-2-yl)propanoate **S6** (100 mg, 0.37 mmol) in THF (3.7 mL). The vessel was cooled to 0 °C, and lithium aluminium hydride (2.4 M, 0.3 mL, 0.73 mmol) in THF was added dropwise, and stirred for 5 min. Diethyl ether (2 mL) was added, followed by water (1 mL) dropwise. The reaction mixture was then taken up in ethyl acetate (10 mL) and washed with water (3 × 10 mL). The organics were dried over Na_2SO_4, filtered and the solvent removed. The mixture was purified by flash column chromatography using ethyl acetate to yield:

S14, a colourless oil (74.8 mg, 88%). R_f 0.22 (ethyl acetate); HRMS (ES) $[M+H]^+$ $C_9H_{12}Br^{79}NO+H^+$, calc. 230.1081, obs. 230.0176; v_{max}/cm^{-1} 3295 (OH), 2951, 2930, 2857 (C-H stretch), 1562 (C-C stretch), 1055 (C-O stretch); ^{1}H-NMR (500 MHz, $CDCl_3$) δ 7.17 (2H, app. s, 3-CH, 5-CH), 3.77 (1H, s, OH), 3.68 (2H, t, *J* = 5.8 Hz, 9-CH_2), 2.88 (2H, t, *J* = 6.8 Hz, 7-CH_2), 2.48 (3H, s, 10-CH_3), 1.98 – 1.89 (2H, m, 8-CH_2); ^{13}C-NMR (126 MHz, $CDCl_3$) δ 162.3 (2-C), 159.0 (6-C), 133.8 (4-C), 124.1 (3-C), 123.5 (5-C), 62.2 (9-C), 35.3 (7-C), 31.6 (8-C), 24.0 (10-C).

S14a, a colourless oil (5.5 mg, 10%). R_f 0.11 (ethyl acetate); HRMS (ES) $[M+H]^+$ $C_9H_{13}NO+H^+$, calc. 152.1075, obs. 152.1026; v_{max}/cm^{-1} 3316 (OH), 2925 (C-H stretch), 1594, 1577, 1458 (C-C stretch), 1058 (C-O stretch); ^{1}H-NMR (500 MHz, $CDCl_3$) δ 7.46 (1H, app. t, *J* = 7.7 Hz, 4-CH), 6.95 (1H, d, *J* = 7.7, 3-CH), 6.94 (1H, d, *J* = 7.7, 5-CH), 4.27 (1H, s, OH), 3.67 (2H, t, *J* = 5.8 Hz, 9-CH_2), 2.89 (2H, t, *J* = 6.8 Hz, 7-CH_2), 2.47 (3H, s, 10-CH_3), 1.96 – 1.86 (2H, m, 8-CH_2); ^{13}C-NMR (126 MHz, $CDCl_3$) δ 160.8 (2-C), 157.4 (6-C), 137.2 (4-C), 120.8 (3-C), 120.1 (4-C), 62.2 (9-C), 35.5 (7-C), 31.8 (8-C), 24.1 (10-C).

Synthesis of 3-(4-bromo-6-methylpyridin-2-yl)propanal S15

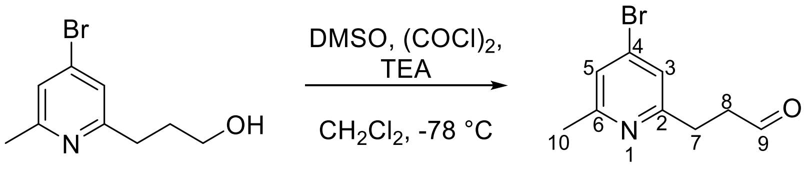

S14 **S15**

To a 30 mL microwave vial was added dichloromethane (5.6 mL). The reaction vessel was cooled to -78 °C. Dimethyl sulphoxide (270 μL, 3.7 mmol) was added followed by the dropwise addition of oxalyl chloride (160 μL, 1.8 mmol). After 15 min, 3-(4-bromo-6-methylpyridin-2-yl)propan-1-ol **S14** (324 mg, 1.4 mmol) was added in dichloromethane (2.7 mL). After 15 min, triethylamine (0.4 mL, 2.9 mmol) was added, and the reaction turned gradually from orange to black. After a further 15 min, more triethylamine (0.4 mL, 2.9 mmol) was added, and the reaction was warmed to 0 °C. 15 min afterwards, water (10.2 mL, 0.57 mmol) was added, and the reaction mixture was poured into saturated sodium bicarbonate solution (20 mL). The organic phase was removed and the aqueous phase was washed with dichloromethane (3 × 15 mL). The organics were dried over Na_2SO_4, filtered, and the volatiles removed. The mixture was purified by flash column chromatography using 30% ethyl acetate in hexanes to yield **S15** as a colourless oil (251 mg, 78%). R_f 0.16 (30% ethyl acetate in hexanes); HRMS (ES) $[M+H]^+$ $C_9H_{10}Br^{79}NO+H^+$, calc. 228.0024, obs. 228.0019; v_{max}/cm^{-1} 2915 (C-H stretch), 1719 (CO), 1564 (C-C stretch); ^{1}H-NMR (500 MHz, $CDCl_3$) δ 9.84 (1H, s, 9-CH), 7.17 (1H, s, 3-CH), 7.16 (1H, s, 5-CH), 3.04 (2H, t, *J* = 7.2 Hz, 7-CH_2), 2.89 (2H, t, *J* = 7.2 Hz, 8-CH_2), 2.46 (3H, s, 10-CH_3); ^{13}C-NMR (126 MHz, $CDCl_3$) δ 201.3 (9-C), 160.6 (2-C), 159.4 (6-C), 133.3 (4-C), 124.3 (5-C), 123.3 (3-C), 42.7 (8-C), 30.3 (7-C), 24.3 (10-C).

Synthesis of diethyl 2,2'-{[3-(4-bromo-6-methylpyridin-2-yl)propyl]azanediyl}diacetate S16

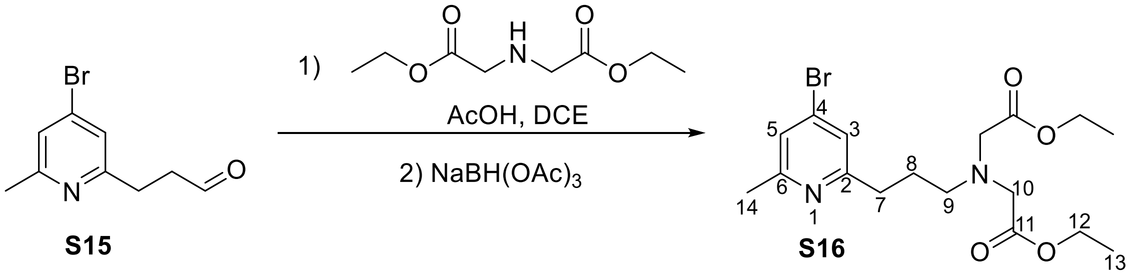

To a 100 mL round-bottom flask was added 3-(4-bromo-6-methylpyridin-2-yl)propanal **S15** (1.14 g, 5.0 mmol), 1,2-dichloroethane (36 mL), diethyl iminodiacetate (1.8 mL, 10.0 mmol), and acetic acid (0.6 mL, 10.5 mmol). The mixture was stirred for 30 min, followed by the addition of sodium triacetoxyborohydride (2.12 g, 10.0 mmol). After 16 h, the reaction mixture was poured into saturated sodium carbonate solution (50 mL), and extracted with dichloromethane (3 × 100 mL). The organics were combined, dried over Na_2SO_4, filtered, and the solvent removed *in vacuo*. The mixture was purified by flash column chromatography using 20% ethyl acetate in hexanes to yield **S16** as a colourless oil (1.53 g, 76%). R_f 0.11 (30% ethyl acetate in hexanes); HRMS (ES) $[M+H]^+$ $C_{17}H_{25}Br^{79}N_2O_4+H^+$, calc. 401.1076, obs. 401.1048; v_{max}/cm^{-1} 2980 (C-H stretch), 1735 (CO), 1563 (C-C stretch), 1183 (CO); ^{1}H-NMR (500 MHz, $CDCl_3$) δ 7.14 (1H, d, *J* = 1.8 Hz, 5-CH), 7.13 (1H, d, *J* = 1.8 Hz, 3-CH), 4.13 (4H, q, *J* = 7.1 Hz, 12-CH_2), 3.52 (4H, s, 10-CH_2), 2.78 – 2.68 (4H, m, 7-CH_2, 9-CH_2), 2.46 (3H, s, 14-CH_3), 1.90 – 1.79 (m, 8-CH_2), 1.23 (6H, t, *J* = 7.1 Hz, 13-CH_3); ^{13}C-NMR (126 MHz, $CDCl_3$) δ 171.4 (11-C), 162.7 (2-C), 159.2 (6-C), 133.2 (4-C), 123.9 (3-C), 123.2 (5-C), 60.6 (12-C), 55.1 (10-C), 53.8 (9-C), 35.5 (7-C), 28.0 (8-C), 24.3 (14-C), 14.3 (15-C).

Synthesis of 4-bromo-2,6-bis(bromomethyl)pyridine S17[69]

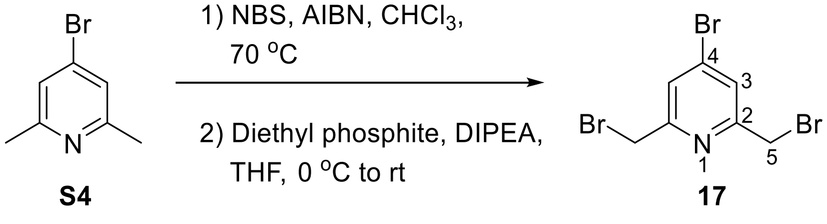

To a 5 mL microwave vial was added $CHCl_3$ (1.8 mL), 4-bromolutidine **S4** (100 mg, 0.54 mmol), *N*-bromosuccinimide (480 mg, 2.69 mmol), and 2,2′-azobis(2-methylpropionitrile) (9

mg, 0.05 mmol). The reaction was heated to reflux, and monitored by TLC and allowed to proceed until any known dibrominated species were no longer detected. Upon completion, the solvent was removed *in vacuo* and the reaction mixture neutralised with saturated sodium bicarbonate solution (5 mL). The aqueous mixture was extracted with dichloromethane (3 × 15 mL), the organics were combined, dried over Na_2SO_4 and filtered. The crude mixture was added to a 5 mL microwave vial and dissolved in THF (2 mL). The solution was cooled to 0 °C and diethyl phosphite (140 μL, 1.07 mmol) and DIPEA (190 μL, 1.07 mmol) were added. The reaction was monitored by TLC and allowed to proceed until analysis showed no sign of change. Upon completion, the solvent was removed *in vacuo* and the reaction mixture neutralised with saturated sodium bicarbonate solution (5 mL). The aqueous mixture was extracted with dichloromethane (3 × 15 mL), the organics were combined, dried over Na_2SO_4 and filtered. The crude mixture was purified by flash column chromatography using 10% ethyl acetate in hexanes to yield **S17** as a colourless solid (134.4 mg, 72%). Characterisation data matches that of **S17** given above.

Synthesis of diethyl 3,3'-(4-bromopyridine-2,6-diyl)dipropanoate S18

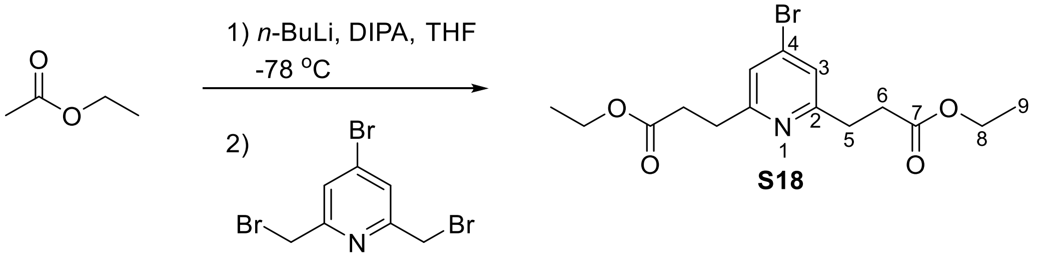

THF (5.8 mL) and diisopropylamine (615 μL, 4.39 mmol) were added to a 30 mL microwave vial and cooled to -78 °C. *n*-BuLi (2.5 M, 1.7 mL, 4.25 mmol) was added and the reaction was stirred for 1 h. Ethyl acetate (415 μL, 4.25 mmol) was added in THF (2.0 mL), dropwise, to the reaction mixture and stirred for 1 h. Next, 4-bromo-2,6-bis(bromomethyl)pyridine **S17** (737 mg, 2.14 mmol) was added in THF (2.2 mL), in one portion. The reaction mixture was allowed to reach room temperature over 16h. The reaction mixture was reduced *in vacuo* and purified by flash column chromatography using 10% ethyl acetate in hexanes to yield **S18** as a colourless oil (428.8 mg, 56%). HRMS (ES) $[M+H]^+$ $C_{15}H_{20}Br^{79}NO_4+H^+$, calc. 358.0654, obs. 358.0652; ν_{max}/cm^{-1} 2978 (C-H stretch), 1729 (CO), 1563 (C-C stretch), 1176 (C-O stretch); ^{1}H-NMR (400 MHz, $CDCl_3$) δ 7.18 (2H, s, 3-CH), 4.12 (4H, q, *J* = 7.1 Hz, 8-

CH2), 3.03 (4H, t, J = 7.4 Hz, 5-CH2), 2.75 (4H, t, J = 7.4 Hz, 6-CH2), 1.23 (6H, t, J = 7.1 Hz, 9-CH3); ^{13}C-NMR (101 MHz, $CDCl_3$) δ 173.1 (7-C), 161.0 (2-C), 133.1 (4-C), 123.9 (3-C), 60.6 (8-C), 33.0 (6-C), 32.6 (5-C), 14.3 (9-C).

Synthesis of ethyl 3-[4-bromo-6-(bromomethyl)pyridin-2-yl]propanoate S19

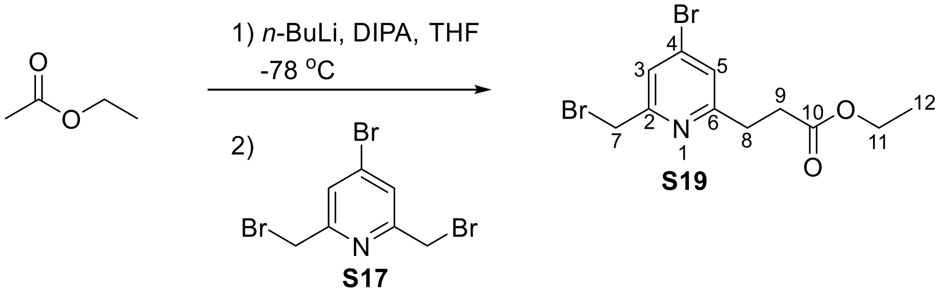

THF (5.8 mL) and diisopropylamine (615 µL, 4.39 mmol) were added to a 30 mL microwave vial and cooled to -78 °C. *n*-BuLi (2.5 M, 1.7 mL, 4.25 mmol) was added and the reaction was stirred for 1 h. Ethyl acetate (415 µL, 4.25 mmol) was added in THF (2.0 mL), dropwise, to the reaction mixture and stirred for 1 h. Next, 4-bromo-2,6-bis(bromomethyl)pyridine **S17** (737 mg, 2.14 mmol) was added in THF (2.2 mL), in one portion. The reaction mixture was allowed to reach room temperature over 16h. The reaction mixture was reduced *in vacuo* and purified by flash column chromatography using 10% ethyl acetate in hexanes to yield **S19** as a colourless oil (237.3 mg, 33%). HRMS (ES) $[M+H]^+$ $C_{11}H_{13}Br^{79}{}_2NO_2+H^+$, calc. 351.9371, obs. 351.9361; v_{max}/cm^{-1} 2991 (C-H stretch), 1725 (CO), 1563 (C-C stretch), 1194, 1154 (C-O stretch); ^{1}H-NMR (500 MHz, $CDCl_3$) δ 7.44 (1H, d, J = 1.5 Hz, 3-CH), 7.29 (1H, d, J = 1.5 Hz, 5-CH), 4.43 (2H, s, 7-CH2), 4.13 (2H, q, J = 7.1 Hz, 11-CH2), 3.07 (2H, t, J = 7.3 Hz, 8-CH2), 2.78 (2H, t, J = 7.3 Hz, 9-CH2), 1.23 (3H, t, J = 7.1 Hz, 12-CH3); ^{13}C-NMR (126 MHz, $CDCl_3$) δ 172.8 (10-C), 161.6 (6-C), 157.6 (2-C), 133.7 (4-C), 125.7 (3-C), 124.5 (5-C), 60.6 (11-C), 33.0 (9-C), 33.0 (7-C), 32.5 (8-C), 14.3 (12-C).

Synthesis of ethyl 3-[6-(azidomethyl)-4-bromopyridin-2-yl]propanoate S20

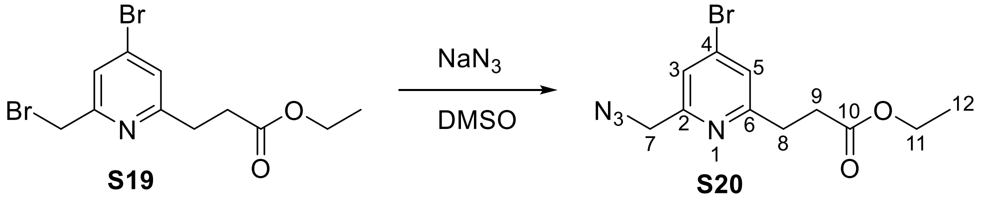

A 30 mL microwave vial was charged with ethyl 3-[4-bromo-6-(bromomethyl)pyridin-2-yl]propanoate **S19** (739.2 mg, 2.11 mmol), DMSO (10 mL), and sodium azide (205 mg, 3.16 mmol). The reaction mixture was stirred at room temperature for 3 h. The solution was poured into diethyl ether (100 mL) and washed with water (5 × 50 mL). The organics were dried over Na_2SO_4, filtered and the solvent volume reduced *in vacuo* to yield **S20** as a yellow oil (623.2 mg, 94%). HRMS (ES) $[M+H]^+$ $C_{11}H_{14}Br^{79}N_4O_2+H^+$, calc. 313.0300, obs. 313.0297; v_{max}/cm^{-1} 2927 (C-H stretch), 2101 (N_3), 1729 (CO), 1565 (C-C stretch), 1258, 1181 (C-O stretch); ^{1}H-NMR (500 MHz, $CDCl_3$) δ 7.33 (1H, d, *J* = 1.7 Hz, 5-CH), 7.32 (1H, d, *J* = 1.7 Hz, 3-CH), 4.37 (2H, s, 7-CH_2), 4.11 (2H, q, *J* = 7.1 Hz, 11-CH_2), 3.06 (2H, t, *J* = 7.3 Hz, 8-CH_2), 2.78 (2H, t, *J* = 7.3 Hz, 9-CH_2), 1.22 (3H, t, *J* = 7.1 Hz, 12-CH_3); ^{13}C-NMR (126 MHz, $CDCl_3$) δ 172.8 (10-C), 161.7 (6-C), 156.7 (2-C), 133.9 (4-C), 125.6 (5-C), 122.9 (3-C), 60.6 (11-C), 55.0 (7-C), 32.8 (9-C), 32.4 (8-C), 14.3 (12-C).

Synthesis of ethyl 3-(4-bromo-6-{[4-(2-hydroxypropan-2-yl)-1H-1,2,3-triazol-1-yl]methyl}pyridin-2-yl)propanoate S21

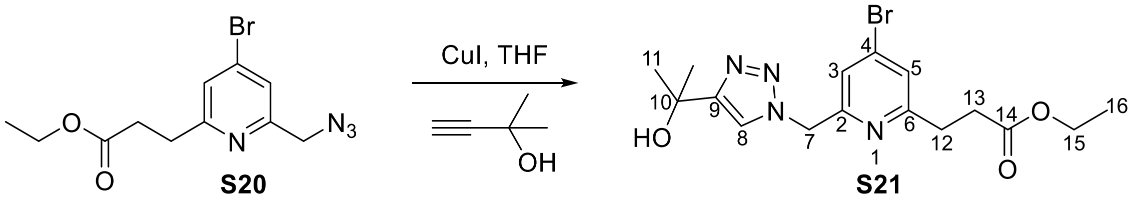

A 5 mL microwave vial was charged with 3-[6-(azidomethyl)-4-bromopyridin-2-yl]propanoate **S20** (100 mg, 0.32 mmol), copper iodide (6.1 mg, 0.03 mmol), THF (1.6 mL), and methyl-3-butyn-2-ol (50 µL, 0.48 mmol). The reaction was stirred at room temperature for 16h. Upon completion, the solvent volume was reduced *in vacuo*, and the residue taken up in ethyl acetate (15 mL). The organic solution was washed with $EDTA_{(aq)}$ (5 mL), water (3 × 10 mL), and brine (5 mL). The organics were dried over Na_2SO_4, filtered and the solvent removed. The mixture was purified by flash column chromatography using 50% dichloromethane in

acetone to yield **S21** as a colourless oil (121.8 mg, 96%). HRMS (ES) $[M+H]^+$ $C_{16}H_{21}Br^{79}N_4O_3+Na^+$, calc. 419.0695, obs. 419.0674; $ν_{max}/cm^{-1}$ 2976 (C-H stretch), 1725 (CO), 1566 (C-C stretch), 1373 (C-H), 1173 (C-O stretch); ^{1}H-NMR (400 MHz, $CDCl_3$) δ 7.61 (1H, s, 8-CH), 7.30 (1H, s, 3-CH), 7.16 (1H, s, 5-CH), 5.50 (2H, s, 7-CH_2), 4.06 (2H, q, *J* = 7.1 Hz, 15-CH_2), 3.15 – 3.01 (3H, m, OH, 12-CH_2), 2.73 (1H, t, *J* = 7.2 Hz, 13-CH_2), 1.61 (6H, s, 11-CH_3), 1.17 (3H, t, *J* = 7.1 Hz, 16-CH_3); ^{13}C-NMR (101 MHz, $CDCl_3$) δ 172.7 (14-C), 161.7 (6-C), 156.3 (9-C), 155.0 (2-C), 134.0 (4-C), 126.0 (3-C), 123.3 (5-C), 120.2 (8-C), 68.5 (10-C), 60.6 (15-C), 55.0 (7-C), 32.7 (13-C), 32.2 (12-C), 30.5 (11-C), 14.2 (16-C).

Synthesis of ethyl 3-(4-bromo-6-{[(*t*-butoxycarbonyl)amino]methyl}pyridin-2-yl)propanoate S22

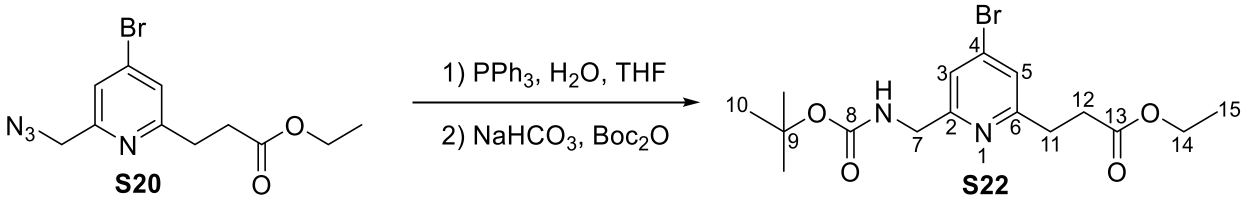

A 5 mL microwave vial was charged with ethyl 3-[6-(azidomethyl)-4-bromopyridin-2-yl]propanoate **S20** (100 mg, 0.32 mmol). The solid was dissolved in THF (2.2 mL), and triphenylphosphine (126 mg, 0.48 mmol) was added and the reaction stirred at room temperature. Once the effervescence has ceased, water (580 µL, 32 mmol) was added and the reaction was stirred for 16h. Solid sodium bicarbonate (300 mg, 3.57 mmol) was added, followed by Boc_2O (105 mg, 0.48 mmol), and the reaction stirred at room temperature overnight. The solvent volume was reduced *in vacuo*, and the remaining solids taken up in ethyl acetate (20 mL) and washed with water (3 × 10 mL) and brine (10 mL). The organics were dried over Na_2SO_4, filtered and the solvent removed. The colourless solid was purified by flash column chromatography using 30% ethyl acetate in hexanes to yield **S22** as a colourless oil (116.2 mg, 94%). HRMS (ES) $[M+H]^+$ $C_{16}H_{23}Br^{79}N_2O_4+H^+$, calc. 387.0919, obs. 387.0930; $ν_{max}/cm^{-1}$ 2976 (C-H stretch), 1711 (CO), 1566 (C-C stretch), 1247, 1164 (C-O stretch); ^{1}H-NMR (500 MHz, $CDCl_3$) δ 7.24 (1H, d, *J* = 1.7 Hz, 3-CH), 7.22 (1H, d, *J* = 1.7 Hz, 5-CH), 5.52 (1H, br. d, *J* = 5.2 Hz, NH), 4.34 (2H, d, *J* = 5.2 Hz, 7-CH_2), 4.11 (2H, q, *J* = 7.1 Hz, 14-CH_2), 3.03 (2H, t, *J* = 7.3 Hz, 11-CH_2), 2.73 (2H, t, *J* = 7.3 Hz, 12-CH_2), 1.44 (9H, s, 10-CH_3), 1.21 (3H, t, *J* = 7.1 Hz, 15-CH_3); ^{13}C-NMR (126 MHz, $CDCl_3$) δ 172.9 (8-C),

161.0 (6-C), 158.5 (2-C), 156.0 (8-C), 133.7 (4-C), 124.7 (5-C), 122.4 (3-C), 79.7 (9-C), 60.6 (14-C), 45.4 (7-C), 33.1 (12-C), 32.4 (11-C), 28.5 (10-C), 14.3 (15-C).

Synthesis of 2,6-bis(azidomethyl)-4-bromopyridine S23

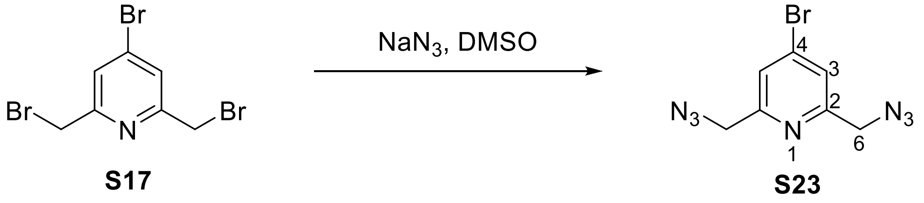

A 5 mL microwave vial was charged with 4-bromo-2,6-bis(bromomethyl)pyridine **S17** (100 mg, 0.29 mmol), DMSO (2 mL), and sodium azide (60 mg, 0.92 mmol). The reaction mixture was stirred at room temperature for 3 h. The solution was poured into diethyl ether (20 mL) and washed with water (5 × 10 mL). The organics were dried over Na_2SO_4, filtered and the solvent volume reduced *in vacuo* to yield **S23** as a colourless solid (75.4 mg, 97%). Mp 56 – 60 ° C; HRMS (ES) $[M+H]^+$ $C_7H_6Br^{79}N_7+H^+$, calc. 267.9946, obs. 267.9935; ν_{max}/cm^{-1} 2087 (N_3), 1572 (C-C stretch); ^{1}H-NMR (400 MHz, $CDCl_3$) δ 7.47 (2H, s, 3-CH), 4.46 (4H, s, 6-CH_2); ^{13}C-NMR (101 MHz, $CDCl_3$) δ 157.3 (2-C), 134.8 (4-C), 124.4 (3-C), 54.9 (6-C).

Synthesis of di-*t*-butyl [(4-bromopyridine-2,6-diyl)bis(methylene)]biscarbamate S24[70]

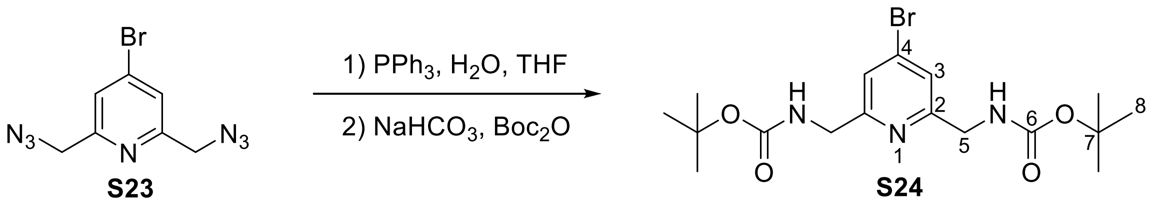

A 50 mL round-bottom flask was charged with 2,6-bis(azidomethyl)-4-bromopyridine **S23** (780 mg, 2.91 mmol). The solid was dissolved in THF (20.3 mL), and triphenylphosphine (1.68 g, 6.40 mmol) was added and the reaction stirred at room temperature. Once the effervescence has ceased, water (5.24 mL, 291 mmol) was added and the reaction was stirred for 16h. Solid sodium bicarbonate (550 mg, 6.55 mmol) was added, followed by Boc_2O (1.67 mL, 7.27 mmol), and the reaction stirred at room temperature overnight. The solvent volume was reduced *in vacuo*, and the remaining solids taken up in ethyl acetate (100 mL) and washed with water (3 × 20 mL) and brine (15 mL). The organics were dried

over Na_2SO_4, filtered and the solvent removed. The colourless solid was purified by flash column chromatography using 60% diethyl ether in hexanes to yield **S24** as a colourless solid (1.19 g, 98%). Mp 153 – 156 °C; R_f 0.15 (60% diethyl ether in hexanes); HRMS (ES) $[M+Na]^+$ $C_{17}H_{26}Br^{79}N_3O_4+Na^+$, calc. 438.1004, obs. 438.0979; v_{max}/cm^{-1} 3359 (NH), 2976 (C-H stretch), 1686 (CO), 1568 (C-C stretch); ^{1}H-NMR (500 MHz, $CDCl_3$) δ 7.33 (2H, s, 3-CH), 5.46 (2H, s, NH), 4.38 (4H, d, J = 5.0 Hz, 5-CH_2), 1.45 (18H, s, 8-CH_3). ^{13}C-NMR (126 MHz, $CDCl_3$) δ 158.8 (2-C), 156.0 (6-C), 134.5 (4-C), 123.5 (3-C), 80.0 (7-C), 45.4 (5-C), 28.5 (8-C).

Synthesis of *t*-butyl {[6-(azidomethyl)-4-bromopyridin-2-yl]methyl}carbamate S25

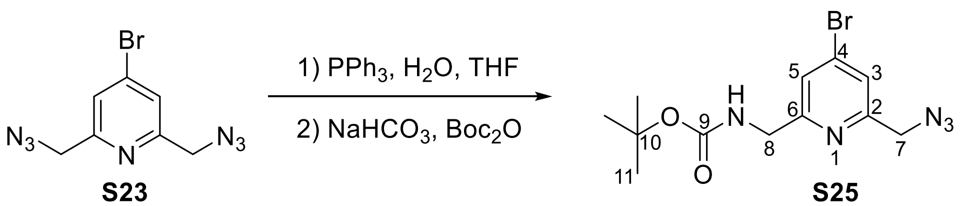

A 10 mL round-bottom flask was charged with 2,6-bis(azidomethyl)-4-bromopyridine **S23** (200 mg, 0.75 mmol). The solid was dissolved in THF (3.5 mL) and water (1.3 mL, 74.6 mmol) was added. With stirring, triphenylphosphine (196 mg, 0.75 mmol) was added in THF (0.5 mL) dropwise, and the reaction was stirred at room temperature for 16h. Solid sodium bicarbonate (500 mg, 5.95 mmol) was added, followed by Boc_2O (240 mL, 1.12 mmol), and the reaction stirred at room temperature overnight. The solvent volume was reduced *in vacuo*, and the remaining solids taken up in ethyl acetate (20 mL) and washed with water (3 × 10 mL) and brine (10 mL). The organics were dried over Na_2SO_4, filtered and the solvent removed. The colourless solid was purified by flash column chromatography using 10% ethyl acetate in hexanes to yield **S25** as a colourless oil (150.3 g, 59%). R_f 0.38 (30% ethyl acetate in hexane); HRMS (ES) $[M+H]^+$ $C_{12}H_{16}Br^{79}N_5O_2+H^+$, calc. 344.0546, obs. 344.0538; v_{max}/cm^{-1} 2976 (C-H stretch), 2103 (N_3), 1700 (CO), 1565 (C-C stretch); ^{1}H-NMR (500 MHz, $CDCl_3$) δ 7.40 (2H, app. s, 3-CH, 5-CH), 5.46 (1H, br. d, J = 5.4 Hz, NH), 4.42 (2H, s, 7-CH_2), 4.40 (2H, d, J = 5.4 Hz, 8-CH_2), 1.45 (9H, s, 11-CH_3); ^{13}C-NMR (126 MHz, $CDCl_3$) δ 159.4 (6-C), 156.8 (2-C), 156.0 (9-C), 134.5 (4-C), 124.1 (5-C), 123.8 (3-C), 80.0 (10-C), 54.9 (7-C), 45.4 (8-C), 28.5 (11-C).

Synthesis of *t*-butyl [(4-bromo-6-{[4-(2-hydroxypropan-2-yl)-1H-1,2,3-triazol-1-yl]methyl}pyridin-2-yl)methyl]carbamate S26

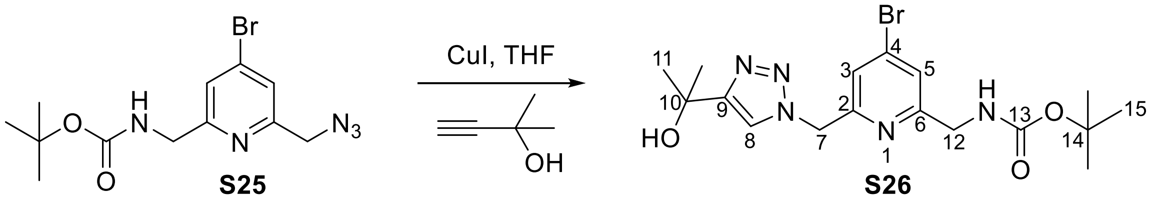

A 5 mL microwave vial was charged with diethyl *t*-butyl {[6-(azidomethyl)-4-bromopyridin-2-yl]methyl}carbamate **S25** (120 mg, 0.29 mmol), copper iodide (5.5 mg, 0.03 mmol), THF (2.9 mL), and methyl-3-butyn-2-ol (31 µL, 0.32 mmol). The reaction was stirred at room temperature for 16h. Upon completion, the solvent volume was reduced *in vacuo*, and the residue taken up in ethyl acetate (15 mL). The organic solution was washed with $EDTA_{(aq)}$ (5 mL), water (3 × 10 mL), and brine (5 mL). The organics were dried over Na_2SO_4, filtered and the solvent removed. The mixture was purified by flash column chromatography using 50% dichloromethane in acetone to yield **S26** as a colourless oil (76.8 mg, 62%). R_f 0.38 (50% dichloromethane in acetone); HRMS (ES) $[M+H]^+$ $C_{17}H_{24}Br^{79}N_5O_3+H^+$, calc. 426.1141, obs. 426.1148; v_{max}/cm^{-1} 3338 (OH), 2975 (C-H stretch), 1692 (CO), 1568 (C-C stretch); ^1H-NMR (500 MHz, $CDCl_3$) δ 7.59 (1H, s, 8-CH), 7.41 (1H, s, 5-CH), 7.23 (1H, s, 3-CH), 5.56 (2H, s, 7-CH_2), 5.44 (1H, br. d, *J* = 5.5 Hz, NH), 4.39 (1H, d, *J* = 5.5 Hz, 12-CH_2), 2.74 (1H, s, OH), 1.63 (6H, s, 11-CH_3), 1.45 (9H, s, 15-CH_3); ^{13}C-NMR (126 MHz, $CDCl_3$) δ 159.7 (6-C), 156.4 (2-C), 156.0 (13-C), 155.1 (9-C), 134.9 (4-C), 124.6 (5-C), 124.3 (3-C), 120.1 (8-C), 80.2 (14-C), 68.6 (10-C), 54.9 (7-C), 45.4 (12-C), 30.5 (11-C), 28.5 (15-C).

Synthesis of 2,2'-[(4-bromopyridine-2,6-diyl)bis(methylene-1H-1,2,3-triazole-1,4-diyl)]di(propan-2-ol) S27

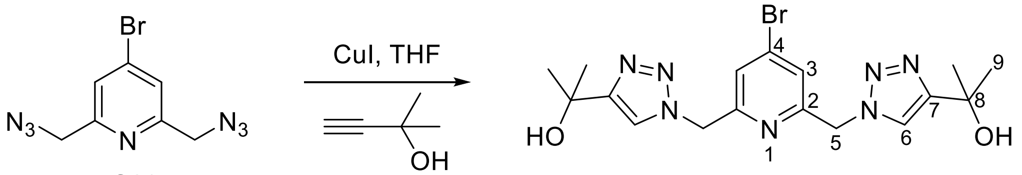

S23 **S27**

A 5 mL microwave vial was charged with 2,6-bis(azidomethyl)-4-bromopyridine **S23** (100 mg, 0.37 mmol), copper iodide (14.2 mg, 0.07 mmol), THF (1.9 mL), and methyl-3-butyn-2-ol (110 µL, 1.12 mmol). The reaction was stirred at room temperature for 16h. Upon completion, the solvent volume was reduced *in vacuo*, and the residue taken up in ethyl acetate (15 mL). The organic solution was washed with $EDTA_{(aq)}$ (5 mL), water (3 × 10 mL), and brine (5 mL). The organics were dried over Na_2SO_4, filtered and the solvent removed. The mixture was purified by flash column chromatography using 50% dichloromethane in acetone to yield **S27** as a colourless oil (148.1 mg, 92%). R_f 0.28 (50% dichloromethane in acetone); HRMS (ES) $[M+H]^+$ $C_{17}H_{22}Br^{79}N_7O_2+H^+$, calc. 437.1097, obs. 437.1121; ν_{max}/cm^{-1} 3380 (OH), 2977 (C-H stretch), 1571 (C-C stretch); ^1H-NMR (500 MHz, $CDCl_3$) δ 7.55 (2H, s, 3-CH), 7.38 (2H, s, 6-CH), 5.51 (4H, s, 5-CH_2), 3.55 (2H, s, OH), 1.59 (12H, s, 9-CH_3); ^{13}C-NMR (126 MHz, $CDCl_3$) δ 156.4 (2-C), 155.7 (7-C), 135.0 (4-C), 125.4 (3-C), 120.6 (6-C), 68.6 (8-C), 54.4 (5-C), 30.4 (9-C).

Synthesis of 4-iodopyridine *N*-oxide S29

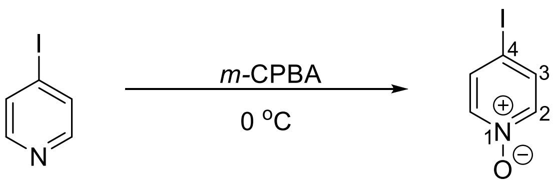

S28 **S29**

A 10 mL round-bottom flask was charged with 4-iodopyridine **S28** (100 mg, 0.49 mmol) and dichloromethane (5 mL). The flask was cooled to 0 °C in an ice bath and *m*-CPBA (210 mg, 0.61 mmol) was added. The reaction was left to stir overnight. Upon completion, the pale-yellow solution was diluted with dichloromethane (10 mL) and treated with 6 M potassium hydroxide solution (5 mL). The layers were separated, and the aqueous layer extracted with

dichloromethane (3 × 5 mL). The organics were then combined and dried over $MgSO_4$. The solvent was removed *in vacuo* to yield **S29** as a pale orange solid (106 mg, 98%). Mp 160 – 162 °C; R_f 0.48 (25% methanol in ethyl acetate); HRMS (ES) $[M+H]^+$ $C_5H_5INO+H^+$, calc. 221.9416, obs. 221.9183; v_{max}/cm^{-1} 3071, 2990 (C-H stretch) 863 (C-H bend); ^{1}H-NMR (500 MHz, $CDCl_3$) δ 7.93 (2H, d, J = 7.0 Hz, 2-CH), 7.57 (2H, d, J = 7.0 Hz, 3-CH); ^{13}C-NMR (126 MHz, $CDCl_3$) δ 140.4 (2-C), 135.3 (3-C), 88.6 (4-C).

Synthesis of 2-(pyrrolidin-1-yl)-4-iodopyridine S30

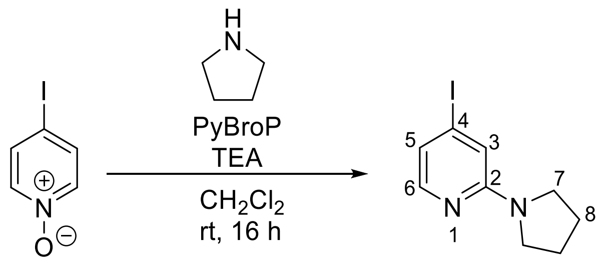

To a 30 mL microwave vial was added 4-iodopyridine *N*-oxide **S29** (200 mg, 0.90 mmol), pyrrolidine (100 μL, 1.20 mmol), and PyBroP (550 mg, 1.18 mmol). Triethylamine (5.2 mL) was added, and the mixture stirred. Dichloromethane (5.2 mL) was added until the contents of the vial dissolved. The reaction was stirred at room temperature overnight. Upon completion, the reaction mixture was poured into a saturated sodium bicarbonate solution (30 mL), and the organic layer washed with brine (3 × 15 mL). The combined aqueous layers were extracted with dichloromethane (3 × 30 mL) and the organics combined. The combined organics were dried over Na_2SO_4, filtered, and the solvent removed *in vacuo* and purified by flash column chromatography using 20% ethyl acetate in petroleum ether (40-60) to yield **S30** as a colourless solid (179.4 mg, 73%). Mp 56 – 60 °C; R_f 0.42 (20% ethyl acetate in petroleum ether (40-60)); HRMS (ES) $[M+H]^+$ $C_9H_{11}IN_2+H^+$, calc. 275.0045, obs. 275.0039; v_{max}/cm^{-1} 2971 and 2854 (C-H stretch); ^{1}H-NMR (500 MHz, $CDCl_3$) δ 7.79 (1H, d, J = 5.3 Hz, 6-CH), 6.84 (1H, dd, J = 5.3, 1.2 Hz, 5-CH), 6.75 (1H, d, J = 1.2 Hz, 3-CH), 3.42 (4H, d, J = 6.6 Hz, 7-CH_2), 2.03 – 1.96 (4H, m, 8-CH_2); ^{13}C-NMR (126 MHz, $CDCl_3$) δ 157.3 (2-C), 148.4 (6-C), 120.0 (5-C), 115.6 (3-C), 106.3 (4-C), 46.9 (7-C), 25.6 (8-C).

Synthesis of methyl (*S*)-2-((*t*-butoxycarbonyl)amino)-3-(4-((4-iodopyridin-2-yl)oxy)phenyl)propanoate S31

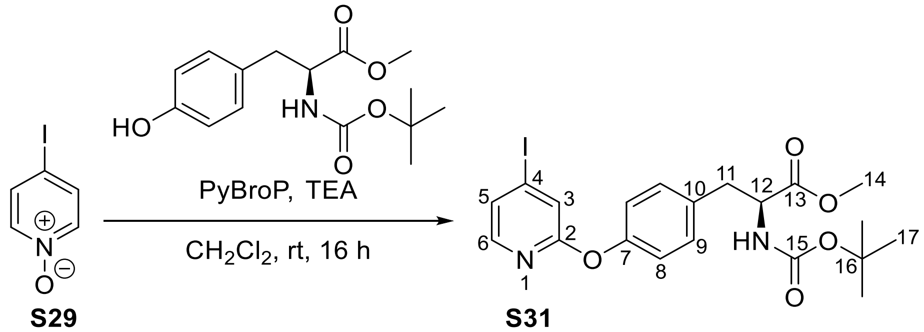

To a 30 mL microwave vial was added 4-iodopyridine *N*-oxide **S29** (210 mg, 0.95 mmol), methyl (*t*-butoxycarbonyl)-L-tyrosinate (350 mg, 1.19 mmol), and PyBroP (580 mg, 1.24 mmol). The solids were dissolved in dichloromethane (5.3 mL), and triethylamine (5.3 mL) was added. The reaction was stirred at room temperature overnight. Upon completion, the reaction mixture was poured into a sodium hydroxide solution (2 M, 30 mL), extracted with dichloromethane (3 × 10 mL), and the organics combined and dried over $MgSO_4$, filtered, and the solvent removed *in vacuo.* The crude mixture was purified by flash column chromatography using 10% ethyl acetate in hexanes to yield **S31** as a colourless oil (434.1 mg, 92%). R_f 0.09 (10% ethyl acetate in hexanes); HRMS (ES) $[M+H]^+$ $C_{20}H_{23}IN_2O_5+H^+$, calc. 499.0730, obs. 499.0731; v_{max}/cm^{-1} 2978 (C-H stretch), 1710 (C=O stretch), 1563 (N-H stretch), 1504, 1378 , 1216, 1164 (C-O stretch), 735 (C-I stretch); ^{1}H-NMR (500 MHz, $CDCl_3$) δ 7.84 (1H, d, *J* = 5.3 Hz, 6-CH), 7.33 (1H, dd, *J* = 5.3, 1.0 Hz, 5-CH), 7.31 (1H, br. s, 3-CH), 7.16 (2H, d, *J* = 8.4 Hz, 9-CH), 7.04 (2H, d, *J* = 8.4 Hz, 8-CH), 5.02 (1H, br. d, *J* = 8.0 Hz, NH), 4.59 (1H, app. q, *J* = 6.3 Hz, 12-CH), 3.72 (3H, s, 14-CH_3), 3.12 (1H, dd, *J* = 13.9, 6.3 Hz, 11-CH), 3.03 (1H, dd, *J* = 13.9, 6.3 Hz, 11-CH), 1.42 (9H, s, 17-CH_3); ^{13}C-NMR (126 MHz, $CDCl_3$) δ 172.4 (13-C), 163.7 (2-C), 155.2 (15-C), 152.7 (7-C), 147.9 (6-C), 133.0 (10-C), 130.8 (9-C), 127.7 (5-C), 121.4 (3-C), 120.9 (8-C), 107.3 (4-C), 80.1 (16-C), 54.5 (12-C), 52.4 (14-C), 37.9 (11-C), 28.4 (17-C).

General vinylation methodology:

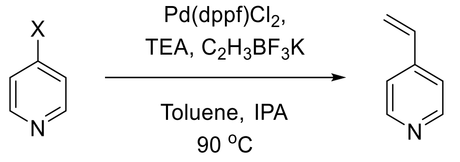

In an oven dried reaction vessel, the 4-haloaromatic species (1 equivalent) was dissolved in toluene (0.2 M). To this solution, was added potassium vinyltrifluoroborate (2 eq.), [1,1′-bis(diphenylphosphino)ferrocene] dichloropalladium$^{(II)}$ (5 mol%), triethylamine (3 eq.), and isopropyl alcohol (0.1 M). The solution was degassed at room temperature and then heated to 90 °C. Upon completion, the reaction mixture was loaded onto silica and purified by flash column chromatography.

Ethyl 3-(4-ethenyl-6-methylpyridin-2-yl)propanoate A from S6

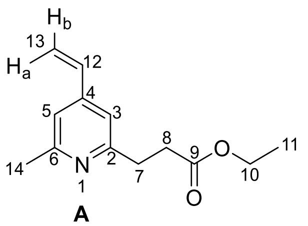

A

Upon completion, the reaction mixture was loaded onto silica and purified by flash column chromatography using 20% ethyl acetate in hexanes to yield **A** as a yellow oil (263.2 mg, 93%). R_f 0.16 (20% ethyl acetate in hexanes); HRMS (ES) $[M+H]^+$ $C_{13}H_{17}NO_2+H^+$, calc. 220.1332, obs. 220.1329; v_{max}/cm^{-1} 2978 (C-H stretch), 1730 (CO), 1602, 1558, 1158; ^{1}H-NMR (500 MHz, $CDCl_3$) δ 6.95 (1H, s, 5-CH), 6.94 (1H, s, 3-CH), 6.57 (1H, dd, *J* = 17.6, 10.8 Hz, 12-CH), 5.89 (1H, dd, *J* = 17.6, 0.6 Hz, 13-CH_a), 5.39 (1H, dd, *J* = 10.8, 0.6 Hz, 13-CH_b), 4.10 (2H, q, *J* = 7.1 Hz, 10-CH_2), 3.04 (2H, t, *J* = 7.6 Hz, 7-CH_2), 2.74 (2H, t, *J* = 7.6 Hz, 8-CH_2), 2.48 (3H, s, 14-CH_3), 1.20 (3H, t, *J* = 7.1 Hz, 11-CH_3); ^{13}C-NMR (126 MHz, $CDCl_3$) δ 173.1 (9-C), 159.9 (6-C), 158.3 (2-C), 145.5 (4-C), 135.2 (12-C), 118.2 (13-C), 118.2 (3-C), 117.1 (5-C), 60.4 (10-C), 34.0 (8-C), 33.1 (7-C), 24.4 (14-C), 14.3 (11-C).

Diethyl 2-[(4-ethenyl-6-methylpyridin-2-yl)methyl]butanedioate B from S7

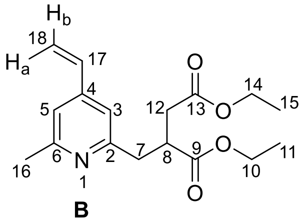

B

Upon completion, the reaction mixture was loaded onto silica and purified by flash column chromatography using 40% ethyl acetate in hexanes to yield **B** as a colourless oil (82.5 mg, 96%). R_f 0.35 (40% ethyl acetate in hexanes); HRMS (ES) $[M+H]^+$ $C_{17}H_{23}NO_4+H^+$, calc. 306.1700, obs. 306.1697; v_{max}/cm^{-1} 2979 (C-H stretch), 1728, 1601 (C=C stretch), 1557, 1373, 1174 (C-O stretch); ^{1}H-NMR (500 MHz, $CDCl_3$) δ 6.96 (1H, s, 5-CH), 6.91 (1H, s, 3-CH), 6.57 (1H, dd, *J* = 17.6, 10.9 Hz, 17-CH), 5.90 (1H, dd, *J* = 17.6, 0.5 Hz, 18-CH_a), 5.41 (1H, dd, *J* = 10.9, 0.5 Hz, 18-CH_b), 4.13 – 4.05 (4H, m, 10-CH_2, 14-CH_2), 3.38 – 3.30 (1H, m, 8-CH), 3.14 (1H, dd, *J* = 13.8, 6.7 Hz, 7-CH), 2.91 (1H, dd, *J* = 13.8, 7.9 Hz, 7-CH), 2.69 (1H, dd, *J* = 16.7, 9.2 Hz, 12-CH), 2.49 (3H, s, 16-CH_3), 2.50 – 2.45 (1H, m, 12-CH), 1.20 (3H, t, *J* = 7.1 Hz, 11-CH_3), 1.17 (3H, t, *J* = 7.1 Hz, 15-CH_3); ^{13}C-NMR (126 MHz, $CDCl_3$) δ 174.4 (9-C), 172.0 (13-C), 158.5 (6-C), 158.2 (2-C), 145.6 (4-C), 135.1 (17-C), 118.5 (5-C), 118.3 (18-C), 117.8 (3-C), 60.7 (10-C), 60.6 (14-C), 41.6 (8-C), 39.7 (7-C), 35.5 (12-C), 24.5 (16-C), 14.2 (11-C), 14.2 (15-C).

Diethyl 2,2'-(((6-methyl-4-vinylpyridin-2-yl)methyl)azanediyl)diacetate C from S8

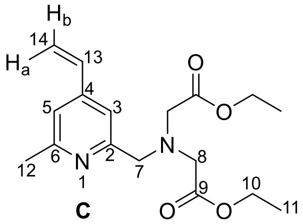

C

Upon completion, the reaction mixture was loaded onto silica and purified by flash column chromatography using 70% diethyl ether in hexanes to yield **C** as a yellow oil (60 mg, 90%). R_f 0.19 (70% diethyl ether in hexanes); HRMS (ES) $[M+H]^+$ $C_{17}H_{24}N_2O_4+H^+$, calc. 321.1814, obs. 321.1829; v_{max}/cm^{-1} 2979 (C-H stretch), 1737, 1601 (C=C stretch), 1557, 1372, 1188, 1150 (C-O stretch); ^{1}H-NMR (500 MHz, $CDCl_3$) δ 7.49 (1H, s, 5-CH), 7.03 (1H, s, 3-CH), 6.61 (1H, dd, *J* = 17.6, 10.9 Hz, 13-CH), 5.97 (1H, d, *J* = 17.6 Hz, 14-CH_a), 5.46 (1H, d, *J* = 10.9 Hz, 14-CH_b), 4.11 (4H, q, *J* = 7.1 Hz, 10-CH_2), 4.07 (2H, s, 7-CH_2), 3.57 (4H, s, 8-CH_2),

2.54 (3H, s, 12-CH_3), 1.21 (6H, t, *J* = 7.1 Hz, 11-CH_3); ^{13}C-NMR (126 MHz, $CDCl_3$) δ 171.1 (9-C), 158.2 (2-C), 157.4 (6-C), 147.0 (4-C) 134.8 (13-C), 119.6 (14-C), 119.5 (3-C), 117.6 (5-C), 60.6 (10-C), 59.2 (7-C), 55.1 (8-C), 23.5 (12-C), 14.3 (11-C).

Diethyl 2,2'-((2-(6-methyl-4-vinylpyridin-2-yl)ethyl)azanediyl)diacetate D from S13

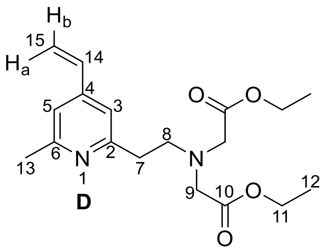

D

Upon completion, the reaction mixture was loaded onto silica and purified by flash column chromatography using 50% ethyl acetate in hexane to yield **D** as a yellow oil (172.2 mg, 78%). R_f 0.19 (50% ethyl acetate in hexanes); HRMS (ES) $[M+H]^+$ $C_{18}H_{26}N_2O_4+H^+$, calc. 335.1971, obs. 335.1974; v_{max}/cm^{-1} 2980 (C-H stretch), 1735, 1602 (C=C stretch), 1558, 1183, 1139, 1027 (C-O stretch); ^{1}H-NMR (500 MHz, $CDCl_3$) δ 7.01 (1H, s, 5-CH), 6.95 (1H, s, 3-CH), 6.59 (1H, dd, *J* = 17.6, 10.9 Hz, 14-CH), 5.92 (1H, dd, *J* = 17.6, 0.7 Hz, 15-CH_a), 5.41 (1H, dd, *J* = 10.9, 0.7 Hz, 15-CH_b), 4.15 (4H, q, *J* = 7.1 Hz, 11-CH_2), 3.58 (4H, s, 9-CH_2), 3.12 (2H, dd, *J* = 8.9, 6.5 Hz, 8-CH_2), 2.94 (2H, dd, *J* = 8.9, 6.5 Hz, 7-CH_2), 2.49 (3H, s, 13-CH_3), 1.25 (6H, t, *J* = 7.1 Hz, 12-CH_3); ^{13}C-NMR (126 MHz, $CDCl_3$) δ 171.5 (10-C), 159.7 (6-C), 158.2 (2-C), 145.5 (4-C), 135.3 (14-C), 118.1 (3-C, 15-C), 117.6 (5-C), 60.6 (11-C), 55.3 (9-C), 54.8 (8-C), 37.3 (7-C), 24.5 (13-C), 14.4 (12-C).

Diethyl 2,2'-((3-(6-methyl-4-vinylpyridin-2-yl)propyl)azanediyl)diacetate E from S16

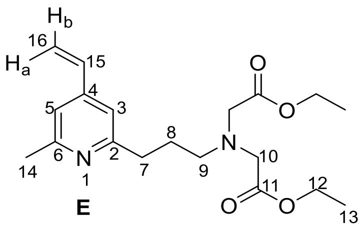

E

Upon completion, the reaction mixture was loaded onto silica and purified by flash column chromatography using 50% ethyl acetate in hexanes to yield **E** as a yellow oil (171 mg, 97%). R_f 0.23 (50% ethyl acetate in hexanes); HRMS (ES) $[M+H]^+$ $C_{19}H_{28}N_2O_4+H^+$, calc.

349.2127, obs. 349.2143; v_{max}/cm^{-1} 2933 (C-H stretch), 1735, 1601 (C=C stretch), 1558, 1372, 1182, 1137, 1028 (C-O stretch); ^{1}H-NMR (500 MHz, $CDCl_3$) δ 6.96 (1H, s, 5-CH), 6.95 (1H, s, 3-CH), 6.60 (1H, dd, *J* = 17.6, 10.9 Hz, 15-CH), 5.92 (1H, dd, *J* = 17.6, 0.7 Hz, 16-CH_a), 5.42 (1H, dd, *J* = 10.9, 0.7 Hz, 16-CH_b), 4.15 (4H, q, *J* = 7.1 Hz, 12-CH_2), 3.55 (4H, s, 10-CH_2), 2.81 – 2.73 (4H, m, 7-CH_2, 9-CH_2), 2.51 (3H, s, 14-CH_3), 1.95 – 1.86 (2H, m, 8-CH_2), 1.25 (6H, t, *J* = 7.1 Hz, 13-CH_3); ^{13}C-NMR (126 MHz, $CDCl_3$) δ 171.5 (11-C), 161.6 (2-C), 158.1 (6-C), 145.6 (4-C), 135.3 (15-C), 118.2 (16-C), 118.0 (3-C), 117.2 (5-C), 60.6 (12-C), 55.2 (10-C), 54.0 (9-C), 35.7 (7-C), 28.3 (8-C), 24.4 (14-C), 14.4 (13-C).

Diethyl 3,3'-(4-ethenylpyridine-2,6-diyl)dipropanoate F from S18

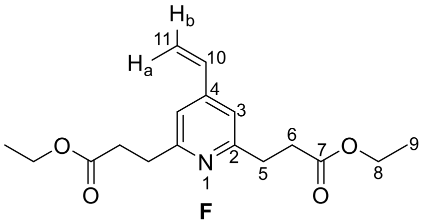

F

Upon completion, the reaction mixture was loaded onto silica and purified by flash column chromatography using 5% acetone in dichloromethane to yield **F** as a colourless oil (84.8 mg, 99%). R_f 0.16 (30% ethyl acetate in hexanes); HRMS (ES) $[M+H]^+$ $C_{17}H_{23}NO_4+H^+$, calc. 306.1705, obs. 306.1709; v_{max}/cm^{-1} 2979 (C-H stretch), 1729, 1602 (C=C stretch), 1555, 1158 (C-O stretch); ^{1}H-NMR (500 MHz, $CDCl_3$) δ 7.00 (2H, s, 3-CH), 6.60 (1H, dd, *J* = 17.6, 10.9 Hz, 10-CH), 5.92 (1H, dd, *J* = 17.6, 0.6 Hz, 11-CH_a), 5.44 (1H, dd, *J* = 10.9, 0.6 Hz, 11-CH_b), 4.13 (4H, q, *J* = 7.1 Hz, 8-CH_2), 3.08 (4H, t, *J* = 7.5 Hz, 5-CH_2), 2.78 (4H, t, *J* = 7.5 Hz, 6-CH_2), 1.23 (6H, t, *J* = 7.1 Hz, 9-CH_3); ^{13}C-NMR (126 MHz, $CDCl_3$) δ 173.4 (7-C), 159.9 (2-C), 145.6 (4-C), 135.2 (10-C), 118.2 (11-C), 117.8 (3-C), 60.4 (8-C), 33.5 (6-C), 32.9 (5-C), 14.3 (9-C).

Ethyl 3-(4-ethenyl-6-{[4-(2-hydroxypropan-2-yl)-1H-1,2,3-triazol-1-yl]methyl}pyridin-2-yl)propanoate G from S21

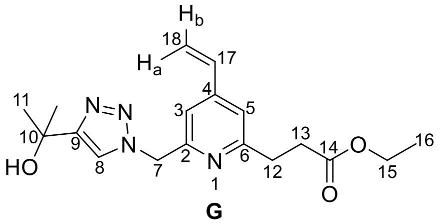

G

Upon completion, the reaction mixture was loaded onto silica and purified by flash column chromatography using 30% acetone in dichloromethane to yield **G** as a colourless solid (84.7 mg, 98%). R_f 0.44 (30% acetone in dichloromethane); HRMS (ES) $[M+H]^+$ $C_{18}H_{24}N_4O_3+H^+$, calc. 345.1921, obs. 345.1920; ν_{max}/cm^{-1} 3379 (OH), 2978 (C-H stretch), 1725, 1606 (C=C stretch), 1561, 1374, 1171 (C-O stretch); ^{1}H-NMR (500 MHz, $CDCl_3$) δ 7.64 (1H, s, 8-CH), 7.10 (1H, s, 3-CH), 7.02 (1H, s, 5-CH), 6.57 (1H, dd, *J* = 17.6, 10.9 Hz, 17-CH), 5.90 (1H, d, *J* = 17.6 Hz, 18-CH_a), 5.54 (2H, s, 7-CH_2), 5.46 (1H, d, *J* = 10.9 Hz, 18-CH_b), 4.08 (2H, q, *J* = 7.1 Hz, 15-CH_2), 3.09 (2H, t, *J* = 7.3 Hz, 12-CH_2), 2.77 (3H, t, *J* = 7.3 Hz, 13-CH_2), 1.62 (6H, s, 11-CH_3), 1.24 (1H, s, OH), 1.18 (3H, t, *J* = 7.1 Hz, 16-CH_3); ^{13}C-NMR (126 MHz, CDCl3) δ 173.1 (14-C), 160.7 (6-C), 156.1 (2-C), 154.1 (9-C), 146.7 (4-C), 134.4 (17-C), 120.1 (8-C), 120.0 (3-C), 119.5 (18-C), 117.4 (5-C), 68.5 (10-C), 60.6 (15-C), 55.5 (7-C), 33.1 (13-C), 32.5 (12-C), 30.5 (11-C), 14.3 (16-C).

Ethyl 3-(6-{[(*t*-butoxycarbonyl)amino]methyl}-4-ethenylpyridin-2-yl)propanoate H from S22

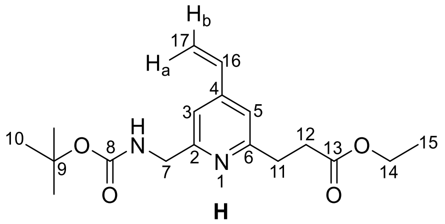

H

Upon completion, the reaction mixture was loaded onto silica and purified by flash column chromatography using 30% ethyl acetate in hexanes to yield **H** a colourless oil (447.2 mg, 96%). R_f 0.29 (30% ethyl acetate in hexanes); HRMS (ES) $[M+H]^+$ $C_{18}H_{26}N_2O_4+H^+$, calc. 335.1971, obs. 335.1996; ν_{max}/cm^{-1} 2976 (C-H stretch), 1711, 1605 (C=C stretch), 1501, 1366, 1247, 1164 (C-O stretch); ^{1}H-NMR (400 MHz, $CDCl_3$) δ 7.02 (1H, s, 5-CH), 7.01 (1H, s, 3-CH), 6.58 (1H, dd, *J* = 17.6, 10.9 Hz, 16-CH), 5.89 (1H, dd, *J* = 17.6, 0.5 Hz, 17-CH_a),

5.64 (1H, s, NH), 5.41 (1H, dd, J = 10.9, 0.5 Hz, 17-CH_b), 4.35 (2H, d, J = 5.1 Hz, 7-CH_2), 4.10 (2H, q, J = 7.1 Hz, 14-CH_2), 3.05 (2H, t, J = 7.4 Hz, 11-CH_2), 2.73 (2H, t, J = 7.4 Hz, 12-CH_2), 1.43 (9H, s, 10-CH_3), 1.20 (3H, t, J = 7.1 Hz, 15-CH_3); ^{13}C-NMR (101 MHz, $CDCl_3$) δ 173.2 (13-C), 159.9 (6-C), 157.1 (2-C), 156.1 (8-C), 145.9 (4-C), 134.9 (16-C), 118.7 (17-C), 118.6 (5-C), 116.4 (3-C), 79.4 (9-C), 60.4 (14-C), 45.6 (7-C), 33.4 (12-C), 32.7(11-C), 28.5 (10-C), 14.3 (15-C).

Di-*t*-butyl [(4-ethenylpyridine-2,6-diyl)bis(methylene)]biscarbamate I from S24

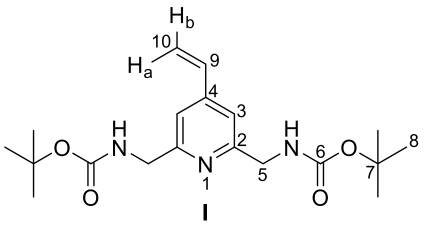

I

Upon completion, the reaction mixture was loaded onto silica and purified by flash column chromatography using 5% acetone in dichloromethane to yield a colourless solid (86.2 mg, 99%). R_f 0.12 (60% diethyl ether in hexanes); Mp 92 – 96 °C; HRMS (ES) $[M+H]^+$ $C_{19}H_{29}N_3O_4+H^+$, calc. 364.2236, obs. 364.2246; ν_{max}/cm^{-1} 3342 (NH), 2973 (C-H stretch), 1684 (C=C stretch), 1521 (N-H bend), 1157 (CO); ^{1}H-NMR (500 MHz, CDCl3) δ 7.13 (2H, s, 3-CH), 6.63 (1H, dd, J = 17.6, 10.9 Hz, 9-CH), 5.95 (1H, d, J = 17.6 Hz, 10-CH_a), 5.52 (2H, s, NH), 5.48 (1H, d, J = 10.9 Hz, 10-CH_b), 4.41 (4H, d, J = 5.3 Hz, 5-CH_2), 1.46 (18H, s, 8-CH_3); ^{13}C-NMR (101 MHz, $CDCl_3$) δ 157.5 (2-C), 156.1 (6-C), 146.5 (4-C), 134.7 (9-C), 119.2 (10-C), 117.4 (3-C), 79.6 (7-C), 45.7 (5-C), 28.5 (8-C).

***t*-Butyl [(4-ethenyl-6-{[4-(2-hydroxypropan-2-yl)-1H-1,2,3-triazol-1-yl]methyl}pyridin-2-yl)methyl]carbamate J from S26**

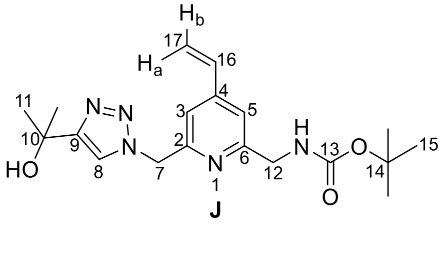

J

Upon completion, the reaction mixture was loaded onto silica and purified by flash column chromatography using 25% acetone in dichloromethane to yield **J** as a colourless oil (41.1 mg, 98%). R_f 0.12 (25% acetone in dichloromethane); HRMS (ES) $[M+H]^+$ $C_{19}H_{27}N_5O_3+H^+$, calc. 374.2192, obs. 374.0785; ν_{max}/cm^{-1} 3342 (OH), 2976 (C-H stretch), 1692, 1606 (C=C stretch), 1365 (C-H bend), 1164; ^{1}H-NMR (500 MHz, CDCl3) δ 7.59 (1H, s, 8-CH), 7.18 (1H, s, 5-CH), 7.04 (1H, s, 3-CH), 6.58 (1H, dd, *J* = 17.6, 10.9 Hz, 16-CH), 5.91 (1H, d, *J* = 17.6 Hz, 17-CH_a), 5.57 (2H, s, 7-CH_2), 5.53 (1H, s, NH), 5.48 (1H, d, *J* = 10.9 Hz, 17-CH_b), 4.40 (2H, d, *J* = 4.8 Hz, 12-CH_2), 2.92 (1H, s, OH), 1.62 (6H, s, 11-CH_3), 1.45 (9H, s, 15-CH_3); ^{13}C-NMR (126 MHz, $CDCl_3$) δ 158.3 (6-C), 156.2 (13-C), 156.1 (2-C), 154.3 (9-C), 147.2 (4-C), 134.3 (16-C), 119.9 (8-C, 17-C), 118.5 (5-C), 118.2 (3-C), 79.9 (14-C), 68.6 (10-C), 55.5 (7-C), 45.6 (12-C), 30.5 (11-C), 28.5 (15-C).

2,2'-[(4-Ethenylpyridine-2,6-diyl)bis(methylene-1H-1,2,3-triazole-1,4-diyl)]di(propan-2-ol) K from S27

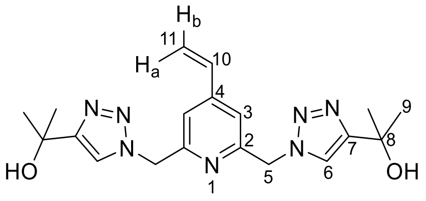

K

Upon completion, the reaction mixture was loaded onto silica and purified by flash column chromatography using 50% acetone in dichloromethane to yield a colourless oil (88.9 mg, 92%). R_f 0.21 (50% acetone in dichloromethane); HRMS (ES) $[M+H]^+$ $C_{19}H_{29}N_3O_4+H^+$, calc. 364.2236, obs. 364.2246; ν_{max}/cm^{-1} 3351 (OH), 2974 (C-H stretch), 1604 (C=C stretch), 1368 (C-H bend), 1209, 1177, 1138, 1055; ^{1}H-NMR (500 MHz, $CDCl_3$) δ 7.55 (2H, s, 6-CH), 7.18 (2H, s, 3-CH), 6.57 (1H, dd, *J* = 17.6, 10.9 Hz, 10-CH), 5.92 (1H, d, *J* = 17.6 Hz, 11-CH_a), 5.52 (4H, s, 5-CH_2), 5.50 (1H, d, *J* = 10.9 Hz, 11-CH_b), 2.14 (2H, s, OH), 1.59 (12H, s, 9-CH_3); ^{13}C-NMR (126 MHz, CDCl3) δ 156.3 (2-C), 154.9 (7-C), 147.7 (4-C), 133.8 (10-C), 120.6 (11-C), 120.5 (6-C), 119.3 (3-C), 68.7 (8-C), 55.0 (5-C), 30.5 (9-C).

2-(Pyrrolidin-1-yl)-4-vinylpyridine L from S30

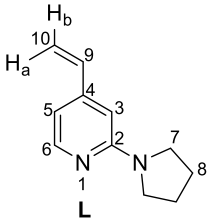

L

Upon completion, the reaction mixture was loaded onto silica and purified by flash column chromatography using 20% ethyl acetate in hexanes to yield **L** as a yellow oil (83%, 53 mg). R_f 0.21 (20% ethyl acetate in petroleum ether (40-60)); HRMS (ES) $[M+H]^+$ $C_{11}H_{14}N+H^+$, calc. 175.1235, obs. 175.1231; ν_{max}/cm^{-1} 3012, 3088 (C-H stretch), 2966, 2856 (C-H stretch), 1598 (C=C stretch); ^{1}H-NMR (500 MHz, $CDCl_3$) δ 8.09 (1H, d, *J* = 5.3 Hz, 6-CH), 6.62 – 6.55 (2H, m, 5-CH, 9-CH), 6.27 (1H, s, 3-CH), 5.86 (1H, dd, *J* = 17.6, 0.8 Hz, 10-CH_a), 5.36 (1H, dd, *J* = 10.8, 0.8 Hz, 10-CH_b), 3.45 (4H, t, *J* = 6.7 Hz, 7-CH_2), 2.01 – 1.97 (4H, m, 8-CH_2); ^{13}C-NMR (126 MHz, $CDCl_3$) δ 158.0 (2-C), 148.4 (6-C), 145.8 (4-C), 136.1 (9-C), 117.2 (10-C), 108.2 (5-C), 104.5 (3-C), 46.2 (7-C), 25.6 (8-C).

Methyl (*S*)-2-((*t*-butoxycarbonyl)amino)-3-(4-((4-vinylpyridin-2-yl)oxy)phenyl)propanoate M from S31

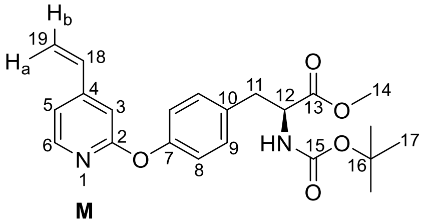

M

Upon completion, the reaction mixture was loaded onto silica and purified by flash column chromatography using 30% ethyl acetate in hexanes to yield **M** as a colourless oil (87.7 mg, 73%). R_f 0.26 (30% ethyl acetate in hexanes); HRMS (ES) $[M+H]^+$ $C_{22}H_{26}N_2O_5+H^+$, calc. 399.1920, obs. 399.1915; ν_{max}/cm^{-1} 2972 (C-H stretch), 1709 (C=O stretch), 1600, 1547 (N-H stretch), 1505, 1385, 1365, 1212, 1164 (C-O stretch); ^{1}H-NMR (500 MHz, $CDCl_3$) δ 8.12 (1H, d, *J* = 5.2 Hz, 6-CH), 7.15 (2H, d, *J* = 8.4 Hz, 9-CH), 7.06 (2H, d, *J* = 8.4 Hz, 8-CH), 7.01 (1H, d, *J* = 5.2 Hz, 5-CH), 6.84 (1H, s, 3-CH), 6.64 (1H, dd, *J* = 17.6, 10.8 Hz, 18-CH), 5.94 (1H, d, *J* = 17.6 Hz, 19-CH_a), 5.48 (1H, d, *J* = 10.8 Hz, 19-CH_b), 5.03 (1H, d, *J* = 8.3 Hz, NH), 4.58 (1H, q, *J* = 6.3 Hz, 12-CH), 3.72 (3H, s, 14-CH_3), 3.12 (1H, dd, *J* = 14.0, 6.1

Hz, 1H), 3.04 (dd, *J* = 14.0, 6.1 Hz, 1H), 1.42 (9H, s, 17-CH_3); ^{13}C-NMR (126 MHz, $CDCl_3$) δ 172.4 (13-C), 164.4 (2-C), 155.2 (15-C), 153.4 (7-C), 148.8 (4-C), 147.9 (6-C), 134.6 (18-C), 132.4 (10-C), 130.7 (9-C), 121.2 (8-C), 119.2 (19-C), 116.0 (5-C), 108.8 (3-C), 80.1 (16-C), 54.5 (12-C), 52.4 (14-C), 37.8 (11-C), 28.4 (17-C).

4-Bromo-6-methyl-2-pyridinemethanol S2

^{1}H-NMR (500 MHz, $CDCl_3$).

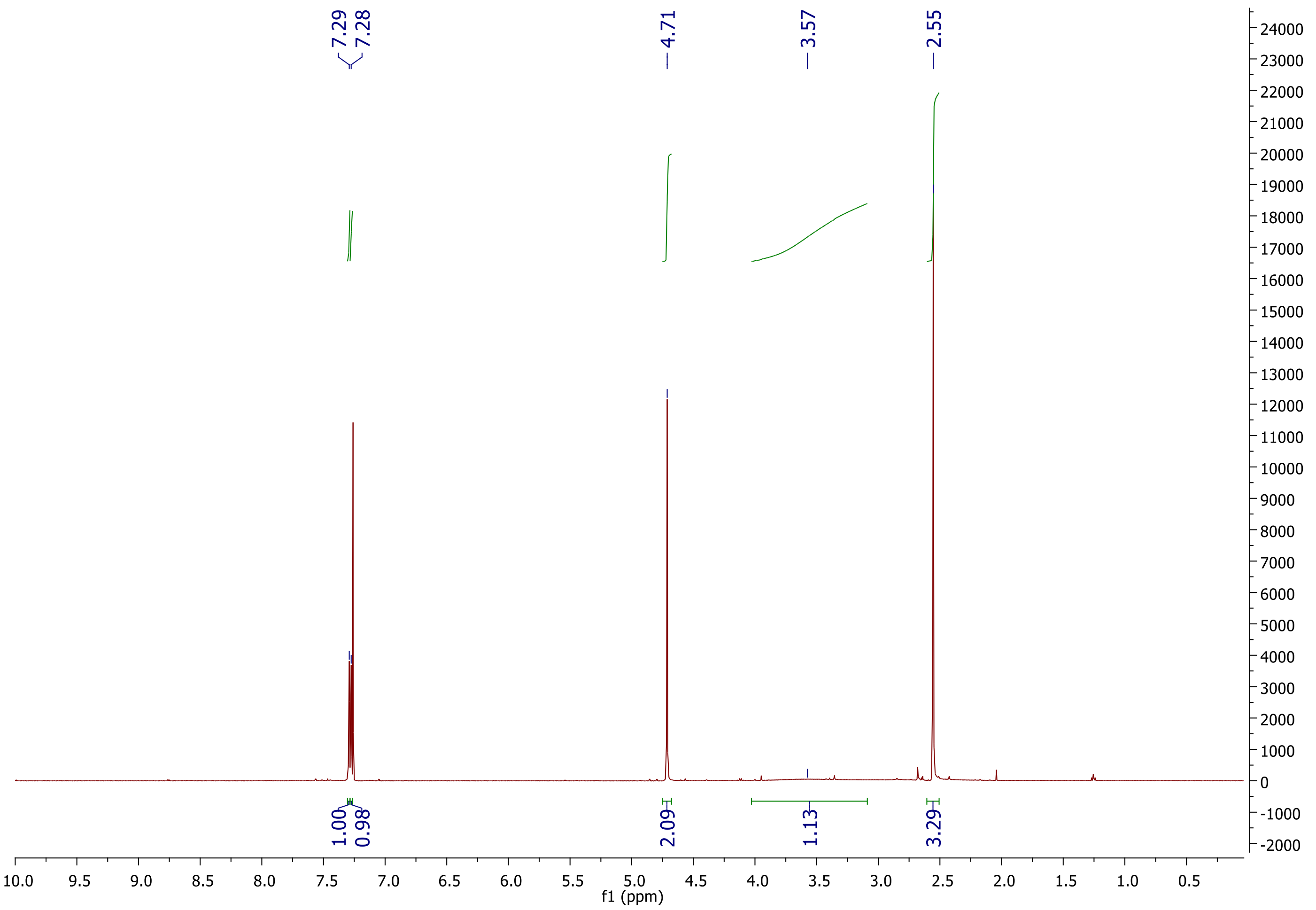

4-Bromo-6-methyl-2-pyridinemethanol S2

^{13}C-NMR (126 MHz, $CDCl_3$)

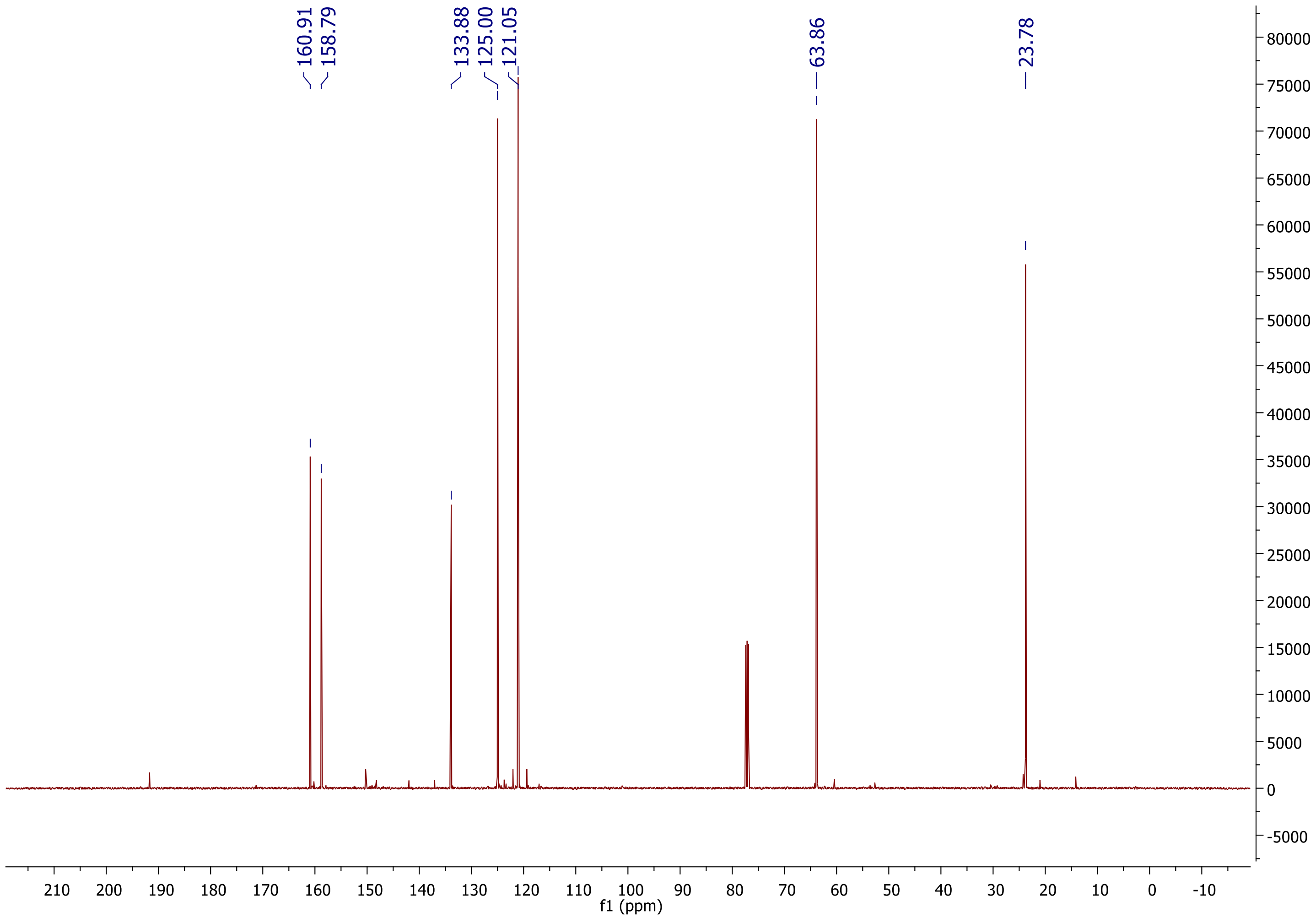

4-Bromo-2-(bromomethyl)-6-methylpyridine S3

^{1}H-NMR (500 MHz, $CDCl_3$).

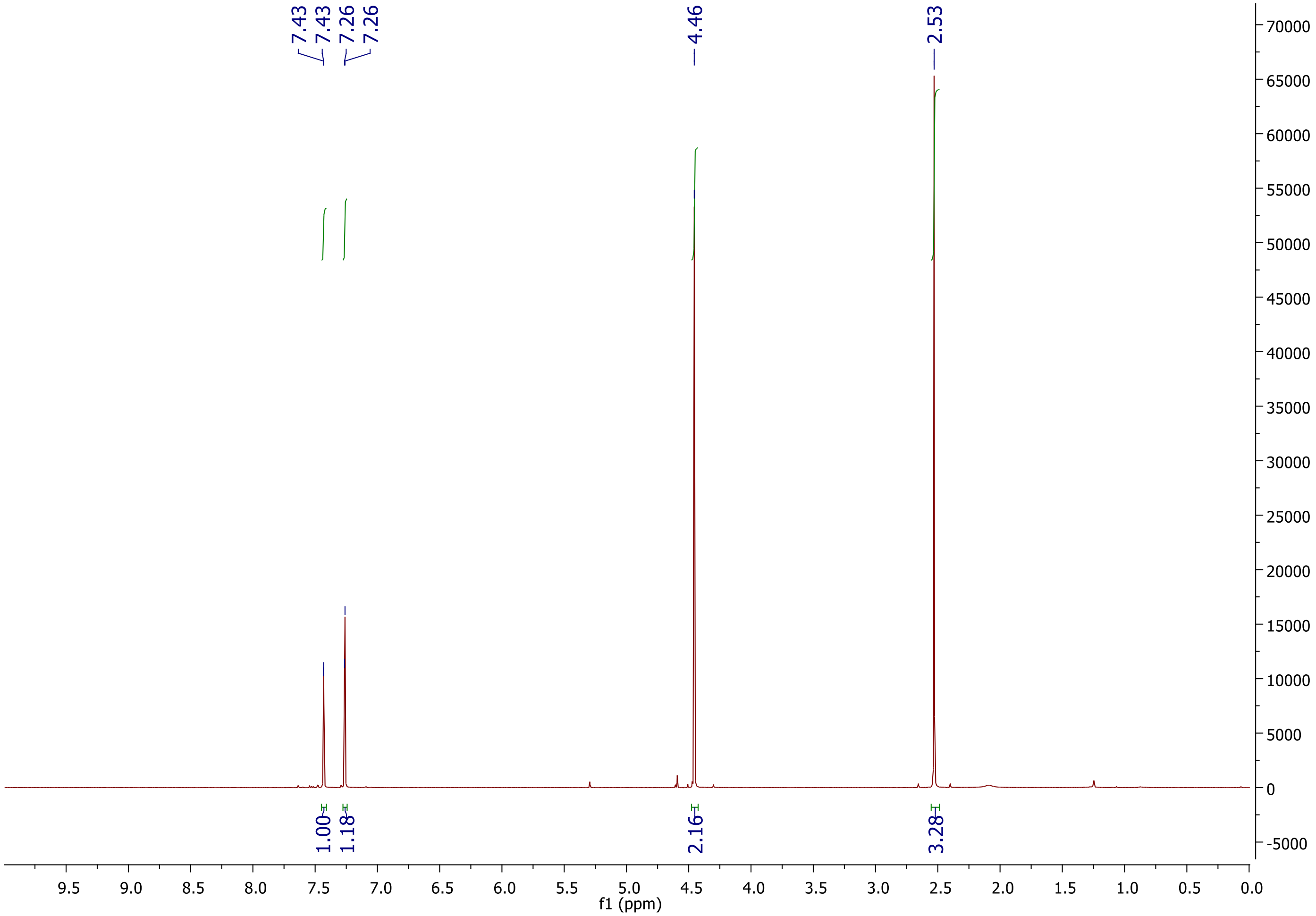

4-Bromo-2-(bromomethyl)-6-methylpyridine S3

^{13}C-NMR (126 MHz, $CDCl_3$)

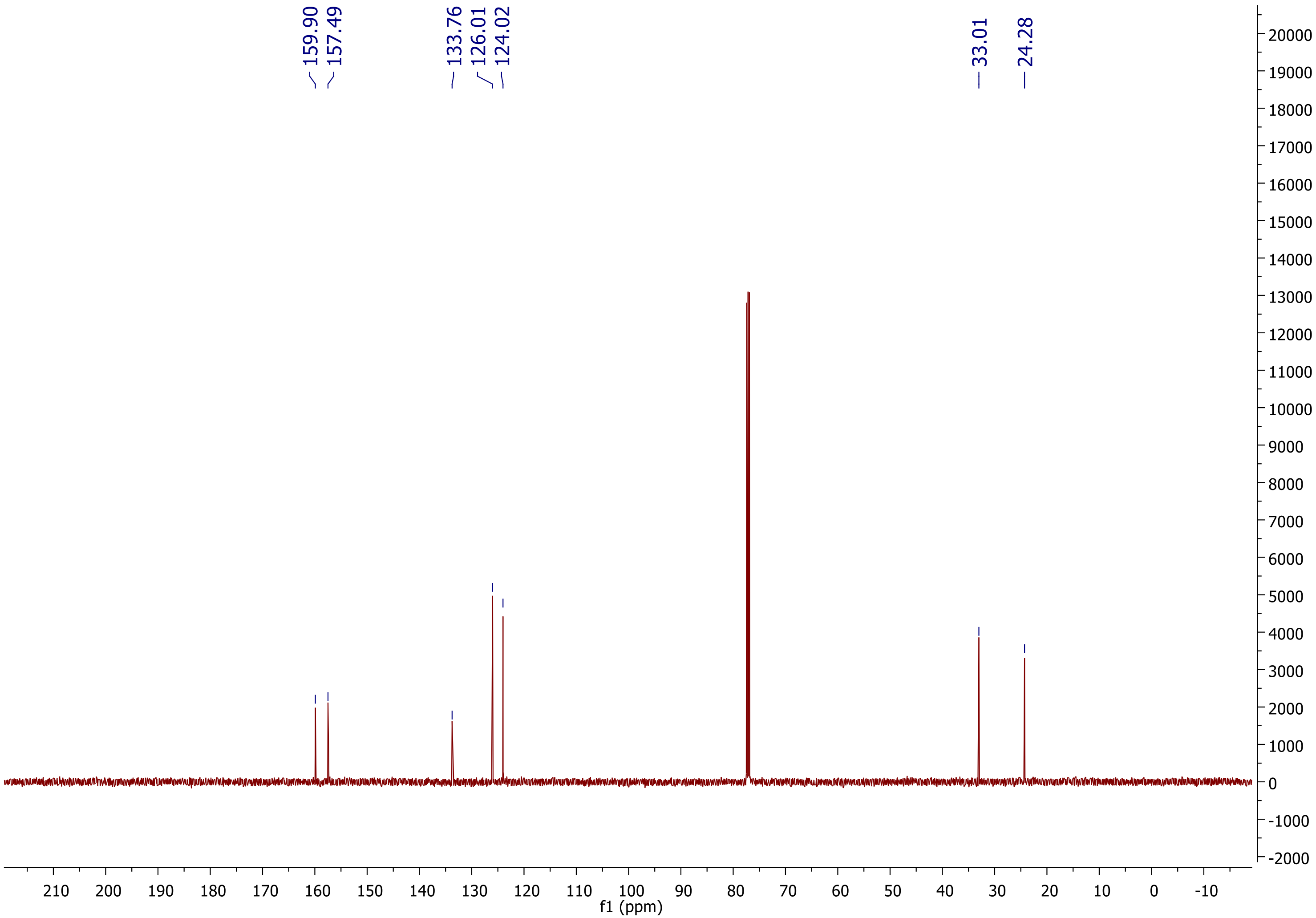

4-Bromo-2-(dibromomethyl)-6-methylpyridine S5

^{1}H-NMR (500 MHz, $CDCl_3$).

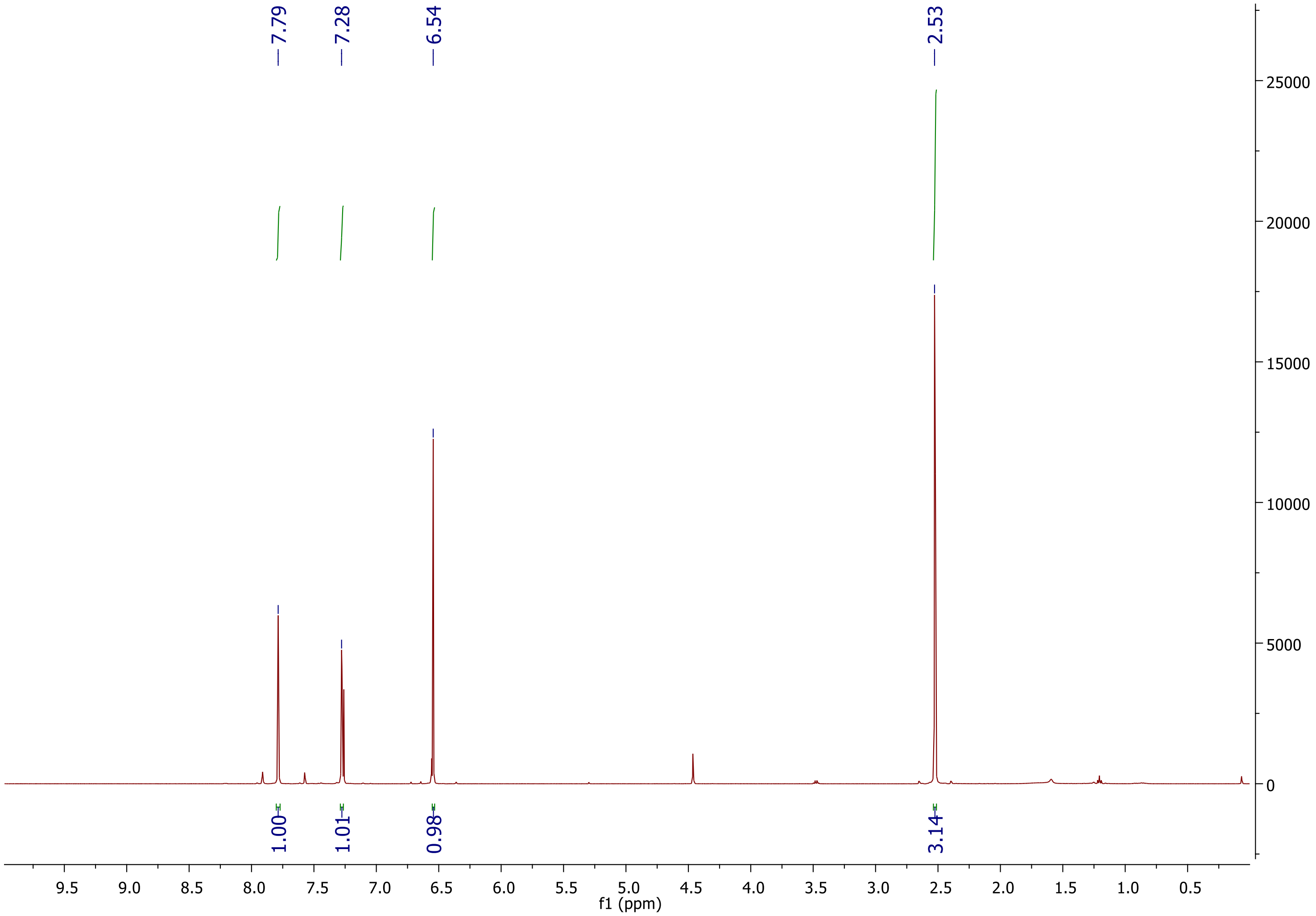

4-Bromo-2-(dibromomethyl)-6-methylpyridine S5

^{13}C-NMR (101 MHz, $CDCl_3$)

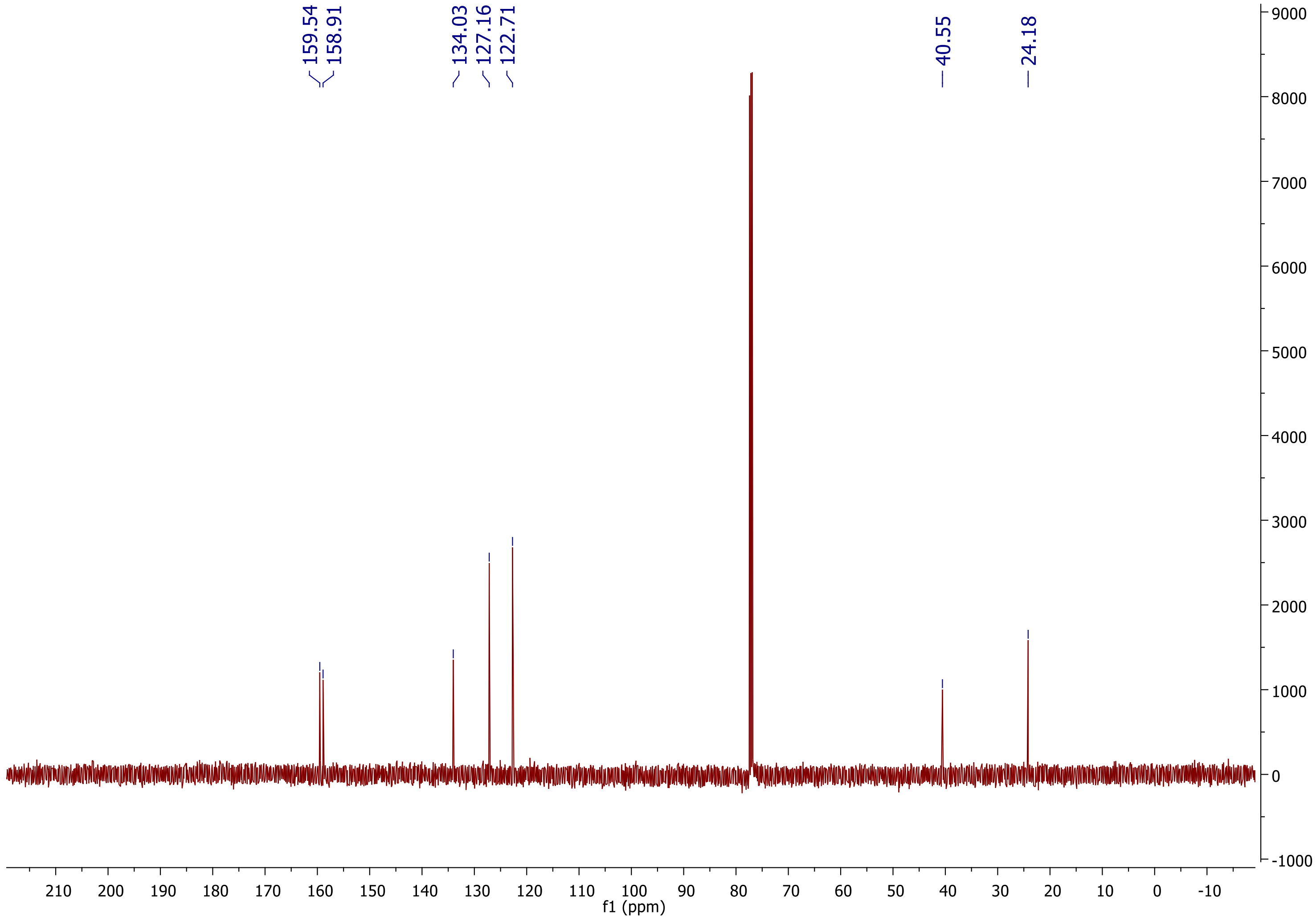

4-Bromo-2,6-bis(bromomethyl)pyridine S17

^{1}H-NMR (500 MHz, $CDCl_3$).

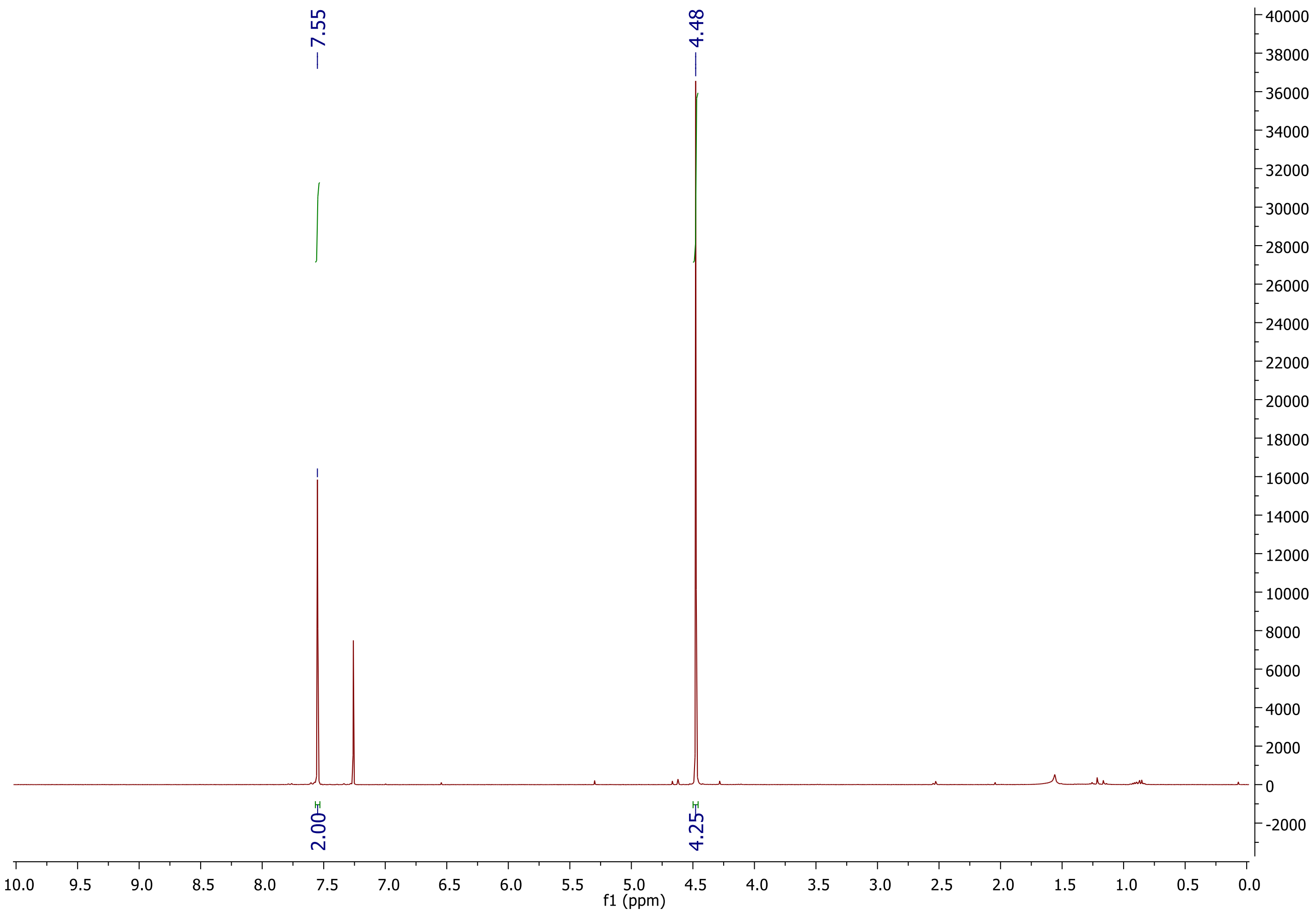

4-Bromo-2,6-bis(bromomethyl)pyridine S17

^{13}C-NMR (101 MHz, $CDCl_3$)

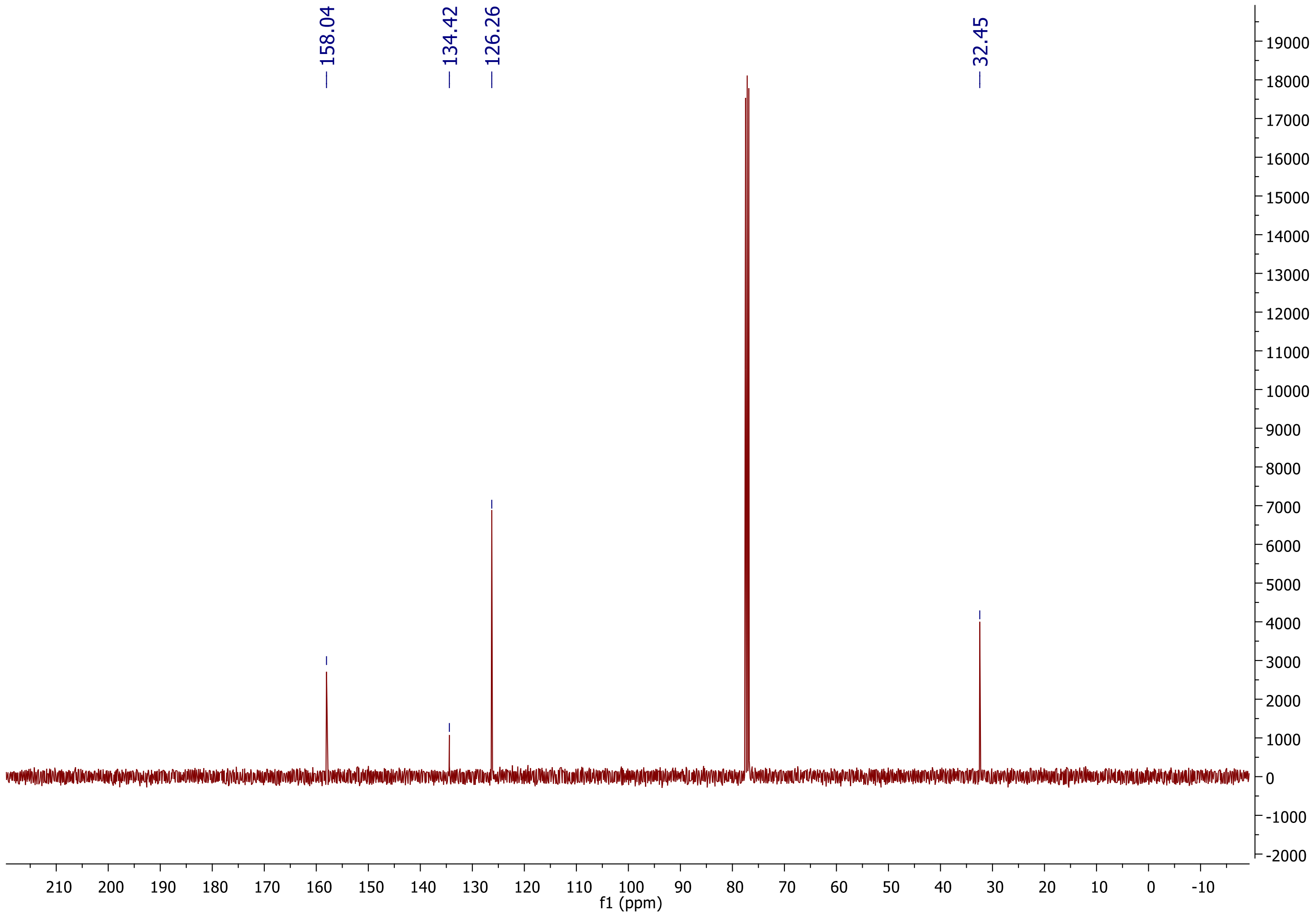

Ethyl 3-(4-bromo-6-methylpyridin-2-yl)propanoate S6

^{1}H-NMR (500 MHz, $CDCl_3$).

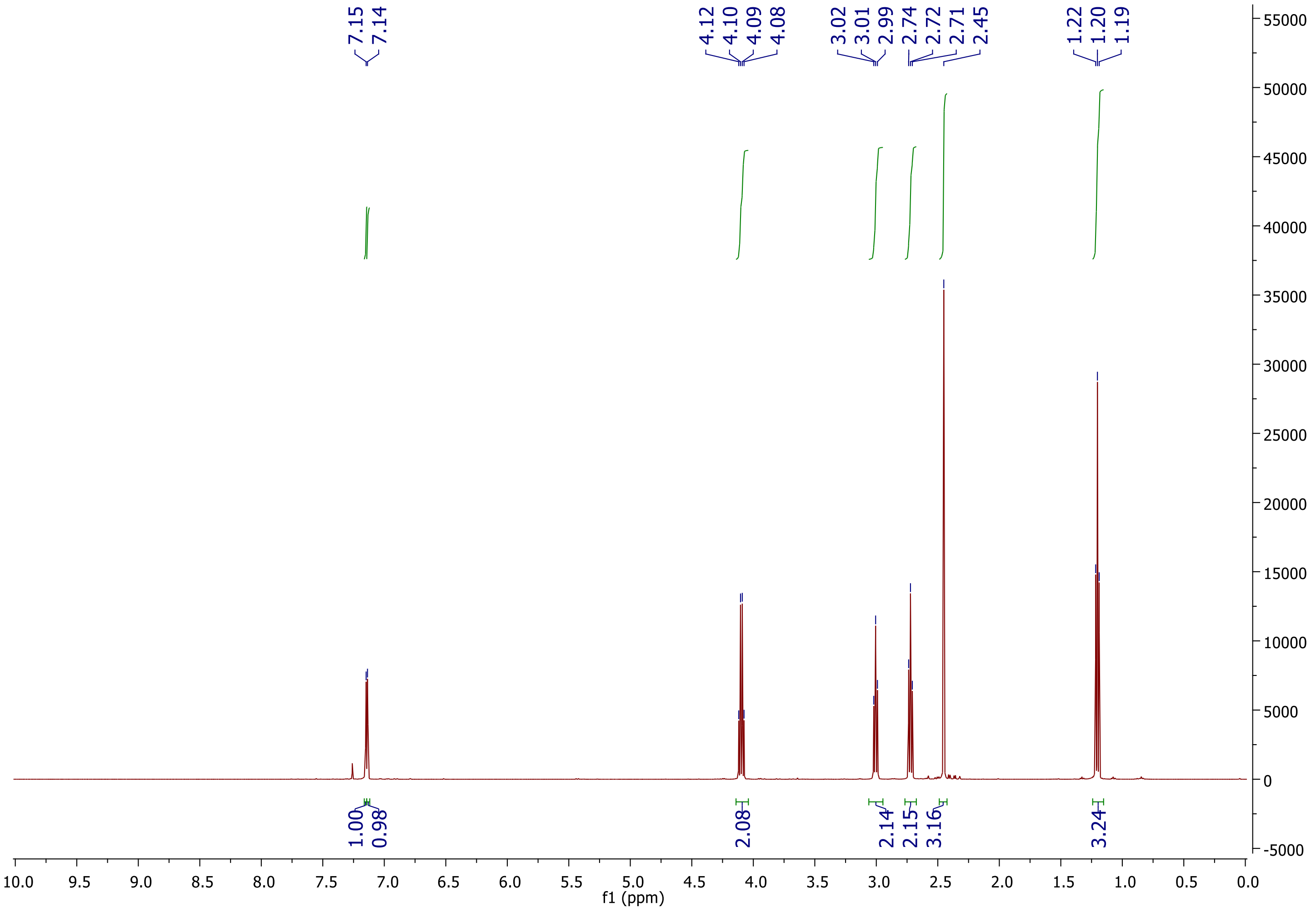

Ethyl 3-(4-bromo-6-methylpyridin-2-yl)propanoate S6

^{13}C-NMR (126 MHz, $CDCl_3$)

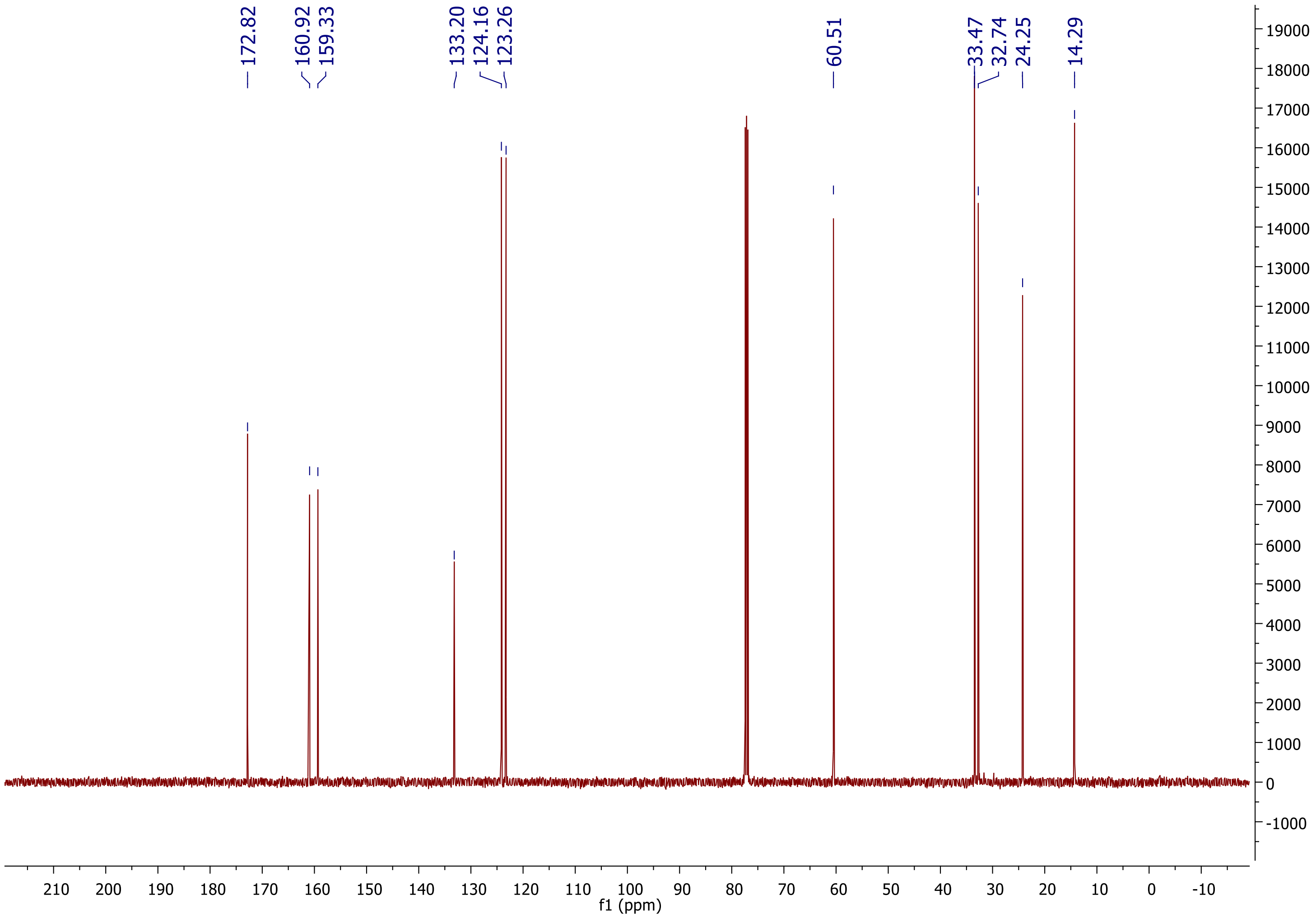

Diethyl 2-[(4-bromo-6-methylpyridin-2-yl)methyl]butanedioate S7

^{1}H-NMR (500 MHz, $CDCl_3$).

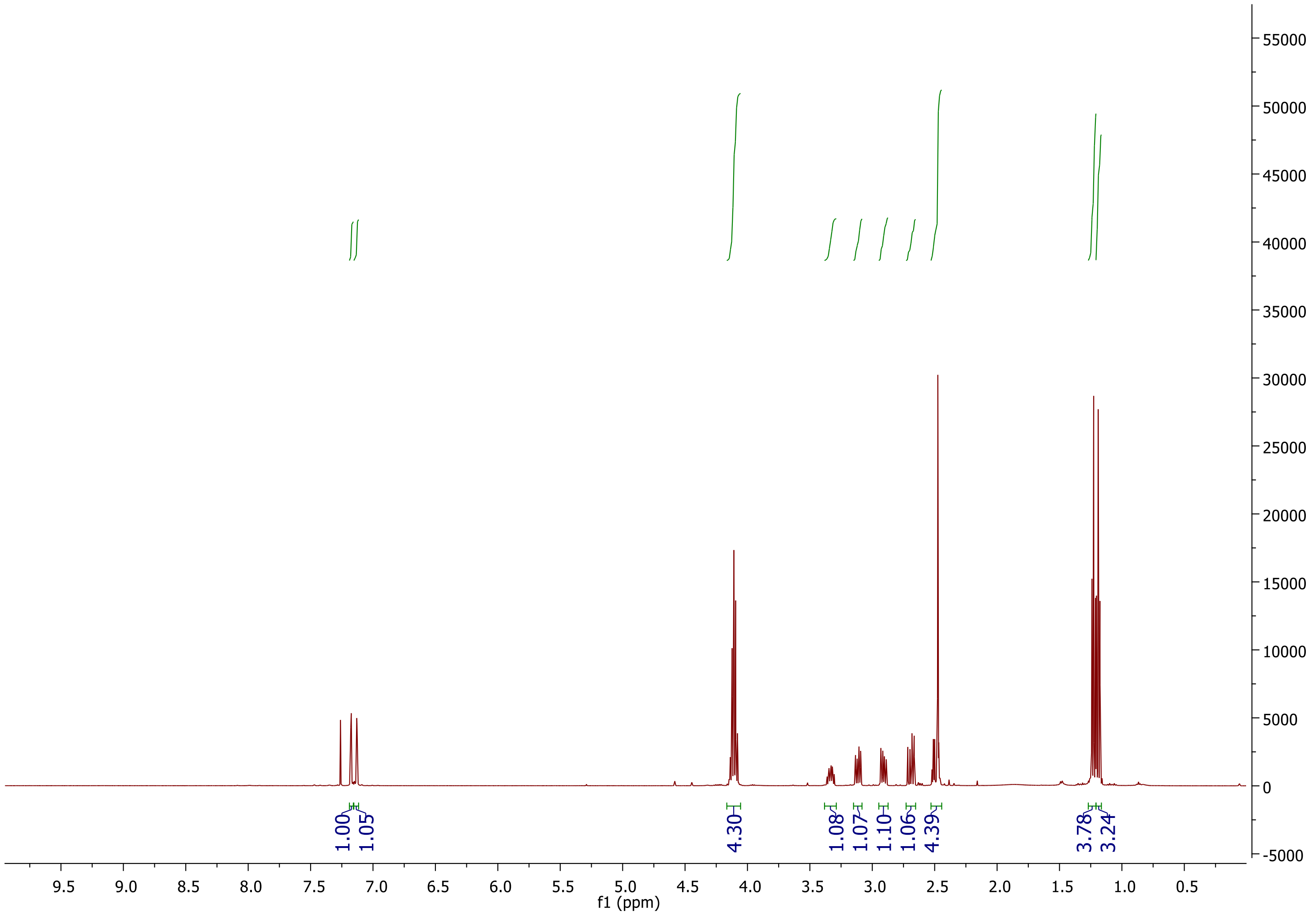

Diethyl 2-[(4-bromo-6-methylpyridin-2-yl)methyl]butanedioate S7

^{13}C-NMR (126 MHz, $CDCl_3$)

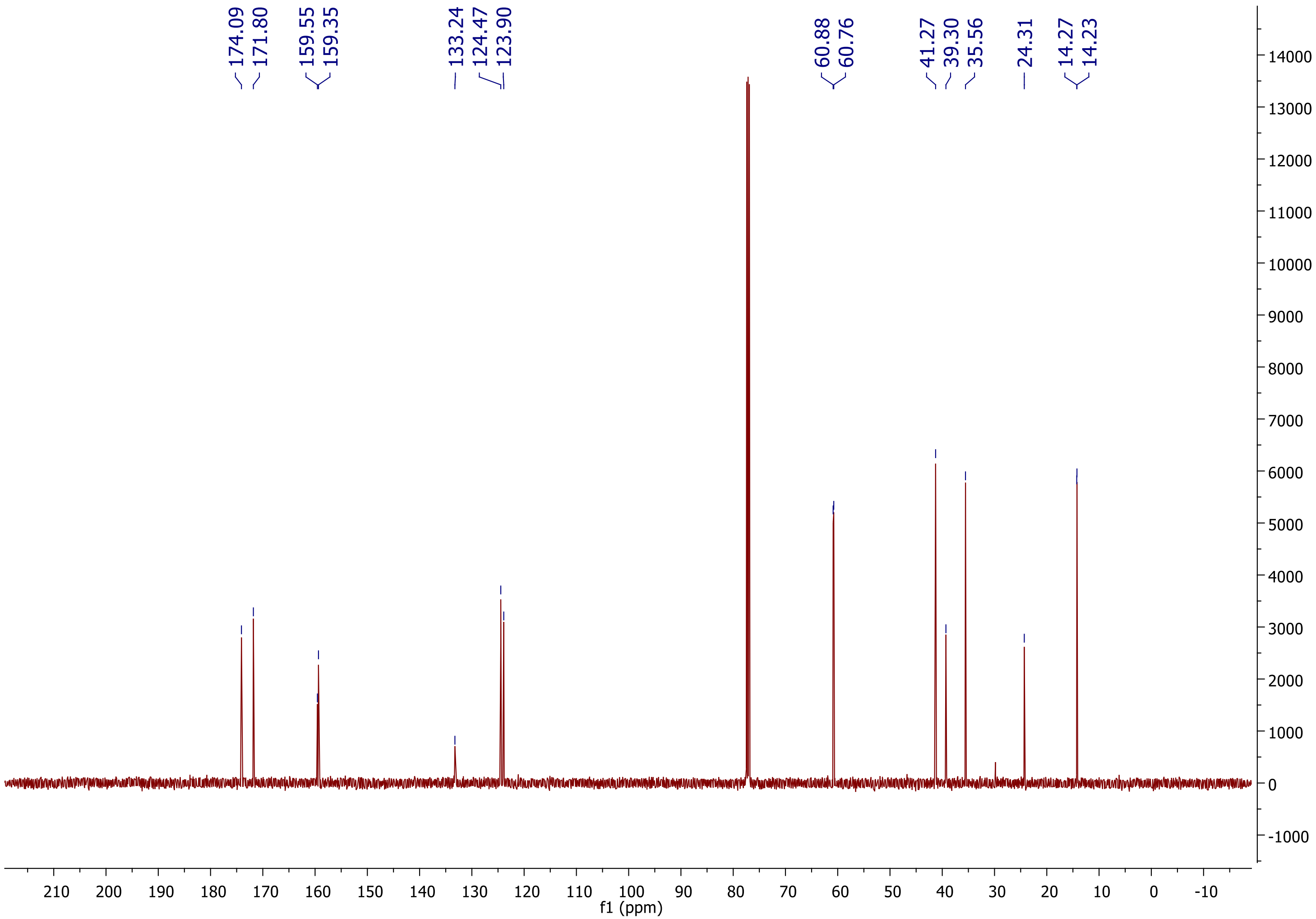

Diethyl 2,2'-(((4-bromo-6-methylpyridin-2-yl)methyl)azanediyl)diacetate S8

^{1}H-NMR (500 MHz, $CDCl_3$).

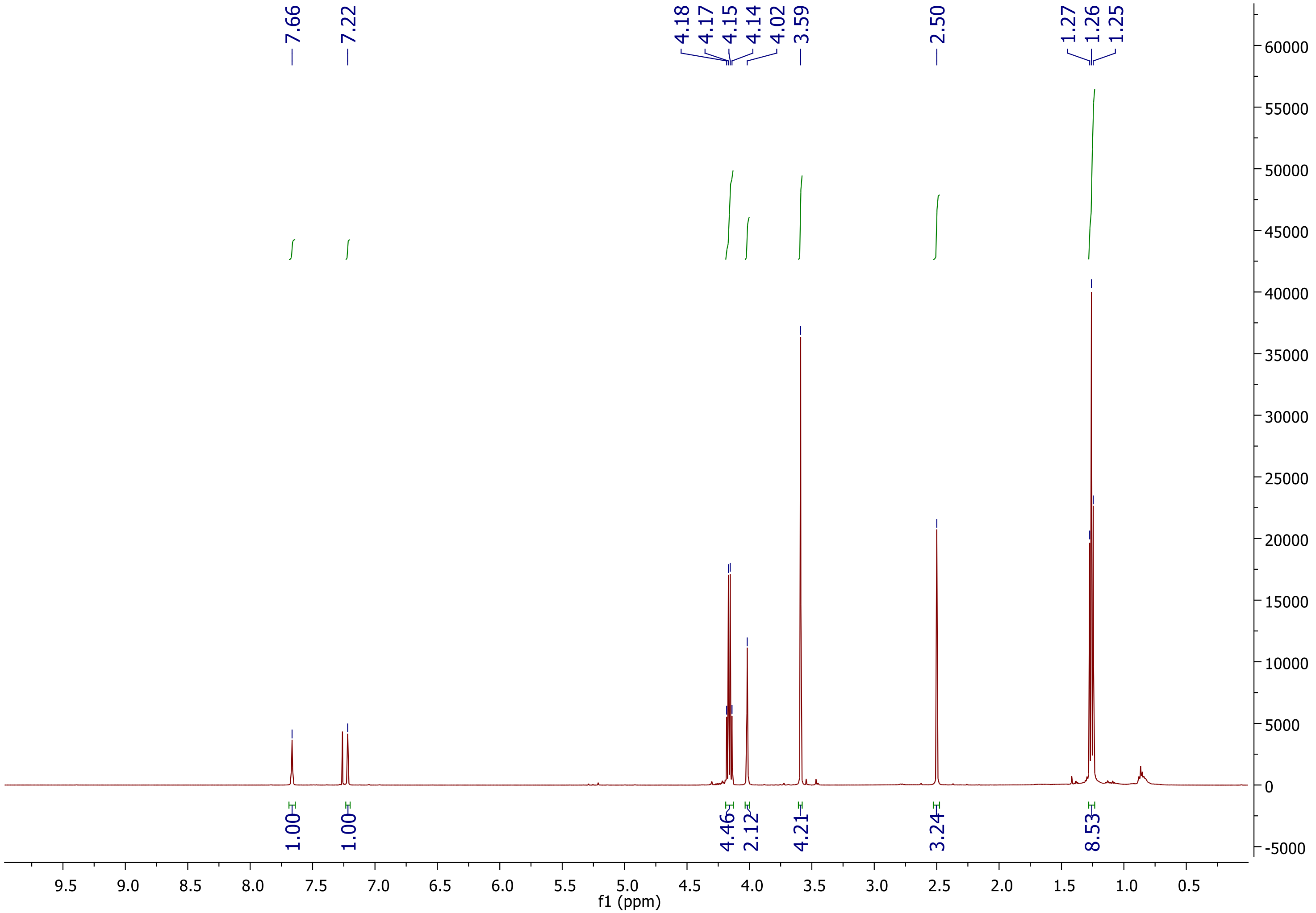

Diethyl 2,2'-(((4-bromo-6-methylpyridin-2-yl)methyl)azanediyl)diacetate S8

^{13}C-NMR (126 MHz, $CDCl_3$)

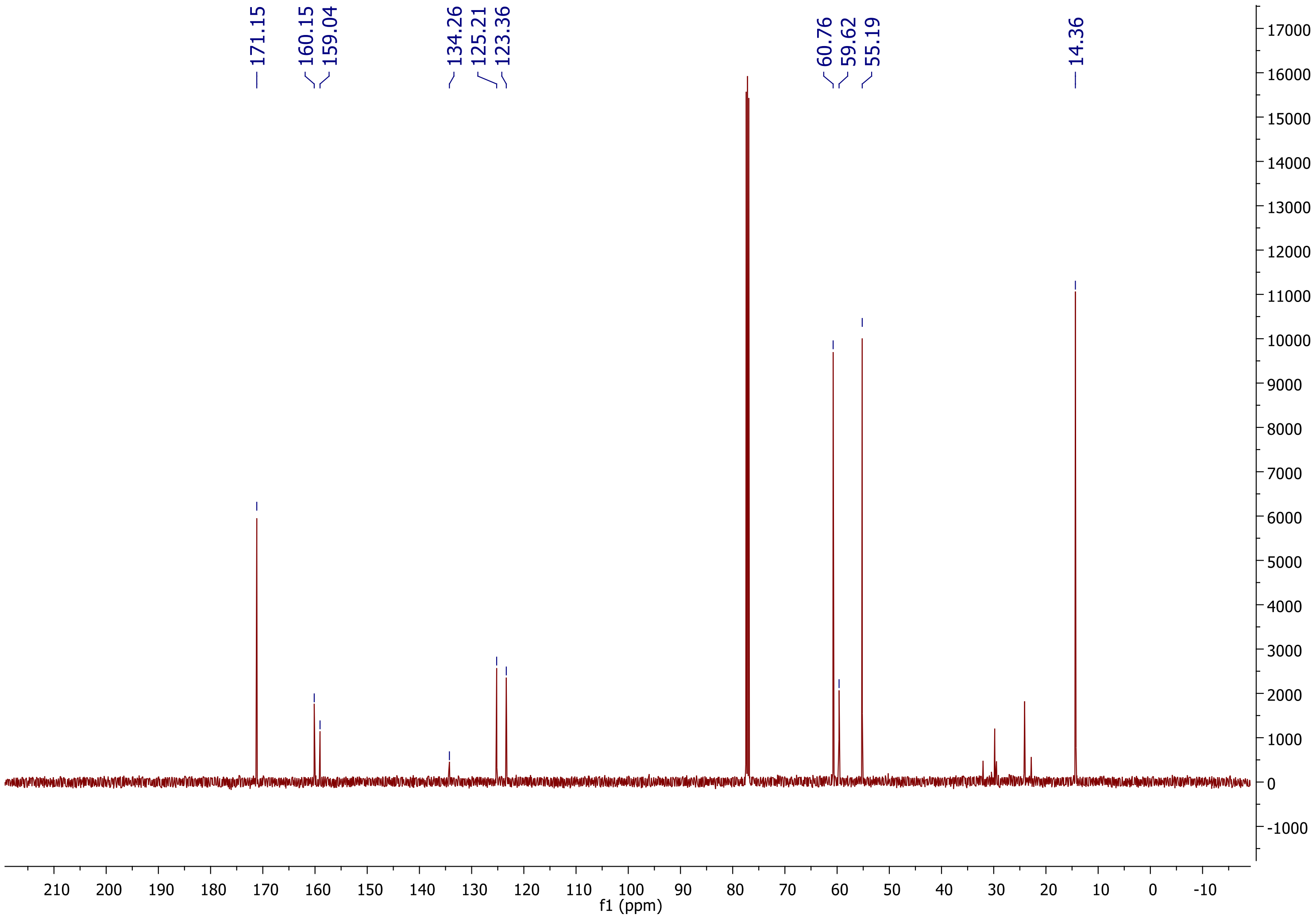

2-(4-Bromo-6-methylpyridin-2-yl)acetonitrile S9

^{1}H-NMR (400 MHz, $CDCl_3$).

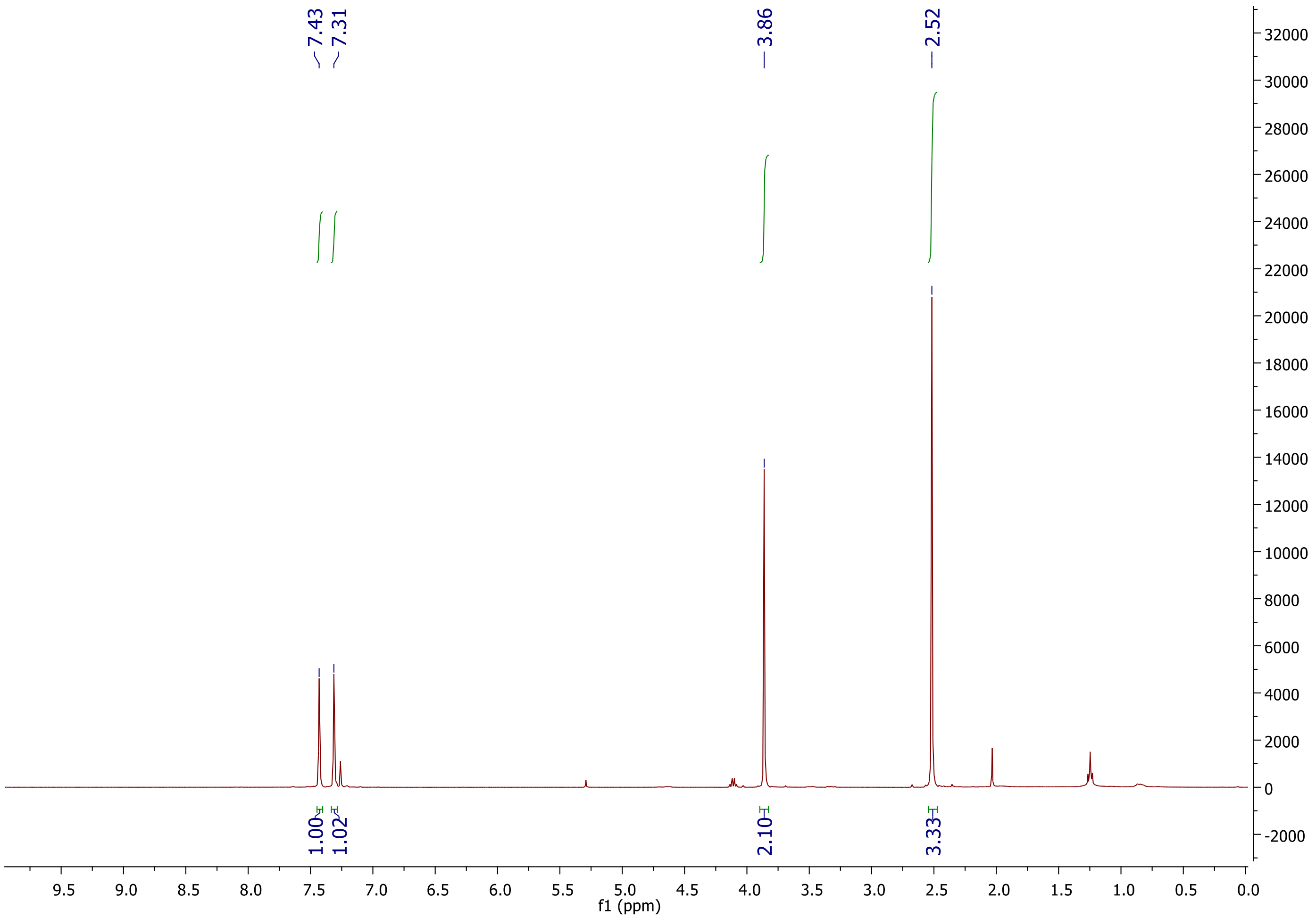

2-(4-Bromo-6-methylpyridin-2-yl)acetonitrile S9

^{13}C-NMR (101 MHz, $CDCl_3$)

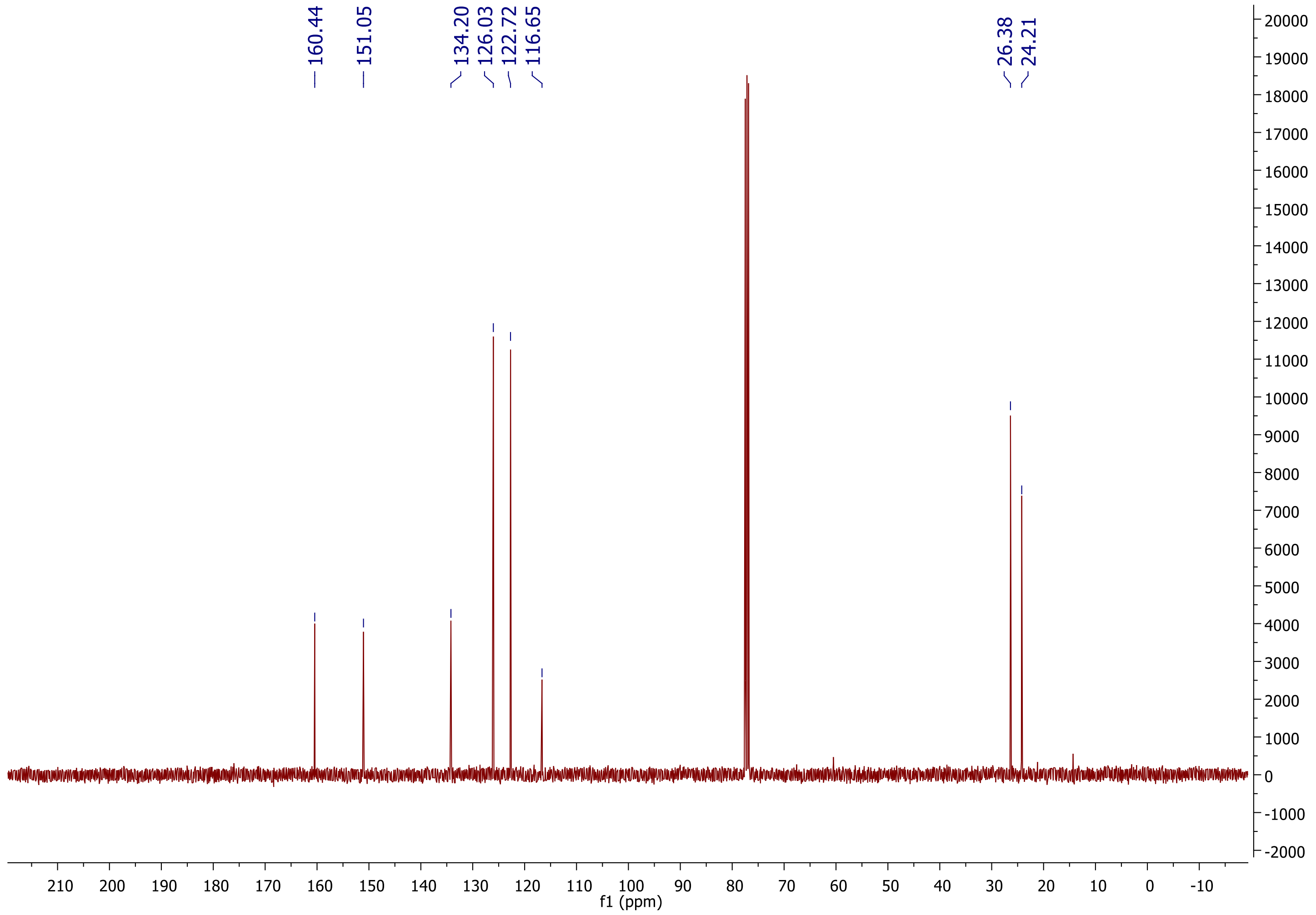

Methyl 2-(4-bromo-6-methylpyridin-2-yl)acetate S10

^{1}H-NMR (500 MHz, $CDCl_3$).

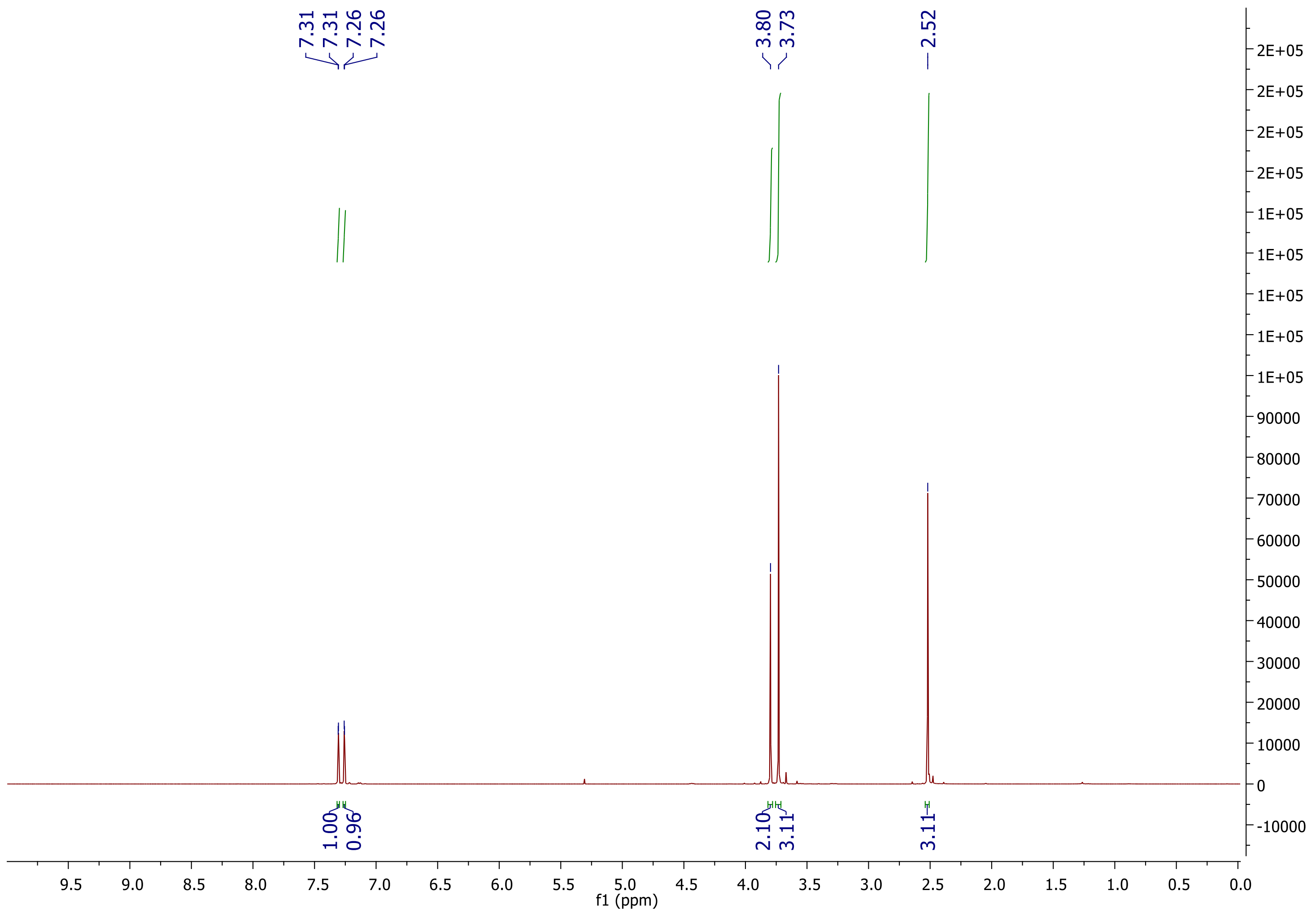

Methyl 2-(4-bromo-6-methylpyridin-2-yl)acetate S10

^{13}C-NMR (126 MHz, $CDCl_3$)

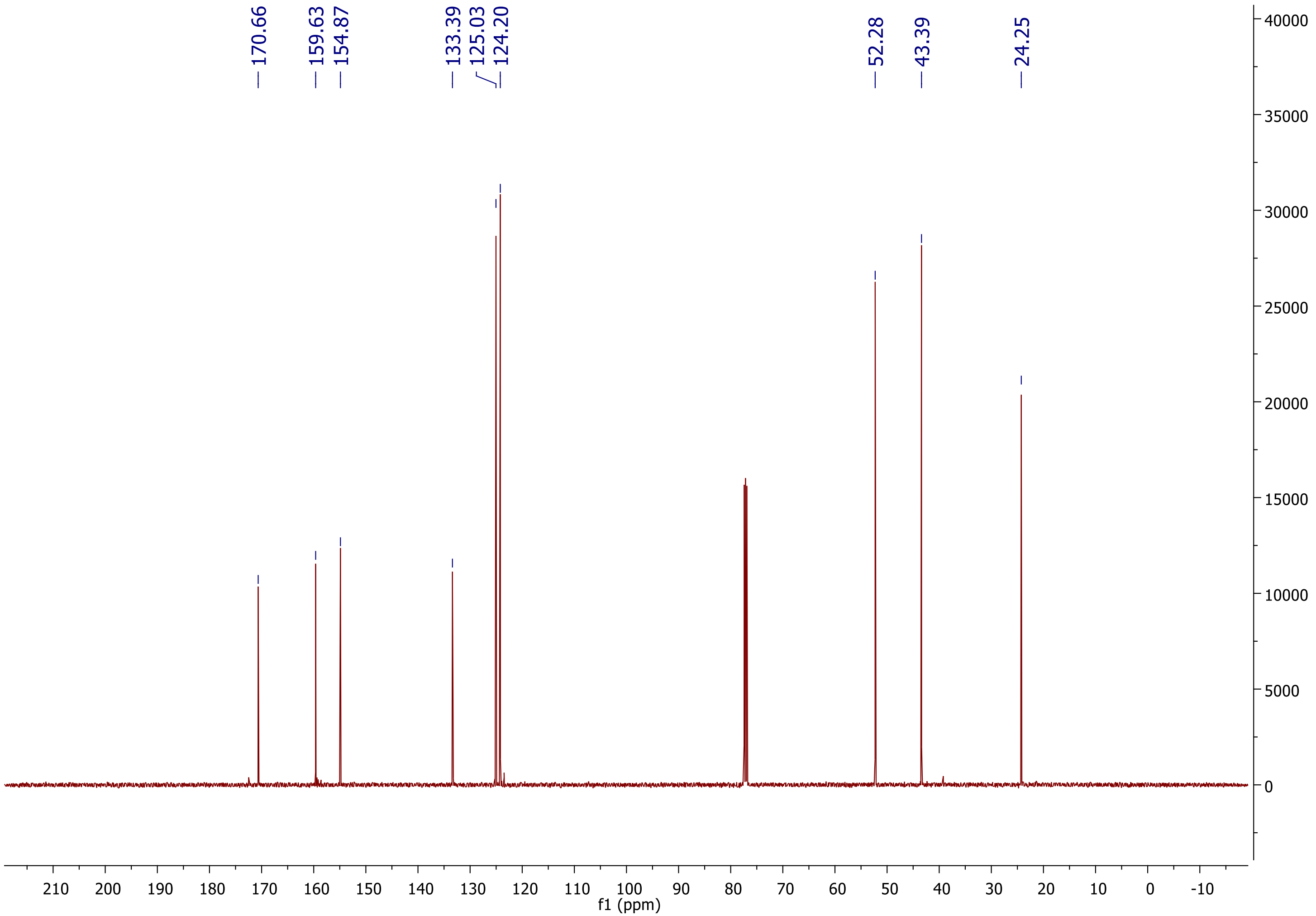

2-(4-Bromo-6-methylpyridin-2-yl)ethan-1-ol S11

^{1}H-NMR (500 MHz, $CDCl_3$).

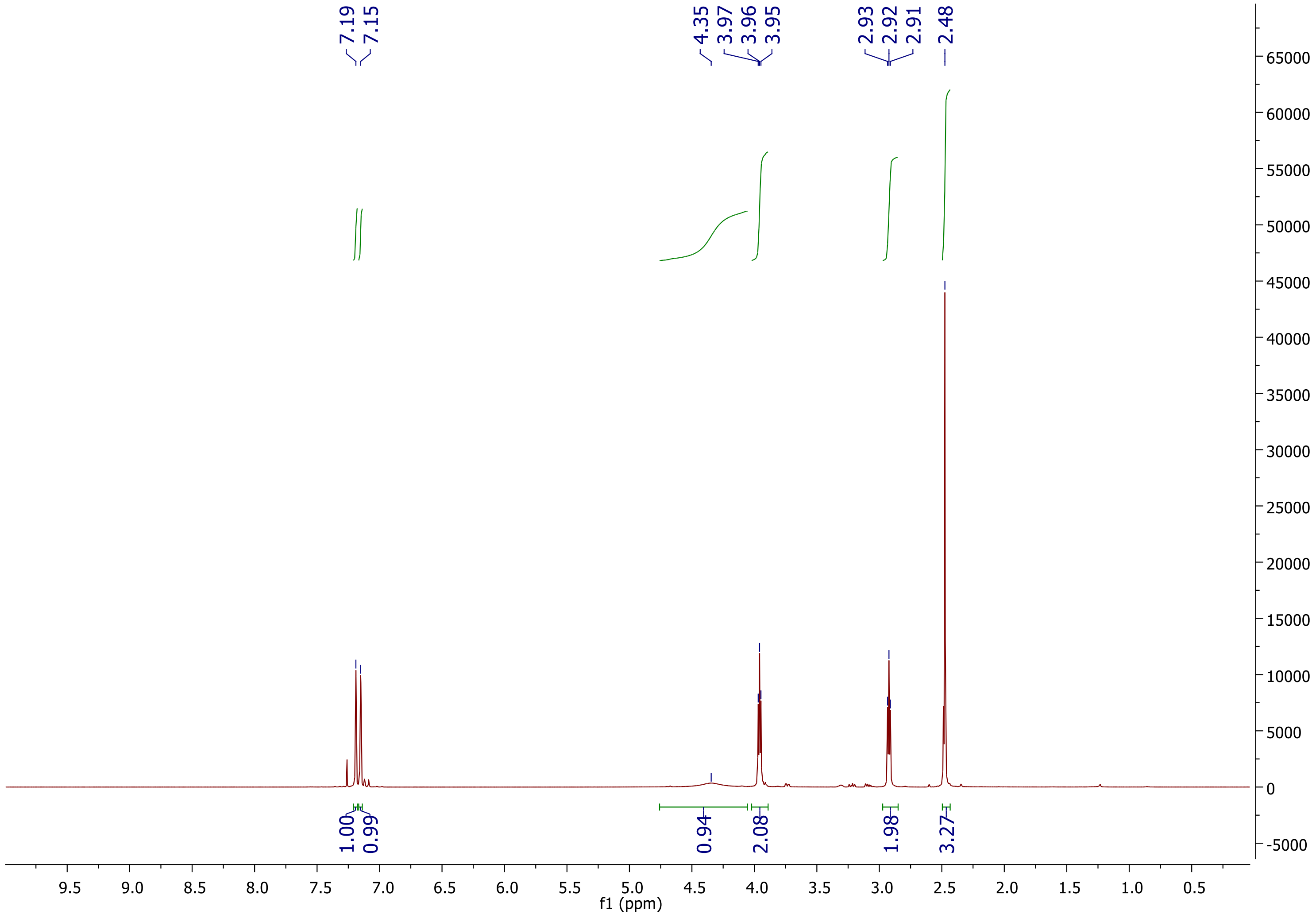

2-(4-Bromo-6-methylpyridin-2-yl)ethan-1-ol S11

^{13}C-NMR (126 MHz, $CDCl_3$)

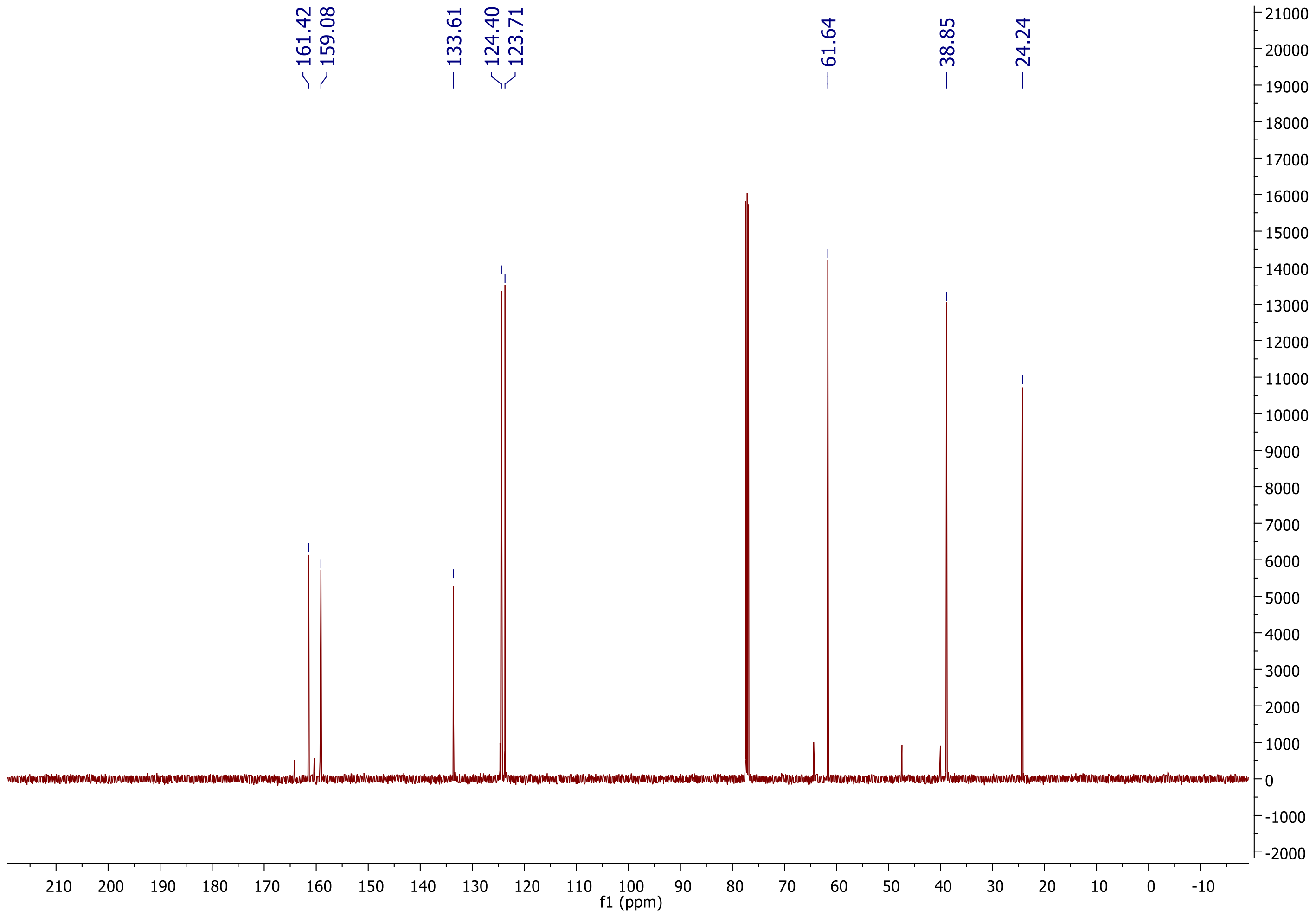

2-(6-Methylpyridin-2-yl)ethan-1-ol S11a

^{1}H-NMR (500 MHz, $CDCl_3$).

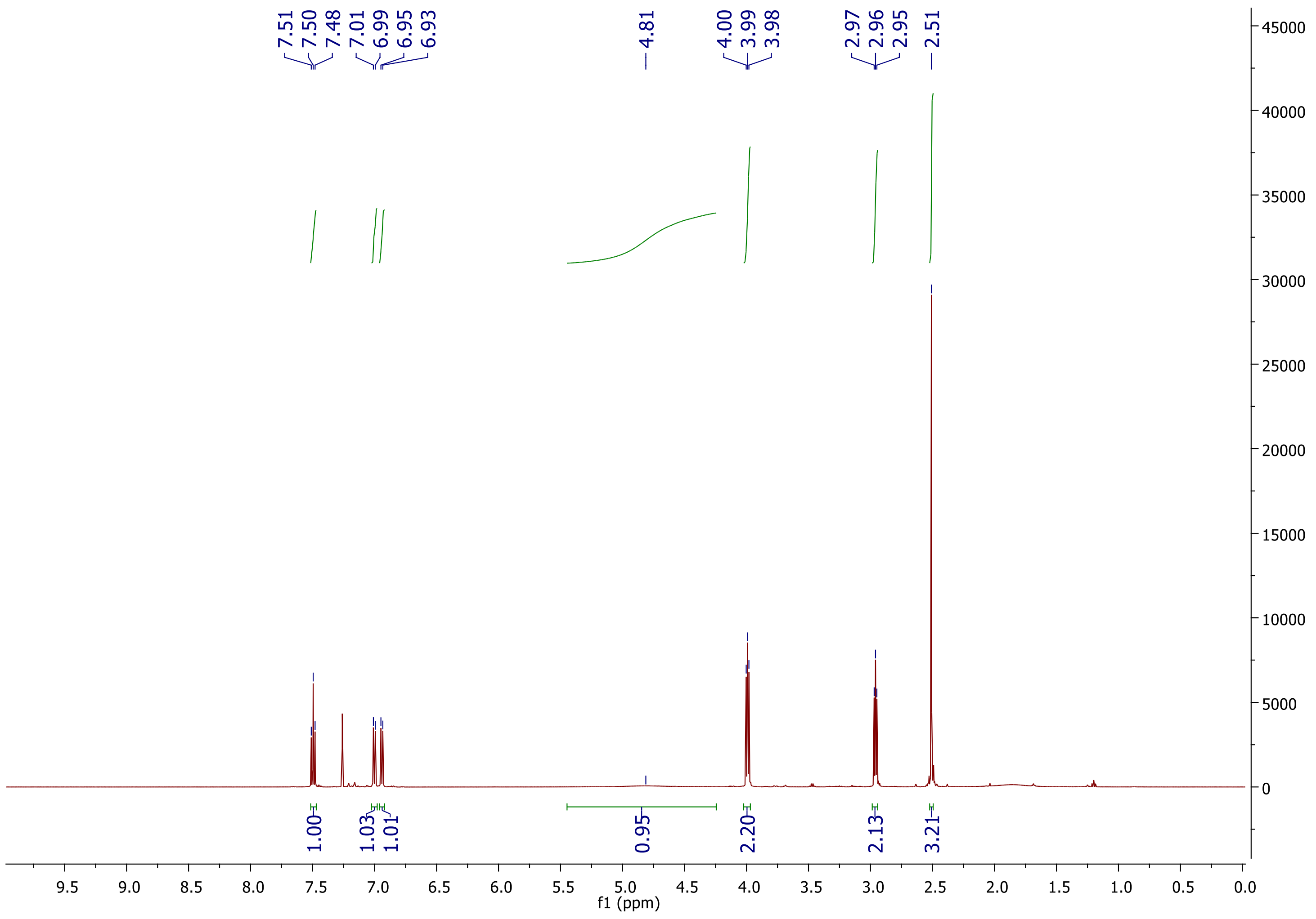

2-(6-Methylpyridin-2-yl)ethan-1-ol S11a

^{13}C-NMR (126 MHz, $CDCl_3$)

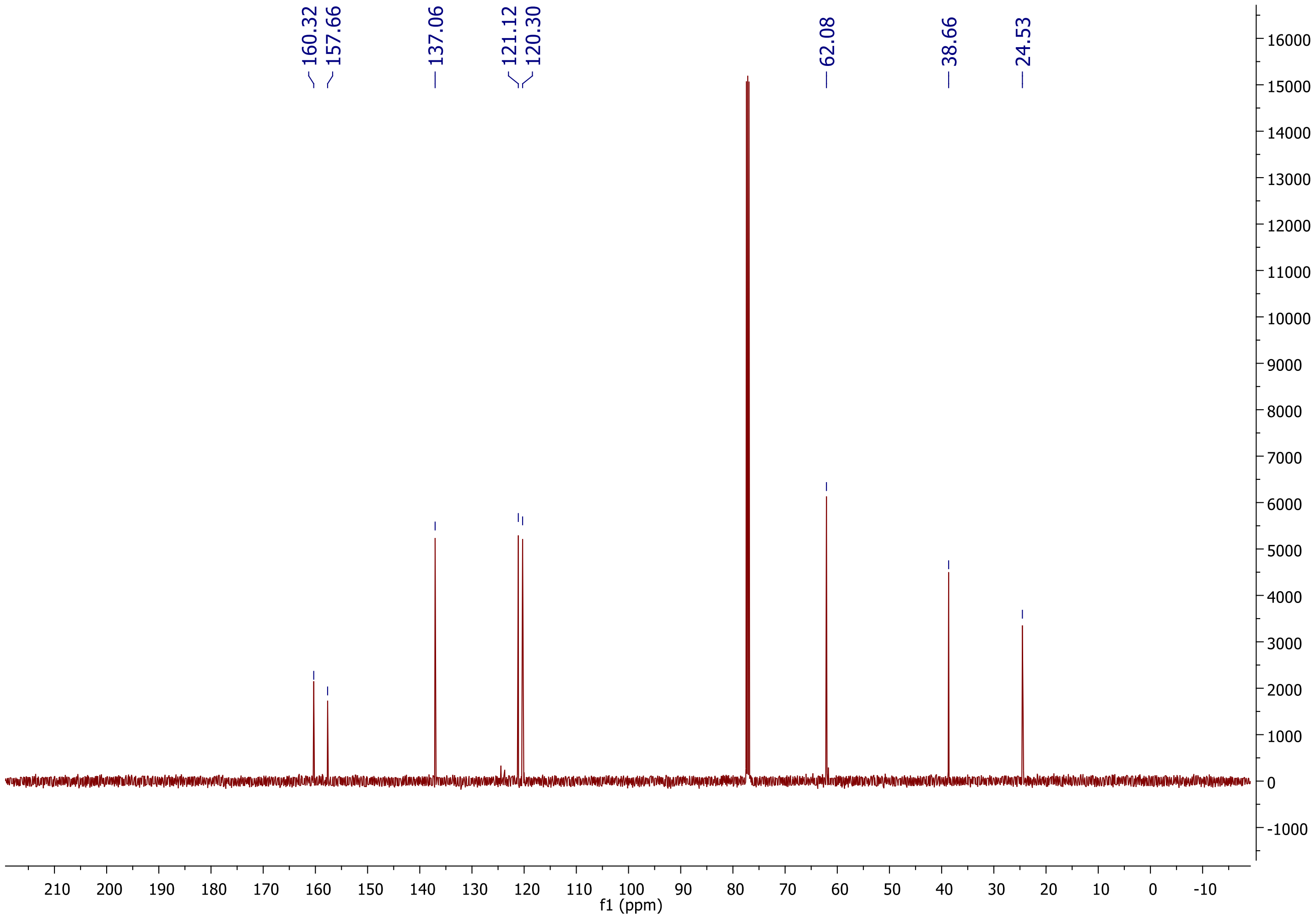

2-(4-Bromo-6-methylpyridin-2-yl)ethyl 4-methylbenzenesulfonate S12

^{1}H-NMR (500 MHz, $CDCl_3$).

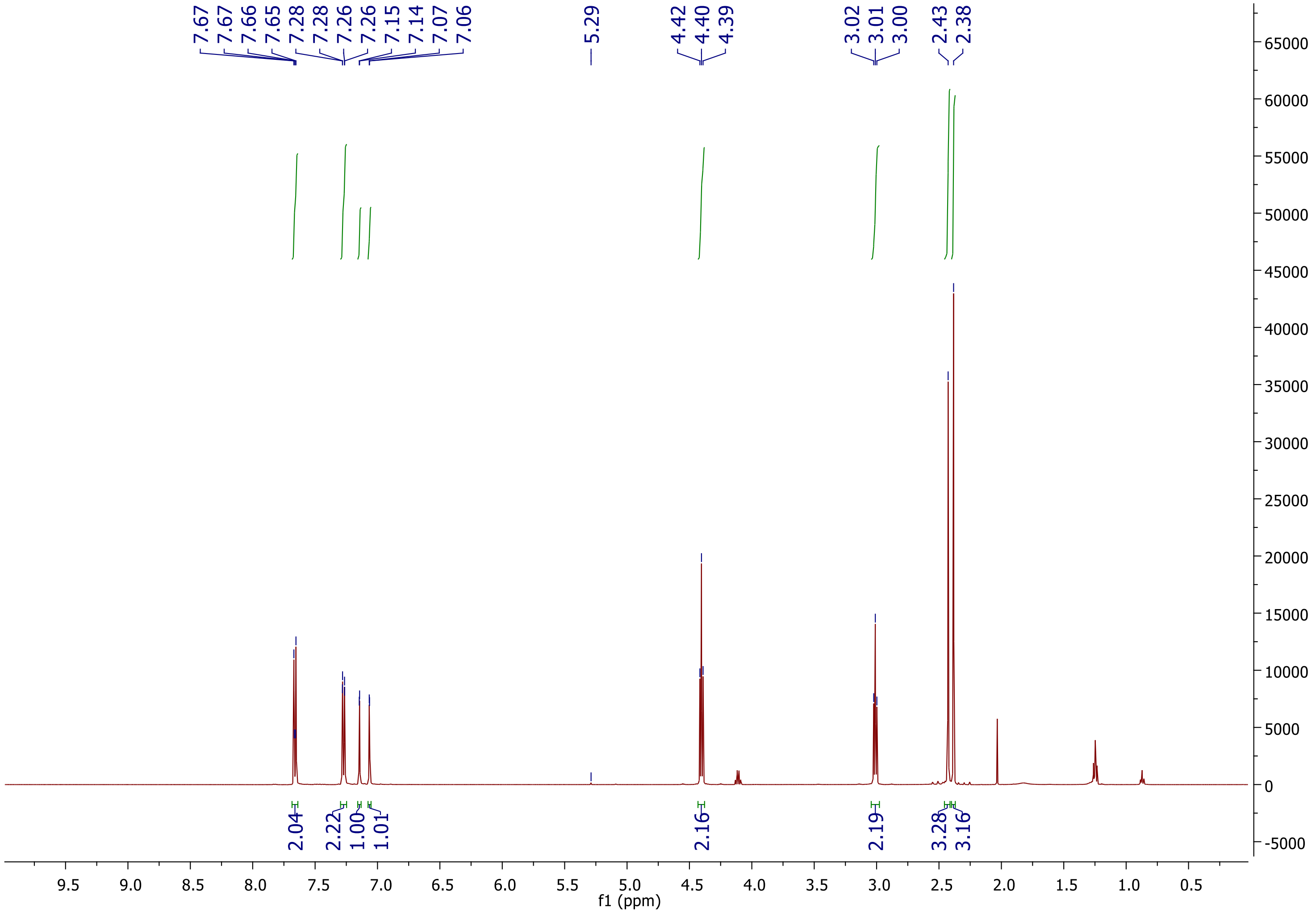

2-(4-Bromo-6-methylpyridin-2-yl)ethyl 4-methylbenzenesulfonate S12

^{13}C-NMR (126 MHz, $CDCl_3$)

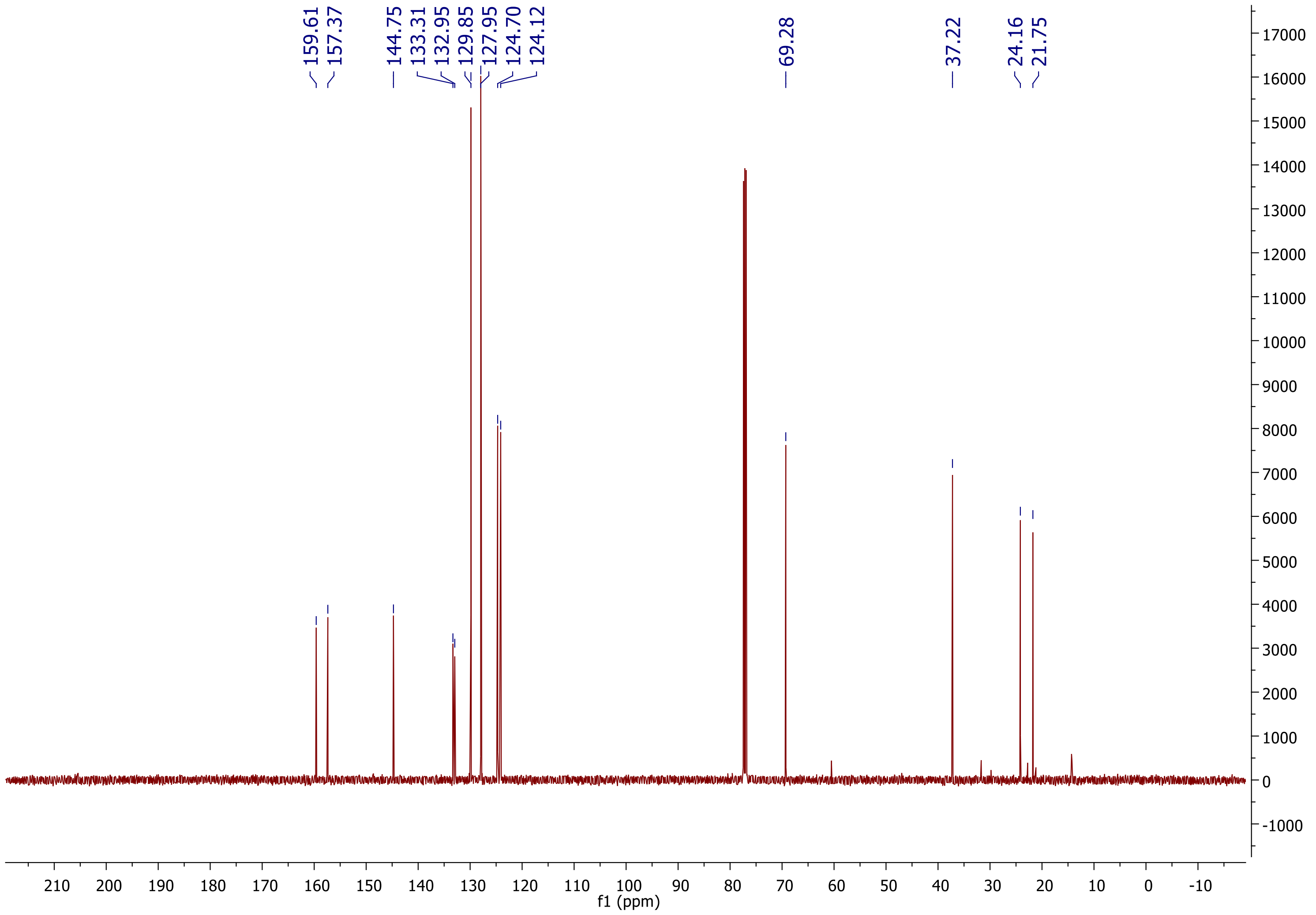

Diethyl 2,2'-((2-(4-bromo-6-methylpyridin-2-yl)ethyl)azanediyl)diacetate S15

^{1}H-NMR (500 MHz, $CDCl_3$).

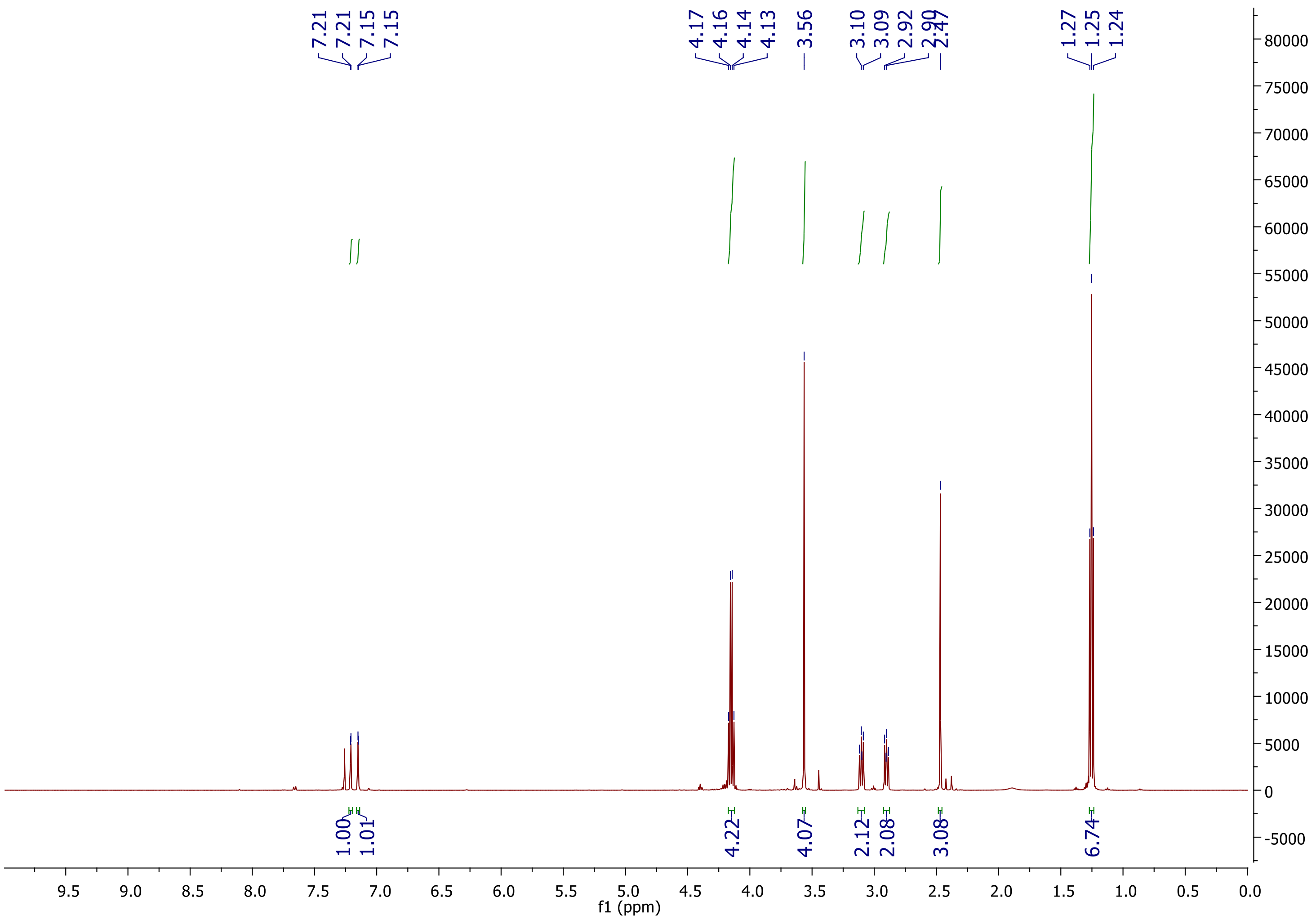

Diethyl 2,2'-((2-(4-bromo-6-methylpyridin-2-yl)ethyl)azanediyl)diacetate S15

^{13}C-NMR (126 MHz, $CDCl_3$)

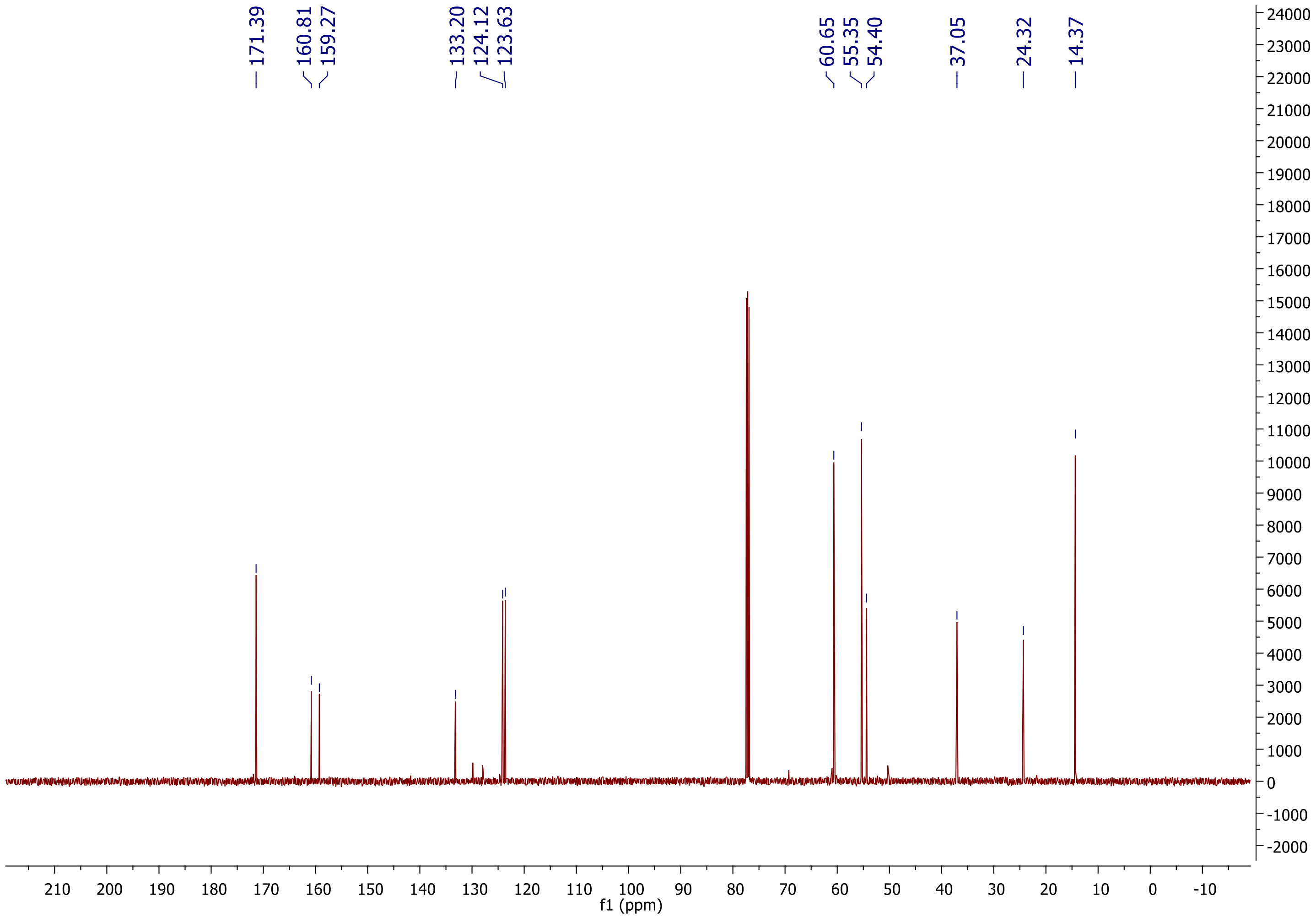

3-(4-Bromo-6-methylpyridin-2-yl)propan-1-ol S14

^{1}H-NMR (500 MHz, $CDCl_3$).

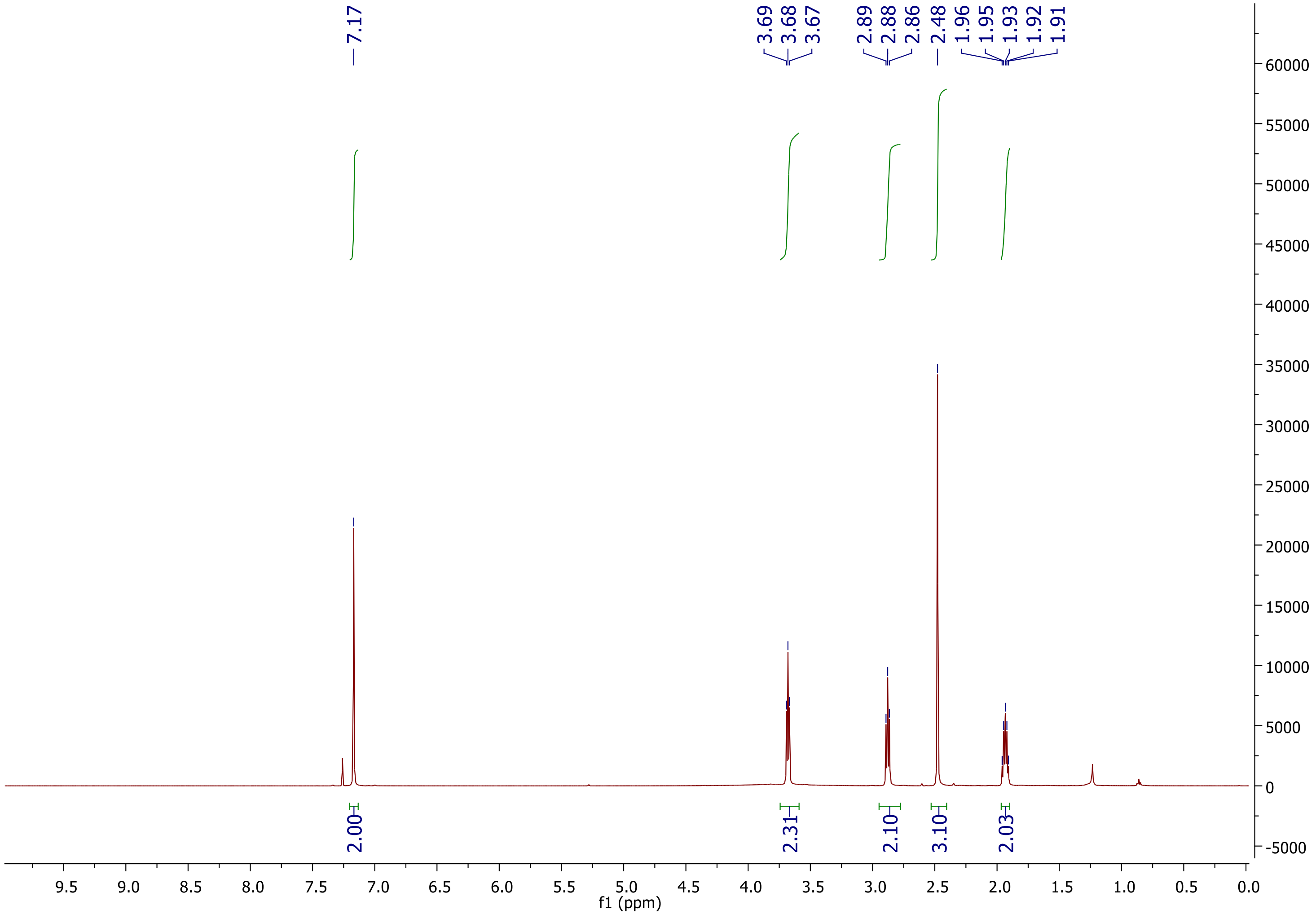

3-(4-Bromo-6-methylpyridin-2-yl)propan-1-ol S14

^{13}C-NMR (126 MHz, $CDCl_3$)

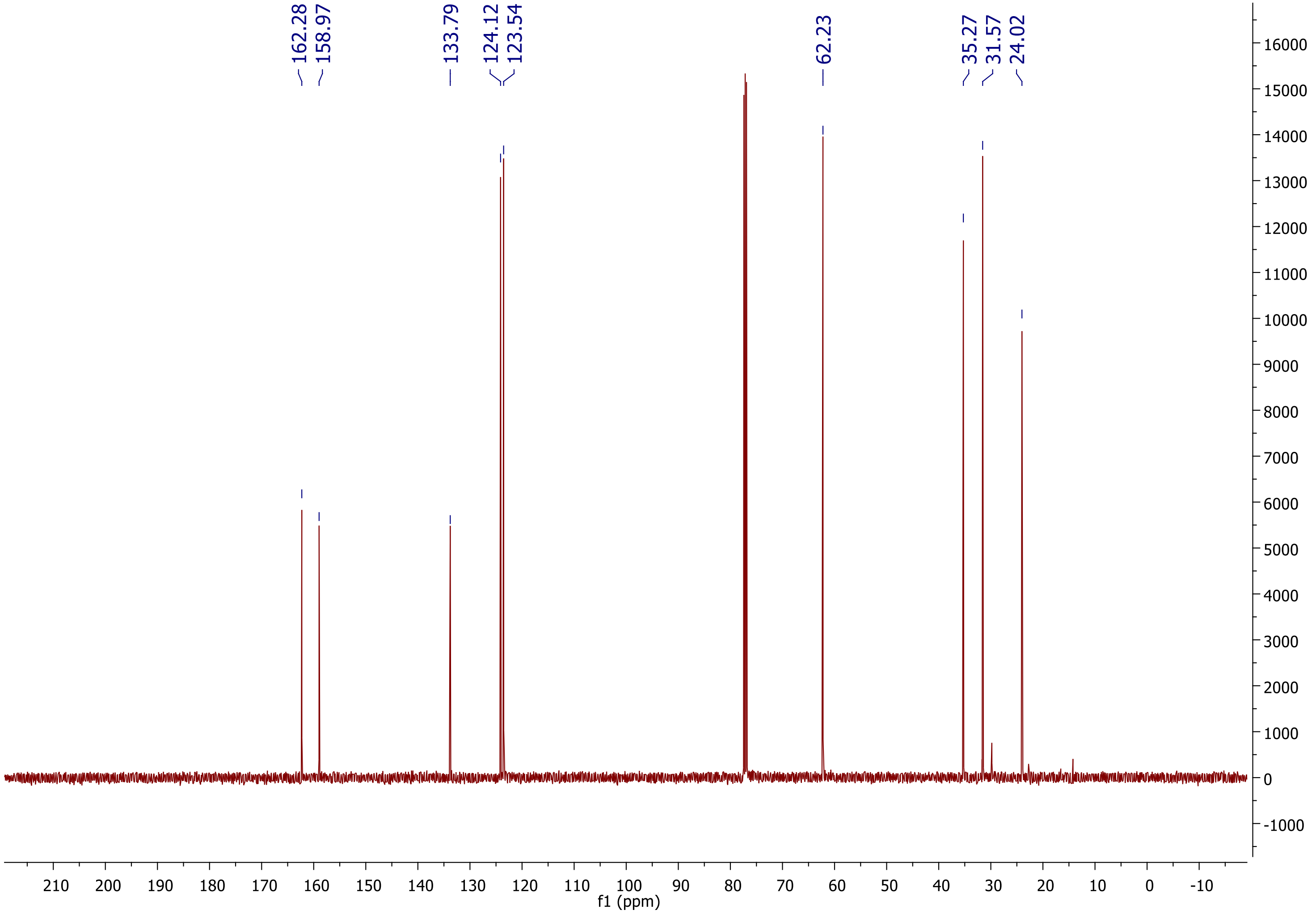

3-(6-Methylpyridin-2-yl)propan-1-ol S14a

^{1}H-NMR (500 MHz, $CDCl_3$).

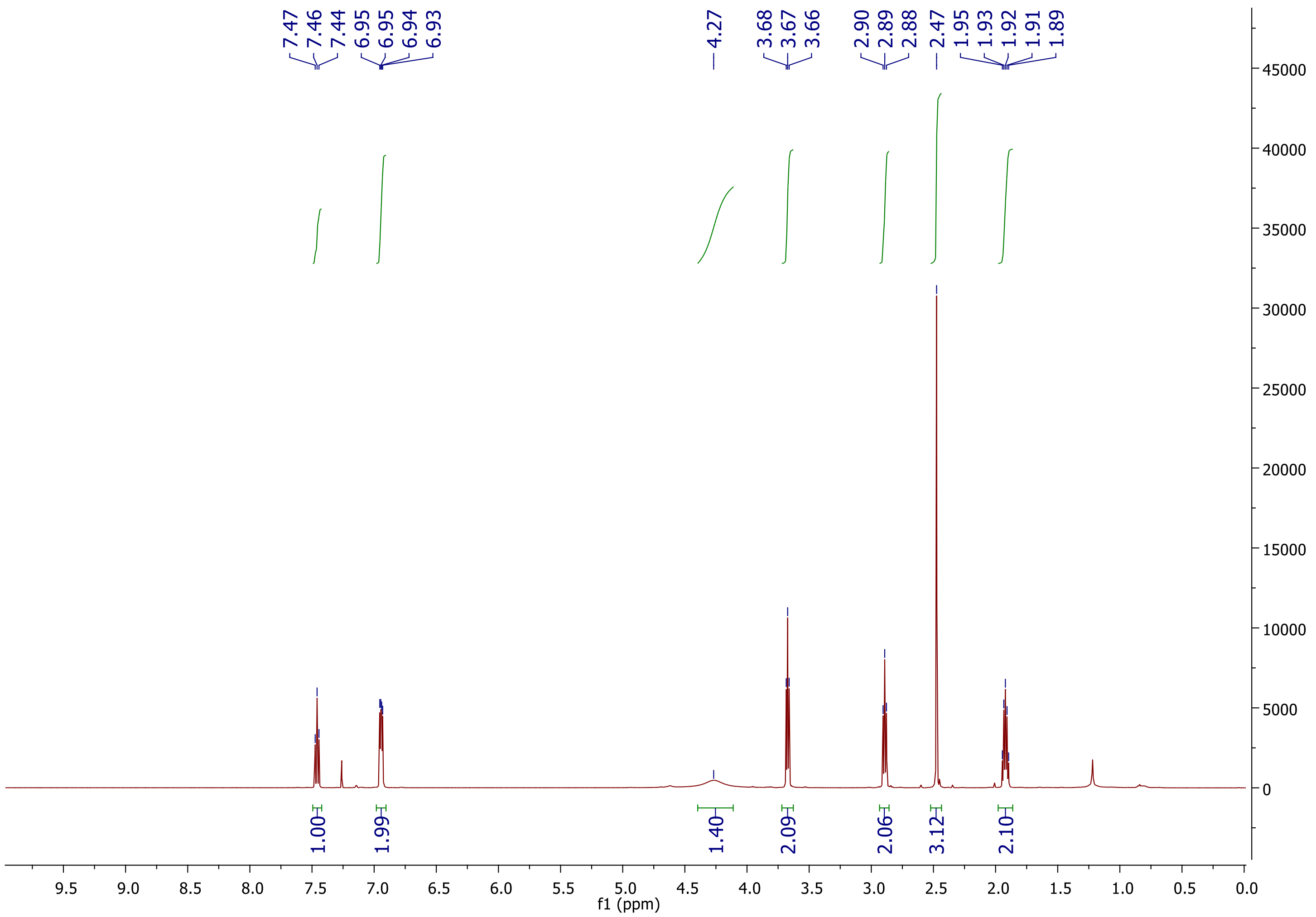

3-(6-Methylpyridin-2-yl)propan-1-ol S14a

^{13}C-NMR (126 MHz, $CDCl_3$)

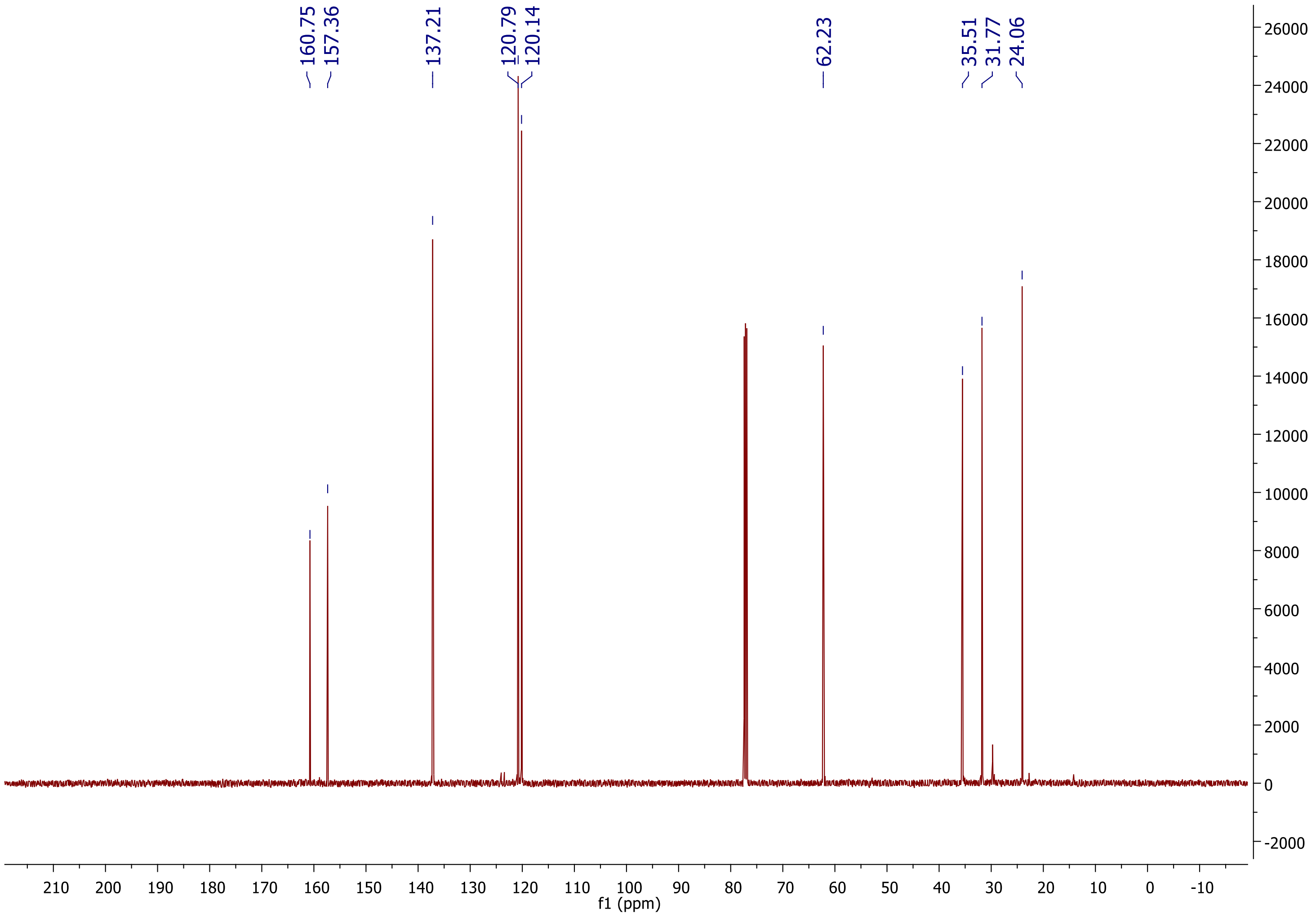

3-(4-Bromo-6-methylpyridin-2-yl)propanal S15

^{1}H-NMR (500 MHz, $CDCl_3$).

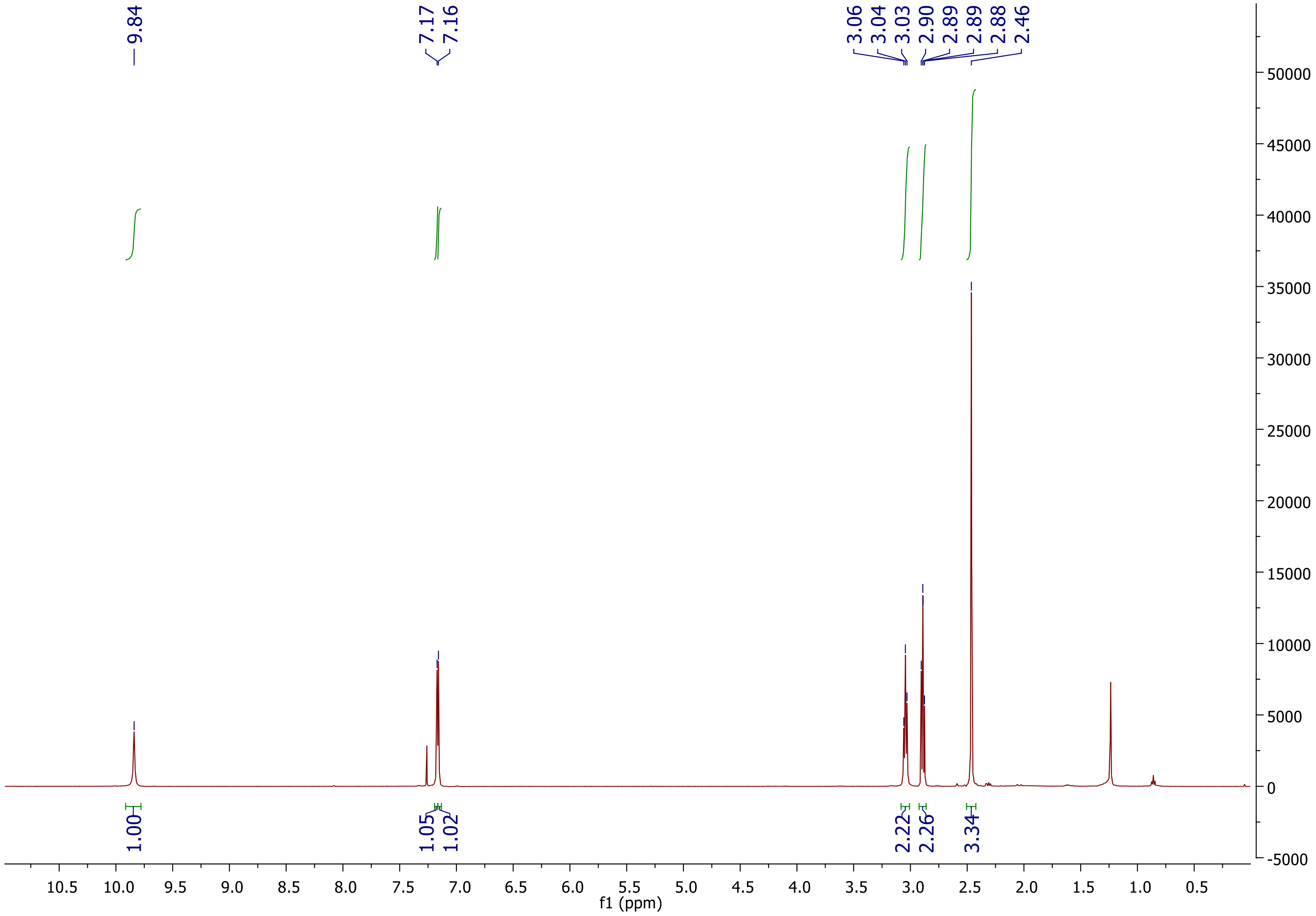

3-(4-Bromo-6-methylpyridin-2-yl)propanal S15

^{13}C-NMR (126 MHz, $CDCl_3$)

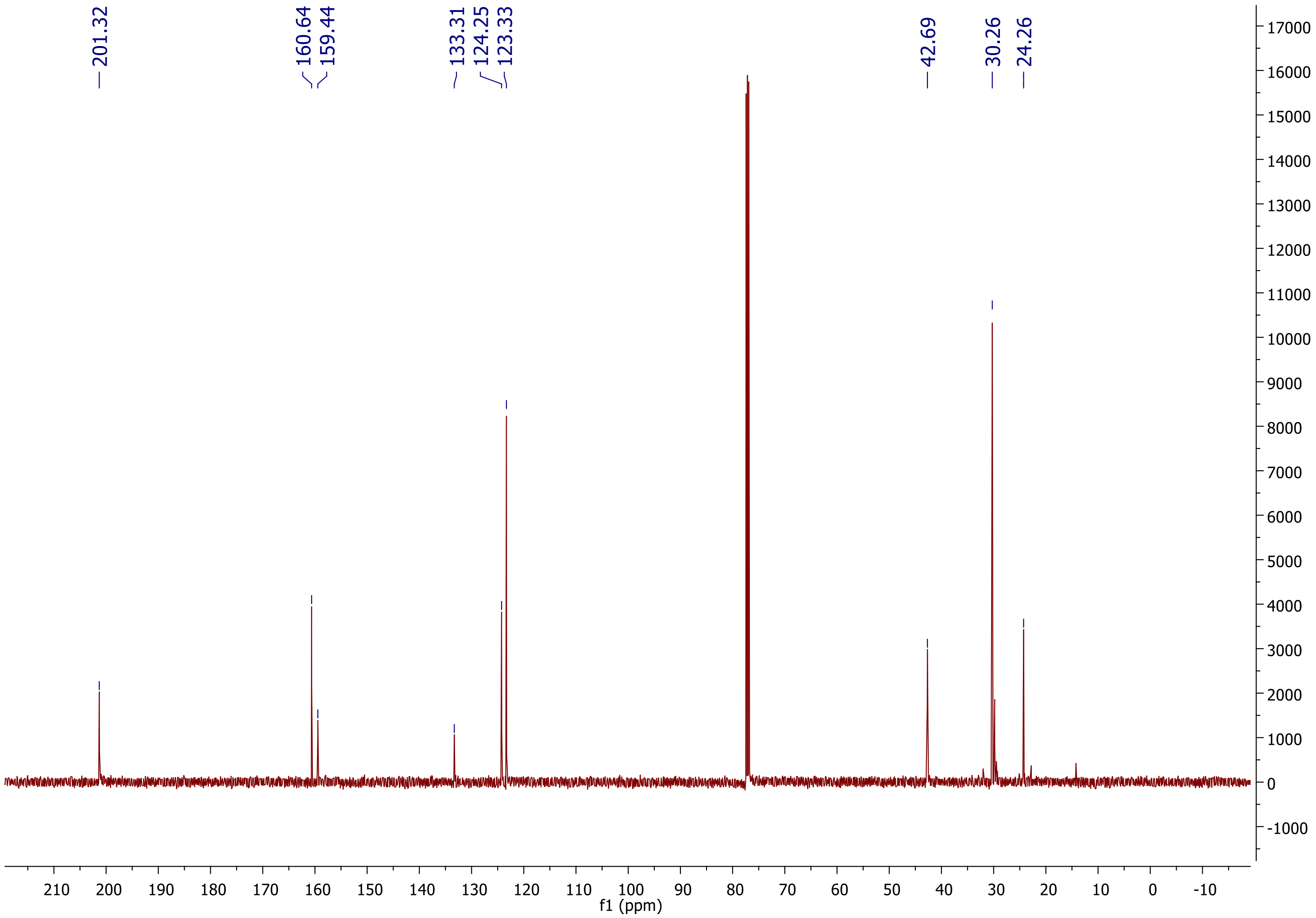

Diethyl 2,2'-{[3-(4-bromo-6-methylpyridin-2-yl)propyl]azanediyl}diacetate S16

^{1}H-NMR (500 MHz, $CDCl_3$).

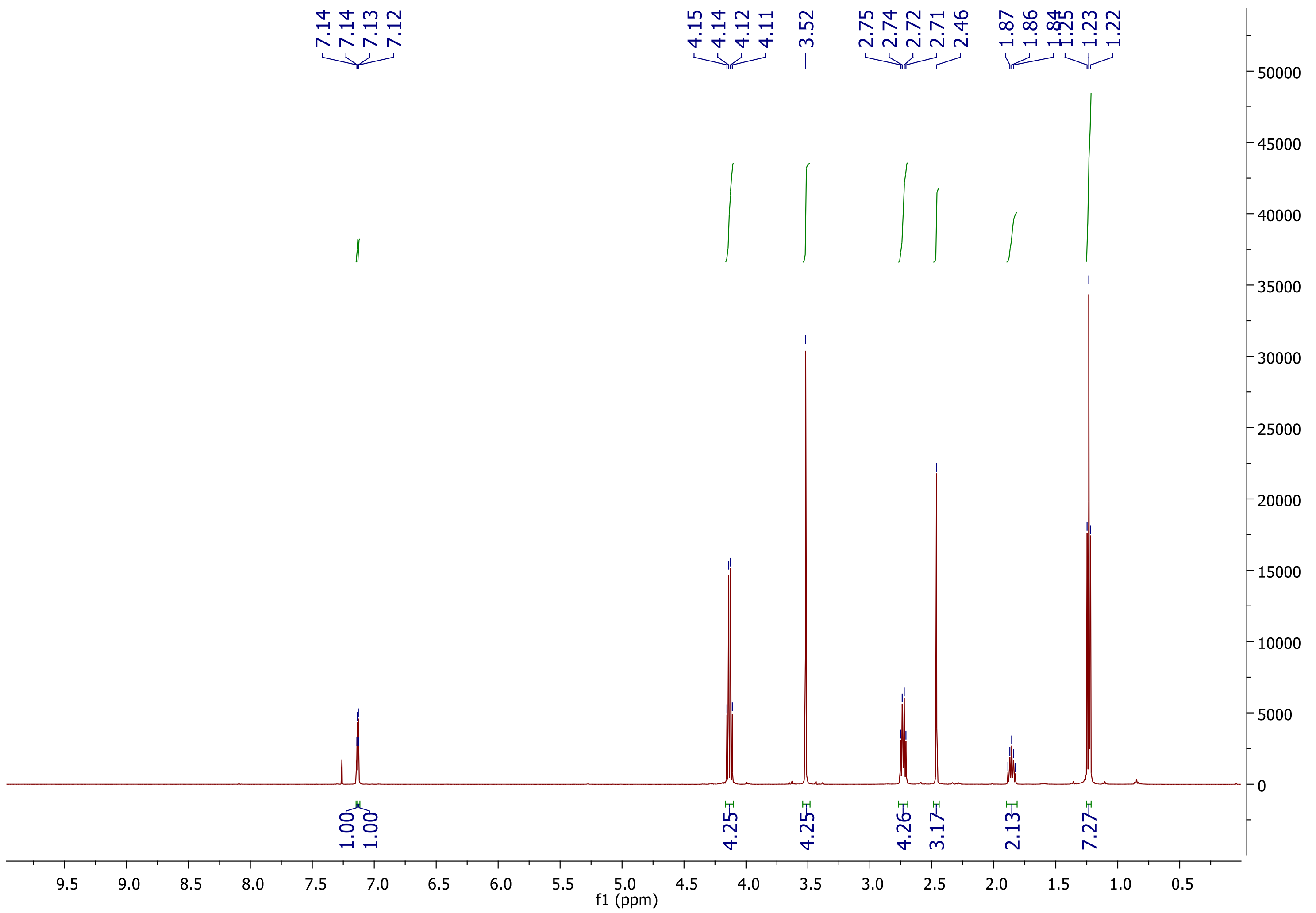

Diethyl 2,2'-{[3-(4-bromo-6-methylpyridin-2-yl)propyl]azanediyl}diacetate S16

^{13}C-NMR (126 MHz, $CDCl_3$)

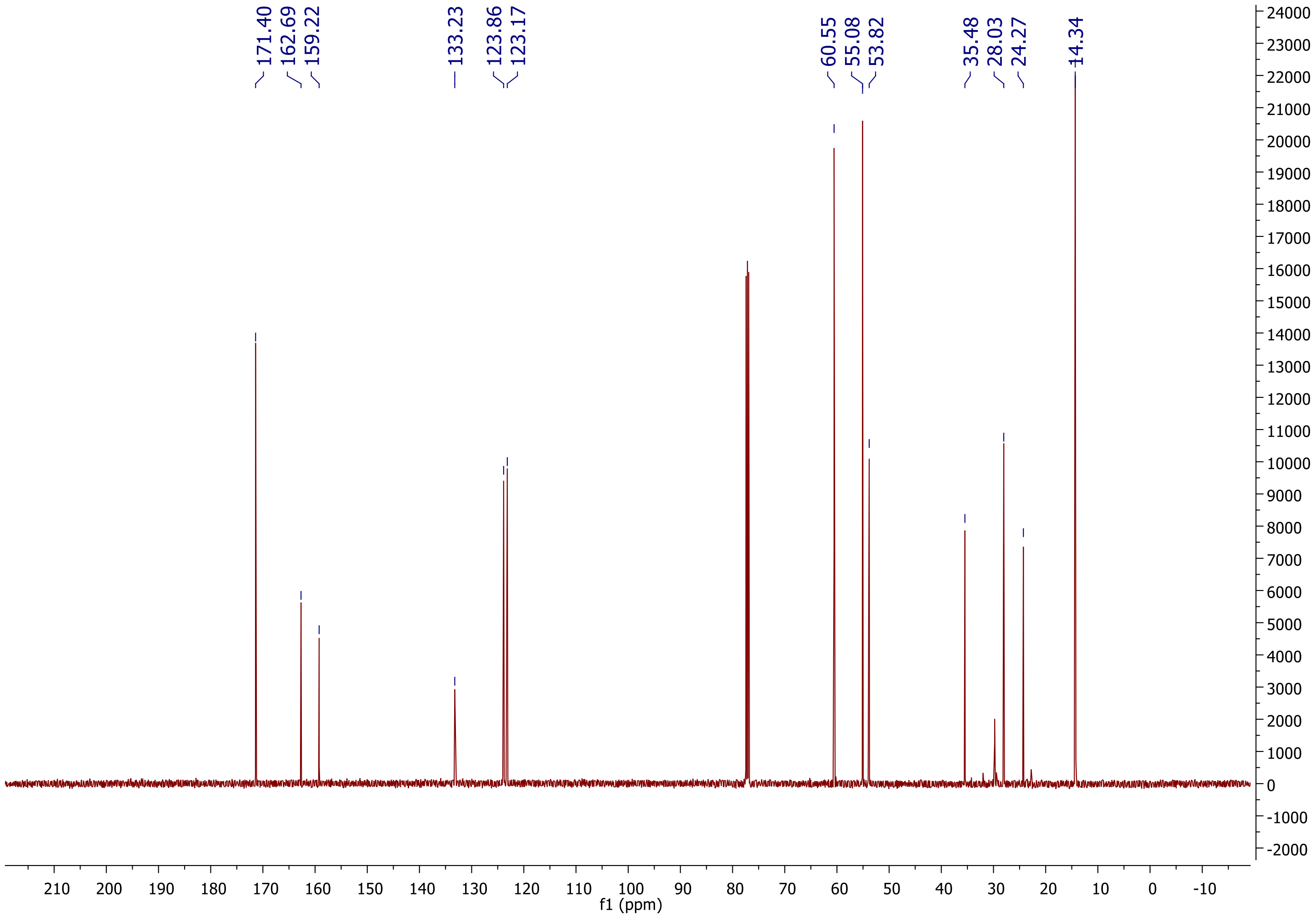

Diethyl 3,3'-(4-bromopyridine-2,6-diyl)dipropanoate S18

^{1}H-NMR (400 MHz, $CDCl_3$).

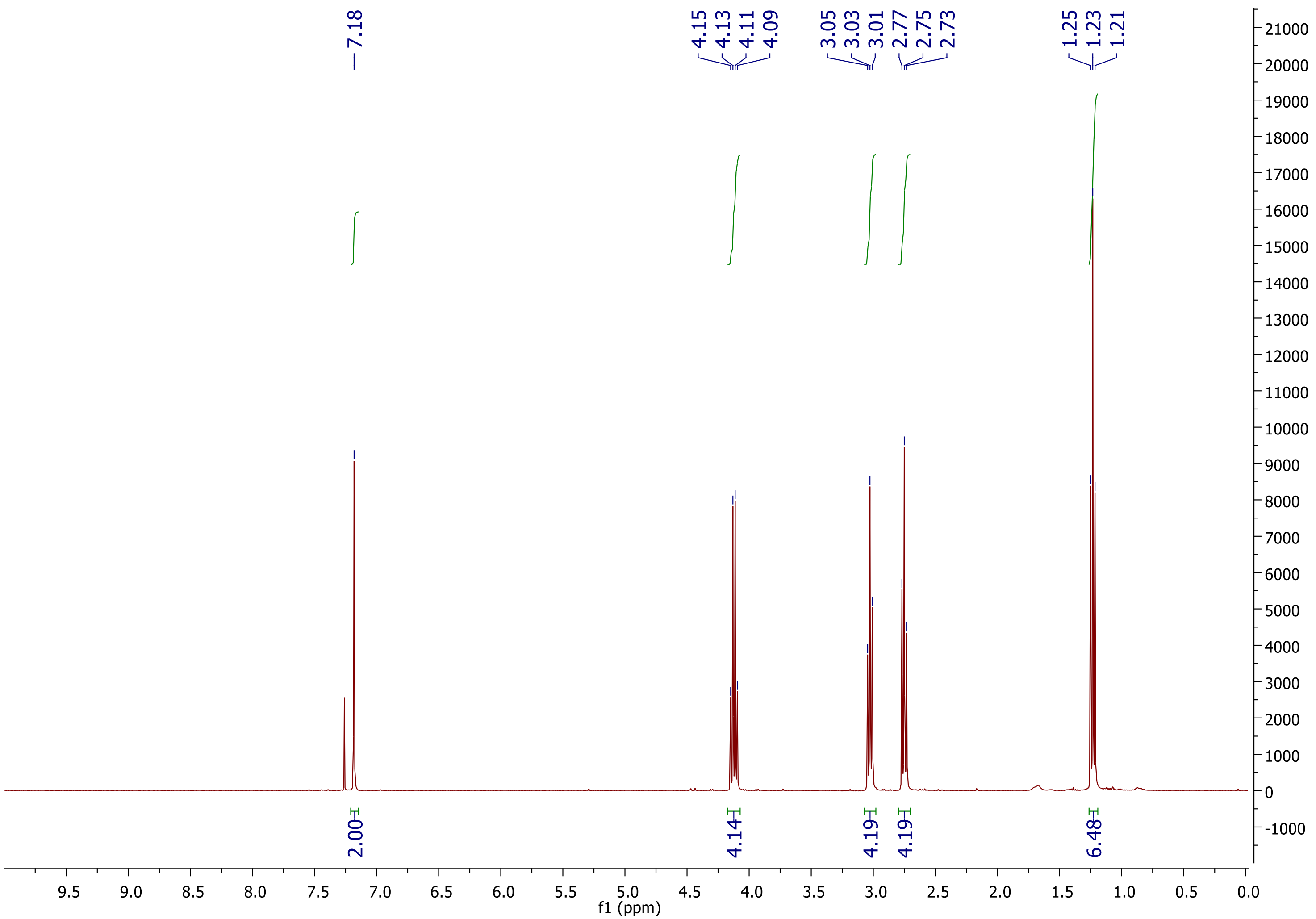

Diethyl 3,3'-(4-bromopyridine-2,6-diyl)dipropanoate S18

^{13}C-NMR (101 MHz, $CDCl_3$)

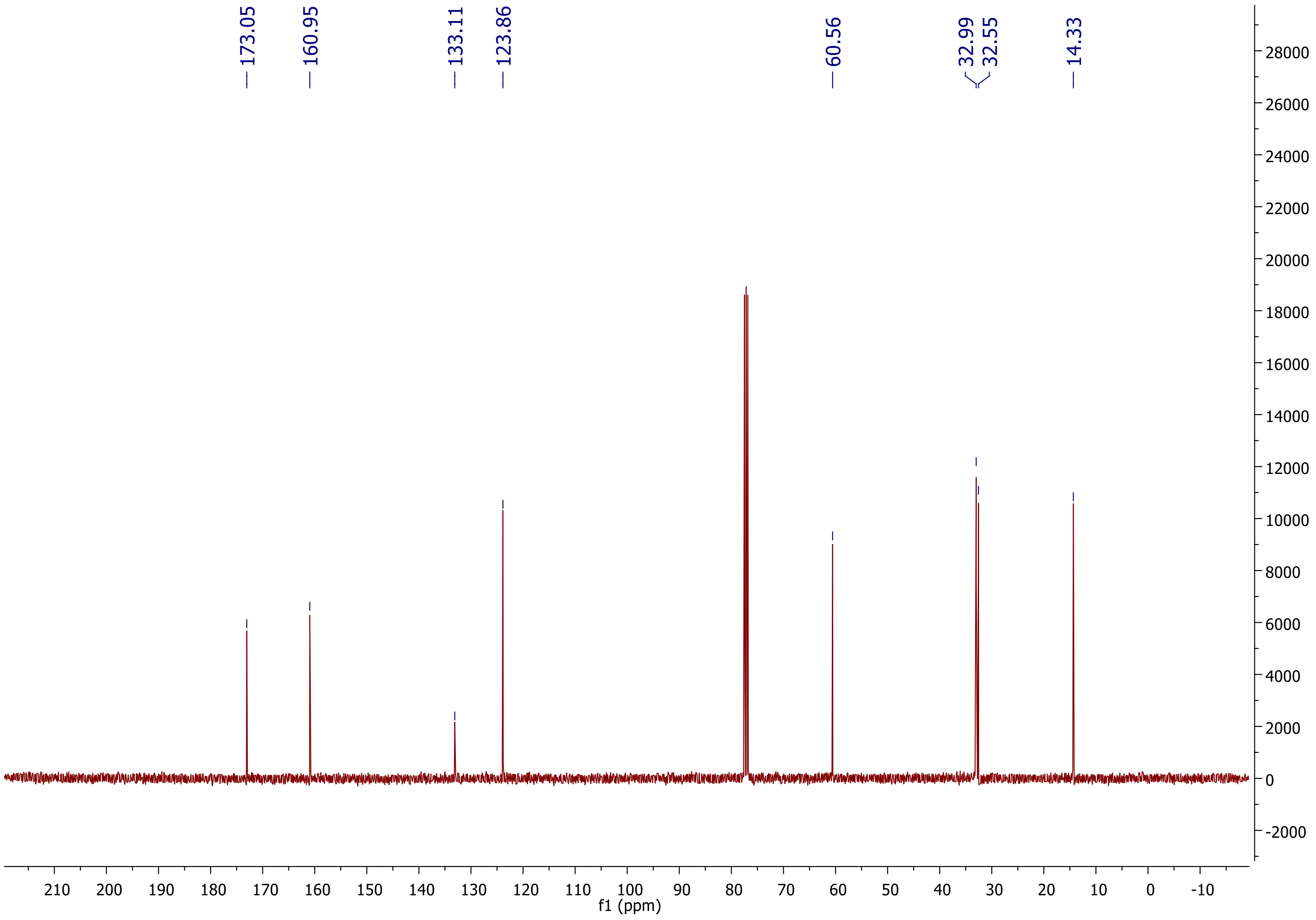

Ethyl 3-[4-bromo-6-(bromomethyl)pyridin-2-yl]propanoate S19

^{1}H-NMR (500 MHz, $CDCl_3$).

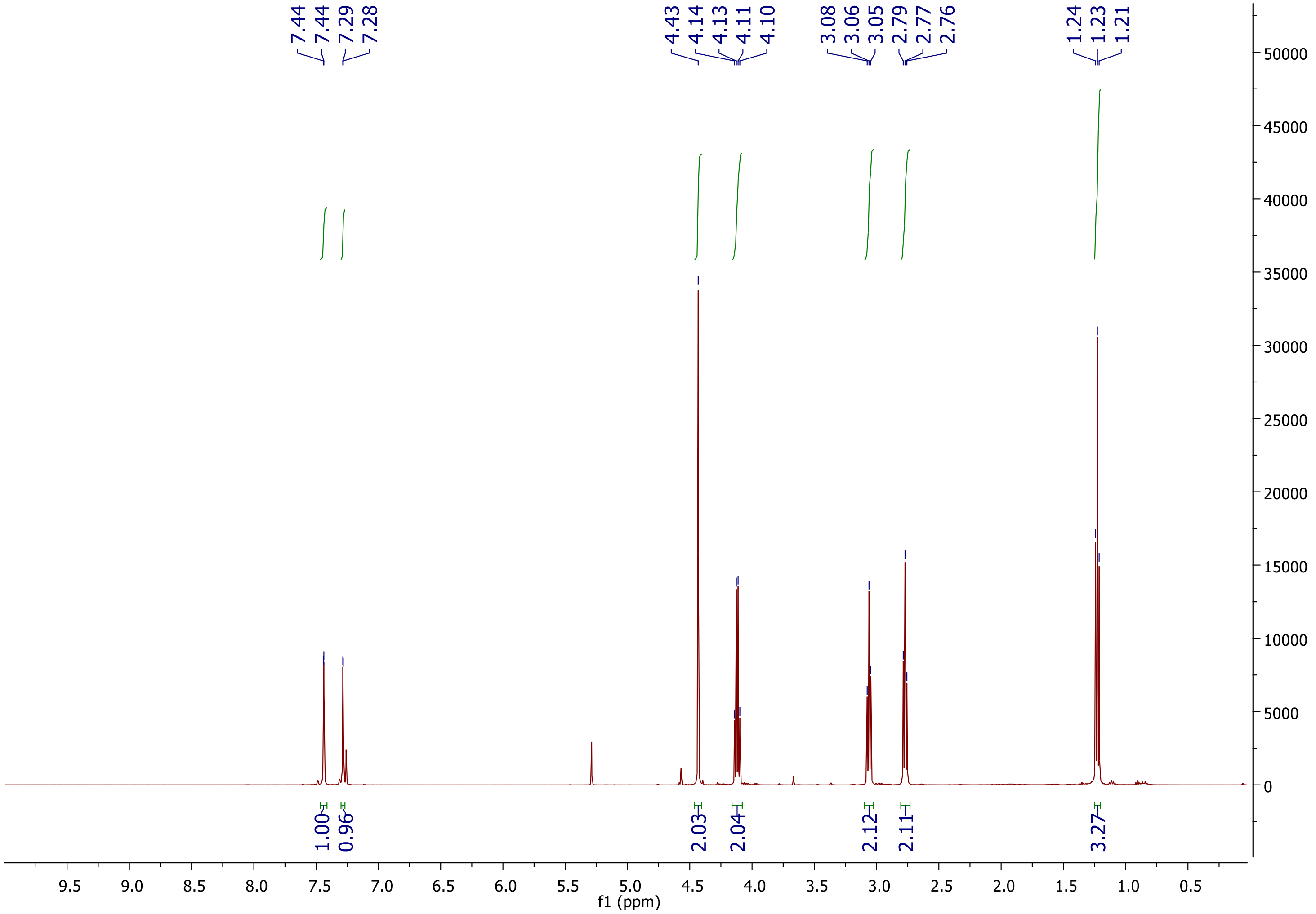

Ethyl 3-[4-bromo-6-(bromomethyl)pyridin-2-yl]propanoate S19

^{13}C-NMR (126 MHz, $CDCl_3$)

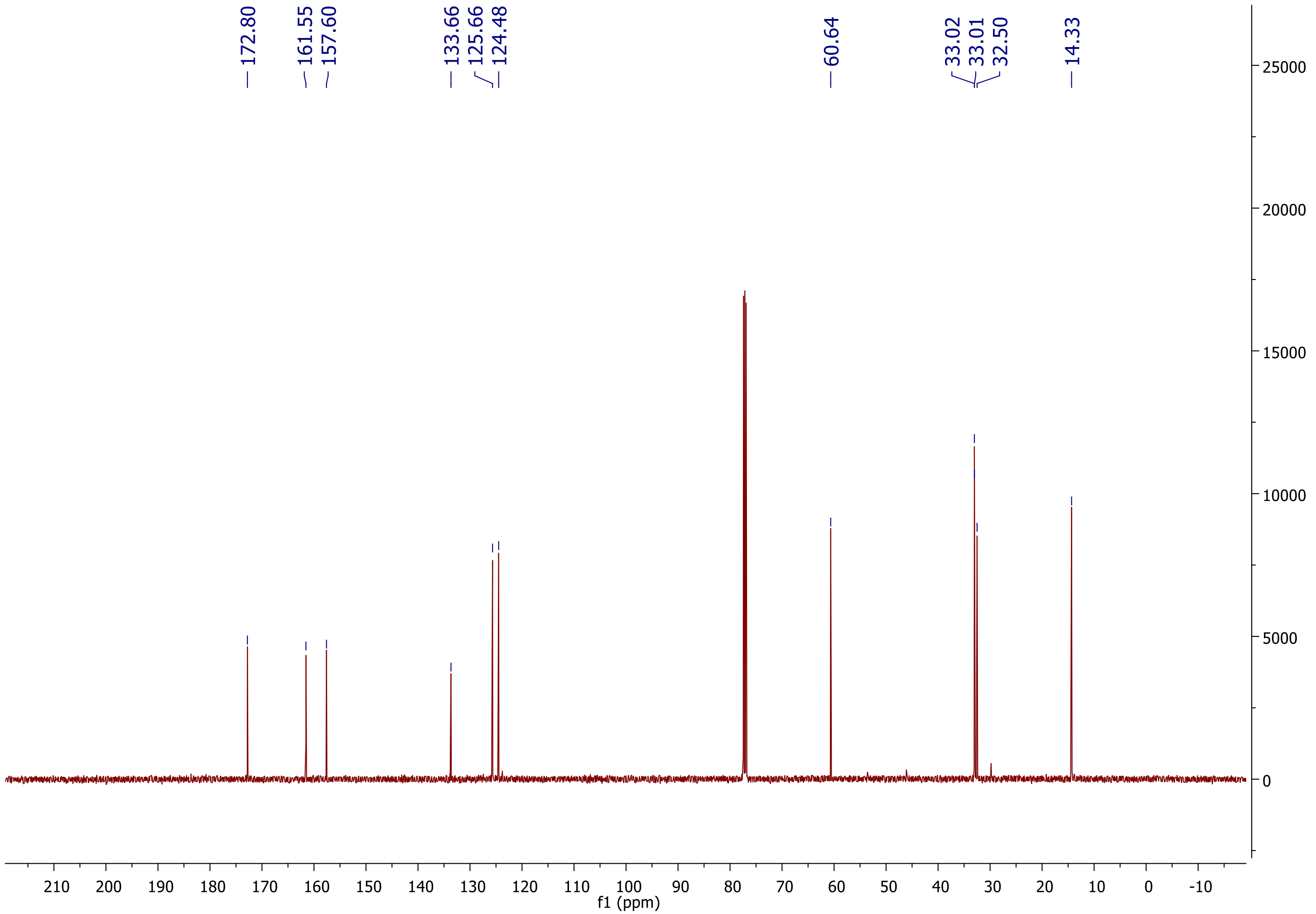

Ethyl 3-[6-(azidomethyl)-4-bromopyridin-2-yl]propanoate S20

^{1}H-NMR (500 MHz, $CDCl_3$).

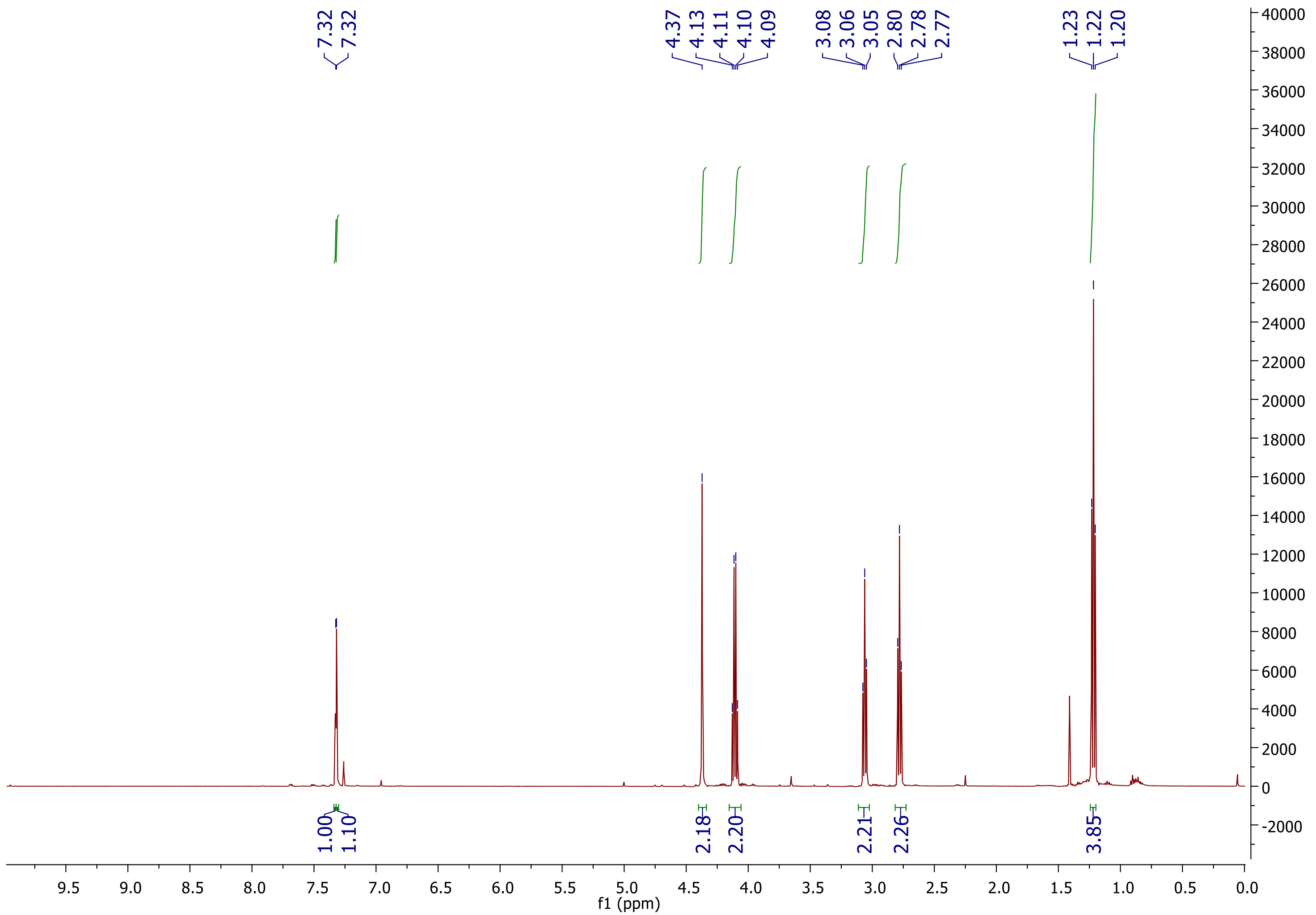

Ethyl 3-[6-(azidomethyl)-4-bromopyridin-2-yl]propanoate S20

^{13}C-NMR (126 MHz, $CDCl_3$)

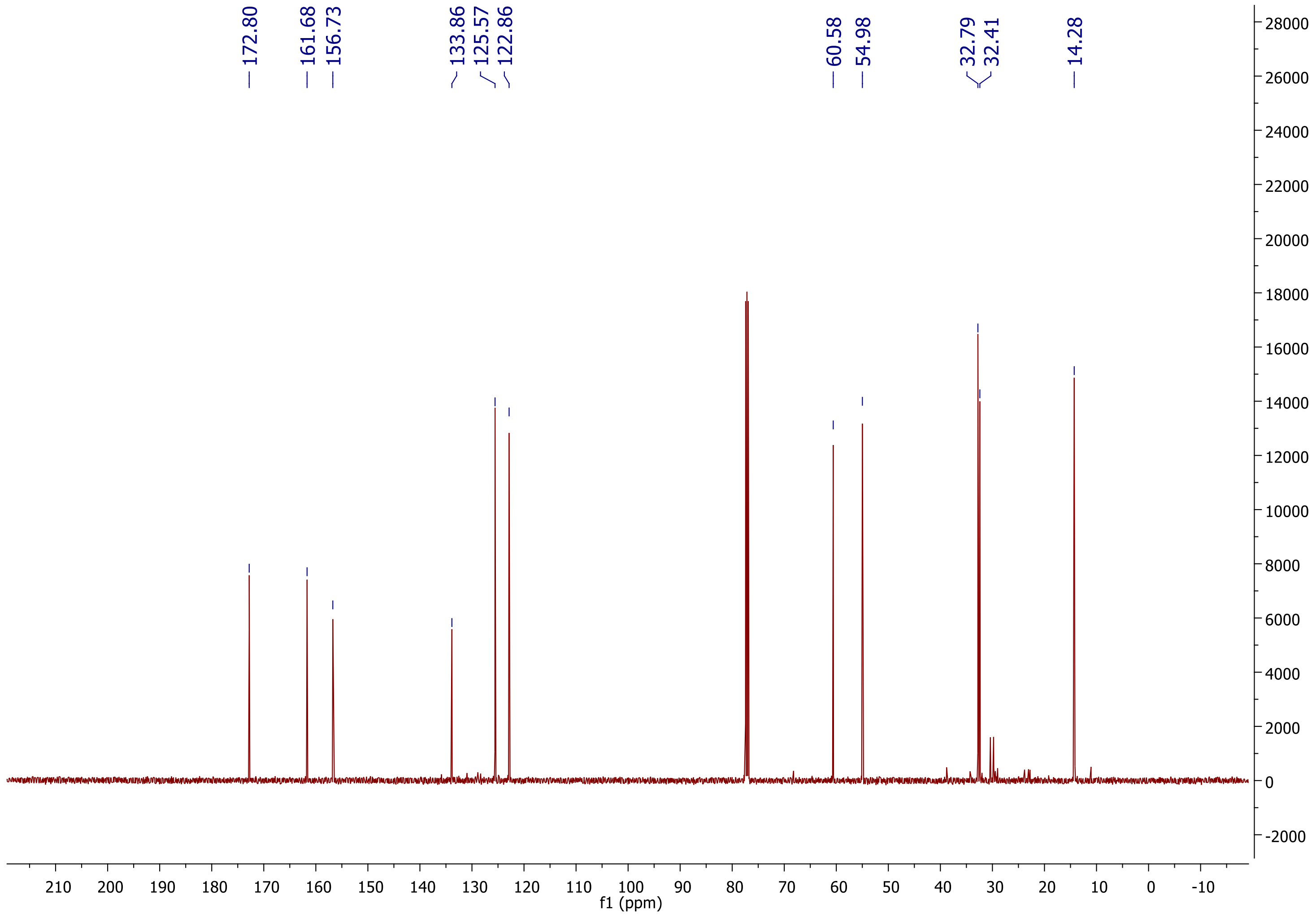

Ethyl 3-(4-bromo-6-{[4-(2-hydroxypropan-2-yl)-1H-1,2,3-triazol-1-yl]methyl}pyridin-2-yl)propanoate S21

^{1}H-NMR (400 MHz, $CDCl_3$).

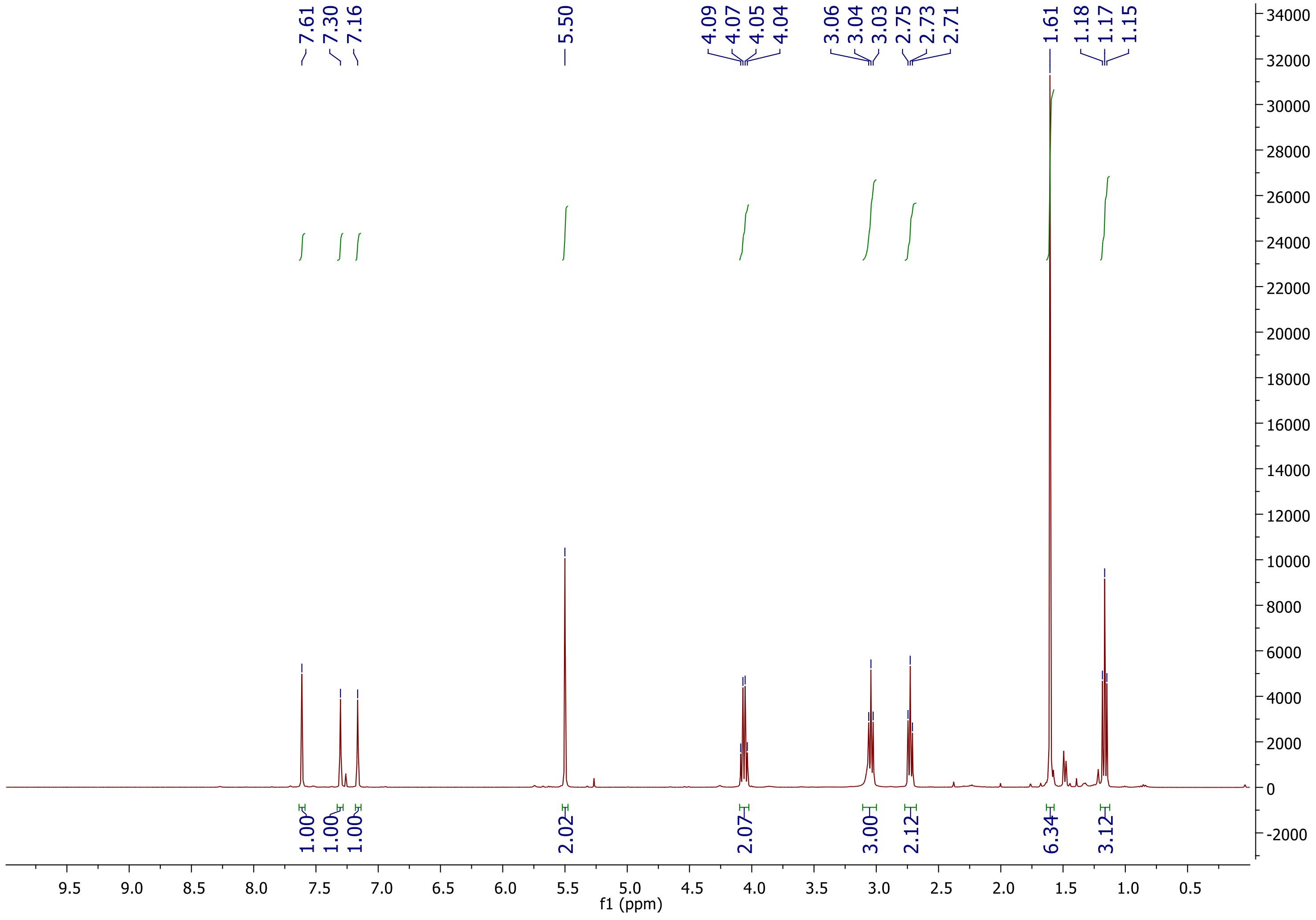

Ethyl 3-(4-bromo-6-{[4-(2-hydroxypropan-2-yl)-1H-1,2,3-triazol-1-yl]methyl}pyridin-2-yl)propanoate S21

^{13}C-NMR (101 MHz, $CDCl_3$)

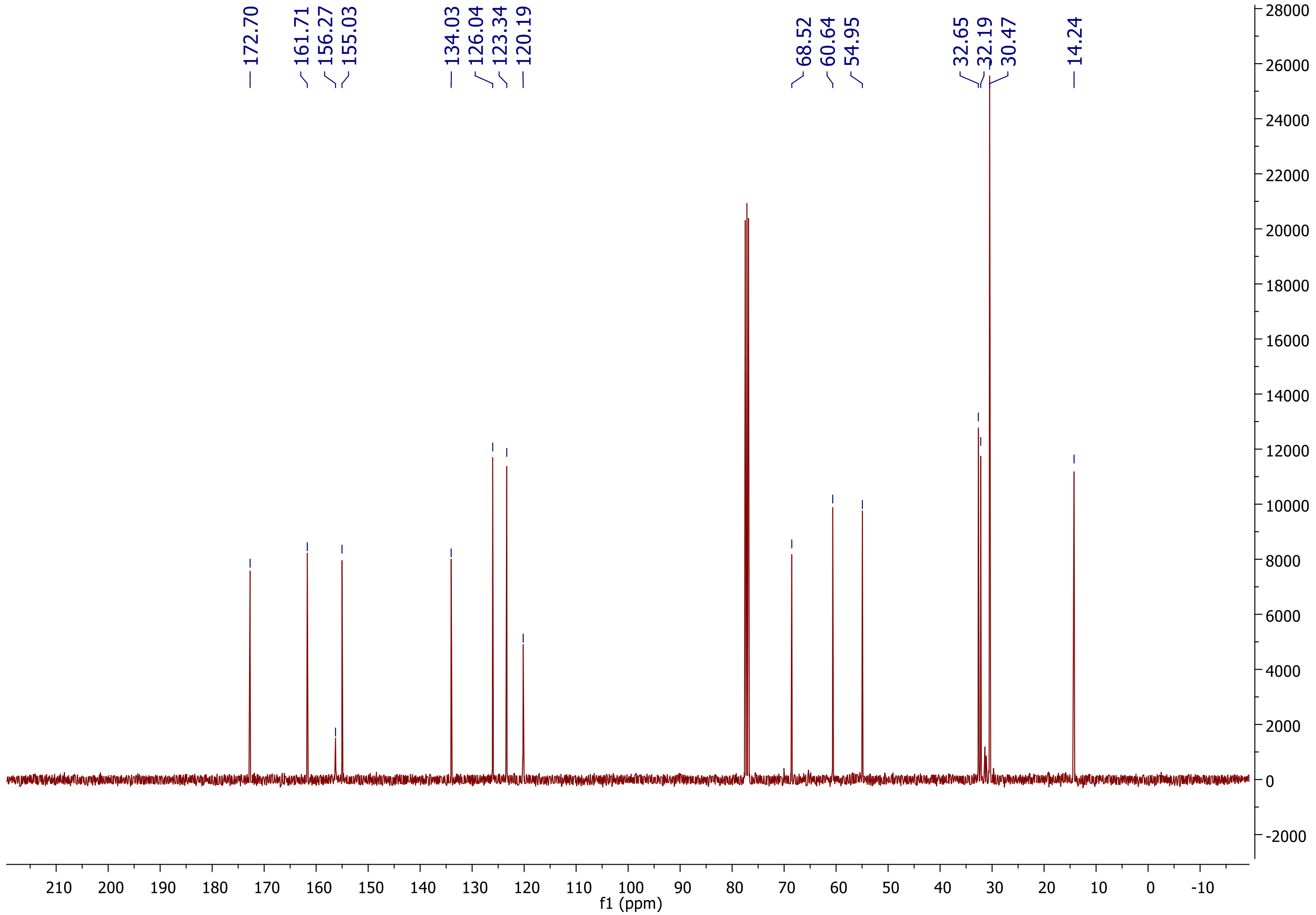

Ethyl 3-(4-bromo-6-{[(*t*-butoxycarbonyl)amino]methyl}pyridin-2-yl)propanoate S22

^{1}H-NMR (500 MHz, $CDCl_3$).

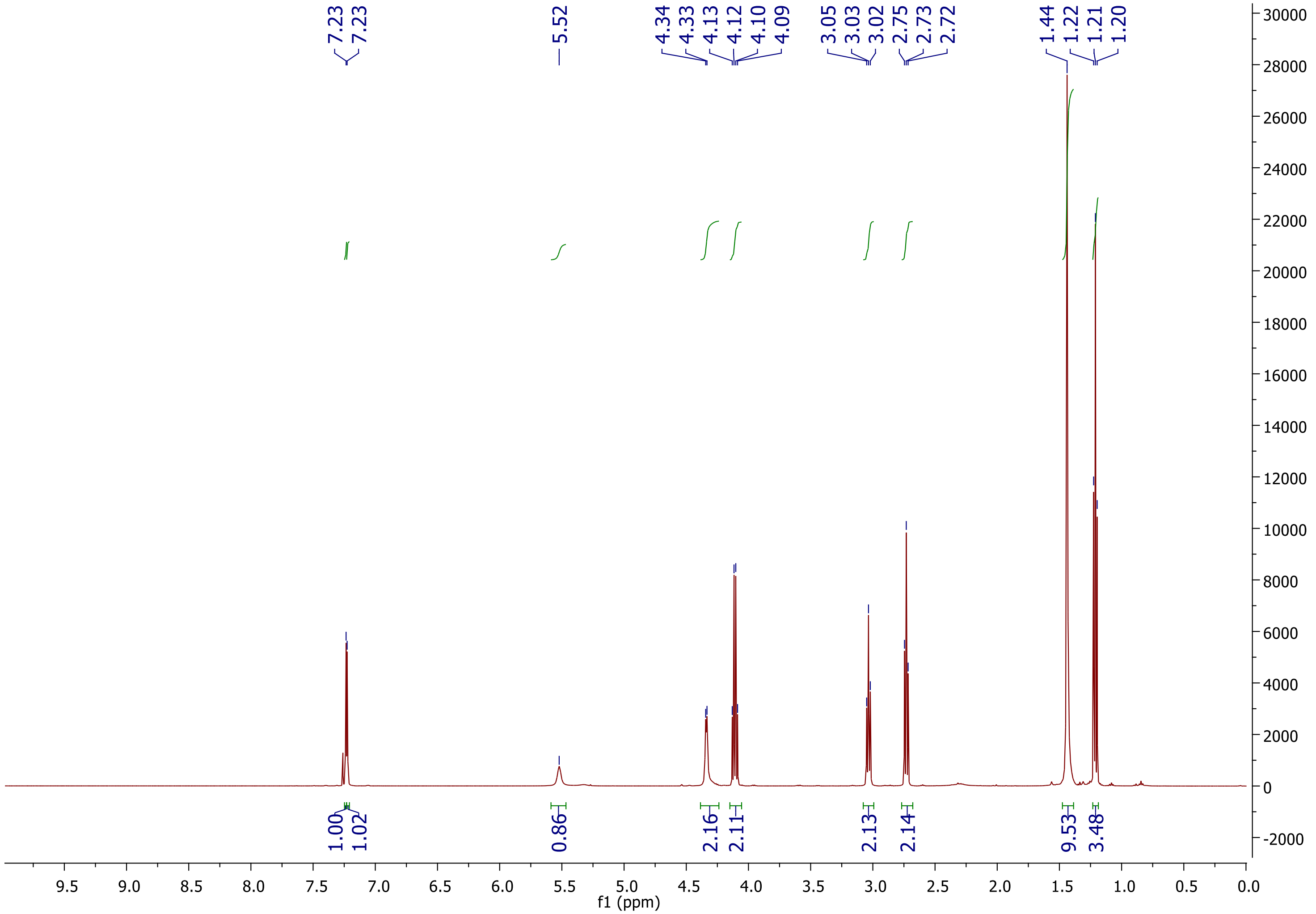

Ethyl 3-(4-bromo-6-{[(*t*-butoxycarbonyl)amino]methyl}pyridin-2-yl)propanoate S22

^{13}C-NMR (126 MHz, $CDCl_3$)

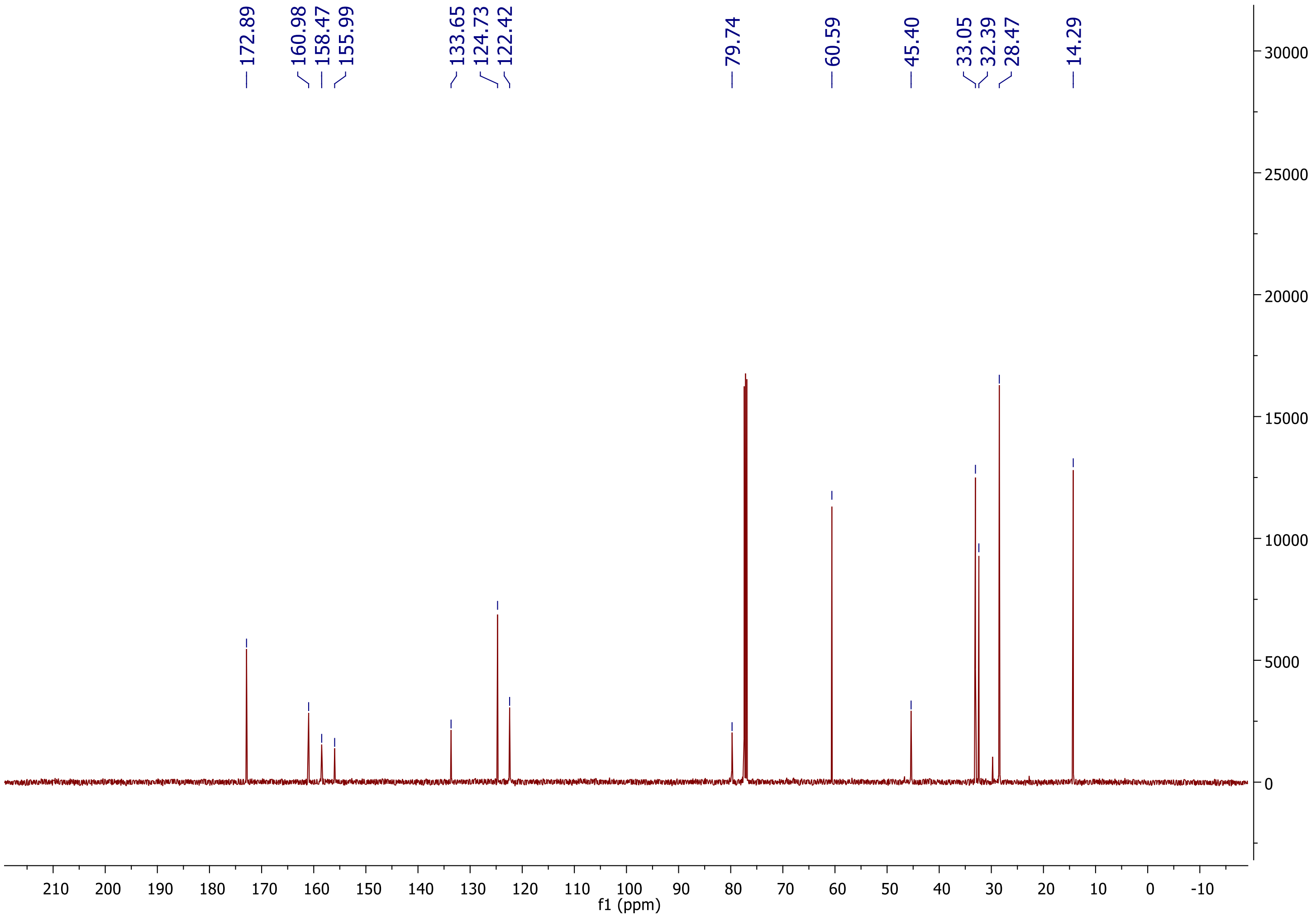

2,6-Bis(azidomethyl)-4-bromopyridine 23

^{1}H-NMR (400 MHz, $CDCl_3$).

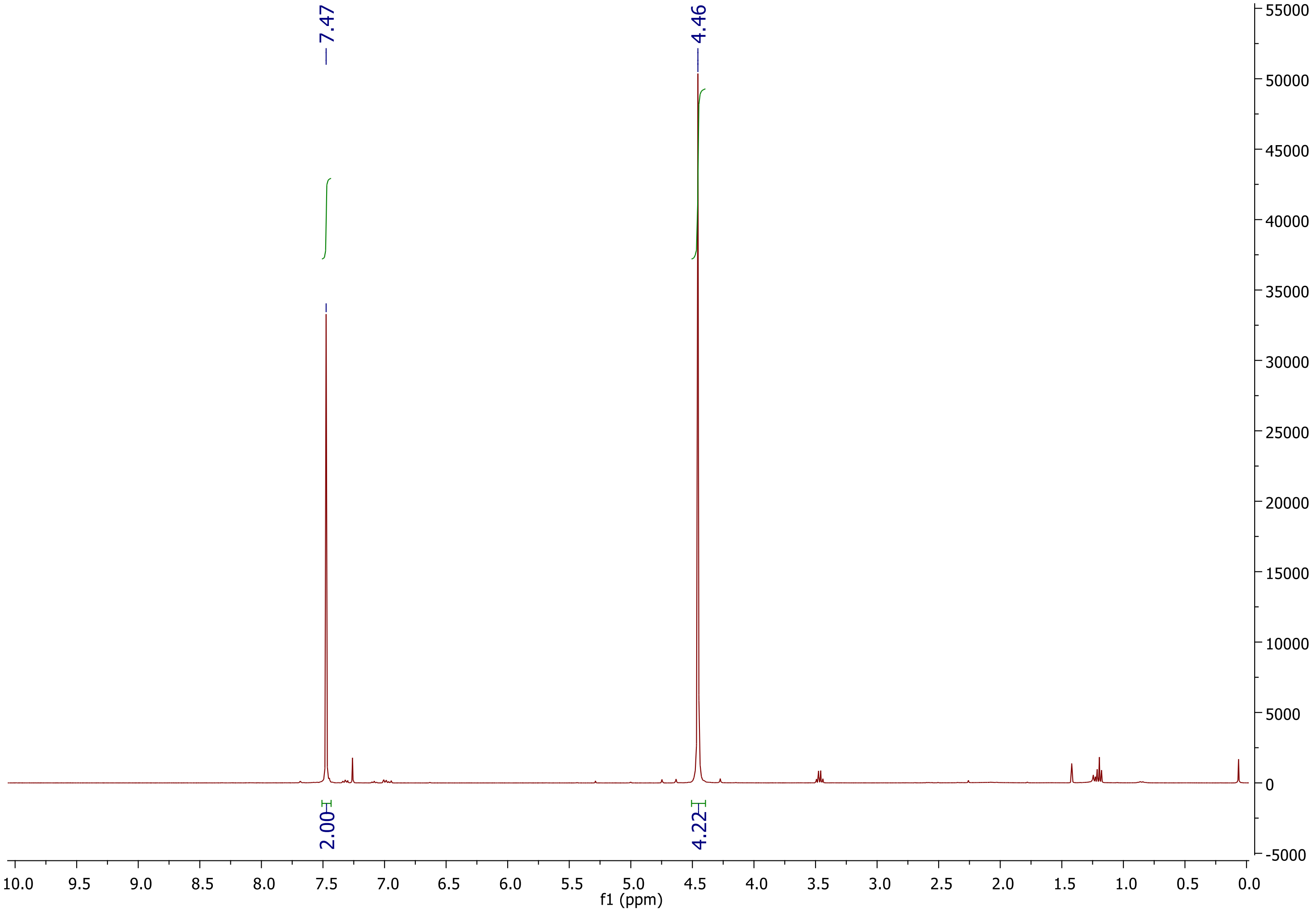

2,6-Bis(azidomethyl)-4-bromopyridine 23

^{13}C-NMR (101 MHz, $CDCl_3$)

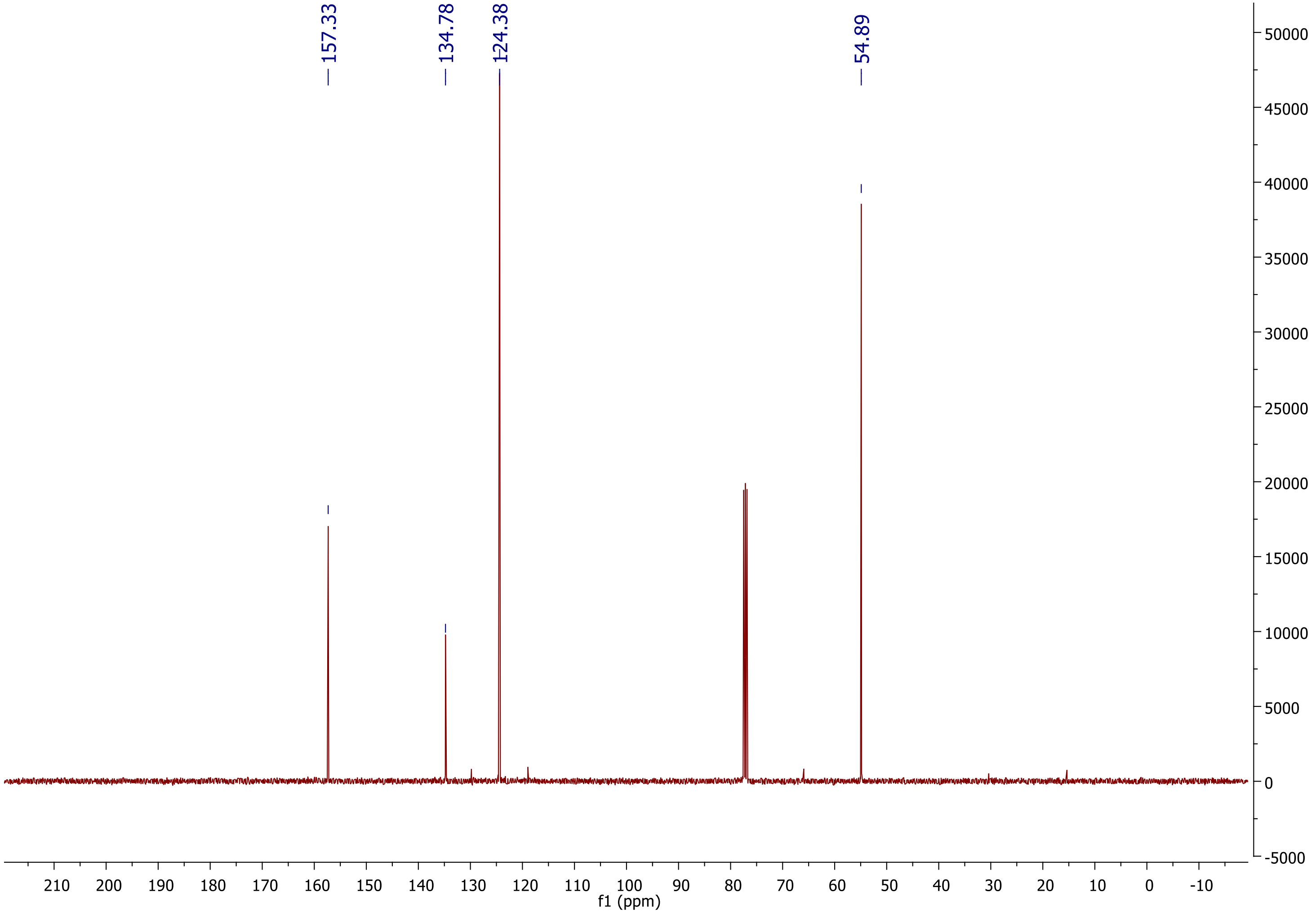

Di-*t*-butyl [(4-bromopyridine-2,6-diyl)bis(methylene)]biscarbamate S24

^{1}H-NMR (500 MHz, $CDCl_3$).

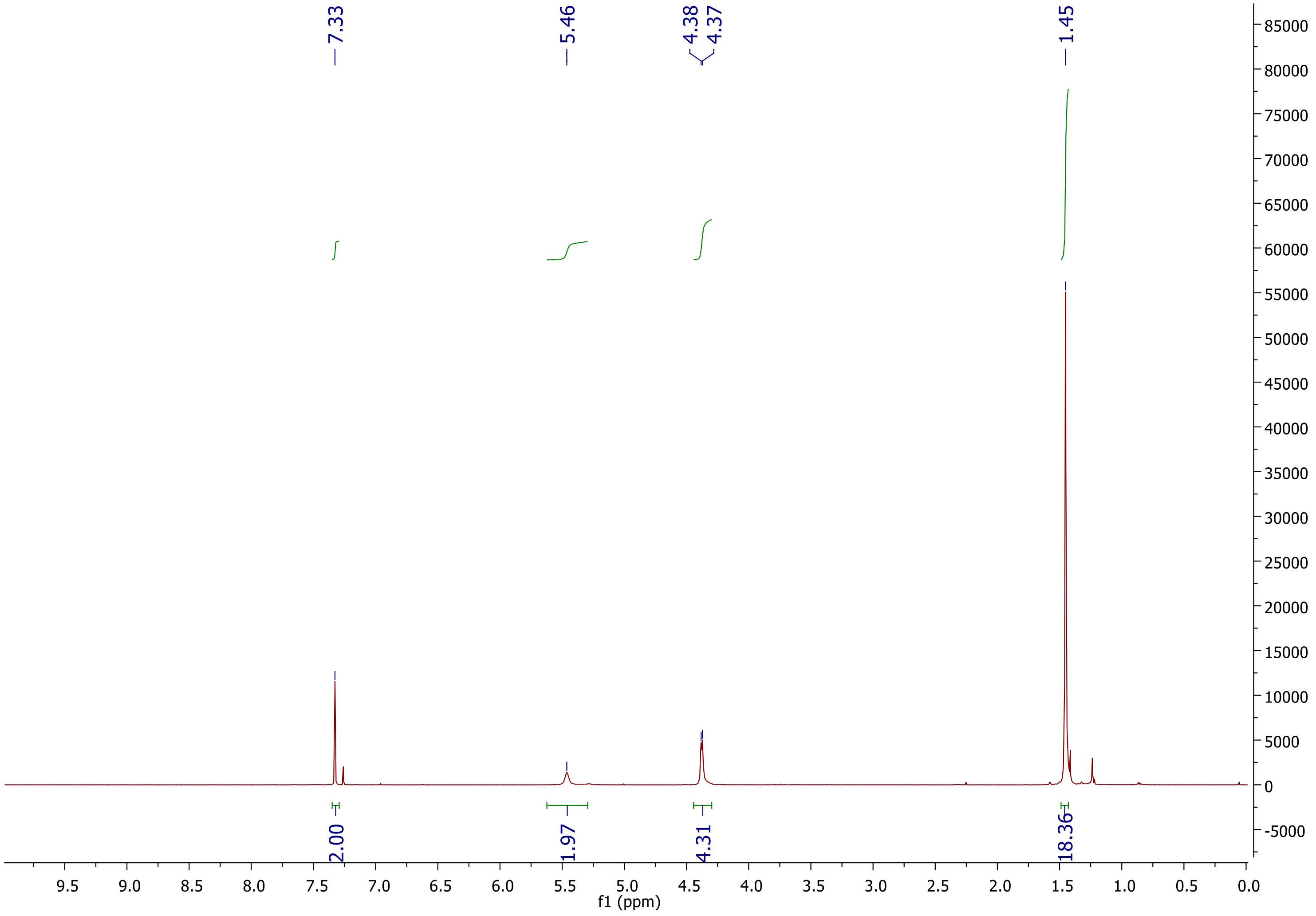

Di-*t*-butyl [(4-bromopyridine-2,6-diyl)bis(methylene)]biscarbamate S24

^{13}C-NMR (126 MHz, $CDCl_3$)

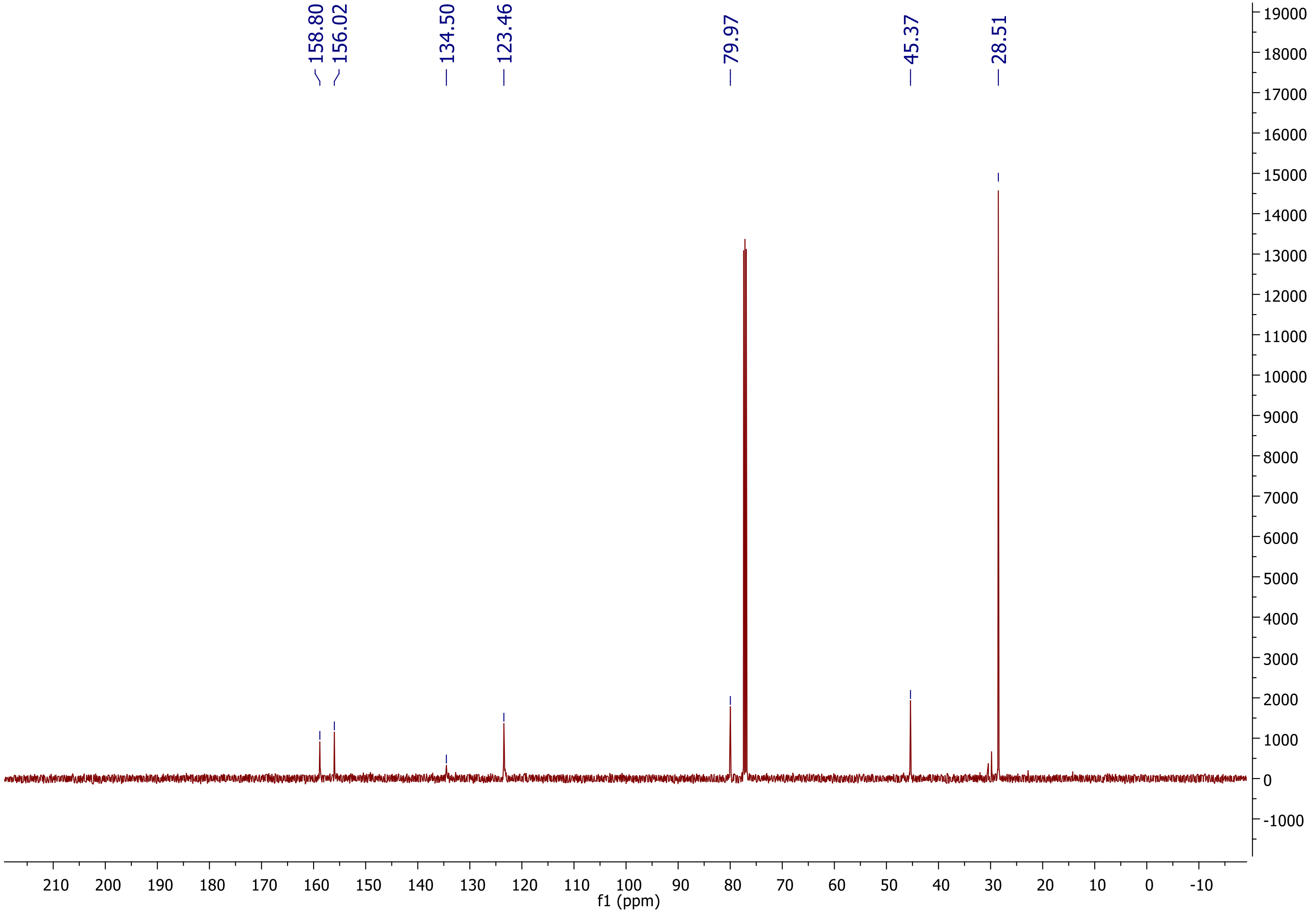

***t*-Butyl {[6-(azidomethyl)-4-bromopyridin-2-yl]methyl}carbamate S25**

^{1}H-NMR (500 MHz, $CDCl_3$).

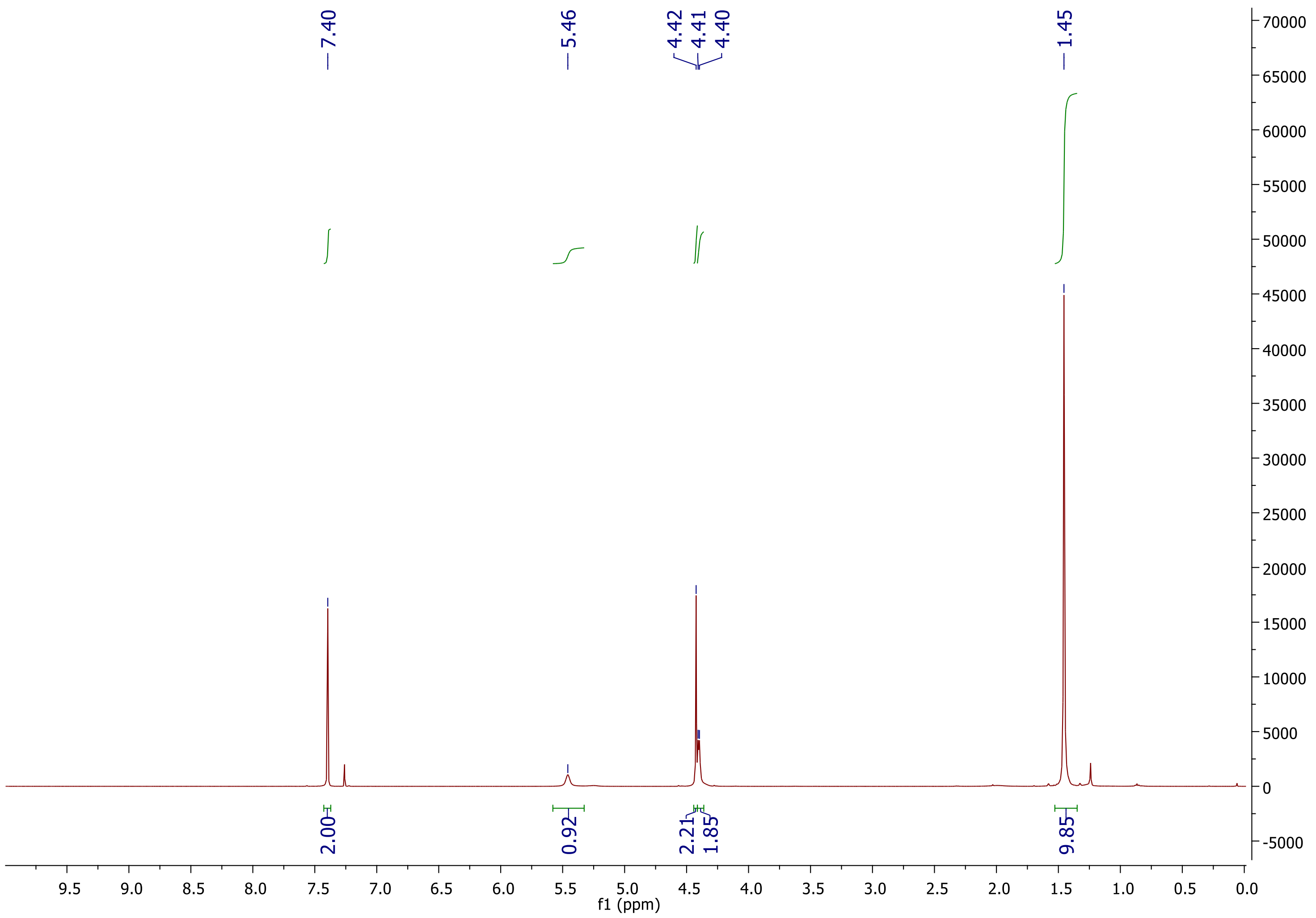

t-Butyl {[6-(azidomethyl)-4-bromopyridin-2-yl]methyl}carbamate S25

^{13}C-NMR (126 MHz, $CDCl_3$)

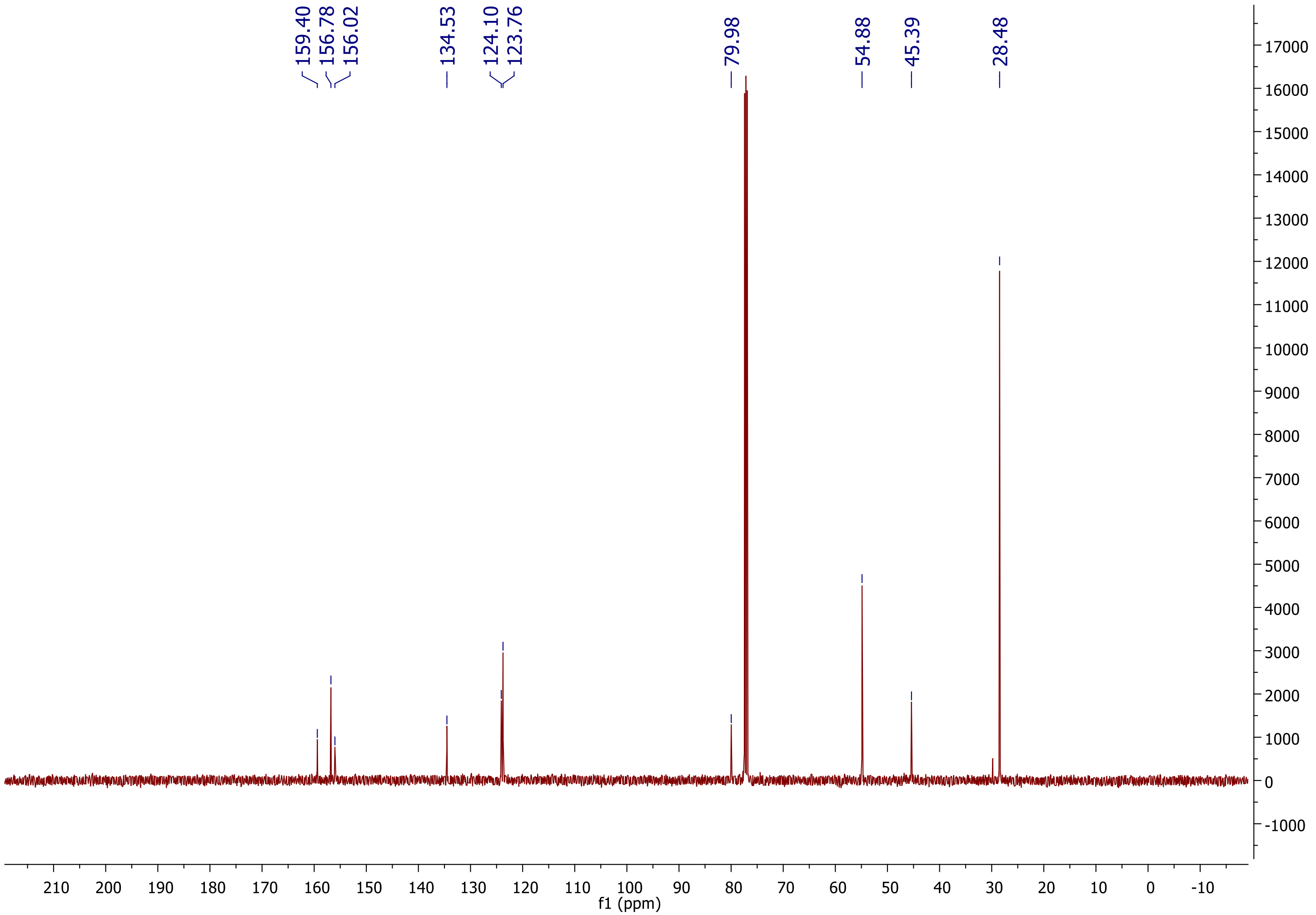

_t_-Butyl [(4-bromo-6-{[4-(2-hydroxypropan-2-yl)-1H-1,2,3-triazol-1-yl]methyl}pyridin-2-yl)methyl]carbamate S26

^{1}H-NMR (500 MHz, $CDCl_3$).

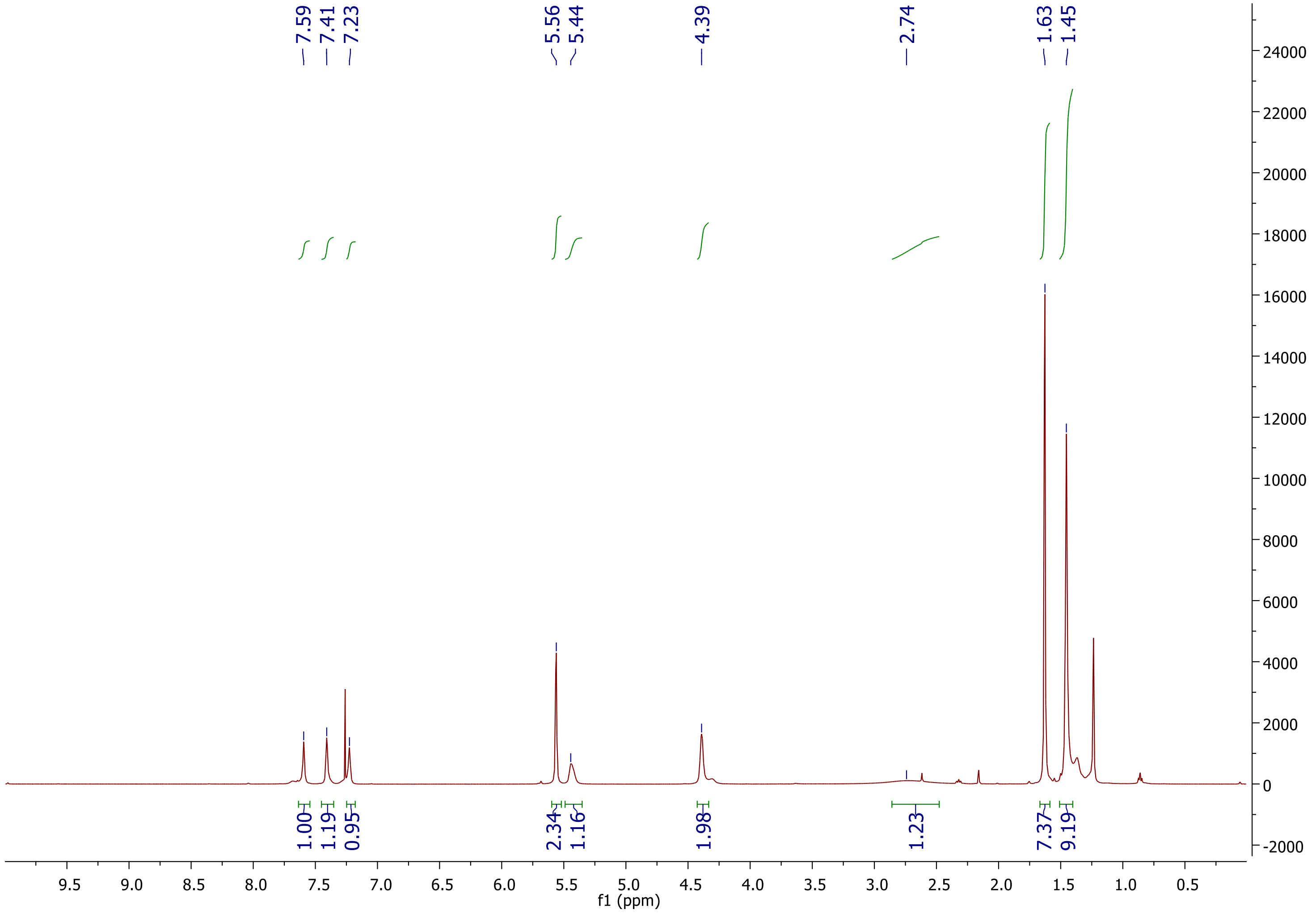

***t*-Butyl [(4-bromo-6-{[4-(2-hydroxypropan-2-yl)-1H-1,2,3-triazol-1-yl]methyl}pyridin-2-yl)methyl]carbamate S26**

^{13}C-NMR (126 MHz, $CDCl_3$)

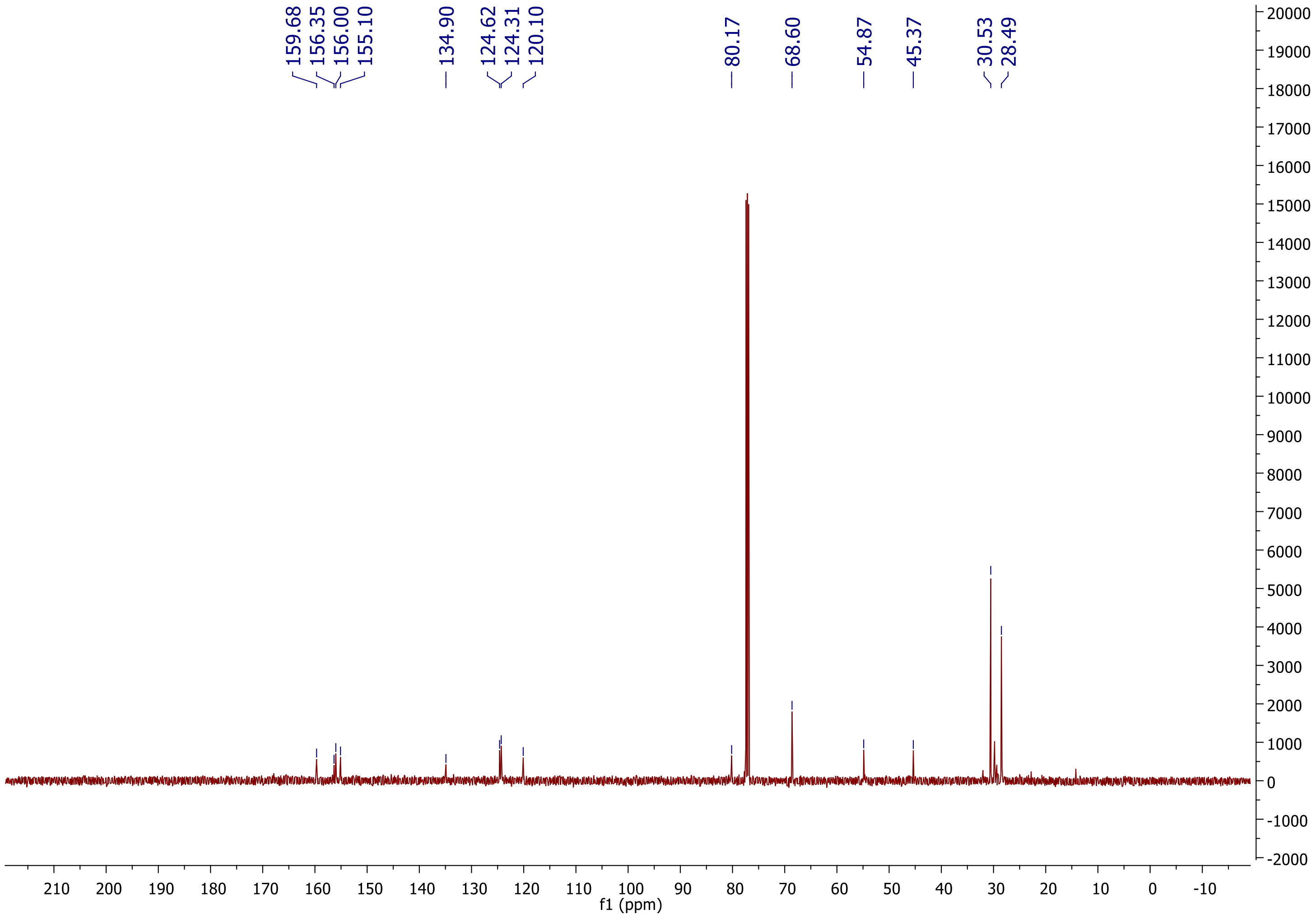

2,2'-[(4-Bromopyridine-2,6-diyl)bis(methylene-1H-1,2,3-triazole-1,4-diyl)]di(propan-2-ol) S27

^{1}H-NMR (500 MHz, $CDCl_3$).

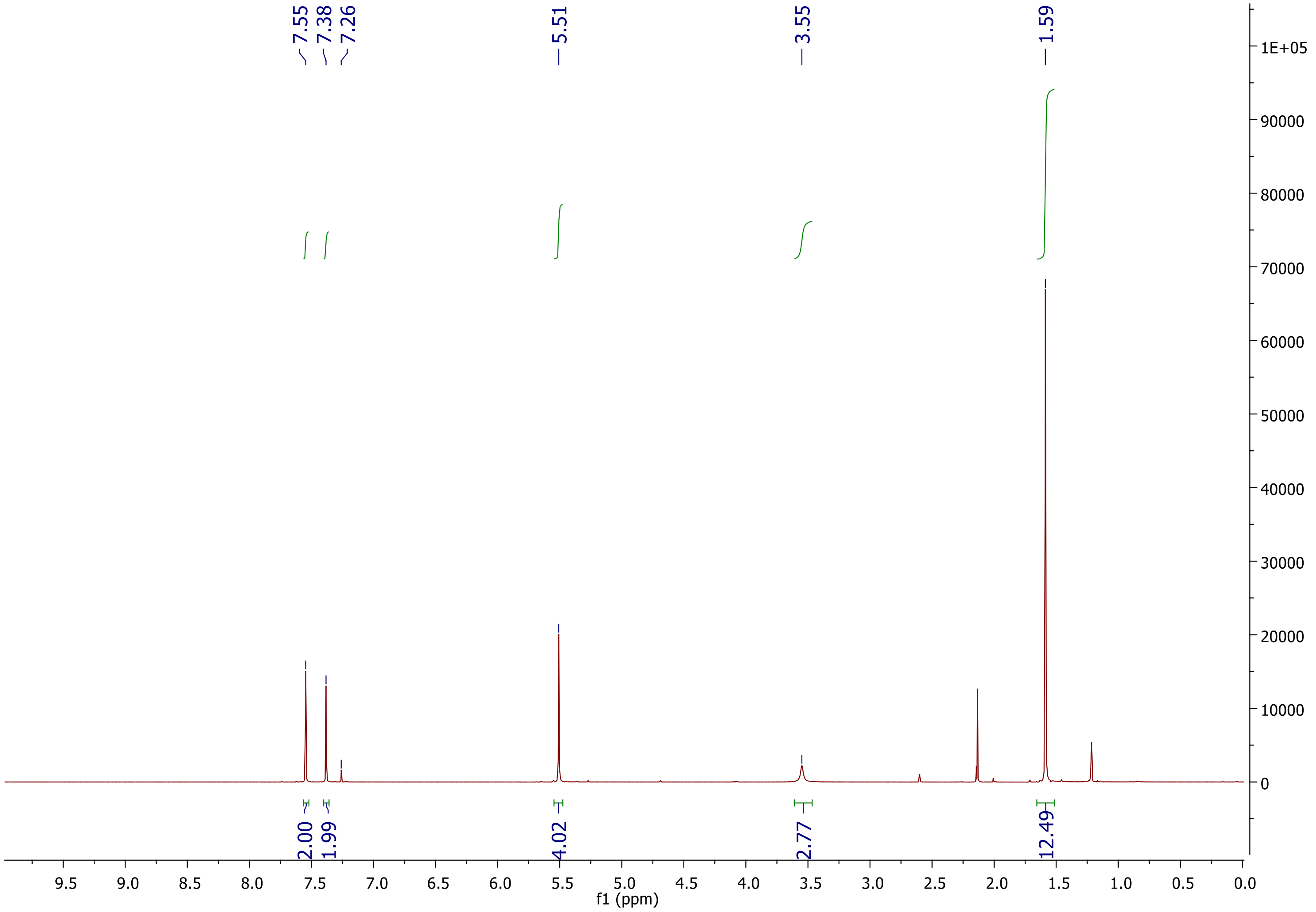

2,2'-[(4-Bromopyridine-2,6-diyl)bis(methylene-1H-1,2,3-triazole-1,4-diyl)]di(propan-2-ol) S27

^{13}C-NMR (126 MHz, $CDCl_3$)

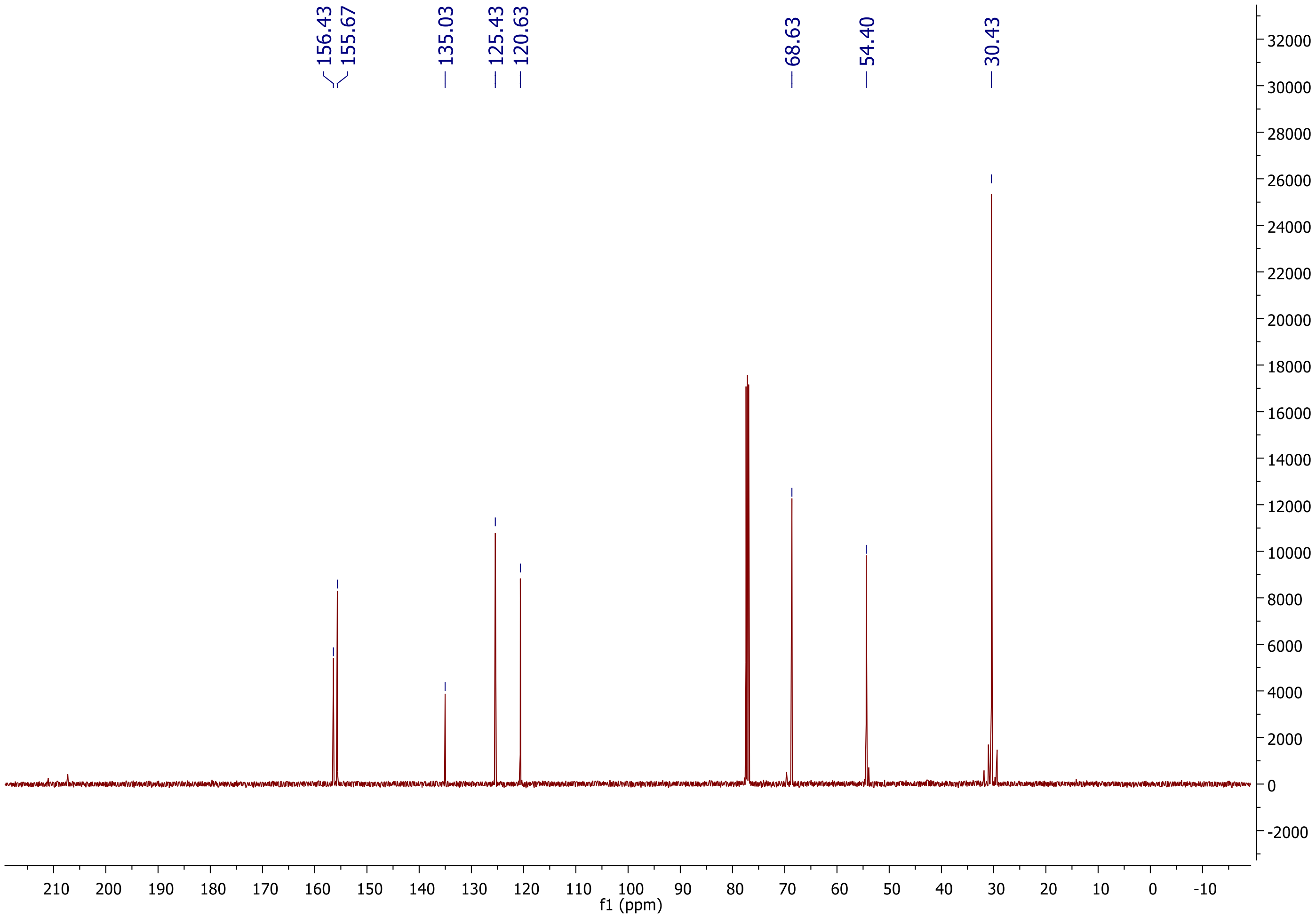

2-(Pyrrolidin-1-yl)-4-iodopyridine S30

^{1}H-NMR (500 MHz, $CDCl_3$).

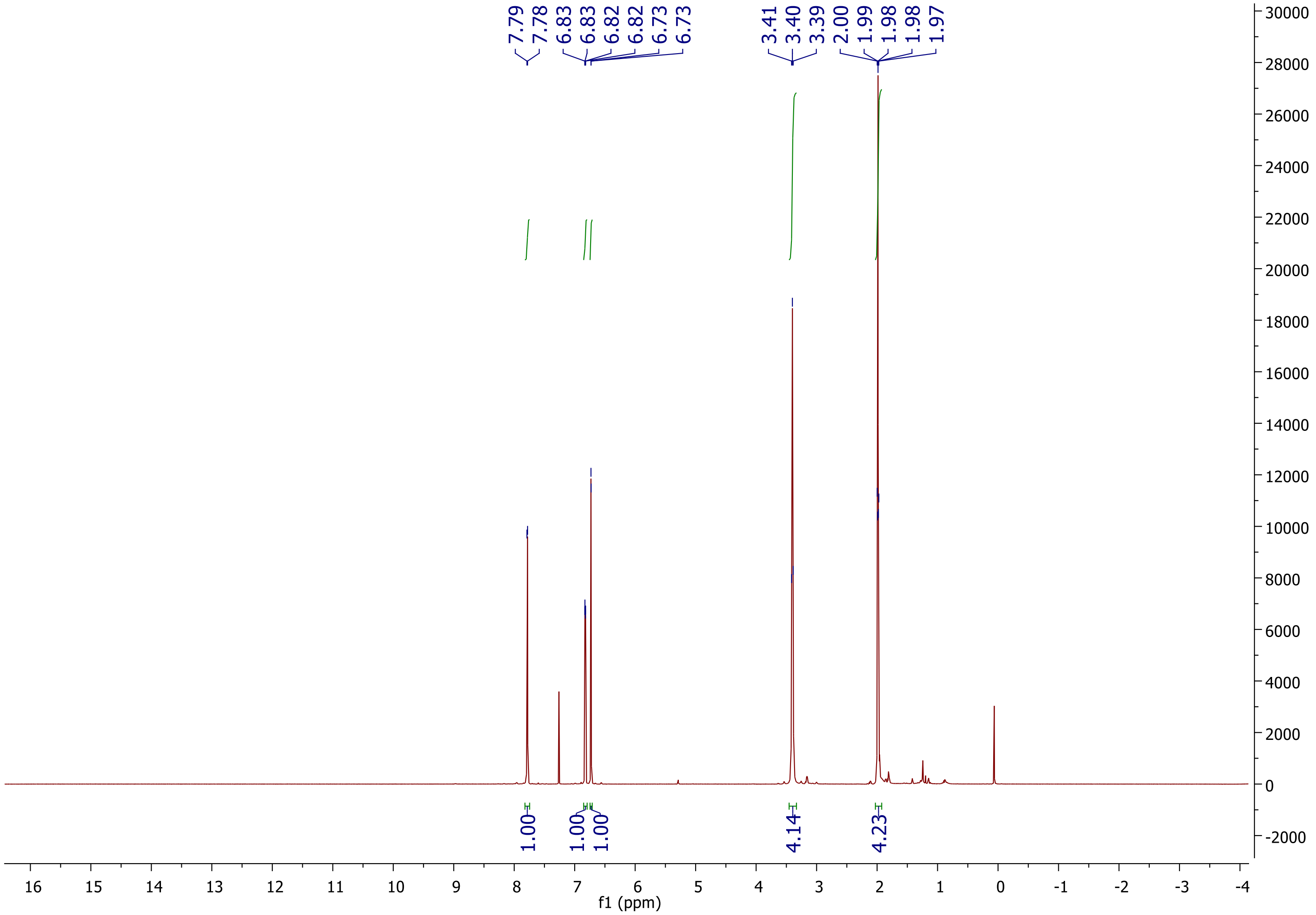

Methyl (*S*)-2-((*t*-butoxycarbonyl)amino)-3-(4-((4-iodopyridin-2-yl)oxy)phenyl)propanoate S30

^{1}H-NMR (500 MHz, $CDCl_3$).

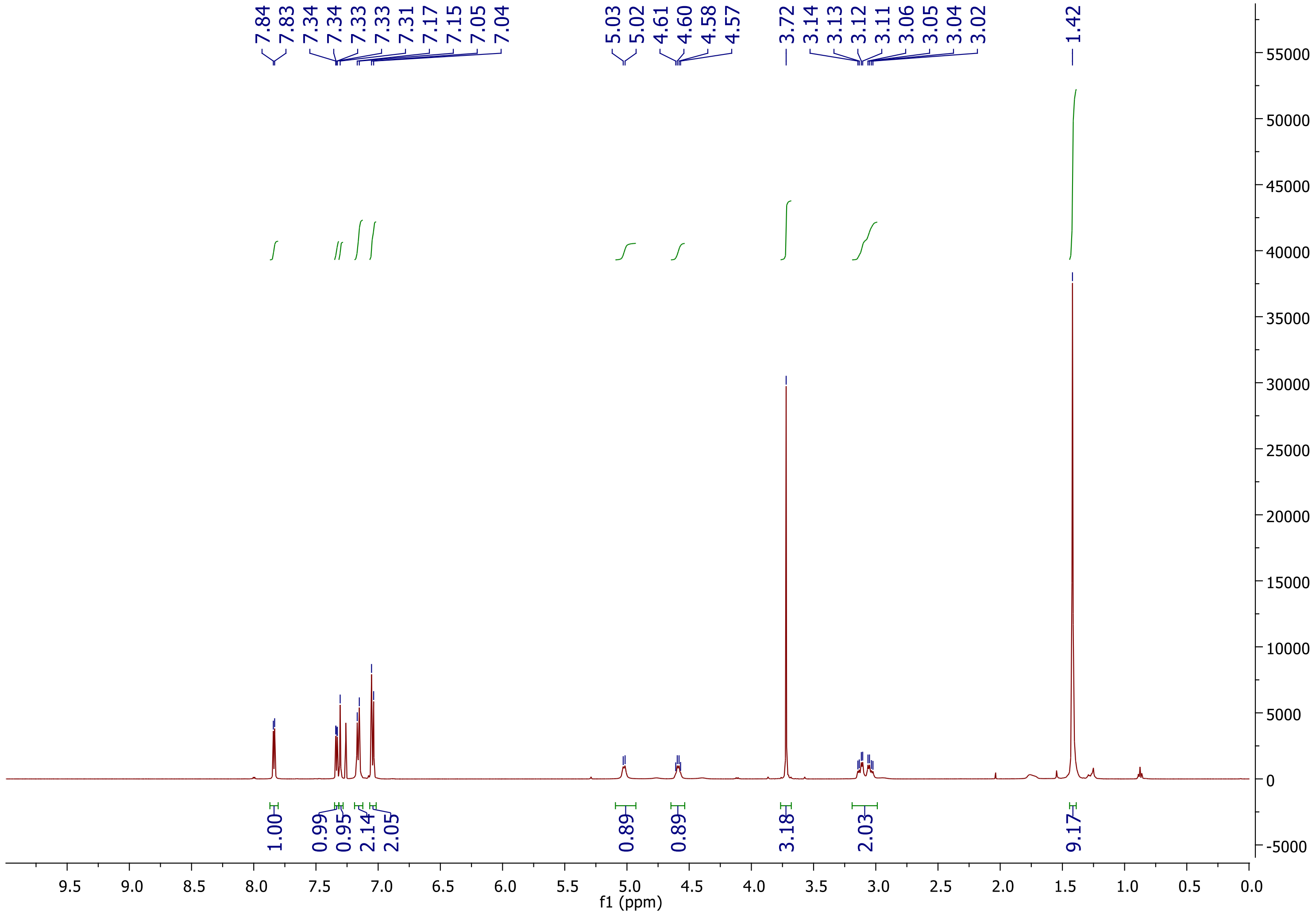

Methyl (*S*)-2-((*t*-butoxycarbonyl)amino)-3-(4-((4-iodopyridin-2-yl)oxy)phenyl)propanoate S31

^{13}C-NMR (126 MHz, $CDCl_3$)

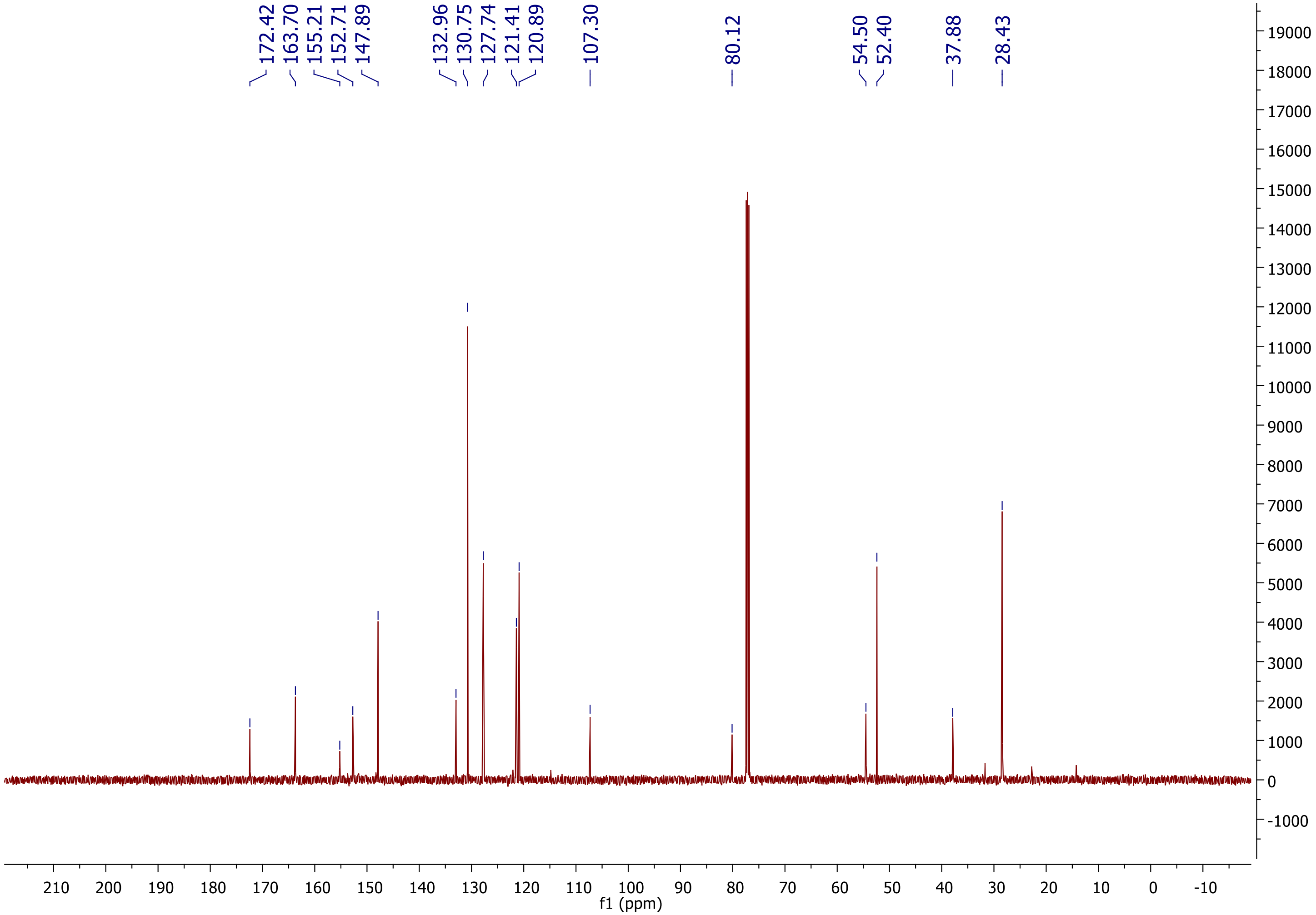

Ethyl 3-(4-ethenyl-6-methylpyridin-2-yl)propanoate A

^{1}H-NMR (500 MHz, $CDCl_3$).

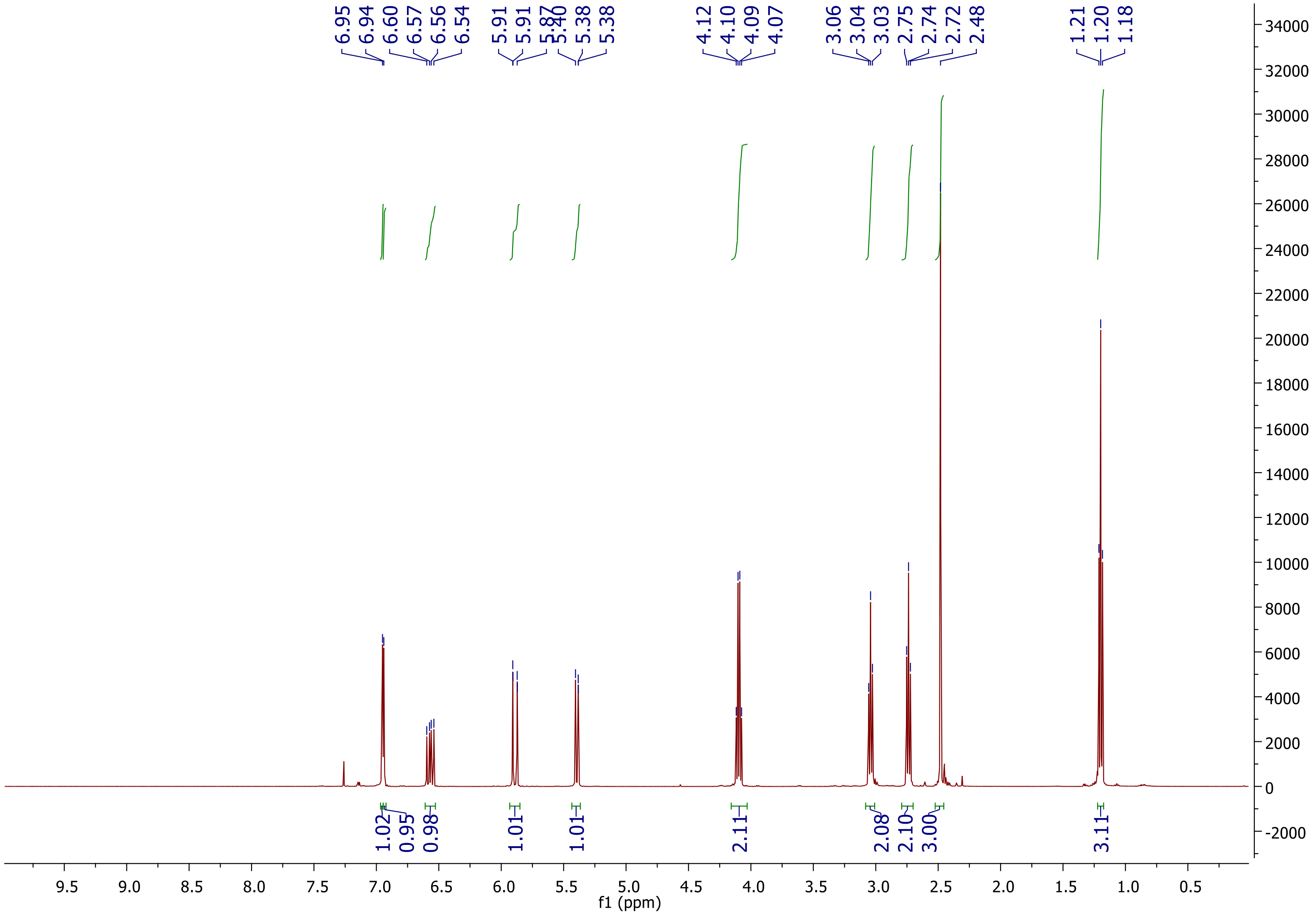

Ethyl 3-(4-ethenyl-6-methylpyridin-2-yl)propanoate A

^{13}C-NMR (126 MHz, $CDCl_3$)

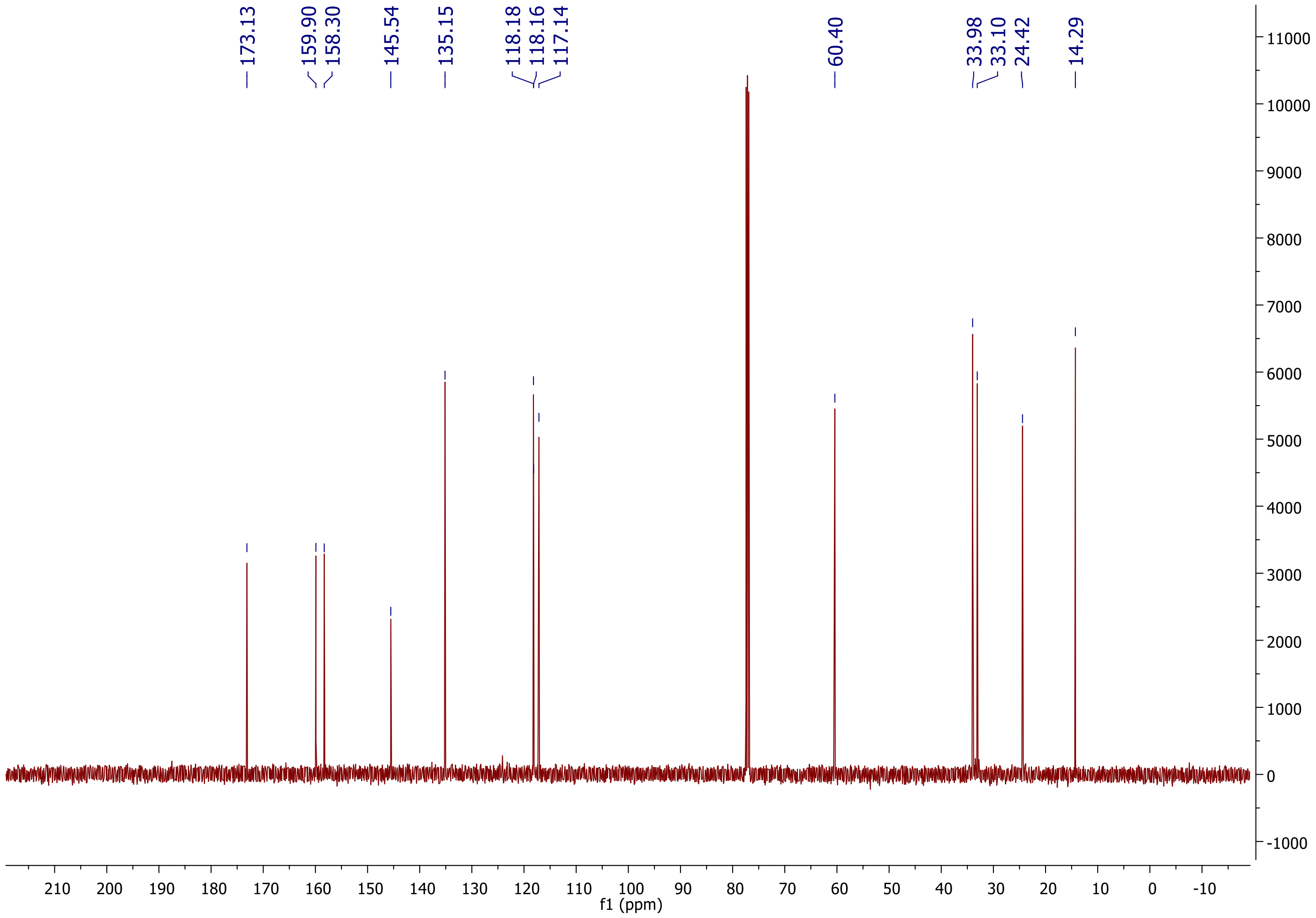

Diethyl 2-[(4-ethenyl-6-methylpyridin-2-yl)methyl]butanedioate B

^{1}H-NMR (500 MHz, $CDCl_3$).

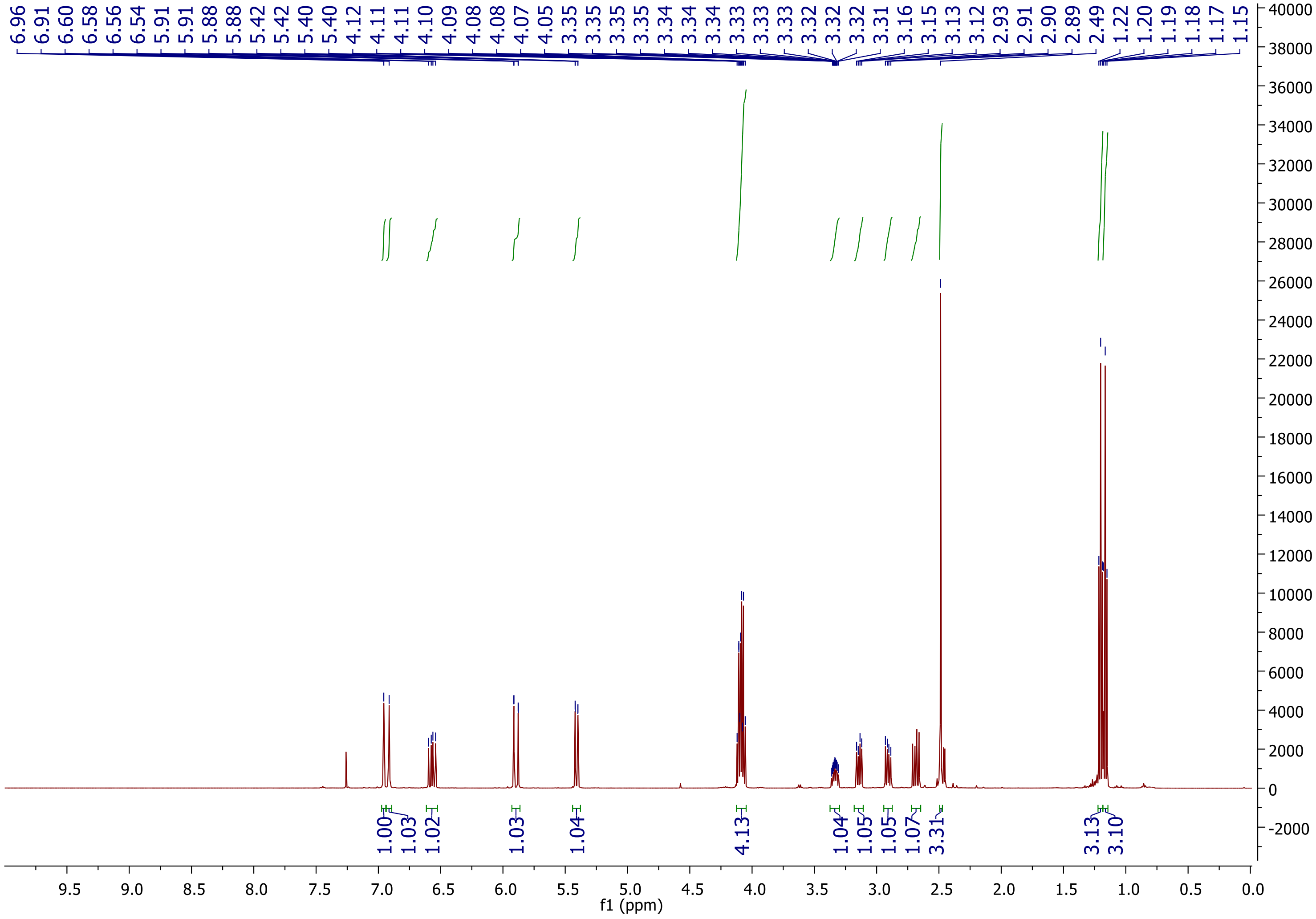

Diethyl 2-[(4-ethenyl-6-methylpyridin-2-yl)methyl]butanedioate B

^{13}C-NMR (126 MHz, $CDCl_3$)

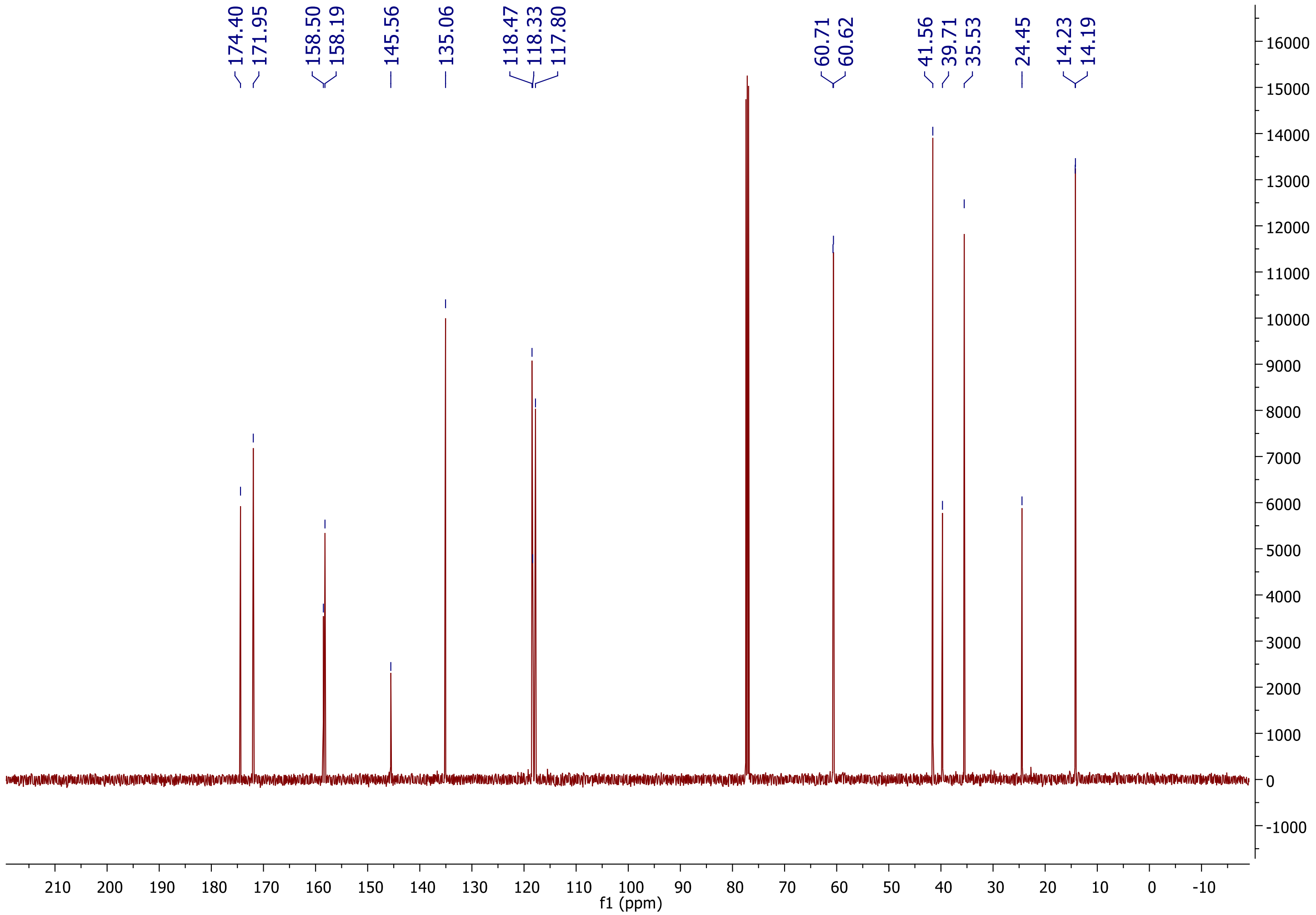

Diethyl 2,2'-(((6-methyl-4-vinylpyridin-2-yl)methyl)azanediyl)diacetate C

^{1}H-NMR (500 MHz, $CDCl_3$).

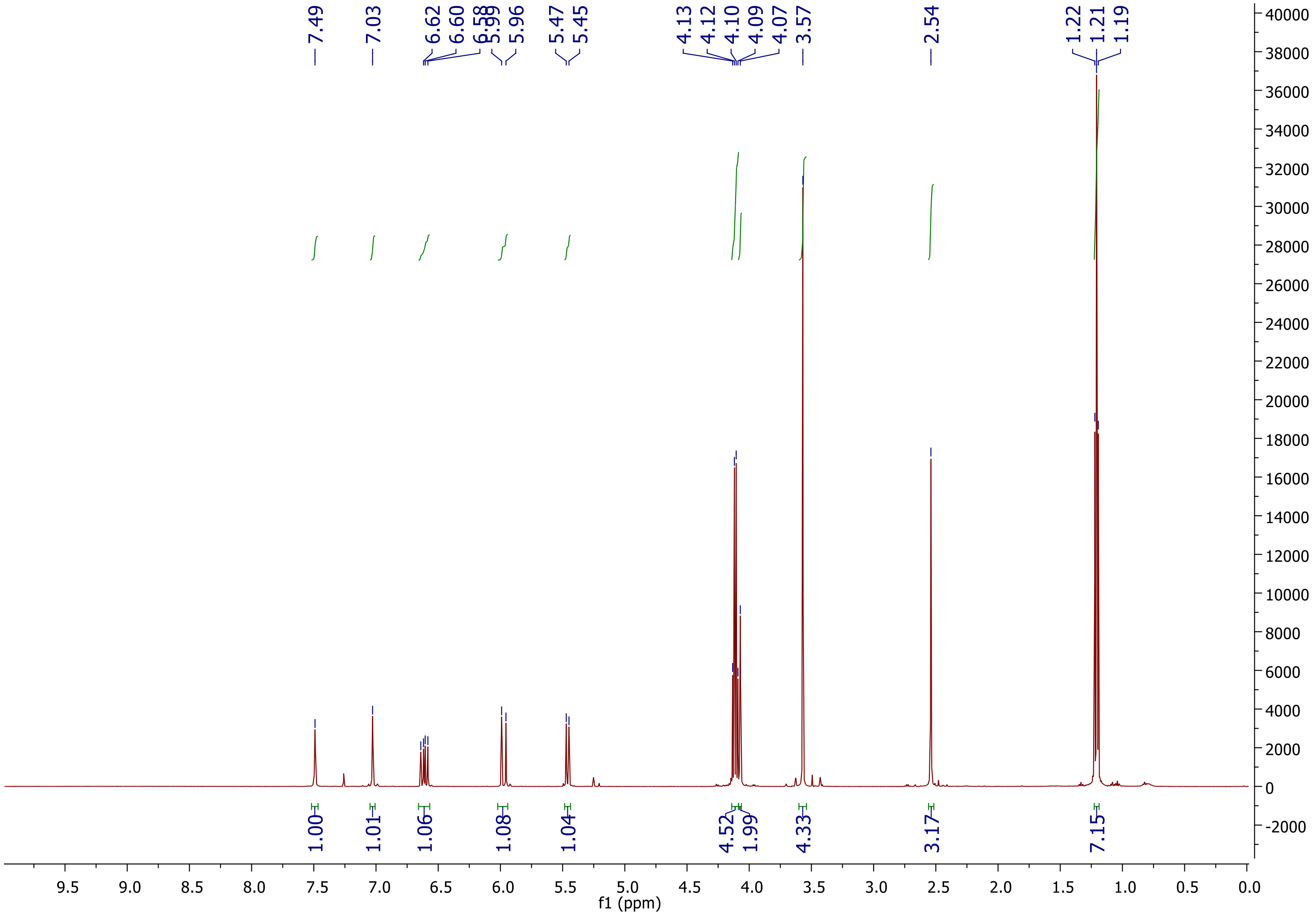

Diethyl 2,2'-(((6-methyl-4-vinylpyridin-2-yl)methyl)azanediyl)diacetate C

^{13}C-NMR (126 MHz, $CDCl_3$)

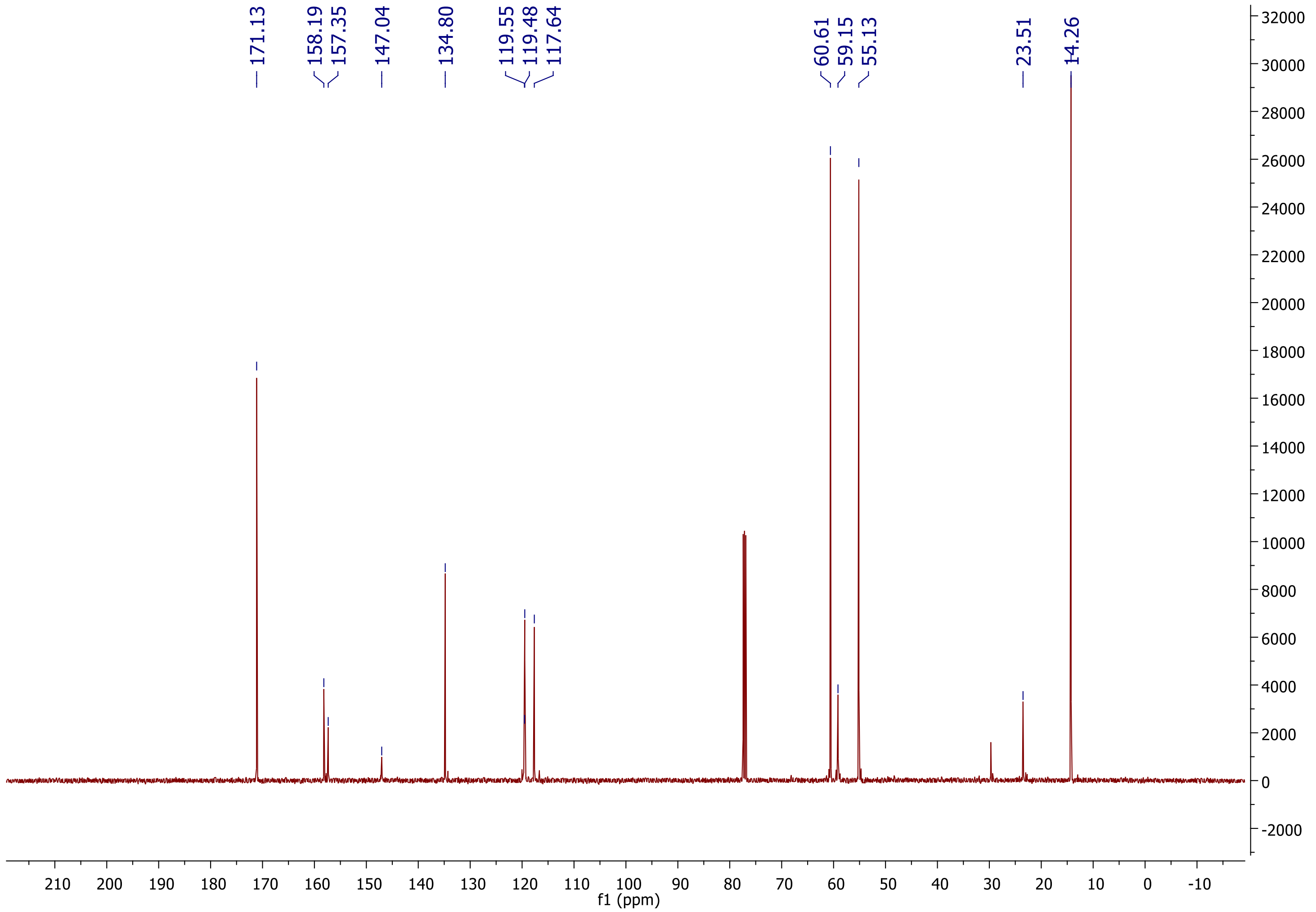

Diethyl 2,2'-((2-(6-methyl-4-vinylpyridin-2-yl)ethyl)azanediyl)diacetate D

^{1}H-NMR (500 MHz, $CDCl_3$).

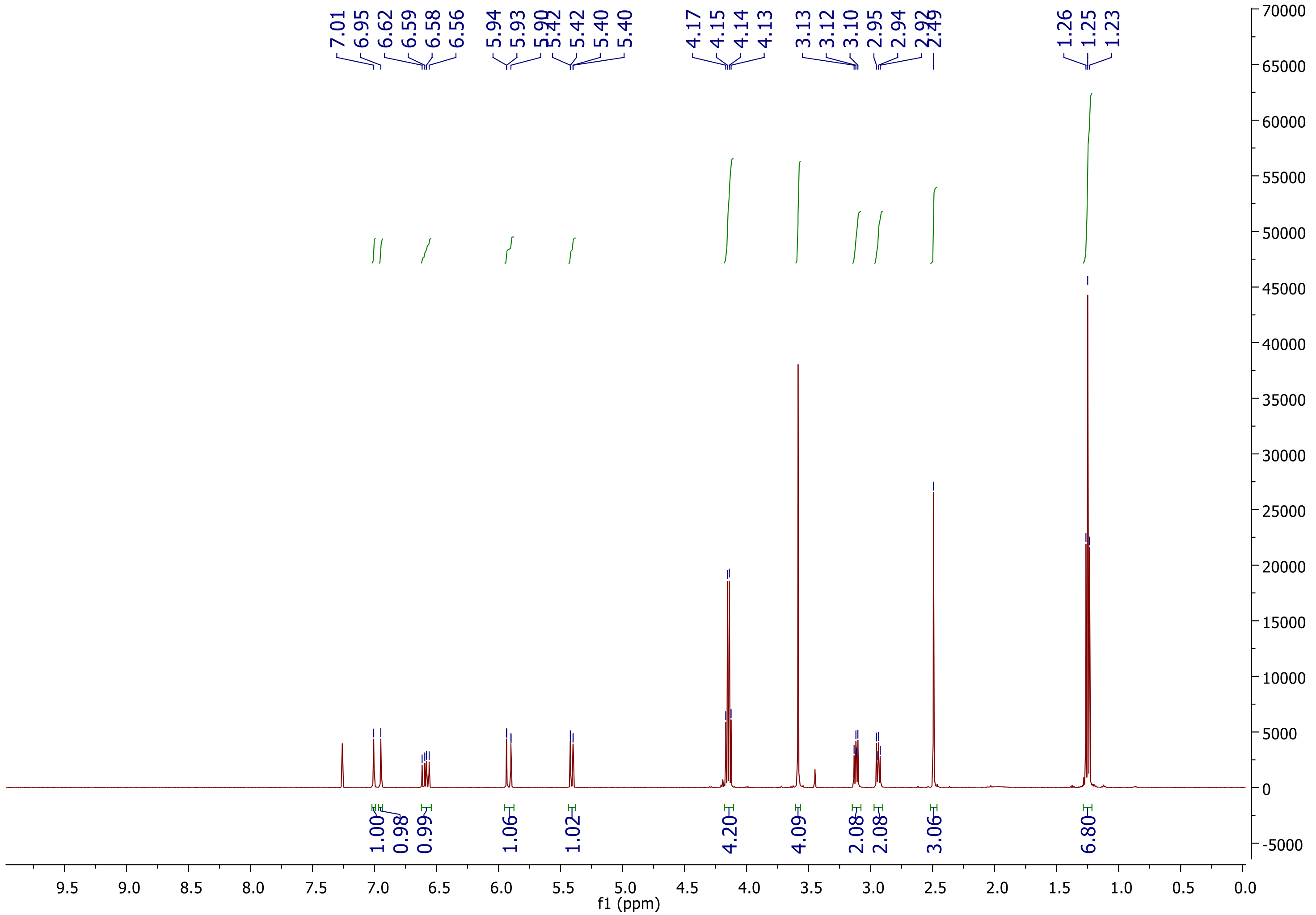

Diethyl 2,2'-((2-(6-methyl-4-vinylpyridin-2-yl)ethyl)azanediyl)diacetate D

^{13}C-NMR (126 MHz, $CDCl_3$)

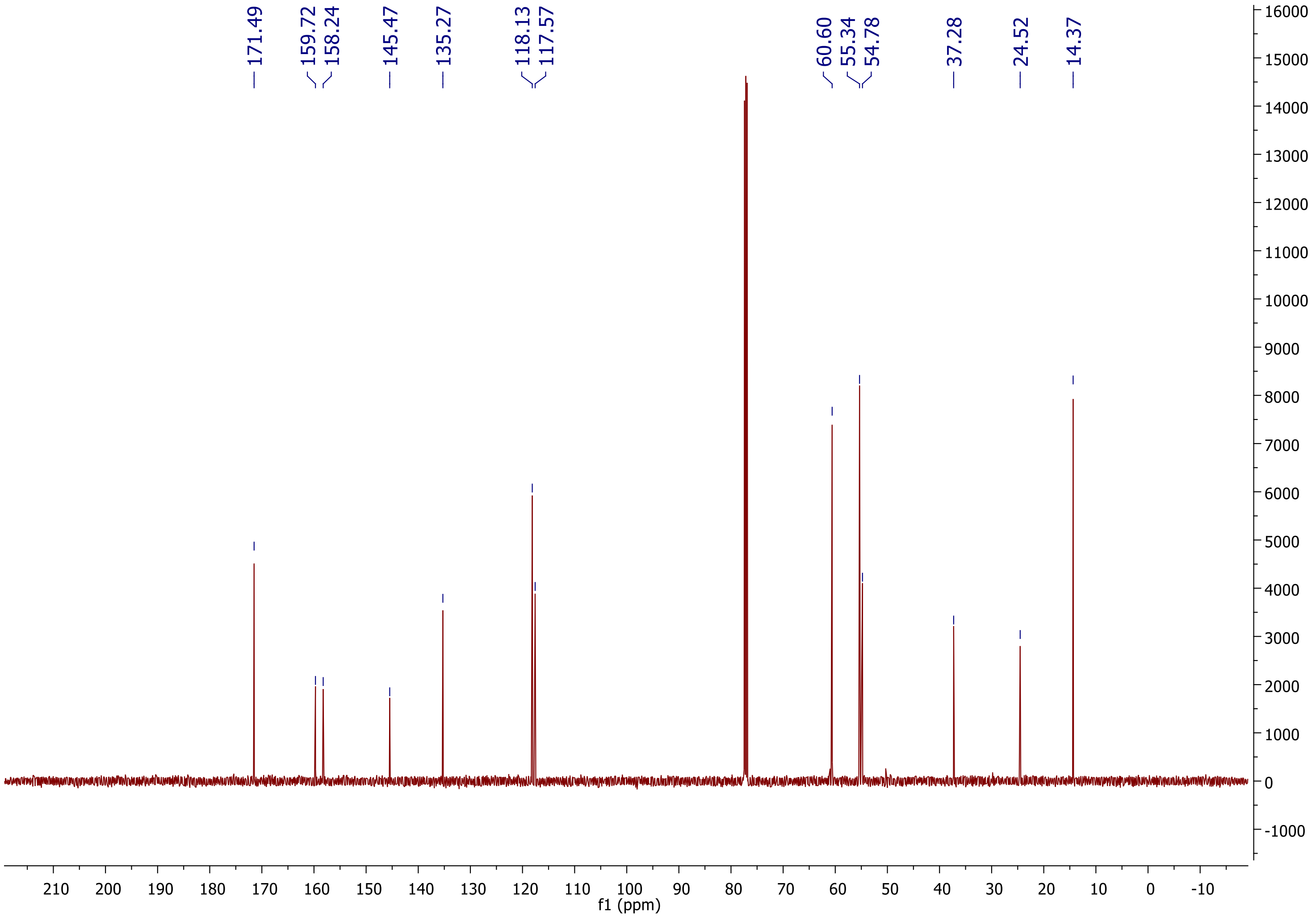

Diethyl 2,2'-((3-(6-methyl-4-vinylpyridin-2-yl)propyl)azanediyl)diacetate E

^{1}H-NMR (500 MHz, $CDCl_3$).

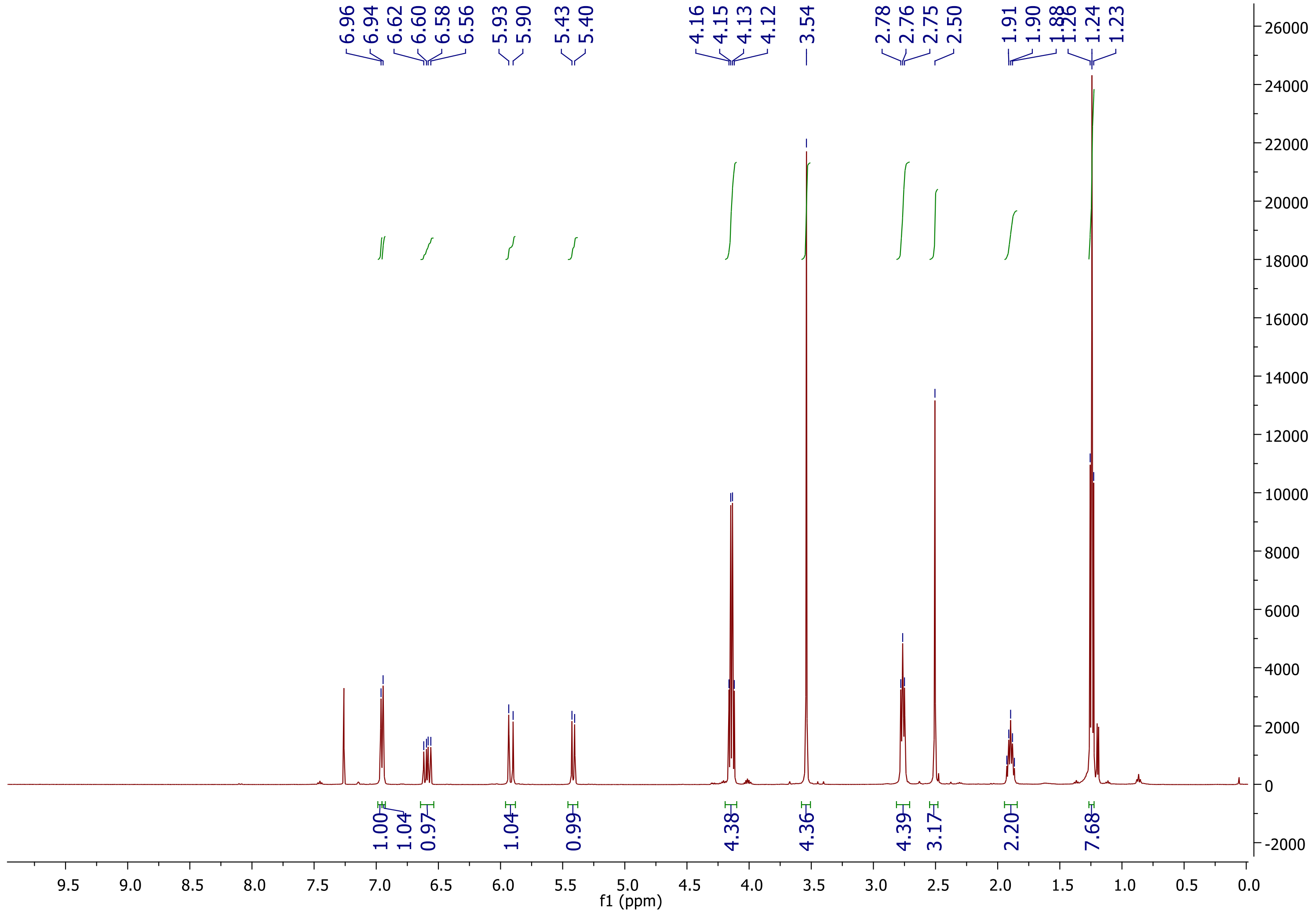

Diethyl 2,2'-((3-(6-methyl-4-vinylpyridin-2-yl)propyl)azanediyl)diacetate E

^{13}C-NMR (126 MHz, $CDCl_3$)

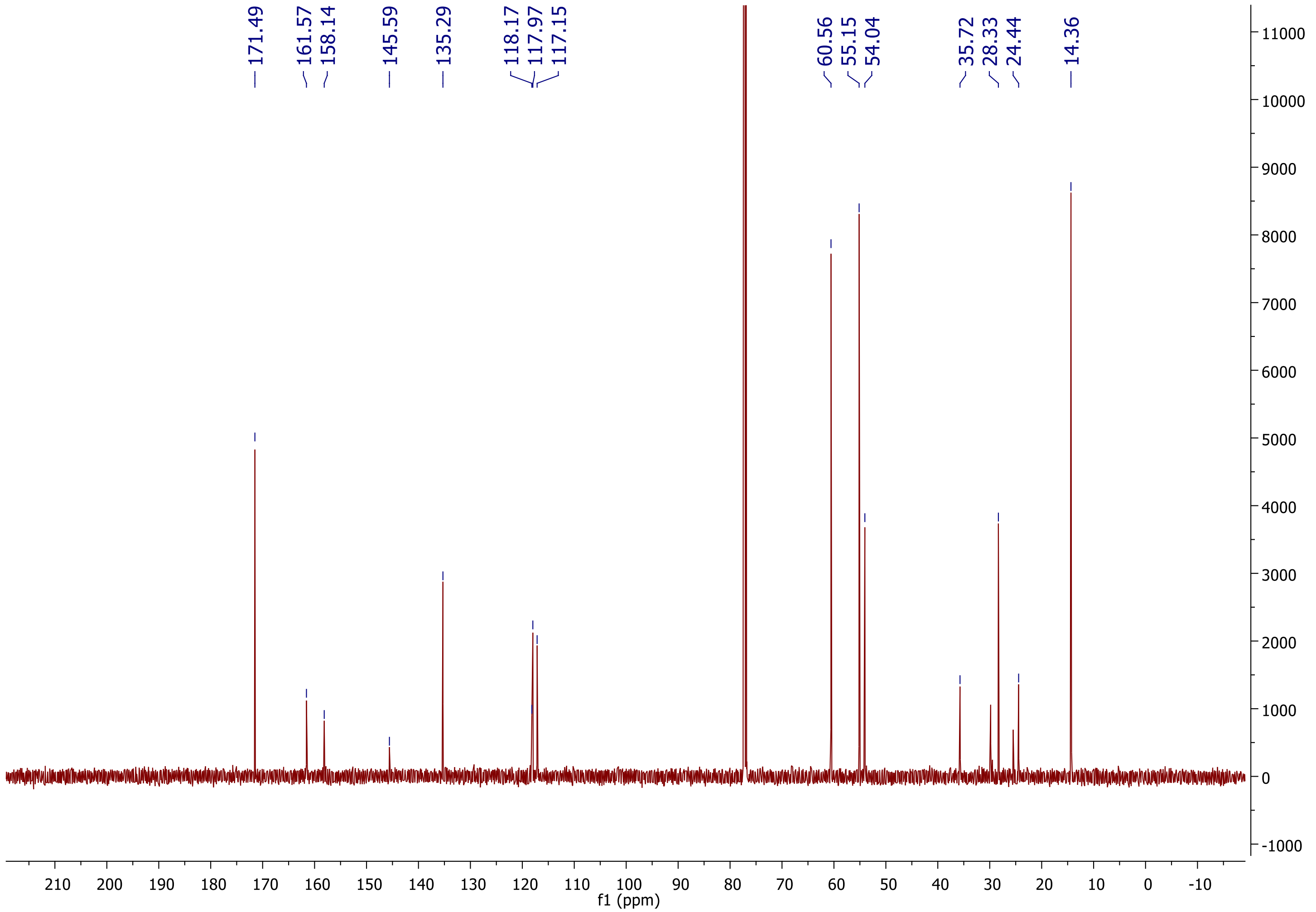

Diethyl 3,3'-(4-ethenylpyridine-2,6-diyl)dipropanoate F

^{1}H-NMR (500 MHz, $CDCl_3$).

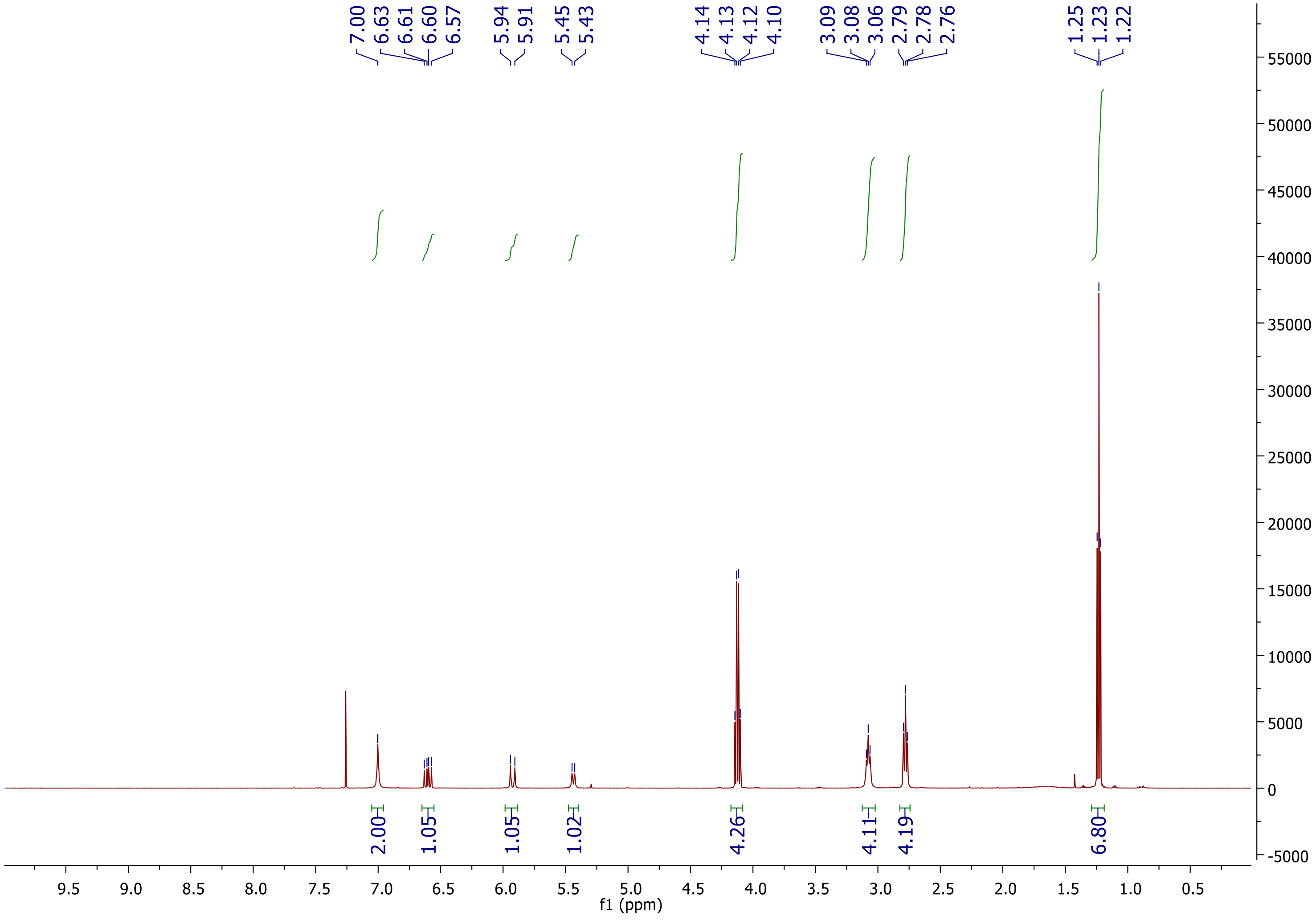

Diethyl 3,3'-(4-ethenylpyridine-2,6-diyl)dipropanoate F

^{13}C-NMR (126 MHz, $CDCl_3$)

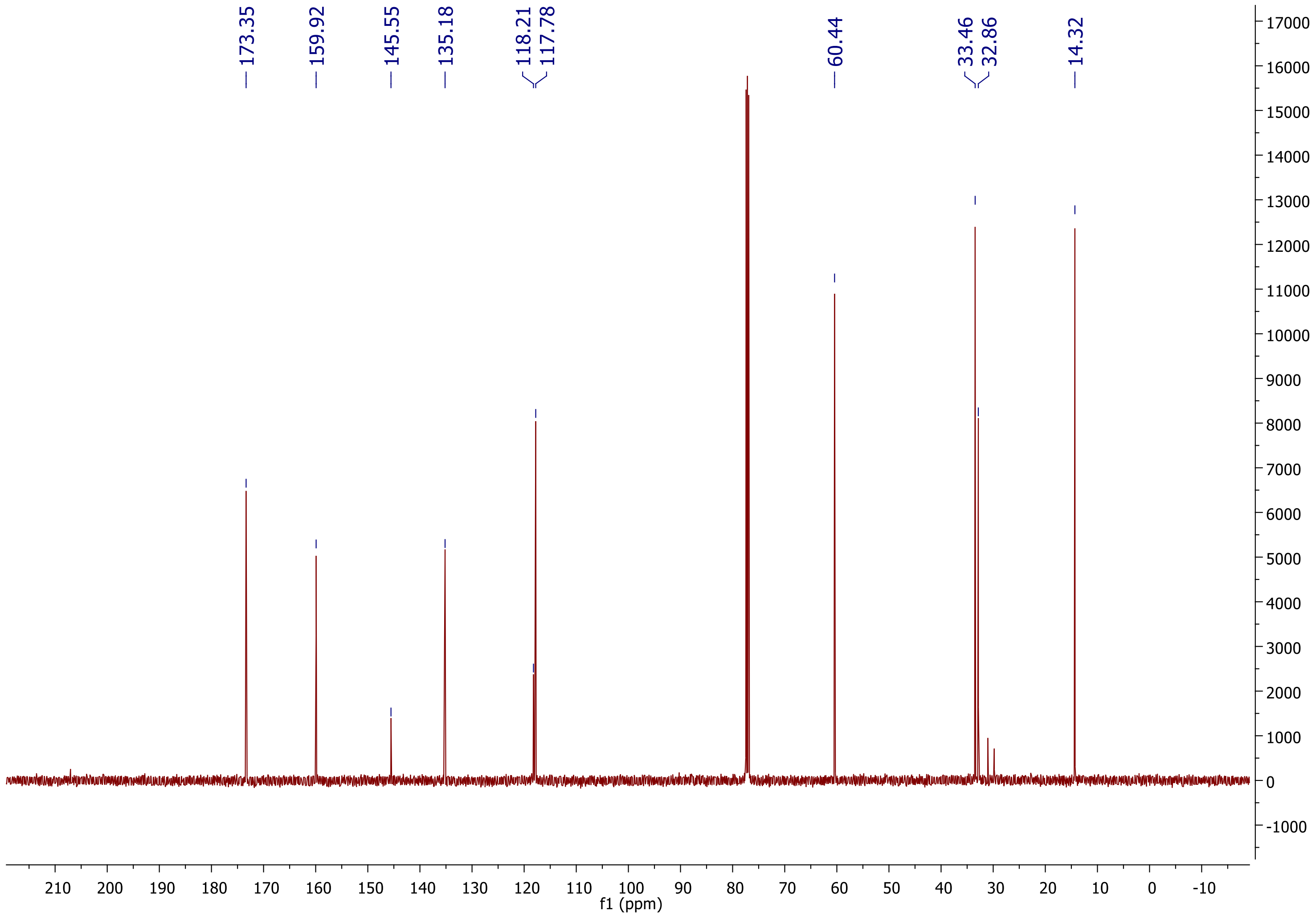

Ethyl 3-(4-ethenyl-6-{[4-(2-hydroxypropan-2-yl)-1H-1,2,3-triazol-1-yl]methyl}pyridin-2-yl)propanoate G

^{1}H-NMR (500 MHz, $CDCl_3$).

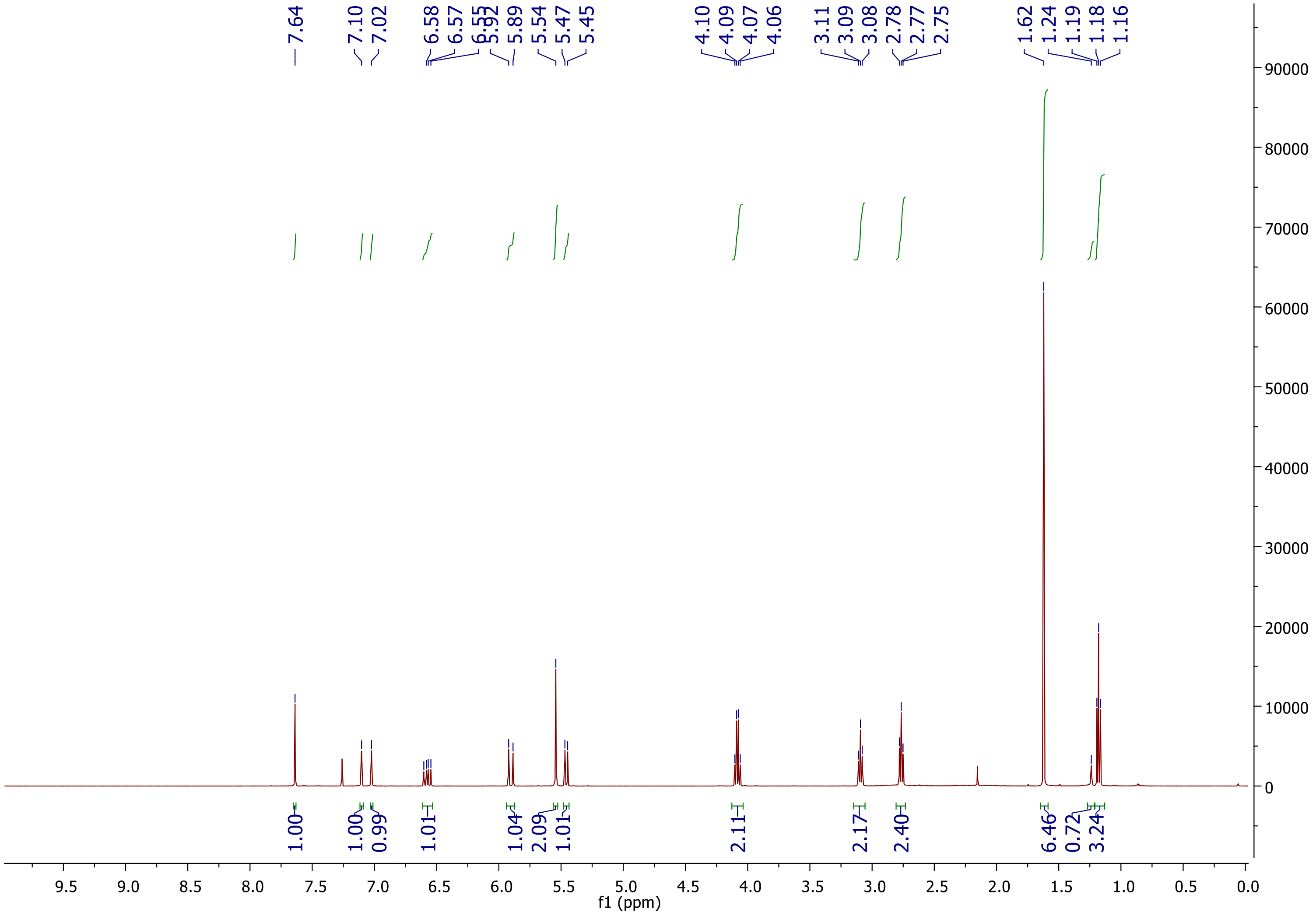

Ethyl 3-(4-ethenyl-6-{[4-(2-hydroxypropan-2-yl)-1H-1,2,3-triazol-1-yl]methyl}pyridin-2-yl)propanoate G

^{13}C-NMR (126 MHz, $CDCl_3$)

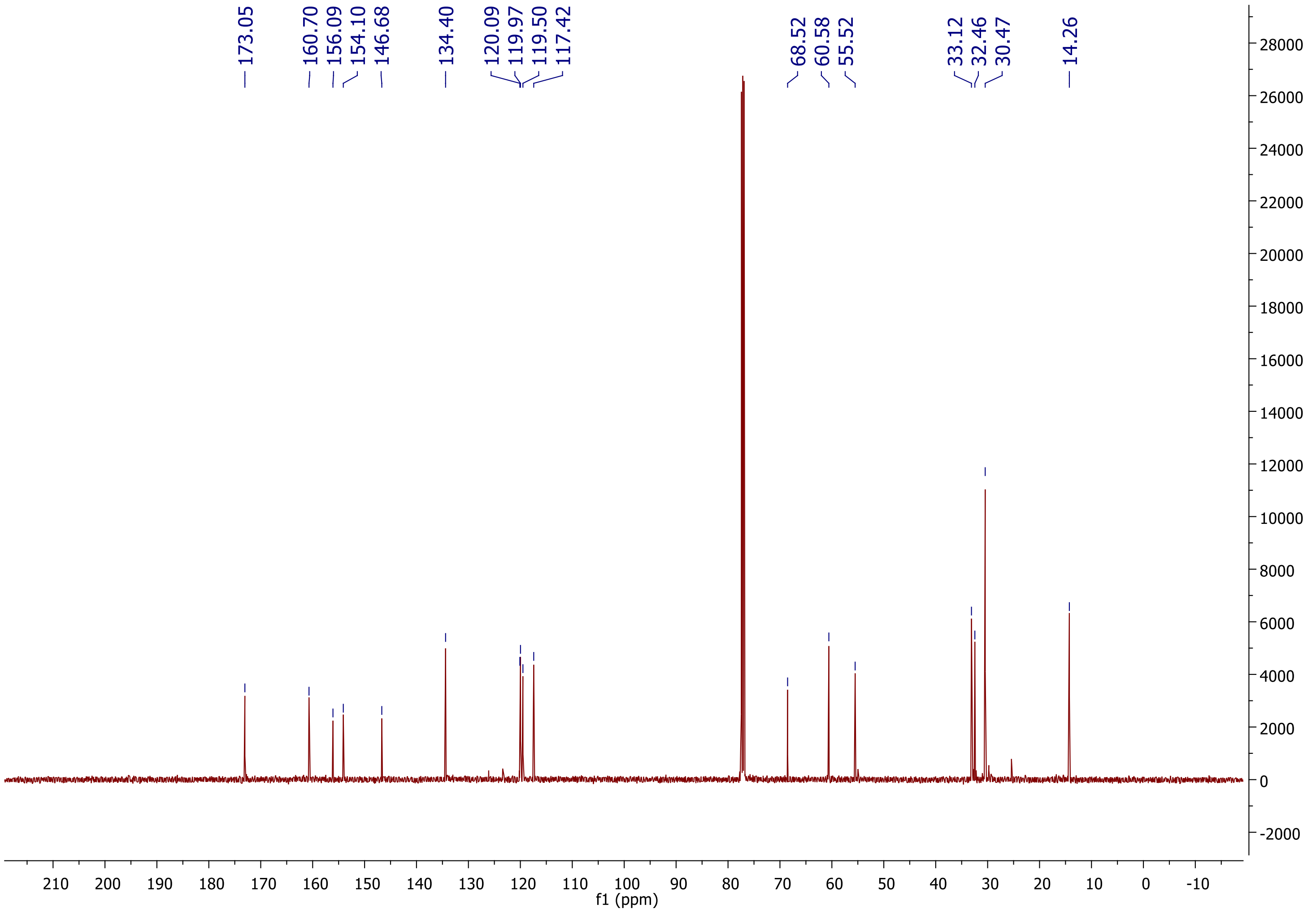

Ethyl 3-(6-{[(*t*-butoxycarbonyl)amino]methyl}-4-ethenylpyridin-2-yl)propanoate H

^{1}H-NMR (400 MHz, $CDCl_3$).

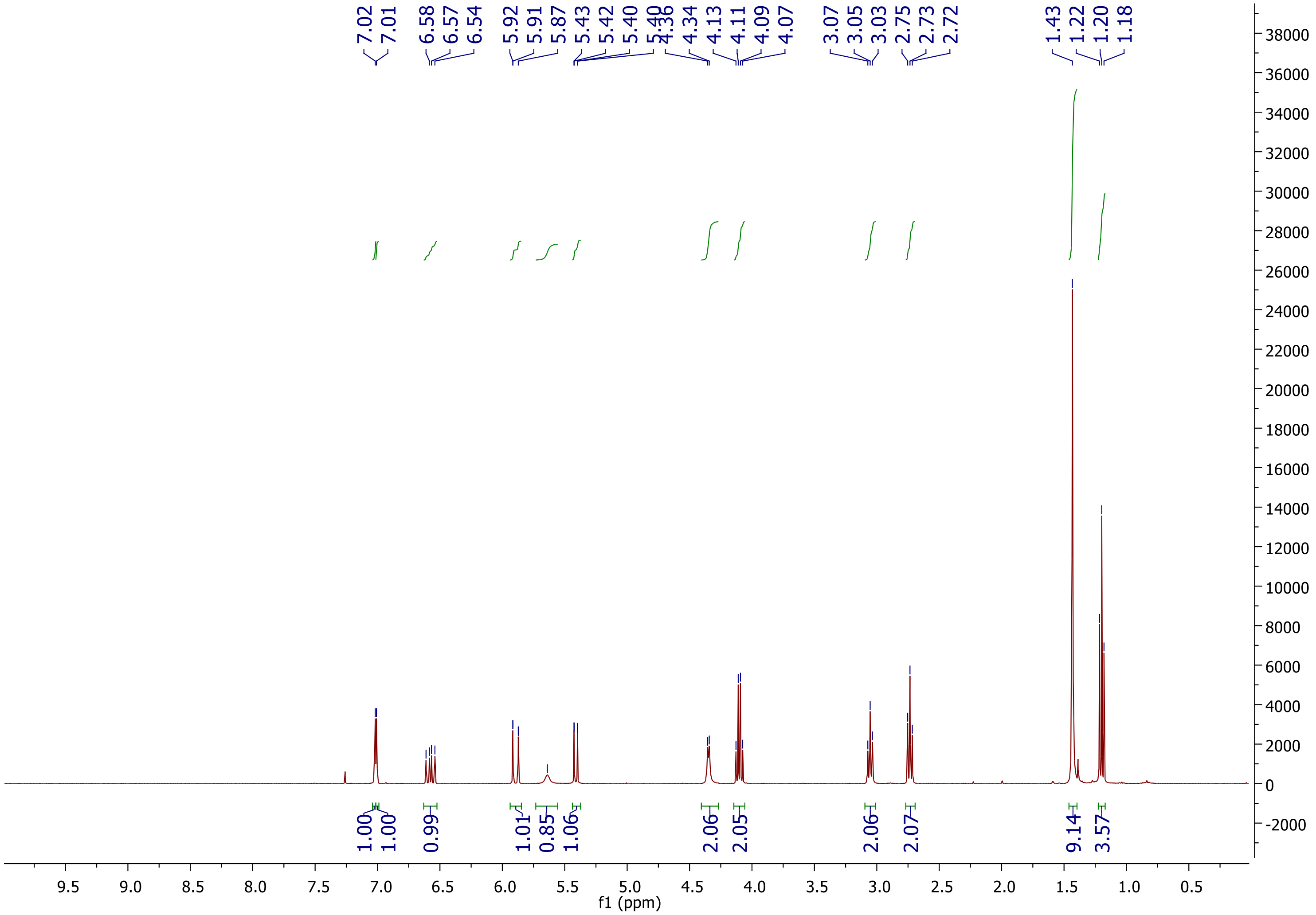

Ethyl 3-(6-{[(*t*-butoxycarbonyl)amino]methyl}-4-ethenylpyridin-2-yl)propanoate H

^{13}C-NMR (101 MHz, $CDCl_3$)

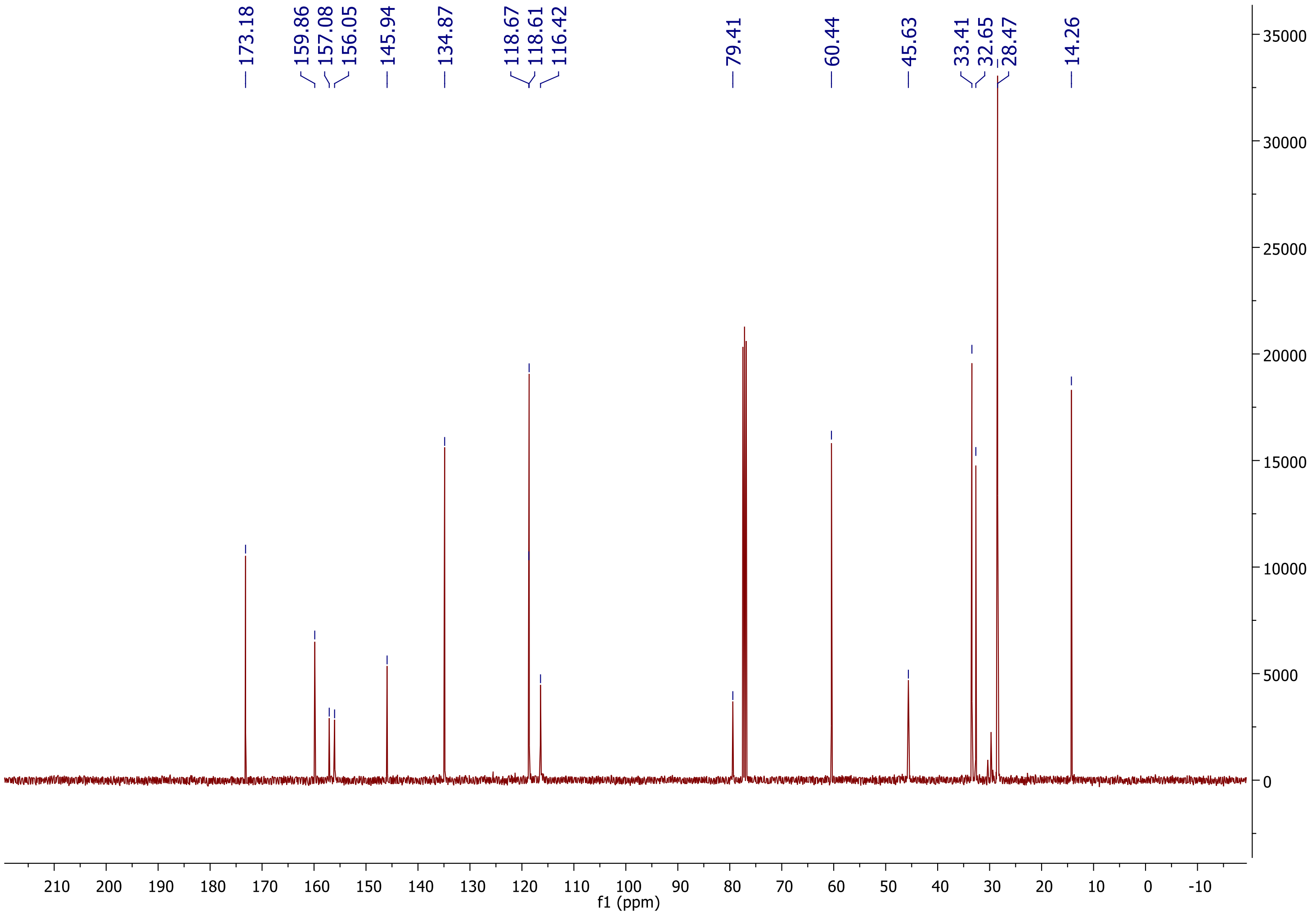

Di-*t*-butyl [(4-ethenylpyridine-2,6-diyl)bis(methylene)]biscarbamate I

^{1}H-NMR (500 MHz, $CDCl_3$).

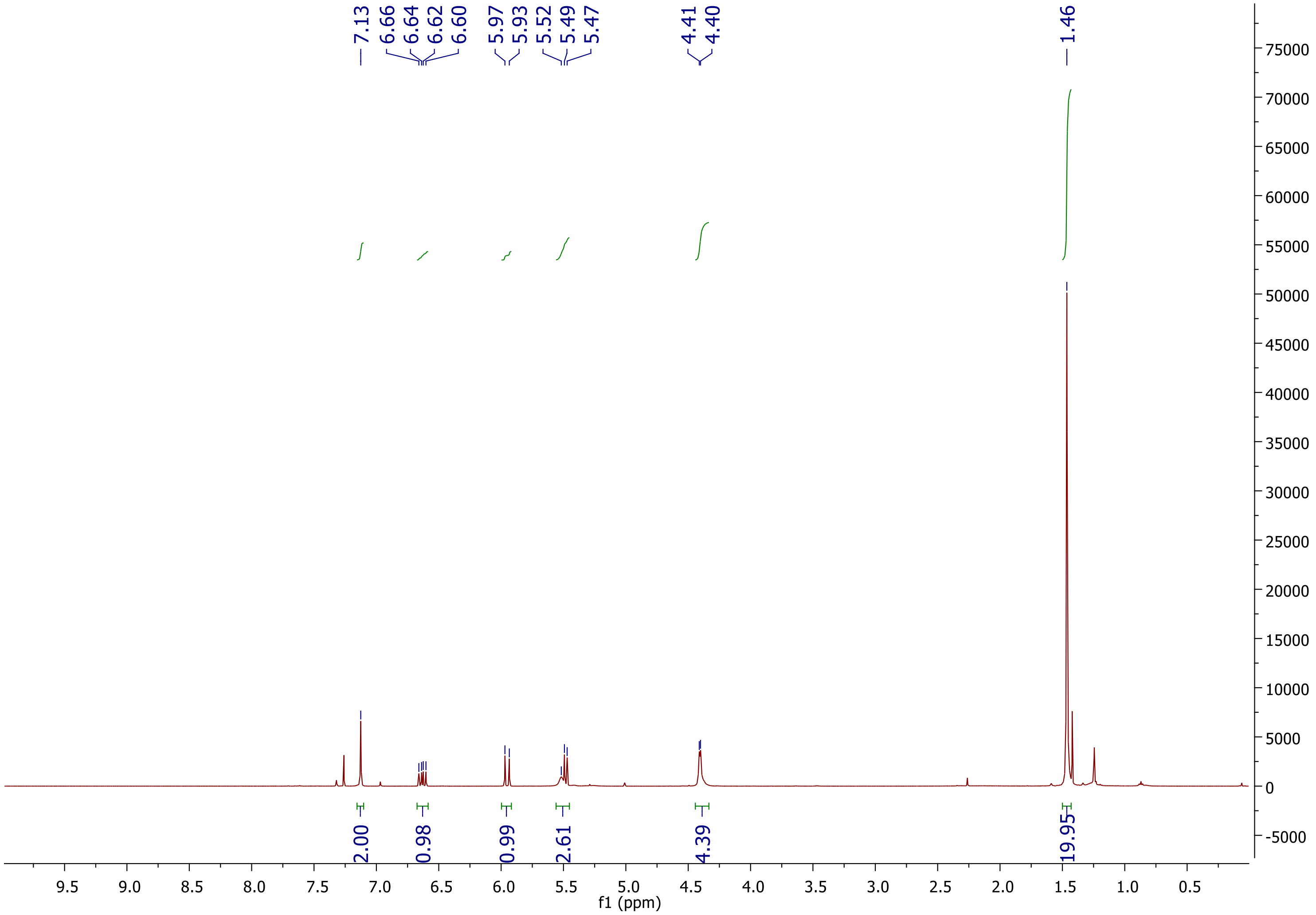

Di-*t*-butyl [(4-ethenylpyridine-2,6-diyl)bis(methylene)]biscarbamate I

^{13}C-NMR (101 MHz, $CDCl_3$)

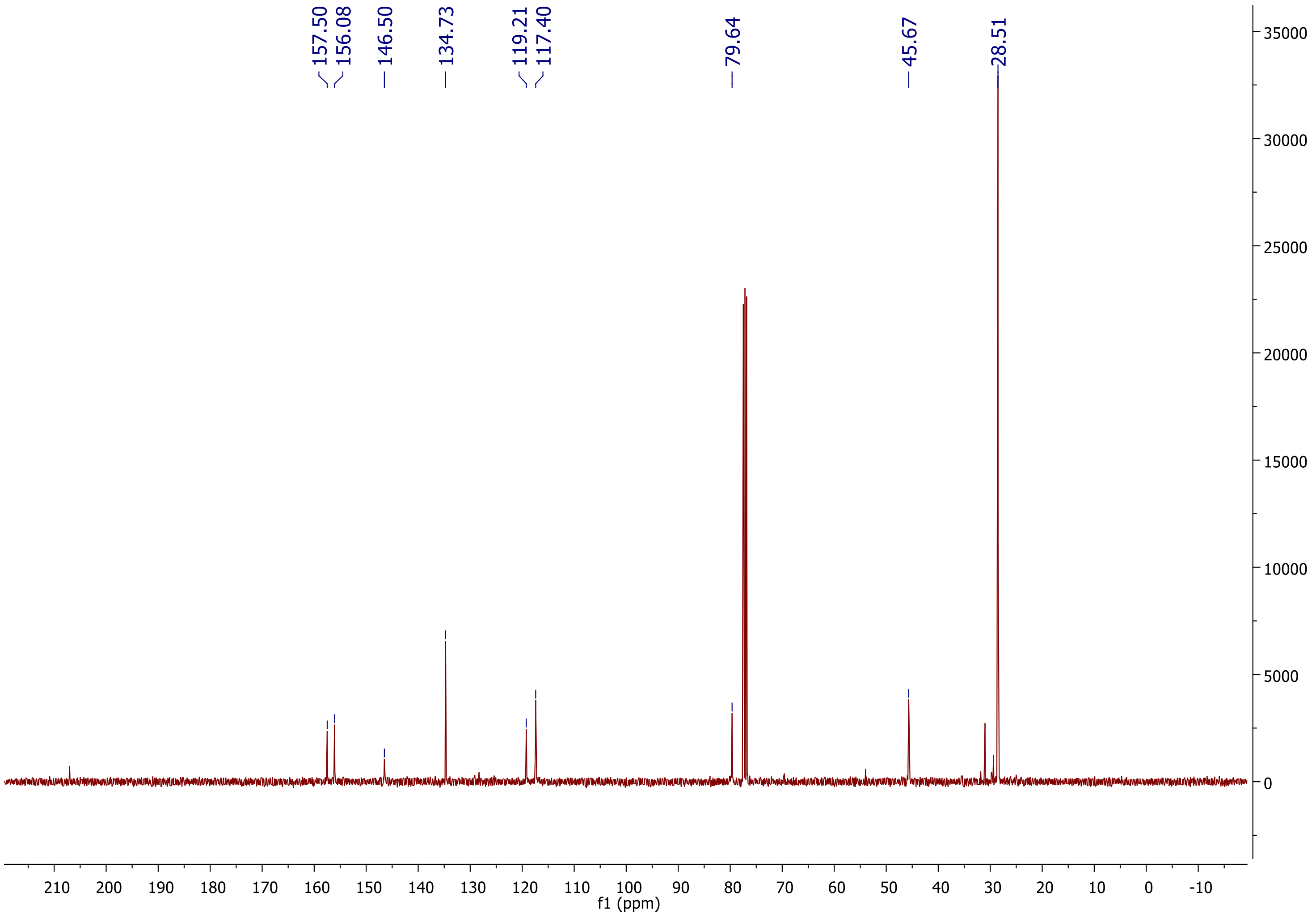

***t*-Butyl [(4-ethenyl-6-{[4-(2-hydroxypropan-2-yl)-1H-1,2,3-triazol-1-yl]methyl}pyridin-2-yl)methyl]carbamate J**

^{1}H-NMR (500 MHz, $CDCl_3$).

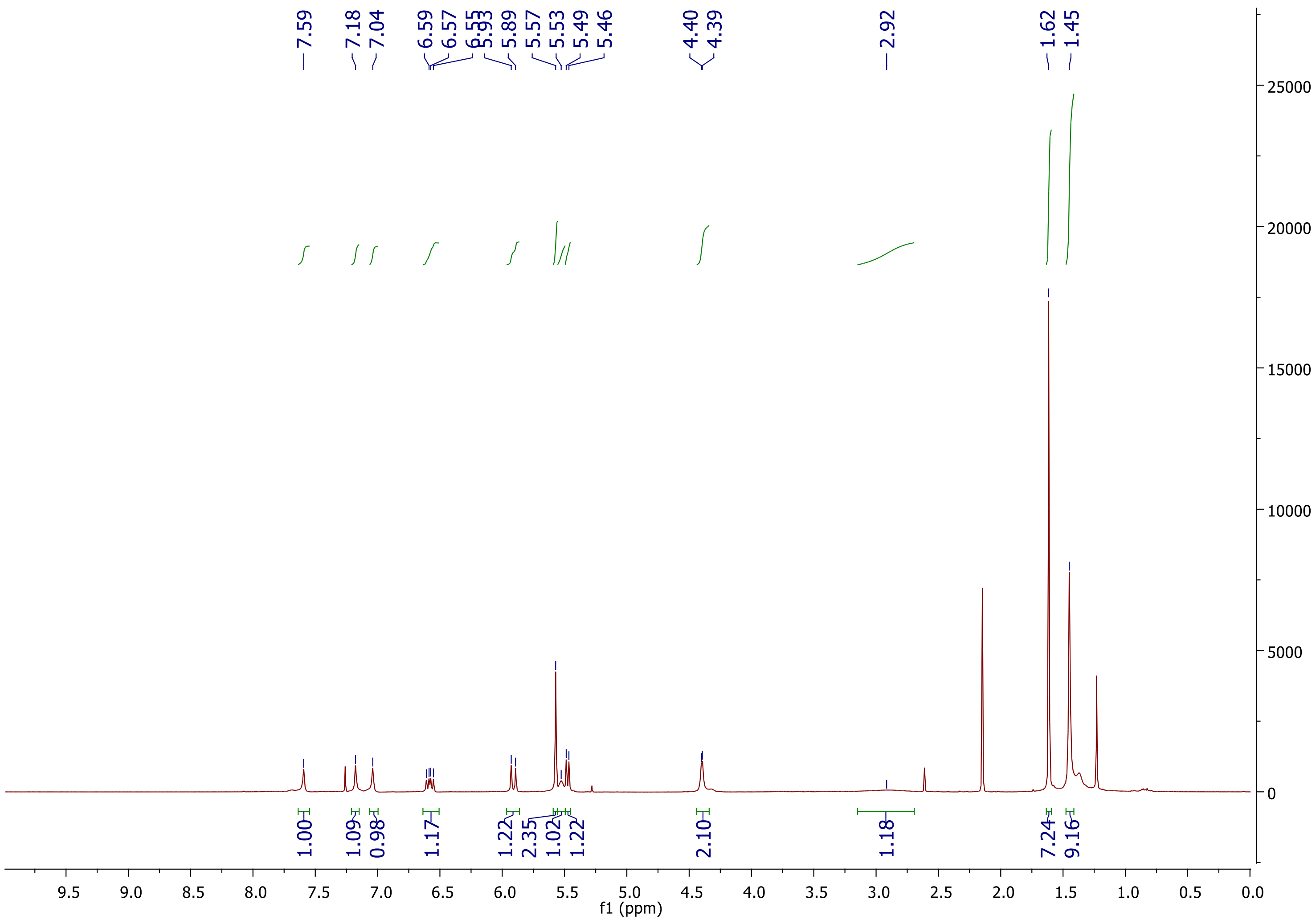

_t_-Butyl [(4-ethenyl-6-{[4-(2-hydroxypropan-2-yl)-1H-1,2,3-triazol-1-yl]methyl}pyridin-2-yl)methyl]carbamate J

^{13}C-NMR (126 MHz, $CDCl_3$)

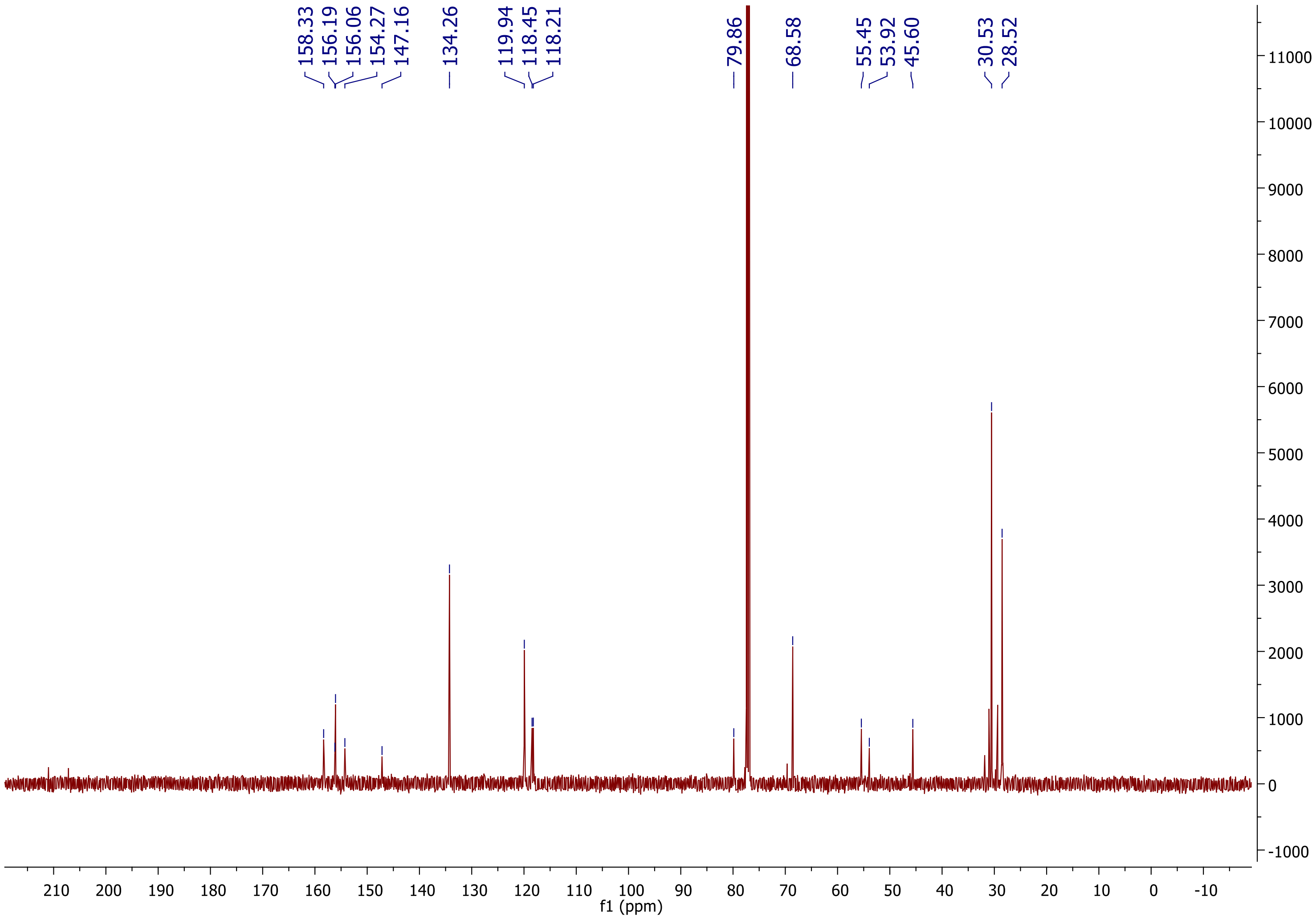

2,2'-[(4-Ethenylpyridine-2,6-diyl)bis(methylene-1H-1,2,3-triazole-1,4-diyl)]di(propan-2-ol) K

^{1}H-NMR (500 MHz, $CDCl_3$).

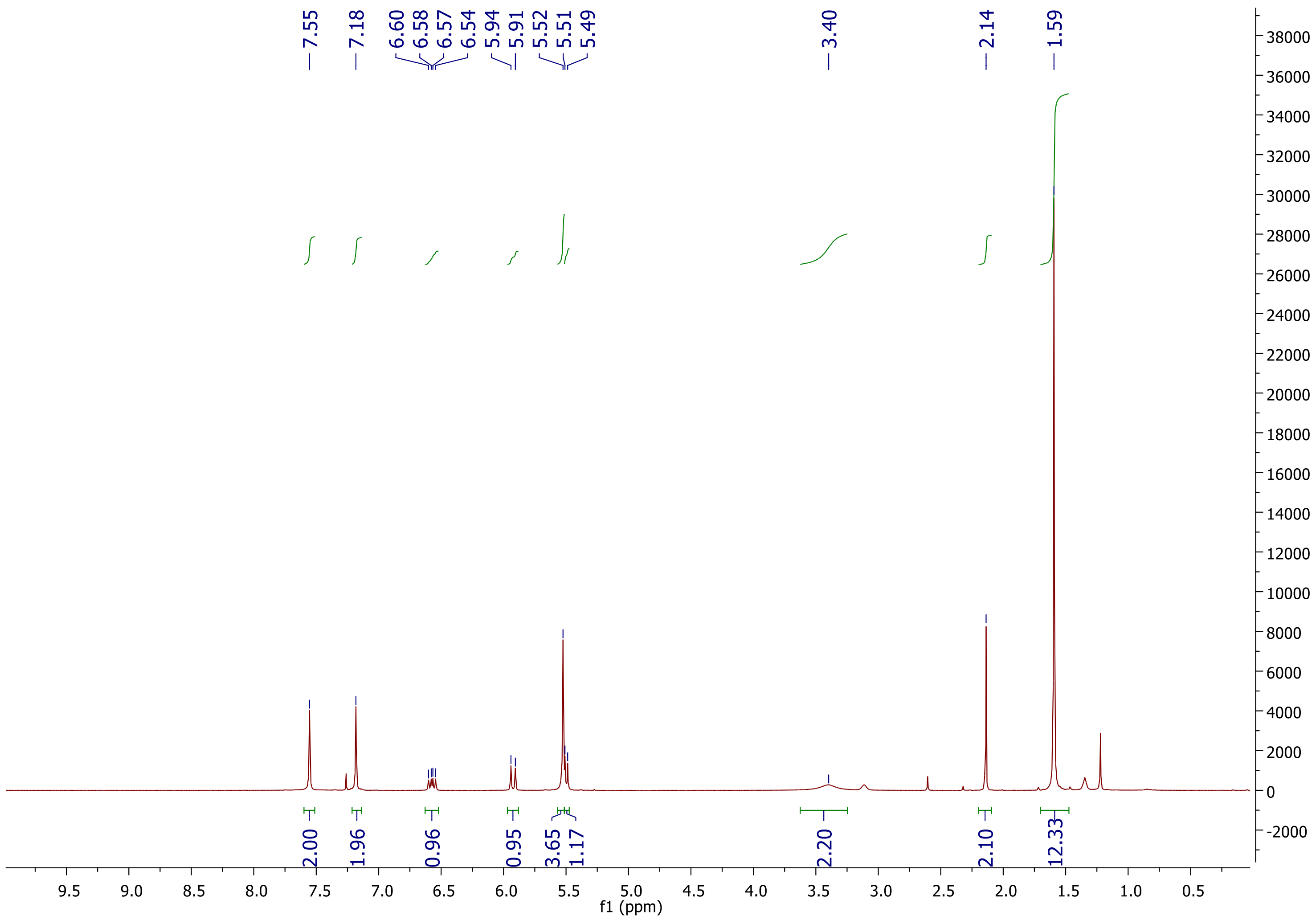

2,2'-[(4-Ethenylpyridine-2,6-diyl)bis(methylene-1H-1,2,3-triazole-1,4-diyl)]di(propan-2-ol) K

^{13}C-NMR (126 MHz, $CDCl_3$)

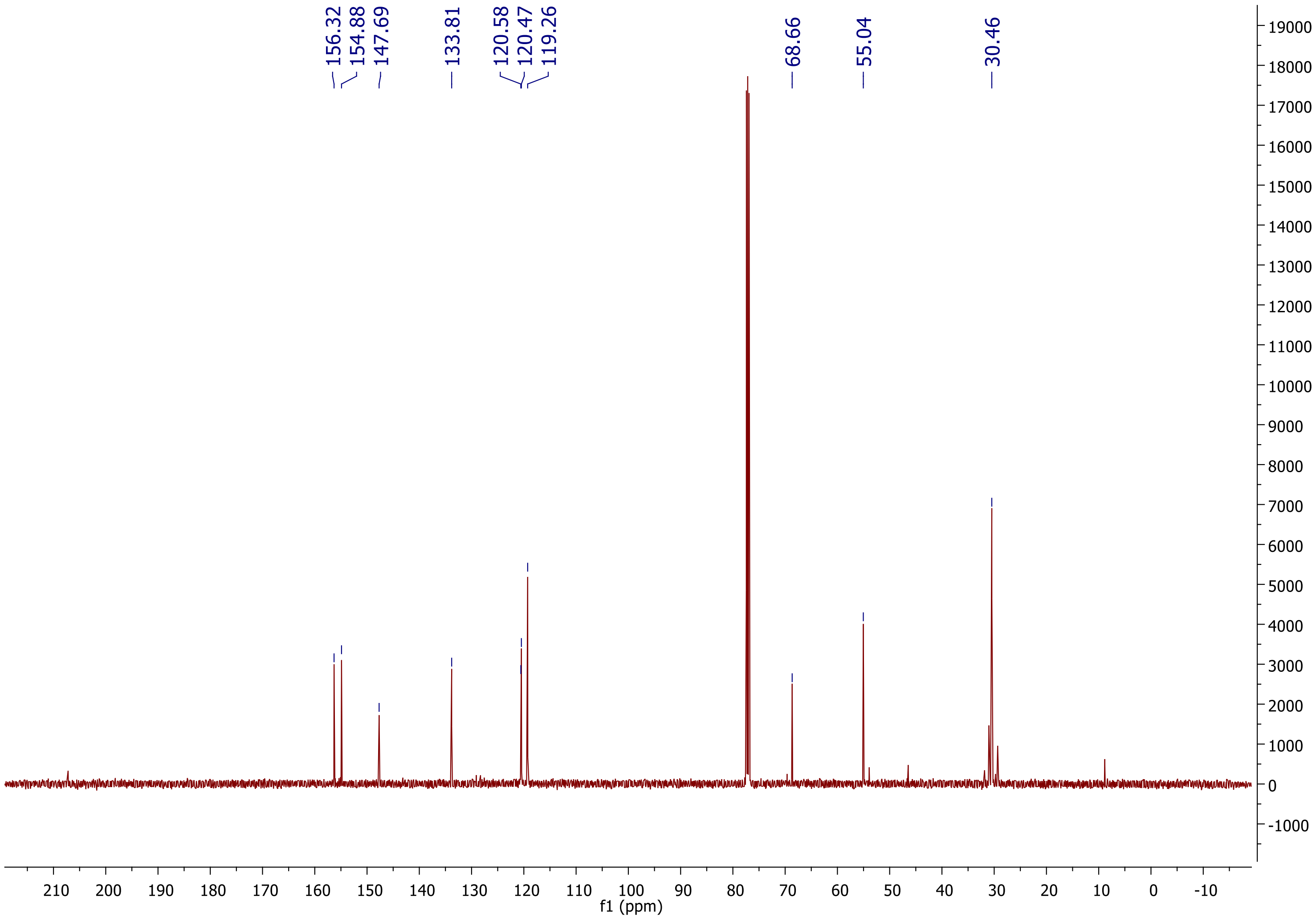

2-(Pyrrolidin-1-yl)-4-vinylpyridine L

^{1}H-NMR (500 MHz, $CDCl_3$).

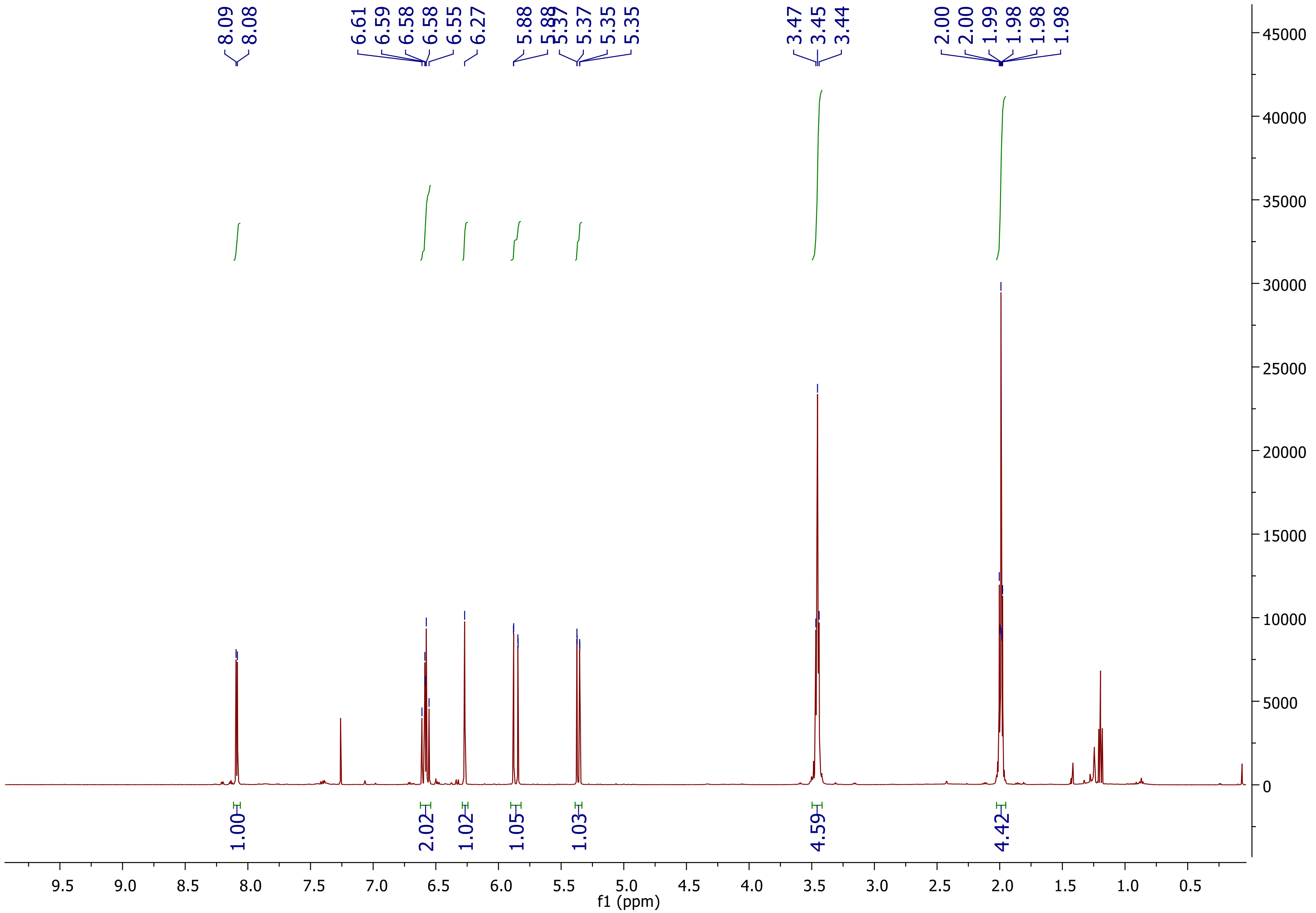

2-(Pyrrolidin-1-yl)-4-vinylpyridine L

^{13}C-NMR (126 MHz, $CDCl_3$)

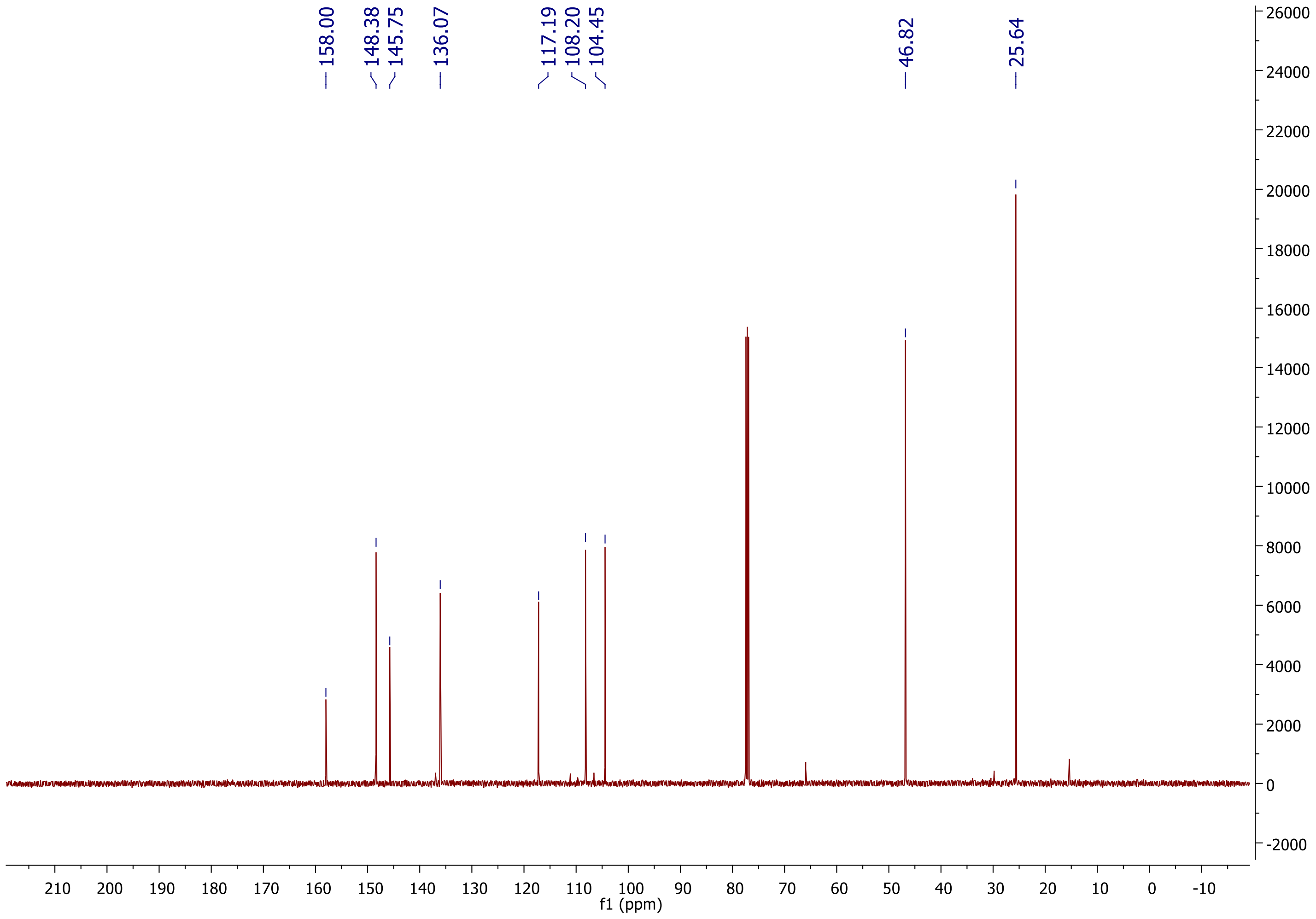

Methyl (*S*)-2-((*t*-butoxycarbonyl)amino)-3-(4-((4-vinylpyridin-2-yl)oxy)phenyl)propanoate M

^{1}H-NMR (500 MHz, $CDCl_3$).

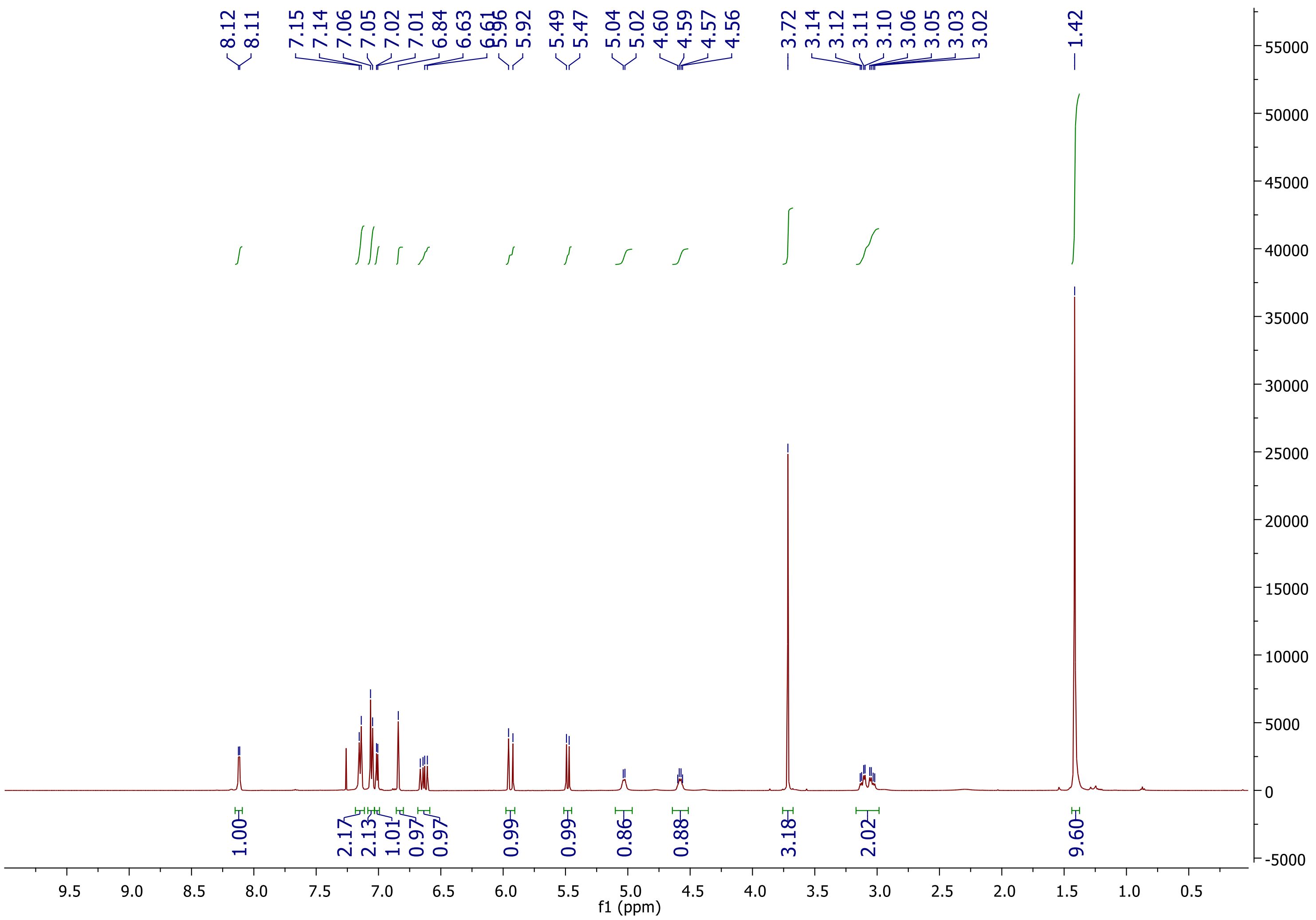

Methyl (*S*)-2-((*t*-butoxycarbonyl)amino)-3-(4-((4-vinylpyridin-2-yl)oxy)phenyl)propanoate M

^{13}C-NMR (126 MHz, $CDCl_3$)

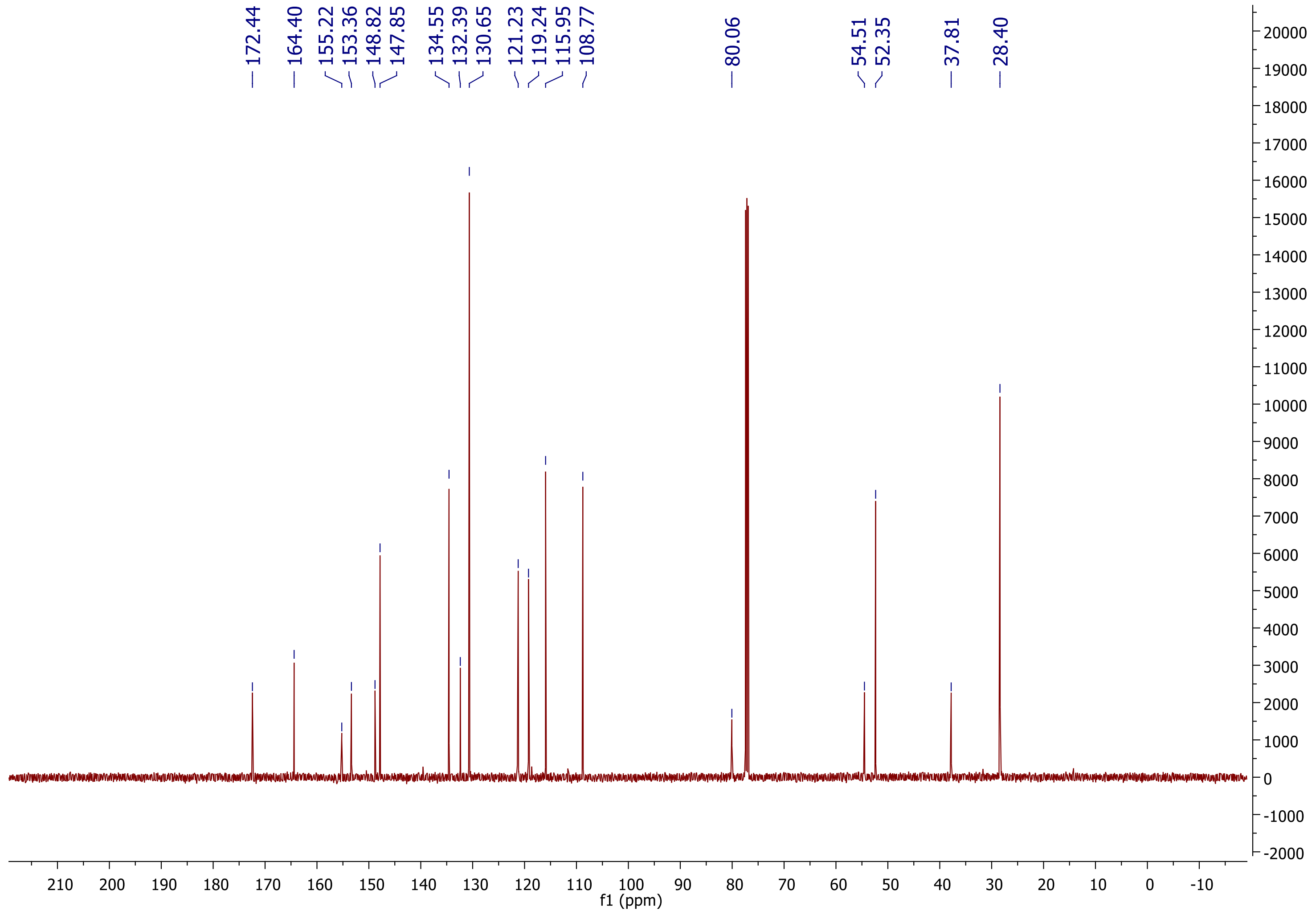

S12. Example kinetic data for reaction of F with glutathione and protonation analysis

Glutathione test:[71]

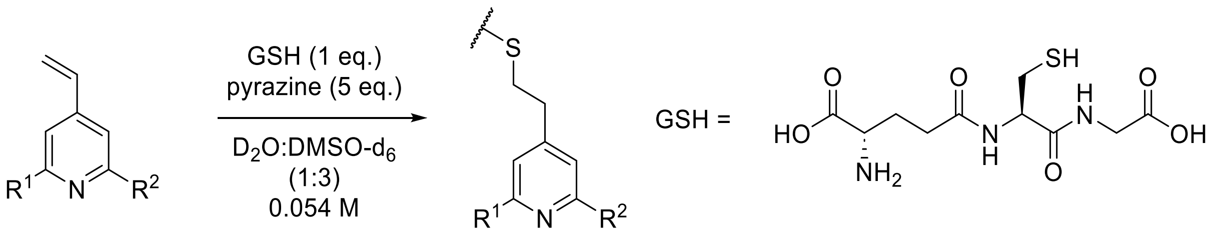

Vinylpyridine (0.033 mmol) and pyrazine (12.8 mg, 0.16 mmol) were taken up in DMSO-d_6 (0.45 mL) and added to an NMR tube. A solution of glutathione (10 mg, 0.033 mmol) in D_2O (0.15 mL) was then added to the same tube. Upon addition, the NMR tube was inverted twice, and the time was noted. The sample was added to the NMR instrument. A ^{1}H-NMR spectrum of the reaction was taken 24 times over 12 h.

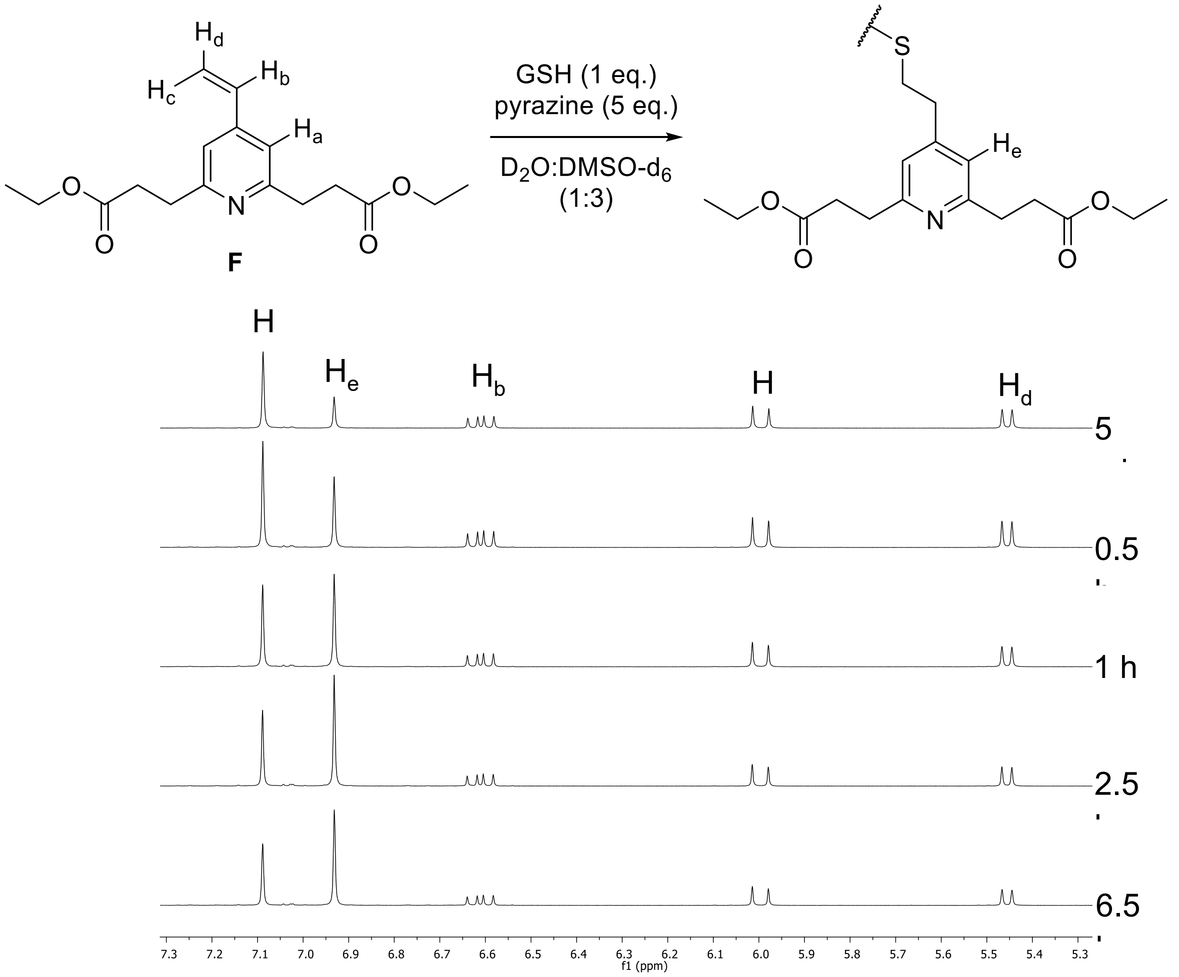

Figure S34. Stacked ^{1}H-NMR spectra of vinyl bis-ester F obtained during glutathione conjugation.

Using pyrazine as a reference, the concentrations of the vinylpyridine and thioether product were determined by integration of the resonances at each time point (Figure 35).

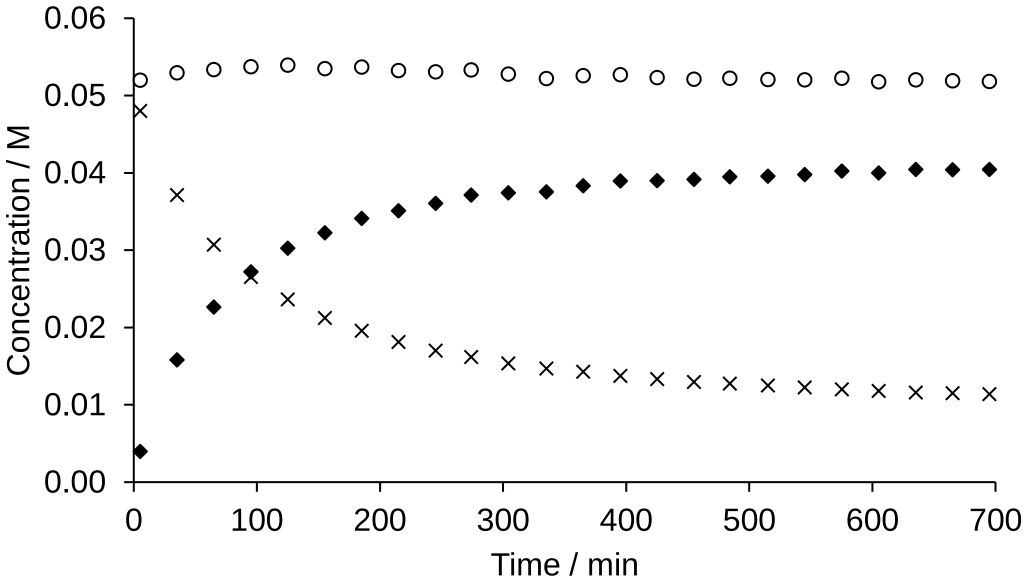

Figure S35. The concentrations of the vinylpyridine starting material bis-ester 90 (×), thioether product 120 (♦), and total (o) against time.

The reciprocal of the concentration of the vinylpyridine starting material was plotted against time, and a linear line of best fit was applied (Figure S36). The gradient of this line was the second order rate constant (k_{obs}).

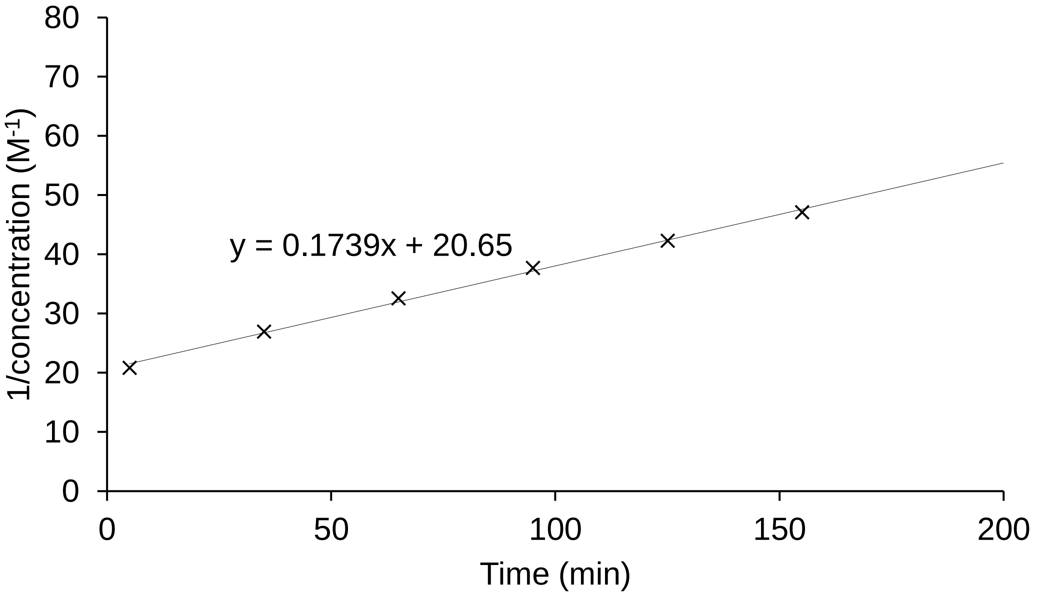

Figure S36. Reciprocal concentration of bis-ester F against time in GSH reaction**.**

The rate quoted in Table 3 for **L** is taken from Reference 71.

From these data (Figure 36), it can be seen that vinyl bis-ester **F** gave a second order rate constant of 0.17 M^{-1} min^{-1}. The second order nature of this reaction has also been confirmed in the literature.[72, 73, 74]

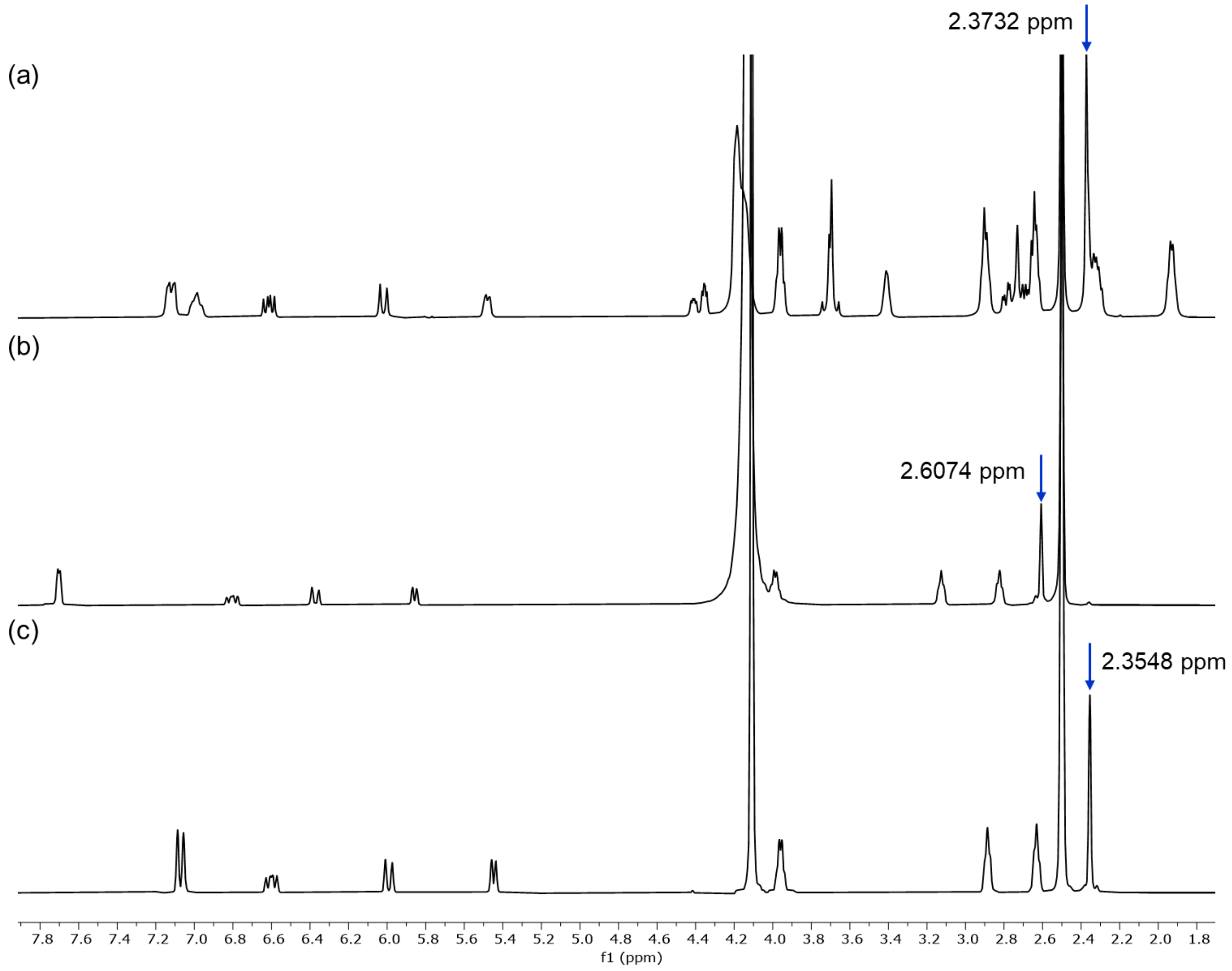

Figure S37. Determination of the degree of protonation of **A** under conditions of the reaction with glutathione. (a) ^{1}H NMR spectrum of reaction mixture for glutathione conjugation described above (Figure S34). (b) ^{1}H NMR spectrum of **A** in reaction mixture, but without GSH, acidified with 20 mM HCl. (c) ^{1}H NMR spectrum of **A** in reaction mixture, but without GSH or HCl. Spectra have been processed with an exponential line broadening factor of 1 Hz and referenced to residual signal of DMSO-d_6 (2.50 ppm). The methyl resonance of **A** is indicated with a blue arrow.

The chemical shift of the methyl resonance of the fully protonated and deprotonated forms of **A** under the conditions of the reaction were determined as 2.6074 ppm and 2.3548 ppm, respectively (Figure S37b,c). The uncertainty in these limiting chemical shifts, Δ_{lim}, in the sample of Figure S37b is taken as 0.005 ppm while the uncertainty in the measurement of a chemical shift, Δ_{obs}, is taken as 0.0005 ppm.[55,79] The fraction of protonation of **A** in the

presence of GSH under the conditions of the reaction (Figure S37a) is thus determined as $1 - \frac{(2.3732 \pm \Delta_{\rm obs}) - (2.6074 \pm \Delta_{\rm lim})}{(2.3548 \pm \Delta_{\rm lim}) - (2.6074 \pm \Delta_{\rm lim})} = 0.073 \pm 0.015$,[55] with the uncertainty estimated as half the difference between the maximum and minimum values calculated by addition or subtraction of $\Delta_{\rm lim}$ and $\Delta_{\rm obs}$ from the measured chemical shifts in the calculation. We note that the relative pK_a values of **A** – **M** are likely to be similar in 75 vol% DMSO-d$_6$/D$_2$O as in aqueous solution.[79,80] We performed calculations (Figure S38) of the pH and percentage of 4-vinylpyridine derivative in the pronated state in aqueous solutions of glutathione (0.054 M) and 4-vinylpyridine derivative (0.054 M) using the CurTiPot package.[50] The percentage of **A** in the protonated state was calculated as 8.2% in good agreement with the value determined experimentally in 75 vol% DMSO-d$_6$/D$_2$O.

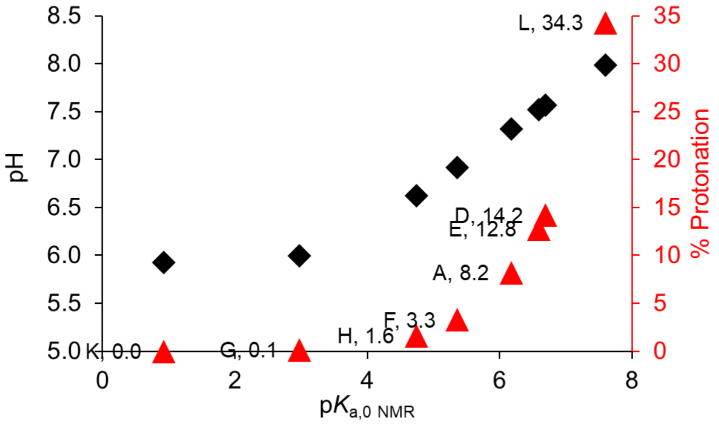

Figure S38. Plot of pH (black diamond) and percentage of 4-vinylpyridine derivative in protonated state (red triangle) in aqueous solutions of glutathione (0.054 M) and 4-vinylpyridine derivative (0.054 M) calculated using the CurTiPot package[50] and p$K_{a,0\ NMR}$ values provided in Table 3. The pK_a values of glutathione were taken as 9.62, 8.66, 3.53 and 2.12.[81]

S13. References

49. R. W. Adams, C. M. Holroyd, J. A. Aguilar, M. Nilsson and G. A. Morris, *ChemComm.*, 2013, **49**, 358-360.
50. I. G. R. Gutz, *CurTiPot −pH and Acid–base Titration Curves: Analysis and Simulation Freeware, Version 4.2*, http://www.iq.usp.br/gutz/Curtipot_.html.
51. M. Wallace, D. J. Adams and J. A. Iggo, *Anal. Chem.*, 2018, **90**, 4160-4166.
52. D. E. Ames and T. F. Grey, *J. Cherm. Soc.*, 1955, DOI: 10.1039/JR9550000631, 631-636.
53. H. C. Brown, S. Johnson and H. Podall, *J. Am. Chem. Soc.*, 1954, **76**, 5556-5557.
54. P. Trigo-Mouriño, C. Merle, M. R. M. Koos, B. Luy and R. R. Gil, *Chem. Eur. J.*, 2013, **19**, 7013-7019.
55. M. Wallace, K. Lam, A. Kuraite and Y. Z. Khimyak, *Anal. Chem.*, 2020, **92**, 12789-12794.
56. A. D. Bochevarov, M. A. Watson, J. R. Greenwood and D. M. Philipp, *J. Chem. Theory Comput.*, 2016, **12**, 6001-6019.

57. H. S. Yu, M. A. Watson and A. D. Bochevarov, *J. Chem. Inf. Model.*, 2018, **58**, 271-286.
58. J. J. Klicić, R. A. Friesner, S. Y. Liu and W. C. Guida, *J Phys Chem A*, 2002, **106**, 1327-1335.
59. F. Mohamadi, N. G. Richard, W. C. Guida, R. Liksamp, M. Lipton, C. Caufield, G. Chang, T. Henrickson and W. C. Still, *J. Comput. Chem*., 1990, **11**, 440–467.
60. K. S. Watts, P. Dalal, A. J. Tebben, D. L. Cheney and J. C. Shelley, *J. Chem. Inf. Model.*, 2014, **54**, 2680–2696.
61. M. H. Bolli, S. Abele, M. Birker, R. Bravo, D. Bur, R. De Kanter, C. Kohl, J. Grimont, P. Hess, C. Lescop, B. Mathys, C. Müller, O. Nayler, M. Rey, M. Scherz, G. Schmidt, J. Seifert, B. Steiner, J. Velker and T. Weller, *J. Med. Chem.*, 2014, **57**, 110–130.
62. W. L. Lee, T. W. Hsu, W. C. Hung and J. M. Fang, *Dalt. Trans.*, 2019, **48**, 8026–8029.
63. A. L. Bradley, S. Izenwasser, D. Wade, C. Klein-Stevens, N. Zhu and M. L. Trudell, *Bioorganic Med. Chem. Lett.*, 2002, **12**, 2387–2390.
64. [a] J. Romero-Ibañez, S. Cruz-Gregorio, L. Quintero and F. Sartillo-Piscil, *Synth.*, 2018, **50**, 2878–2886. [b] WO2008125839A2, 2008.
65. M. P. Smolinski, Y. Bu, J. Clements, I. H. Gelman, T. Hegab, D. L. Cutler, J. W. S. Fang, G. Fetterly, R. Kwan, A. Barnett, J. Y. N. Lau and D. G. Hangauer, *J. Med. Chem.*, 2018, **61**, 4704–4719.
66. D. Posevins, M. B. Li, E. Svensson Grape, A. K. Inge, Y. Qiu and J. E. Bäckvall, *Org. Lett.*, 2020, **22**, 417–421.
67. H. J. Davis, M. T. Mihai and R. J. Phipps, *J. Am. Chem. Soc.*, 2016, **138**, 12759–12762.
68. Y. C. Lin, K. H. Yu, Y. F. Lin, G. H. Lee, Y. Wang, S. T. Liu and J. T. Chen, *Dalt. Trans.*, 2012, **41**, 6661–6670.
69. O. Hassan Omar, F. Babudri, G. M. Farinola, F. Naso and A. Operamolla, *European J. Org. Chem.*, 2011, **2011**, 529–537.
70. M. Rovira, L. Jašíková, E. Andris, F. Acuña-Parés, M. Soler, I. Güell, M. Z. Wang, L. Gómez, J. M. Luis, J. Roithová and X. Ribas, *ChemComm*, 2017, **53**, 8786–8789.
71. J. Andrew, *PhD Thesis*, The University of East Anglia, 2022.
72. H. Seki, S. J. Walsh, J. D. Bargh, J. S. Parker, J. Carroll and D. R. Spring, *Chem. Sci.*, 2021, **12**, 9060–9068.
73. M. J. Matos, C. D. Navo, T. Hakala, X. Ferhati, A. Guerreiro, D. Hartmann, B. Bernardim, K. L. Saar, I. Compañón, F. Corzana, T. P. J. Knowles, G. Jiménez-Osés and G. J. L. Bernardes, *Angew. Chemie - Int. Ed.*, 2019, **58**, 6640–6644
74. P. Ochtrop and C. P. R. Hackenberger, *Curr. Opin. Chem. Biol.*, 2020, **58**, 28–36.
75. A. T. Londregan, S. Jennings and L. Wei, *Org. Lett.*, 2011, **13**, 1840–1843.
76. A. Gero and J. J. Markham, *J. Org. Chem.*, 1951, **16**, 1835–1838.
77. J. F. King, J. H. Hillhouse and S. Skonieczny, *Can. J. Chem.*, 1984, **62**, 1977-1995.
78. Y. Kitamura and T. Itoh, *J. Soln. Chem.*, 1987, **16**, 715-725.
79. M. Wallace, N. Abiama and M. Chipembere, *Anal. Chem.*, 2023, **95**, 15628–15635.
80. J.-C. Hallé, J. Lelievre, and F. Terrier, *Can. J. Chem.*, 1996, **74**, 613-620.
81. N. W. Pirie and K. G. Pinhey, *J. Biol. Chem.,* 1929, **84**, 321-333.

S14. Processing routines, scripts and pulse programs

14.1 Processing routine for Mnova 14.3.1

14.1.1 Extraction of indicator chemical shifts to find pH

Run this macro script in Bruker Topspin using edmac command:

```
# Sets up a CSI dataset for opening and processing in Mnova
# Sets SI to 128 and 32768, LB 3, phase sensitive in both dimensions (adjust if not appropriate)
#This AU is not fully tested and comes without warranty
#The script works on Bruker Topspin 3.6.2 but has not been tested on other versions
#Matthew Wallace, 1/2023
#University of East Anglia, matthew.wallace@uea.ac.uk
1 SI 128
2 SI 32768
2 LB 3
2 WDW EM
1 WDW SINE
2 PHC1 0
2 PHC0 0
2 PH_mod pk
1 PH_mod pk
#1 PHC1 should be 180*number of gradient points acquired
1 PHC1 23040
XFB
#Now open 2rr file from procno folder into Mnova
```

Run processing template, defining cut around the residual water signal (4.5-5.5 ppm), to phase, baseline correct and reference spectra of CSI dataset.

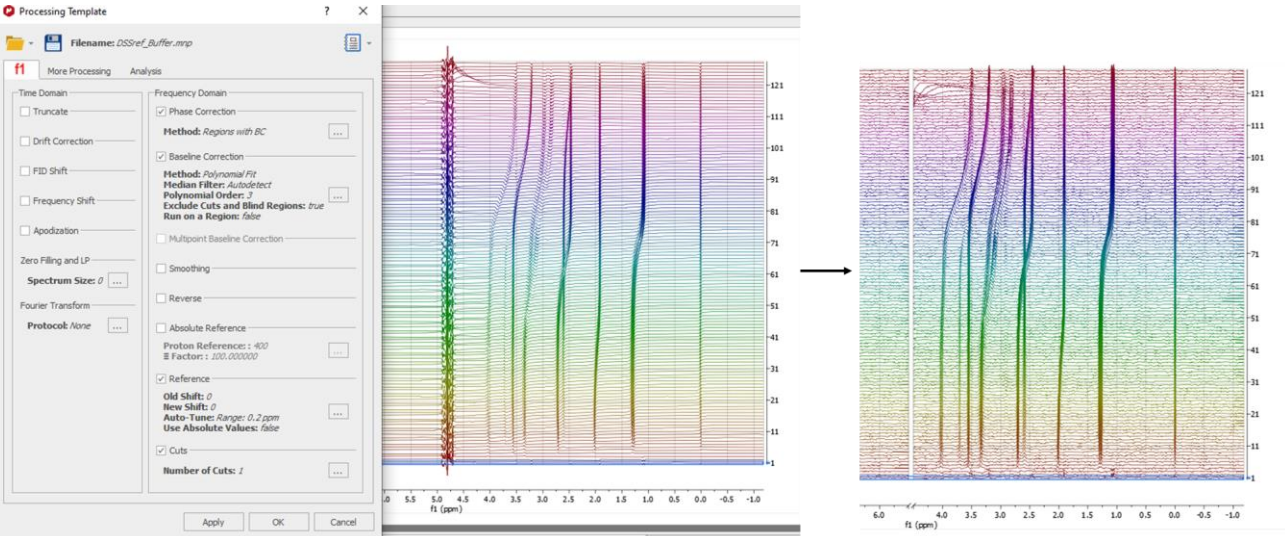

In data analysis tab, define regions to find max peak position of DSS, DCA, MPA, formate, acetate, 2,6-lutidine, NHS, MPA, glycine and methylamine sequentially based on parameters in Table S1. Peak picking options may need to be adjusted. Data analysis table may then be copied and pasted into spreadsheet

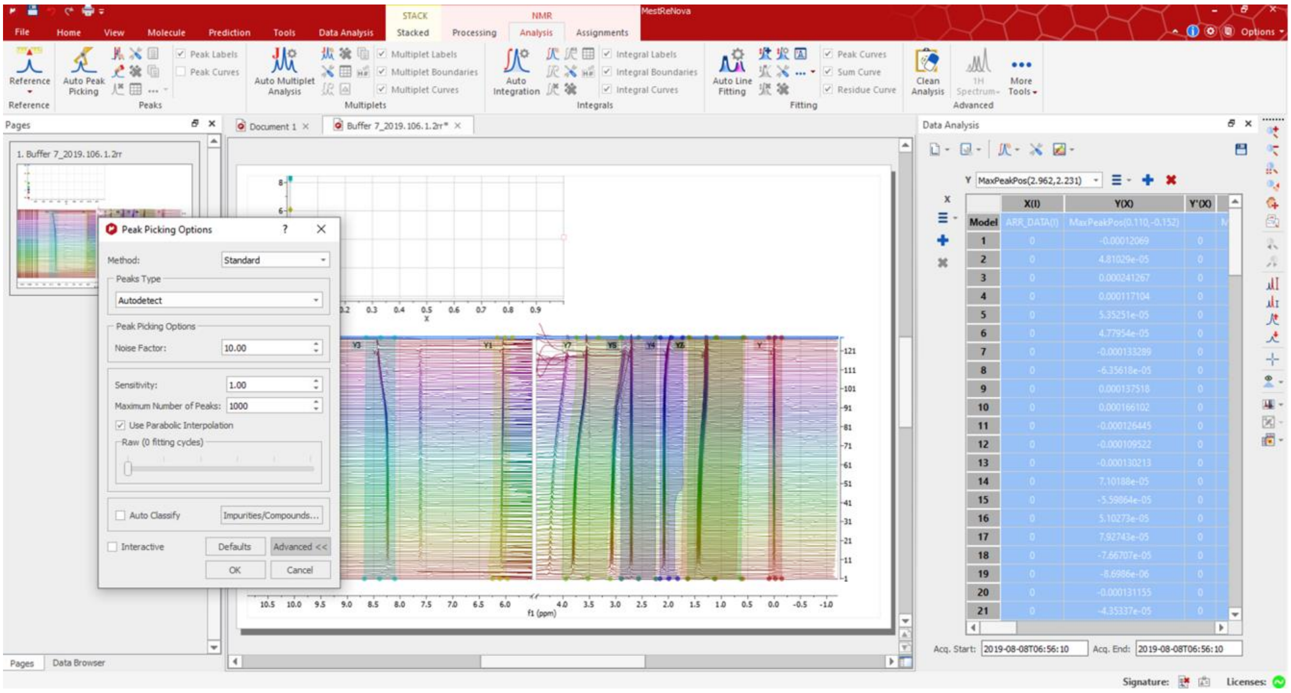

For MPA, it is necessary to find the average chemical shift of the doublet by line fitting the region. Running the script DoubletChemShift, below, will find and export the chemical shift of MPA to a text file which should be copied over the MPA from the peak picking above.

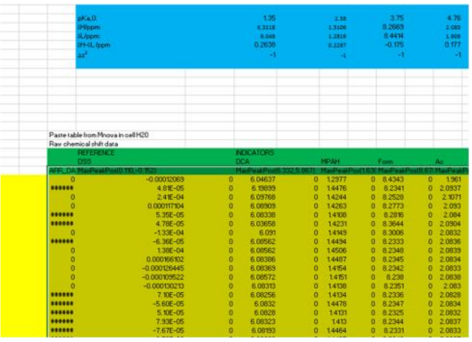

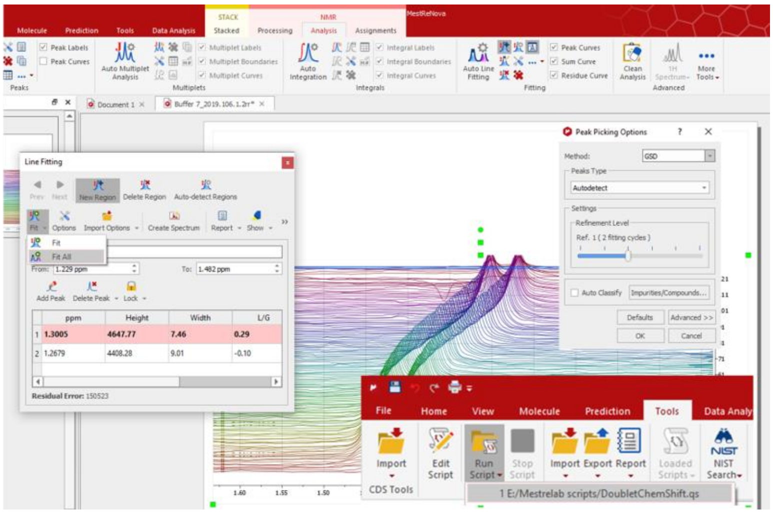

```
/**********************************************************************************************
For finding central chemical shift of multiplet (doublet or quartet) in stacked plot from Chemical Shift Imaging (CSI) dataset
Save this script in Mnova as DoubletChemShift, and Run this script
Define line fitting area with new fit region (clear all previous line fitting regions)
Click fit to fit all spectra in the stacked CSI dataset
Run this script

Script will find the most intense peak within the defined region of a spectrum
Will then find the most upfield and downfield peaks in the region with intensities within sens of biggest peak
Doublet or quartet chemical shift can then be copied and pasted into Excel

Matthew Wallace, University of East Anglia, 01/2023 (matthew.wallace@uea.ac.uk)

Based on Mnova script exportFitRegions (Copyright (C) 2014 Mestrelab Research S.L. All rights reserved, part of the Mnova scripting toolkit).
(Authorized users of Mnova Software may use this file freely, but this file is provided AS IS)
with NO WARRANTY OF ANY KIND, INCLUDING THE WARRANTY OF DESIGN, MERCHANTABILITY AND FITNESS
FOR A PARTICULAR PURPOSE.
*******************************************************************************************/
/*globals settings, Dir, FileDialog, File, TextStream, Application, NMRSpectrum, print, Peak, MnUi*/
/*jslint plusplus: true, indent: 4*/

function DoubletChemShift() {
"use strict";
```

```
function fitRegionToStream(aFitRegion, aFileStream, aNMRPeaks) {
var p, peak, tst, big, sens, tstppm, bigppm, smlppm, dbppm
sens=0.6;
tst=0;
big=0;
tstppm=0;
bigppm=-100;
fitPeaksIds = aFitRegion.peaks;
/*Find most intense peak in fitted region*/
for (p = 0; p < fitPeaksIds.length; p++) {
peak = new Peak(aNMRPeaks.byId(fitPeaksIds[p]));
tst=peak.intensity;
if(tst>big)
{
big=peak.intensity;
bigppm=peak.delta(1);
}
}
/*Find peak with highest chemical shift with intensity within sens of biggest peak*/
for (p = 0; p < fitPeaksIds.length; p++) {
peak = new Peak(aNMRPeaks.byId(fitPeaksIds[p]));
tst=peak.intensity;
tstppm=peak.delta(1);
if(tst>sens*big)
{
if(tstppm>bigppm)
{
bigppm=peak.delta(1);
}
}
}
smlppm=bigppm;
/*Find peak with most upfield chemical shift with intensity within sens of biggest peak*/
for (p = 0; p < fitPeaksIds.length; p++) {
peak = new Peak(aNMRPeaks.byId(fitPeaksIds[p]));
tst=peak.intensity;
tstppm=peak.delta(1);
if(tst>sens*big)
{
if(tstppm<smlppm)
{
smlppm=peak.delta(1);
}
}
}
dbppm=(bigppm+smlppm)/2;
aFileStream.write(dbppm, "\n");
}

var fout, sout, spc, peakList, fitRegions, fr, oldCurSpecIndex, i,
dirSettingsKey = "DoubletChemShift/LastDir",
saveDir = settings.value(dirSettingsKey, Dir.home()),
dw = Application.mainWindow.activeDocument,
spectra = dw.itemCount("NMR Spectrum"),
specIndex = 0,
fileName = FileDialog.getSaveFileName("ASCII Files (*.txt)", "", saveDir);

if (!fileName.length) {
return;
}

fout = new File(fileName);
settings.setValue(dirSettingsKey, fout.absDirPath);
if (!fout.open(File.WriteOnly)) {
throw "Impossible to open file";
}
sout = new TextStream(fout);
sout.precision = 10;

while (specIndex < spectra) {
spc = new NMRSpectrum(dw.item(specIndex, "NMR Spectrum"));
specIndex++;
if (!spc.isValid()) {
throw "Invalid Spectrum";
}
oldCurSpecIndex = spc.curSpecIndex;
for (i = 0; i < spc.specCount; i++) {
spc.curSpecIndex = i;
peakList = spc.peaks();
fitRegions = spc.fitRegions();
print(fitRegions);
for (fr = 0; fr < fitRegions.length; fr++) {
fitRegionToStream(fitRegions[fr], sout, peakList);
}
}
spc.curSpecIndex = oldCurSpecIndex;
}
fout.close();
}

if (this.MnUi && MnUi.scripts_nmr) {
MnUi.scripts_nmr.scripts_nmr_ExportASCIIFitRegions = DoubletChemShift;
}
```

14.1.2 Extraction of analyte pK_a

Having extracted the pH, the analyte chemical shifts can be extracted using the max peak position tool, starting with two handlers and adjusting as appropriate e.g. to track upfield imidazole and α resonance of histidine.

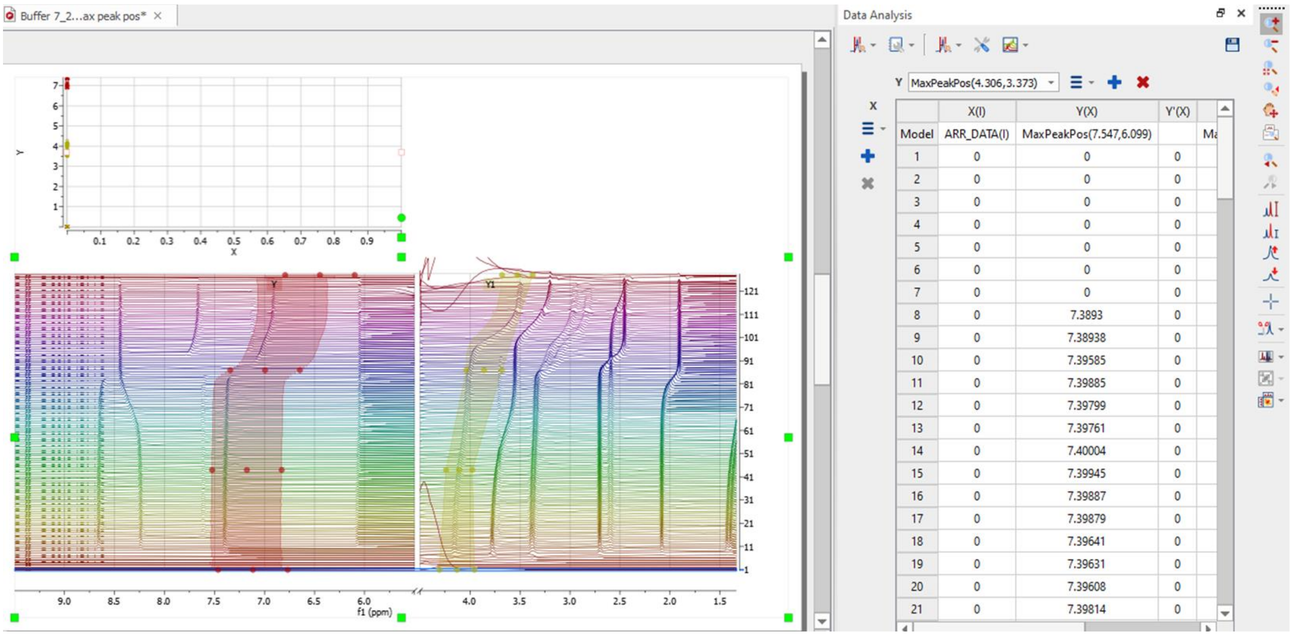

To track the downfield β resonance of histidine, the number of handlers is increased. Each row can be checked using Active Spectrum mode and the peak selection window changed individually as appropriate.

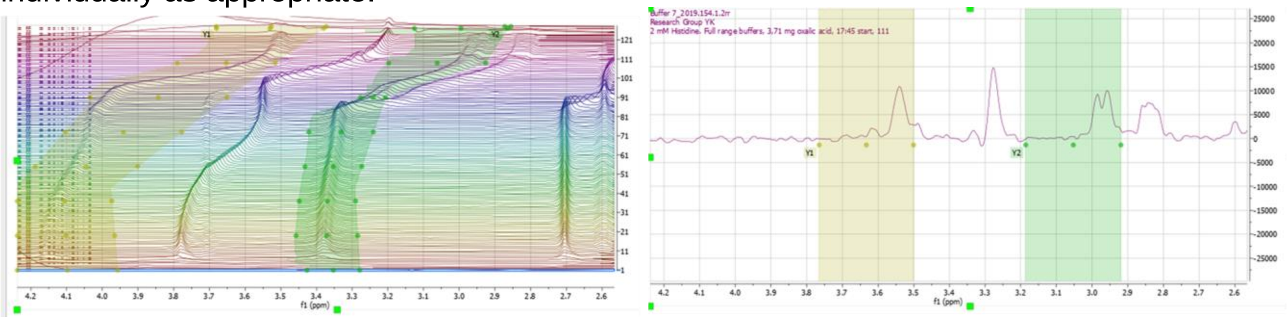

Copy and paste Data Analysis table into spreadsheet. On fitting tab, copy and paste initial guesses into main fitting and uncertainty analysis component. Load appropriate solver model and run to optimise fitting parameters to Equation 1.

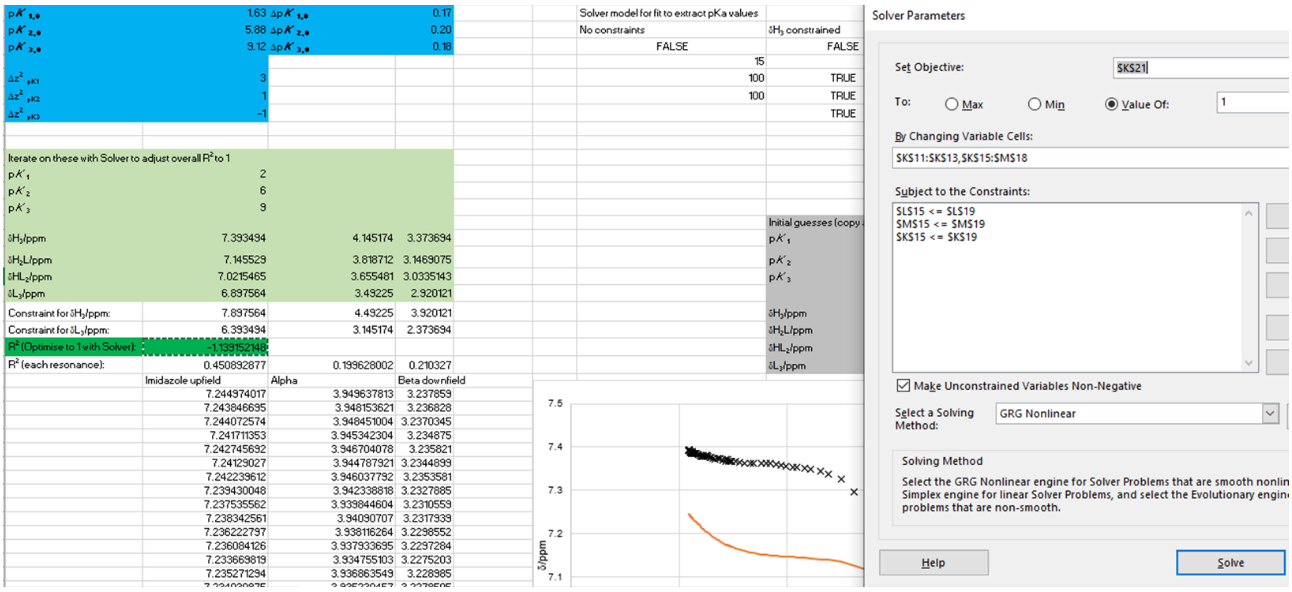

Then load Solver model for uncertainty analysis and optimise the fitting value for the pseudo-experimental datasets.

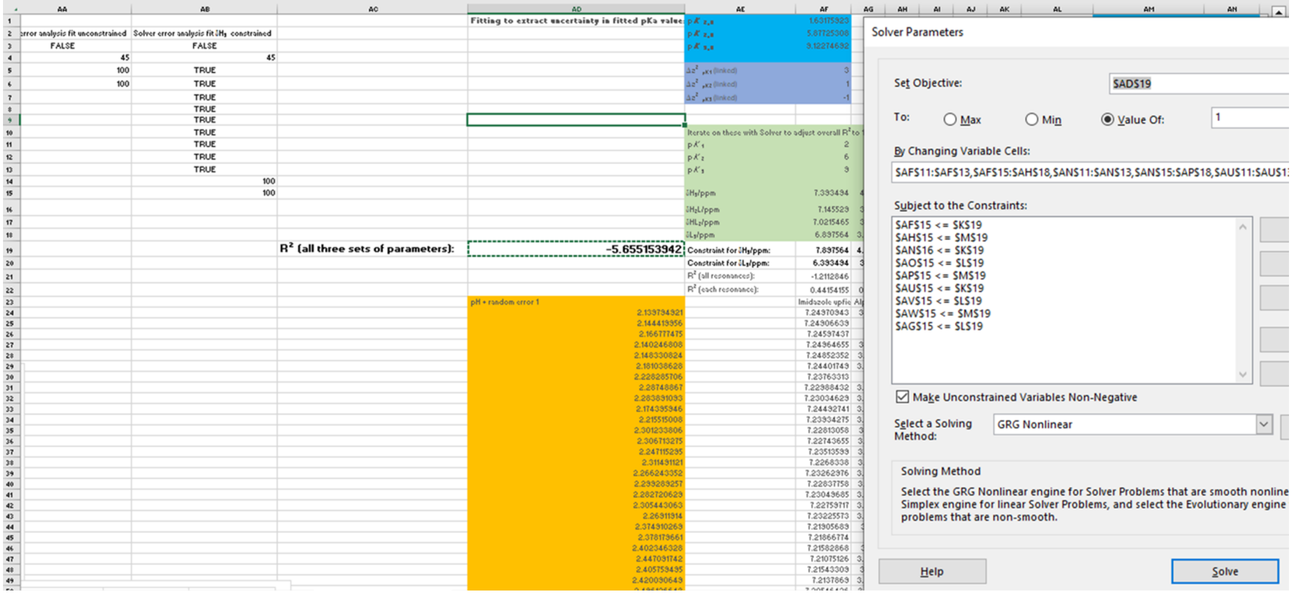

Final p$K_{a,0}$ values and experimental values are provided in blue.

$pK'_{1,\bullet}$	1.39	$\Delta pK'_{1,\bullet}$	0.14
$pK'_{2,\bullet}$	5.82	$\Delta pK'_{2,\bullet}$	0.25
$pK'_{3,\bullet}$	9.13	$\Delta pK'_{3,\bullet}$	0.17
Δz^2_{pK1}	3		
Δz^2_{pK2}	1		
Δz^2_{pK3}	-1		
Iterate on these with Solver to adjust overall R^2 to 1			
pK'_1	1.757974984		
pK'_2	5.94719559		
pK'_3	9.012054243		
δH_3/ppm	7.472917949	4.49225	3.4991803
δH_2L/ppm	7.360198426	4.006147971	3.3220485
δHL_2/ppm	7.016106615	3.954382993	3.1823766
δL_3/ppm	6.892423325	3.484801134	2.9206984
Constraint for δH_3/ppm:	7.897564	4.49225	3.920121
Constraint for δL_3/ppm:	6.393494	3.145174	2.373694
R^2 (Optimise to 1 with Solver):	0.998094281		
R^2 (each resonance):	0.999800321	0.999552582	0.9987414
	Imidazole upfield	Alpha	Beta downfield
	7.391406019	4.140904952	3.3711382
	7.390978636	4.139066137	3.3704678
	7.391064112	4.139433886	3.3706019
	7.390174476	4.135606589	3.3692064
	7.390563129	4.137278539	3.369816
	7.390016722	4.134927979	3.368959
	7.390372765	4.136459596	3.3695174
	7.389323028	4.131944145	3.3678711
	7.388621897	4.128928732	3.3667716

Solver model for fit to extract pKa values	
No constraints	δH_3 constrained
FALSE	FALSE
15	
100	TRUE
100	TRUE
	TRUE
	Initial guesses (copy and pa
	pK'_1
	pK'_2
	pK'_3
	δH_3/ppm
	δH_2L/ppm
	δHL_2/ppm
	δL_3/ppm

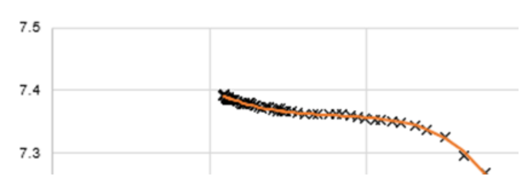

14.2 Scripts to acquire and process CSI datasets (Bruker Topspin)

14.2.1 Script to acquire experiments in 100% H_2O under automation

```
/* AU script for finding water suppression frequency in 100% H2O and running CSI experiment through IconNMR*/
/*Based on standard Bruker script, au_watersc*/
/*Running a 1D proton on a CDCl3 standard sample immediately prior to the CSI experiment can help ensure*/
/*that field is in correct place when this script is run*/
/*create macro for reading in default shim file*/
/*edmac rshim, then in macro text: rsh nameofshimfile*/
/*create macro for topshim on 1h:*/
/*edmac t1h, then in macro text: topshim 1h lockoff o1p=4.85 ordmax=3 convcomp (or as appropriate for your system)*/
/*create a 1 scan proton parameter set (low rg) to find o1p of water, called H2O_SS*/
/*Remember to change peak picking regions in this PAR set to cover the expected range for water signal*/
/*create a CSI parameter set called 1hcsi (or equivalent), this runs the csi experiment*/
/*set parameter AUNM in this par set to the name of this script*/
/*Then add the par set onto your list of experiments in the IconNMR config menu*/
/*when running through Icon, disable locking and shimming by choosing the "IconNMR not responsible" option or define your own solvent*/
/*The script works on Bruker Topspin 3.6.2 but has not been tested on other versions*/
/*This AU is not fully tested and comes without warranty.*/
/*Matthew Wallace, 5/2022*/
/*University of East Anglia, matthew.wallace@uea.ac.uk*/
float peakFreqHz, peakFreqPPM, peakIntensity, maxpsh, maxpsp, maxips, rd;
char path[PATH_MAX];
double sf, sfo1, sppm;
int pscal_save, i, numPeaks, noofscans;
GETCURDATA
FETCHPAR("NS",&noofscans)
FETCHPAR("d1",&rd)
FETCHPAR("sw",&sppm)
/*Can set number of scans, sw and d1 in ICON. All other parameters will be overwritten at end*/
RPAR("H2O_SS","all")
/*STOREPAR("RG",1)*/
XMAC("rshim")
sleep(60);
XMAC("t1h")
sleep(90);
ZG
ERRORABORT
EF
ERRORABORT
APK
```

```
FETCHPAR("PSCAL",&pscal_save)
STOREPAR("PSCAL",0)
PP
ERRORABORT
strcpy(path, PROCPATH(0));
numPeaks = readPeakList(path);
maxips=0.0;
maxpsh=0.0;
for (i=0; i<numPeaks; i++)
{
peakIntensity = getPeakIntensity(i);
peakFreqHz = getPeakFreqHz(i);
peakFreqPPM = getPeakFreqPPM(i);
if (peakIntensity > maxips)
{
maxips = peakIntensity;
maxpsh = peakFreqHz;
maxpsp = peakFreqPPM;
}
}
freePeakList();
FETCHPAR("SF",&sf);
sfo1 = sf + maxpsh * 1.0e-6;
STOREPAR("SFO1",sfo1);
SETCURDATA
/*RGA can go wrong for water suppression*/
RPAR("1hcsi","all")
STOREPAR("SFO1",sfo1)
STOREPAR("NS",noofscans)
STOREPAR("sw",sppm)
STOREPAR("d1",rd)
ZG
QUIT
```

14.2.2 Script to automatically phase and baseline correct CSI dataset

```
/*To produce phase corrected chemical shift image from gradient encoded data*/
/*Will check that region between 4 and -1 ppm contains positive NMR signals by integration*/
/*The residual water signal can fool the APK/APKS commands*/
/*If integral of region 4 to -1 ppm is negative, a 180 degree zero order phase shift will be applied to the row*/
/*Must create a integral file called csicheck using rmisc command*/
/*Set 1st order phase correction for f1 dimension to 180*Td1 (11520 when 64 points in image), 0th order to 0*/
/*PH_mod should be set to PK in both dimensions*/
/*LB3, XFB to produce image*/
/*This is done automatically by script 13.2 above*/
/*With the 2D dataset selected, Run this Au*/
/*AU extracts each row in turn to a procno and automatically phase and baseline corrects/*
/*Reversal of F1 axis may be necessary, depending on NMR probe*/
/*This AU is not fully tested and comes without warranty.*/
/*The script works on Bruker Topspin 3.6.2 but has not been tested on other versions*/
/*Use kill command if all goes wrong*/
/*Matthew Wallace, 1/2023*/
/*University of East Anglia, matthew.wallace@uea.ac.uk*/
FILE *fpnt;
char disk1[32], user1[32], location[128], phtyp[8],chkans[8];
char dummystr[256],intresult[256];
double intval, leftlim, rightlim;
float abf1=8;
float abf2=6;
float inv=180;
float phu=0;
int phpno=1;
int w=1;
int np=64;
int pno=5;
int intnumber=1;
GETCURDATA
int steno=expno;
strcpy(location,disk);
strcpy(phtyp,"k");
strcpy(chkans,"y");
GETSTRING("Enter location of dataset",location)
phpno=procno;
GETINT("Enter experiment number to process",steno)
GETINT("Enter procno containing XFB processed 2D data :",phpno)
pno=phpno+5;
GETINT("Enter procno to write rows to phase and baseline correct (empty):",pno)
REXPNO(steno)
RPROCNO(phpno)
SETCURDATA
FETCHPAR1("SI",&np)
GETINT("Enter number of points in image (autodetects) :",np)
GETSTRING("APKS (s) or APK (k) or apkf (f) auto phase correction?",phtyp)
if(strcmp(phtyp,"f")==0)
{
GETFLOAT("Enter right limit for apkf and absf:",abf2)
GETFLOAT("Enter left limit for apkf and absf:",abf1)
strcpy(chkans,"n");
}
else
{
GETSTRING("Check spectra are correct way up? (Need to create integral file called csicheck 4 to -1ppm)",chkans)
}
if(strcmp(chkans,"y")==0)
{
STOREPAR("CURPRIN","integrals.txt")
}
w=1;
TIMES(np)
{
RPROCNO(phpno)
SETCURDATA
RSR(w,pno)
RPROCNO(pno)
SETCURDATA
if(strcmp(phtyp,"s")==0)
{
APKS
ABS
}
if(strcmp(phtyp,"k")==0)
{
APK
ABS
}
if(strcmp(phtyp,"f")==0)
{
```

```
STOREPAR("absf1",abf1)
STOREPAR("absf2",abf2)
APKF
ABSF
}
/*If upside down puts write way up before writing back to 2D procno*/
if(strcmp(chkans,"y")==0)
{
/*              STOREPAR("intscl",inscl)*/
RMISC("intrng", "csicheck")
LI
strcpy (intresult,PROCPATH("integrals.txt"));
fpnt=fopen(intresult, "r");
(void) fseek (fpnt, 0L, SEEK_SET);
  fgets(dummystr, sizeof(dummystr), fpnt);
while (fgets(dummystr, sizeof(dummystr), fpnt) != NULL)
  (void) sscanf(dummystr,"%d %lf %lf %lf",
                                        &intnumber, &leftlim, &rightlim, &intval);
                                        fclose (fpnt);
                                        if(intval<0)
{
/*Puts additional 180 degree zeroth order phase on whatevers there to begin*/
STOREPAR("phc0",phu)
STOREPAR("phc1",phu)
STOREPAR("PH_mod",1)
PK
STOREPAR("phc0",inv)
PK
ABS
}
}
WSR(w,phpno,steno,name,user,location)
w++;
}
END
QUIT
```

14.2.3 Script to extract peak position of a multiplet

```
/*Bruker AU script for extracting peak positions of a multiplet resonance CSI dataset*/
/*CSI dataset should have been fully processed in phase-sensitive mode*/
/*The script works on Bruker Topspin 3.6.2 but has not been tested on other versions*/
/*The script extracts each row in turn into the empty procno requested (will overwrite existing contents!!!)*/
/*Peak picking routine will find the centre of a multiplet (doublet, quartet, or singlet with bad shim)*/
/*Will treat two peaks as outer edges of a multiplet if their intensity is within ppsens of the biggest peak found in specified range*/
/*This way it will pick the centre of a doublet/quartet, as well as triplet, quintet etc.*/
/*Adjust peak picking ranges as appropriate to avoid overlap*/
/*To use this script, the component of interest must be the most intense signal in the specified range*/
/*Will write peak position/ppm in procno directory of CSI dataset*/
/*These numbers go from first to final row of the CSI dataset*/
/*Numbers can be copied and pasted into spreadsheet*/
/*This AU is not fully tested and comes without warranty*/
/*Use kill command if all goes wrong*/
/*Matthew Wallace, 1/2023*/
/*University of East Anglia, matthew.wallace@uea.ac.uk*/
FILE *fcmp;
float min=0;
double f2pcmp=1;
double f1pcmp=2;
float ppsens=0.9;
double pc=0.1;
int steno=15;
int eno;
int ne=5;
int m=-1;
double peakFreqHz, peakFreqPPM, peakIntensity, maxpsh, maxpsp, maxips, sf,sfo1,so1p;
double mintpp,minpsp,peakppmneg,cent,ppmdif,maxpspneg;
int i, numPeaks;
int np=128;
int row=1;
int v=1;
int phpno=1;
int wrpno=5;
GETCURDATA
steno=expno;
phpno=procno;
wrpno=procno+5;
FETCHPAR1("td",&np)
GETINT("Enter experiment number: ",steno)
GETINT("Enter number of gradient values",np)
GETINT("Enter procno of 2D dataset",phpno)
GETINT("Enter procno to extract rows into for peak picking (blank)",wrpno)
GETDOUBLE("Right peak picking/ppm",f2pcmp)
```

```
GETDOUBLE("Left peak picking/ppm",f1pcmp)
GETFLOAT("Enter peak picking senistitivty factor",pc)
GETDOUBLE("Enter satelite sensitivity factor",ppsens)
/*Reads in peak picking parameters for cmp*/
REXPNO(steno)
SETCURDATA
RPROCNO(phpno)
SETCURDATA
STOREPAR("mi",min)
STOREPAR("pc",pc)
if ((fcmp = fopen(PROCPATH("Selected peak ppm.txt"),"wt")) == 0)
STOPMSG("Cannot create text file")
/*Now extract each row in turn into requested procno, peak positino of each species and write positions to file*/
TIMES(np)
{
RSR(v,wrpno)
RPROCNO(wrpno)
SETCURDATA
/*Extract cmp*/
STOREPAR("f2p",f2pcmp)
STOREPAR("f1p",f1pcmp)
PP
 numPeaks = readPeakList(PROCPATH(0));
maxips=0.0;
    maxpsh=0.0;
    for (i=0; i<numPeaks; i++)
    {
                peakIntensity = getPeakIntensity(i);
                peakFreqHz = getPeakFreqHz(i);
                peakFreqPPM = getPeakFreqPPM(i);
                if (peakIntensity > maxips)
                {
                    maxips = peakIntensity;
                    maxpsh = peakFreqHz;
                    maxpsp = peakFreqPPM;
                }
    }
    /*Pick most downfield side of multiplet*/
    mintpp=maxips*ppsens;
maxpsp=0.0;
    for (i=0; i<numPeaks; i++)
    {
                        peakIntensity = getPeakIntensity(i);
                        if(peakIntensity>mintpp)
                        {
                peakFreqPPM = getPeakFreqPPM(i);
                peakFreqHz = getPeakFreqHz(i);
                if (peakFreqHz >= maxpsh)
                {
                    maxpsp = peakFreqPPM;
                    maxpsh = peakFreqHz;
                }
    }
                }
/*Flips negative to choose most upfield peak of multiplet*/
for (i=0; i<numPeaks; i++)
    {
                        peakIntensity = getPeakIntensity(i);
                        if(peakIntensity>mintpp)
                        {
                peakFreqPPM = getPeakFreqPPM(i);
peakppmneg=peakFreqPPM*m;
maxpspneg=maxpsp*m;
                if (peakppmneg >= maxpspneg)
                {
                    minpsp = peakFreqPPM;
                }
    }
                }
    freePeakList();
/*writes centre of multiplet into text document*/
ppmdif=maxpsp-minpsp;
cent=minpsp+ppmdif*0.5;
    fprintf(fcmp,"%f \n",cent);
/*Now advance row counter and repeat for the next row*/
v++;
RPROCNO(phpno)
SETCURDATA
}
END
fclose(fcmp);
QUIT
```

14.2.4 Script to track the position of a multiplet through a CSI dataset

```
/*Bruker AU script for extracting peak positions of a multiplet resonance CSI dataset*/
/*This script adjusts the searching window according to the peak position detected in the previous row*/
/*It can thus track the position on a doublket through a CSI dataset*/
/*even if another resonance occupies same chemical shift window within the dataset*/
/*CSI dataset should have been fully processed in phase-sensitive mode*/
/*The script works on Bruker Topspin 3.6.2 but has not been tested on other versions*/
/*Start on a row containing useable data (e.g. 20), give the position of the resonance in that row*/
/*Then specify the maximum change in the chemical shift of that resonance between two sequential slices*/
/*Will write peak position/ppm in procno directory of CSI dataset*/
/*These numbers go from thye first row specified to final row of the CSI dataset*/
/*Numbers can be copied and pasted into spreadsheet*/
/*This AU is not fully tested and comes without warranty*/
/*Use kill command if all goes wrong*/
/*Matthew Wallace, 1/2023*/
/*University of East Anglia, matthew.wallace@uea.ac.uk*/
FILE *fcmp;
char pksf[20];
float min=0;
double f2pcmp=3.2;
double f1pcmp=3.7;
double tarinit=3.4;
double tardifac;
double tardifppm=0.05;
double tardifacsq, tardifppmsq,tar,tardifneg,f1pcmpvar,f2pcmpvar;
float ppsens=0.9;
double pc=0.1;
int steno=15;
int strow=20;
int endrow=128;
int eno;
int ne=5;
int m=-1;
double peakFreqHz, peakFreqPPM, peakIntensity, maxpsh, maxpsp, maxips, sf,sfo1,so1p,mintpp,minpsp,peakppmneg,cent,ppmdif,maxpspneg;
double maxpshr, maxpspr, maxipsr,maxpshl, maxpspl, maxipsl;
int i, numPeaks;
int np=128;
int row=1;
int v=1;
int phpno=1;
int wrpno=5;
GETCURDATA
steno=expno;
phpno=procno;
wrpno=procno+6;
FETCHPAR1("td",&np)
GETINT("Enter experiment number to process",steno)
GETINT("Enter procno of 2D dataset",phpno)
GETINT("Enter procno to extract rows into for peak picking (blank)",wrpno)
GETINT("Enter starting row: ",strow)
GETINT("Enter final row: ",endrow)
np=endrow-strow+1;
GETINT("Enter total number of rows to process",np)
/*Reads in peak picking parameters for selected component*/
REXPNO(steno)
SETCURDATA
RPROCNO(phpno)
SETCURDATA
STOREPAR("f2p",f2pcmp)
STOREPAR("f1p",f1pcmp)
GETFLOAT("Enter peak picking senistitivty factor",pc)
GETDOUBLE("Enter satelite sensitivity factor",ppsens)
GETDOUBLE("Enter inital target peak position/ppm",tarinit)
tar=tarinit;
GETDOUBLE("Enter target peak variation/ppm",tardifppm)
f1pcmpvar=tar+tardifppm;
f2pcmpvar=tar-tardifppm;
GETDOUBLE("Target right peak picking/ppm",f2pcmpvar)
GETDOUBLE("Target left peak picking/ppm",f1pcmpvar)
/*GETSTRING("Pick pick rightmost (r), leftmost (l) peak or middle (m) within range?",rlans)*/
STOREPAR("f2p",f2pcmpvar)
STOREPAR("f1p",f1pcmpvar)
STOREPAR("pc",pc)
STOREPAR("mi",min)
if ((fcmp = fopen(PROCPATH("Tracked chemical shift ppm.txt"),"wt")) == 0)
   STOPMSG("Cannot create text file")
   v=strow;
TIMES(np)
{
RSR(v,wrpno)
RPROCNO(wrpno)
SETCURDATA
```

```
/*Find most intense peak in range*/
PP
 numPeaks = readPeakList(PROCPATH(0));
maxips=0.0;
    maxpsh=0.0;
    maxpsp=0.0;
    for (i=0; i<numPeaks; i++)
    {
                peakIntensity = getPeakIntensity(i);
                peakFreqHz = getPeakFreqHz(i);
                peakFreqPPM = getPeakFreqPPM(i);
                if (peakIntensity > maxips)
                {
                    maxips = peakIntensity;
                    maxpsh = peakFreqHz;
                    maxpsp = peakFreqPPM;
                }
    }
/*Pick all peaks within window that are within ppsens of max*/
/*Find peaks that are within target region*/
maxpspl=maxpsp;
maxpspr=maxpsp;
    mintpp=maxips*ppsens;
     for (i=0; i<numPeaks; i++)
    {
                            peakIntensity = getPeakIntensity(i);
                            if(peakIntensity>mintpp)
                            {
                peakFreqPPM = getPeakFreqPPM(i);
                peakFreqHz = getPeakFreqHz(i);
                            /*Pick most intense peak within range*/
                /*Pick leftmost peak within range*/
                            if (peakFreqPPM > maxpspl)
                {
                    maxipsl = peakIntensity;
                    maxpshl = peakFreqHz;
                    maxpspl = peakFreqPPM;
                }
                /*Pick rightmost peak within range*/
                            peakppmneg=peakFreqPPM*-1;
                            maxpspneg=maxpsp*-1;
if (peakppmneg > maxpspneg)
                {
                    maxipsr = peakIntensity;
                    maxpshr = peakFreqHz;
                    maxpspr = peakFreqPPM;
                }
                }
                }
    freePeakList();
                ppmdif=maxpspl-maxpspr;
                cent=maxpspl-ppmdif/2;
                tar=cent;
/*Writes peak position to text file and updates peak picking parameters*/
    RPROCNO(phpno)
    SETCURDATA
f1pcmpvar=tar+tardifppm;
f2pcmpvar=tar-tardifppm;
STOREPAR("f2p",f2pcmpvar)
STOREPAR("f1p",f1pcmpvar)
    fprintf(fcmp,"%f \n",cent);
tar=maxpsp;
v++;
}
END
fclose(fcmp);
QUIT
```

14.2.5 Script to extract peak positions of pH indicators

```
/*Bruker AU script for extracting peak positions of pH indicators from a CSI dataset*/
/*CSI dataset should have been fully processed in phase-sensitive mode*/
/*Rough chemical shift referencing in F2 can also help*/
/*The script works on Bruker Topspin 3.6.2 but has not been tested on other versions*/
/*The script extracts each row in turn into the empty procno requested (will overwrite existing contents!!!)*/
/*Peak picking routine will find the centre of a multiplet (doublet, quartet, or singlet with bad shim)*/
/*Will treat two peaks as outer edges of a multiplet if their intensity is within ppsens of the biggest peak found in specified range*/
/*Adjust peak picking ranges as appropriate to avoid overlap*/
/*To use this script, the component of interest must be the most intense signal*/
/*Will write peak positions/ppm of DCA, dss, formate, acetate, 26-lutidine, NHS,*/
/*glycine, methylamine, DSS to separate.txt files in procno directory of CSI dataset*/
/*These numbers go from first to final row of the CSI dataset*/
/*Numbers can be copied and pasted into spreadsheet*/
/*This AU is not fully tested and comes without warranty.*/
/*Use kill command if all goes wrong*/
/*Matthew Wallace, 1/2023*/
/*University of East Anglia, matthew.wallace@uea.ac.uk*/
FILE *fdss,*fdca,*fmpa,*fform,*fac,*flut,*fnhs,*fgly,*fmenh;
float min=0;
double f2pdss=-0.4;
double f1pdss=0.4;
double f2pdca=5.8;
double f1pdca=6.5;
double f2pmpa=1.0;
double f1pmpa=1.6;
double f1pform=8.6;
double f2pform=8.1;
double f2pac=1.8;
double f1pac=2.2;
double f2plut=2.4;
double f1plut=2.8;
double f2pnhs=2.55;
double f1pnhs=2.85;
double f2pgly=3.1;
double f1pgly=3.7;
double f2pmenh=2.10;
double f1pmenh=2.65;
float ppsens=0.9;
double pc=0.1;
int steno=15;
int eno;
int ne=5;
int m=-1;
double peakFreqHz, peakFreqPPM, peakIntensity, maxpsh, maxpsp, maxips, sf,sfo1,so1p;
double mintpp,minpsp,peakppmneg,cent,ppmdif,maxpspneg;
int i, numPeaks;
int np=128;
int row=1;
int v=1;
int phpno=1;
int wrpno=5;
GETCURDATA
steno=expno;
phpno=procno;
wrpno=procno+4;
FETCHPAR1("td",&np)
GETINT("Enter starting experiment number: ",steno)
GETINT("Enter number of gradient values",np)
GETINT("Enter procno of 2D dataset",phpno)
GETINT("Enter procno to extract rows into for peak picking (blank)",wrpno)
GETDOUBLE("DSS right peak picking/ppm",f2pdss)
GETDOUBLE("DSS left peak picking/ppm",f1pdss)
GETDOUBLE("DCA right peak picking/ppm",f2pdca)
GETDOUBLE("DCA left peak picking/ppm",f1pdca)
GETDOUBLE("MPA right peak picking/ppm",f2pmpa)
GETDOUBLE("MPA left peak picking/ppm",f1pmpa)
GETDOUBLE("Formate right peak picking/ppm",f2pform)
GETDOUBLE("Formate left peak picking/ppm",f1pform)
GETDOUBLE("Acetate right peak picking/ppm",f2pac)
GETDOUBLE("Acetate left peak picking/ppm",f1pac)
GETDOUBLE("2,6-lutidine right peak picking/ppm",f2plut)
GETDOUBLE("2,6-lutidine left peak picking/ppm",f1plut)
GETDOUBLE("NHS right peak picking/ppm",f2pnhs)
GETDOUBLE("NHS left peak picking/ppm",f1pnhs)
GETDOUBLE("Glycine right peak picking/ppm",f2pgly)
GETDOUBLE("Glycine left peak picking/ppm",f1pgly)
GETDOUBLE("Methylamine right peak picking/ppm",f2pmenh)
GETDOUBLE("Methylamine left peak picking/ppm",f1pmenh)
GETFLOAT("Enter peak picking senistitivty factor",pc)
GETDOUBLE("Enter satelite sensitivity factor",ppsens)
```

```
/*Reads in peak picking parameters for dss*/
REXPNO(steno)
SETCURDATA
RPROCNO(phpno)
SETCURDATA
STOREPAR("mi",min)
STOREPAR("pc",pc)
if ((fdss = fopen(PROCPATH("DSS.txt"),"wt")) == 0)
STOPMSG("Cannot create text file")
if ((fdca = fopen(PROCPATH("Dichloroacetate.txt"),"wt")) == 0)
STOPMSG("Cannot create text file")
if ((fmpa = fopen(PROCPATH("MPA.txt"),"wt")) == 0)
STOPMSG("Cannot create text file")
if ((fform = fopen(PROCPATH("Formate.txt"),"wt")) == 0)
STOPMSG("Cannot create text file")
if ((fac = fopen(PROCPATH("Acetate.txt"),"wt")) == 0)
STOPMSG("Cannot create text file")
if ((flut = fopen(PROCPATH("2,6-lutidine.txt"),"wt")) == 0)
STOPMSG("Cannot create text file")
if ((fnhs = fopen(PROCPATH("NHS.txt"),"wt")) == 0)
STOPMSG("Cannot create text file")
if ((fgly = fopen(PROCPATH("Glycine.txt"),"wt")) == 0)
STOPMSG("Cannot create text file")
if ((fmenh = fopen(PROCPATH("Methylamine.txt"),"wt")) == 0)
STOPMSG("Cannot create text file")
/*Now extract each row in turn into requested procno, peak positino of each species and write positions to file*/
TIMES(np)
{
RSR(v,wrpno)
RPROCNO(wrpno)
SETCURDATA
/*Extract DSS*/
STOREPAR("f2p",f2pdss)
STOREPAR("f1p",f1pdss)
PP
 numPeaks = readPeakList(PROCPATH(0));
maxips=0.0;
    maxpsh=0.0;
    for (i=0; i<numPeaks; i++)
    {
                peakIntensity = getPeakIntensity(i);
                peakFreqHz = getPeakFreqHz(i);
                peakFreqPPM = getPeakFreqPPM(i);
                if (peakIntensity > maxips)
                {
                    maxips = peakIntensity;
                    maxpsh = peakFreqHz;
                    maxpsp = peakFreqPPM;
                }
    }
    /*Pick most downfield side of multiplet*/
    mintpp=maxips*ppsens;
maxpsp=0.0;
    for (i=0; i<numPeaks; i++)
    {
                                peakIntensity = getPeakIntensity(i);
                                if(peakIntensity>mintpp)
                                {
                peakFreqPPM = getPeakFreqPPM(i);
                peakFreqHz = getPeakFreqHz(i);
                if (peakFreqHz >= maxpsh)
                {
                    maxpsp = peakFreqPPM;
                    maxpsh = peakFreqHz;
                }
    }
                }
/*Flips negative to choose most upfield peak of multiplet*/
for (i=0; i<numPeaks; i++)
    {
                                peakIntensity = getPeakIntensity(i);
                                if(peakIntensity>mintpp)
                                {
                peakFreqPPM = getPeakFreqPPM(i);
peakppmneg=peakFreqPPM*m;
maxpspneg=maxpsp*m;
                if (peakppmneg >= maxpspneg)
                {
                    minpsp = peakFreqPPM;
                }
    }
                }
    freePeakList();
/*writes centre of multiplet into text document*/
```

```
ppmdif=maxpsp-minpsp;
cent=minpsp+ppmdif*0.5;
   fprintf(fdss,"%f \n",cent);
   /*Extract DCA*/
STOREPAR("f2p",f2pdca)
STOREPAR("f1p",f1pdca)
PP
 numPeaks = readPeakList(PROCPATH(0));
maxips=0.0;
   maxpsh=0.0;
   for (i=0; i<numPeaks; i++)
   {
              peakIntensity = getPeakIntensity(i);
              peakFreqHz = getPeakFreqHz(i);
              peakFreqPPM = getPeakFreqPPM(i);
              if (peakIntensity > maxips)
              {
                 maxips = peakIntensity;
                 maxpsh = peakFreqHz;
                 maxpsp = peakFreqPPM;
              }
   }
   /*Pick most downfield side of multiplet*/
   mintpp=maxips*ppsens;
maxpsp=0.0;
   for (i=0; i<numPeaks; i++)
   {
                        peakIntensity = getPeakIntensity(i);
                        if(peakIntensity>mintpp)
                        {
              peakFreqPPM = getPeakFreqPPM(i);
              peakFreqHz = getPeakFreqHz(i);
              if (peakFreqHz >= maxpsh)
              {
                 maxpsp = peakFreqPPM;
                 maxpsh = peakFreqHz;
              }
   }
              }
/*Flips negative to choose most upfield peak of multiplet*/
for (i=0; i<numPeaks; i++)
   {
                        peakIntensity = getPeakIntensity(i);
                        if(peakIntensity>mintpp)
                        {
              peakFreqPPM = getPeakFreqPPM(i);
peakppmneg=peakFreqPPM*m;
maxpspneg=maxpsp*m;
              if (peakppmneg >= maxpspneg)
              {
                 minpsp = peakFreqPPM;
              }
   }
              }
   freePeakList();
/*writes centre of multiplet into text document*/
ppmdif=maxpsp-minpsp;
cent=minpsp+ppmdif*0.5;
   fprintf(fdca,"%f \n",cent);
/*Extract MPA*/
STOREPAR("f2p",f2pmpa)
STOREPAR("f1p",f1pmpa)
PP
 numPeaks = readPeakList(PROCPATH(0));
maxips=0.0;
   maxpsh=0.0;
   for (i=0; i<numPeaks; i++)
   {
              peakIntensity = getPeakIntensity(i);
              peakFreqHz = getPeakFreqHz(i);
              peakFreqPPM = getPeakFreqPPM(i);
              if (peakIntensity > maxips)
              {
                 maxips = peakIntensity;
                 maxpsh = peakFreqHz;
                 maxpsp = peakFreqPPM;
              }
   }
   /*Pick most downfield side of multiplet*/
   mintpp=maxips*ppsens;
maxpsp=0.0;
   for (i=0; i<numPeaks; i++)
   {
                        peakIntensity = getPeakIntensity(i);
```

```
                    if(peakIntensity>mintpp)
                    {
          peakFreqPPM = getPeakFreqPPM(i);
          peakFreqHz = getPeakFreqHz(i);
          if (peakFreqHz >= maxpsh)
          {
            maxpsp = peakFreqPPM;
            maxpsh = peakFreqHz;
          }
  }
          }
/*Flips negative to choose most upfield peak of multiplet*/
for (i=0; i<numPeaks; i++)
  {
                    peakIntensity = getPeakIntensity(i);
                    if(peakIntensity>mintpp)
                    {
          peakFreqPPM = getPeakFreqPPM(i);
peakppmneg=peakFreqPPM*m;
maxpspneg=maxpsp*m;
          if (peakppmneg >= maxpspneg)
          {
            minpsp = peakFreqPPM;
          }
  }
          }
  freePeakList();
/*writes centre of multiplet into text document*/
ppmdif=maxpsp-minpsp;
cent=minpsp+ppmdif*0.5;
  fprintf(fmpa,"%f \n",cent);
/*Extract FORM*/
STOREPAR("f2p",f2pform)
STOREPAR("f1p",f1pform)
PP
 numPeaks = readPeakList(PROCPATH(0));
maxips=0.0;
  maxpsh=0.0;
  for (i=0; i<numPeaks; i++)
  {
          peakIntensity = getPeakIntensity(i);
          peakFreqHz = getPeakFreqHz(i);
          peakFreqPPM = getPeakFreqPPM(i);
          if (peakIntensity > maxips)
          {
            maxips = peakIntensity;
            maxpsh = peakFreqHz;
            maxpsp = peakFreqPPM;
          }
  }
  /*Pick most downfield side of multiplet*/
  mintpp=maxips*ppsens;
maxpsp=0.0;
  for (i=0; i<numPeaks; i++)
  {
                    peakIntensity = getPeakIntensity(i);
                    if(peakIntensity>mintpp)
                    {
          peakFreqPPM = getPeakFreqPPM(i);
          peakFreqHz = getPeakFreqHz(i);
          if (peakFreqHz >= maxpsh)
          {
            maxpsp = peakFreqPPM;
            maxpsh = peakFreqHz;
          }
  }
          }
/*Flips negative to choose most upfield peak of multiplet*/
for (i=0; i<numPeaks; i++)
  {
                    peakIntensity = getPeakIntensity(i);
                    if(peakIntensity>mintpp)
                    {
          peakFreqPPM = getPeakFreqPPM(i);
peakppmneg=peakFreqPPM*m;
maxpspneg=maxpsp*m;
          if (peakppmneg >= maxpspneg)
          {
            minpsp = peakFreqPPM;
          }
  }
          }
  freePeakList();
/*writes centre of multiplet into text document*/
```

```
ppmdif=maxpsp-minpsp;
cent=minpsp+ppmdif*0.5;
   fprintf(fform,"%f \n",cent);
/*Extract AC*/
STOREPAR("f2p",f2pac)
STOREPAR("f1p",f1pac)
PP
 numPeaks = readPeakList(PROCPATH(0));
maxips=0.0;
   maxpsh=0.0;
   for (i=0; i<numPeaks; i++)
   {
              peakIntensity = getPeakIntensity(i);
              peakFreqHz = getPeakFreqHz(i);
              peakFreqPPM = getPeakFreqPPM(i);
              if (peakIntensity > maxips)
              {
                 maxips = peakIntensity;
                 maxpsh = peakFreqHz;
                 maxpsp = peakFreqPPM;
              }
   }
   /*Pick most downfield side of multiplet*/
   mintpp=maxips*ppsens;
maxpsp=0.0;
   for (i=0; i<numPeaks; i++)
   {
                            peakIntensity = getPeakIntensity(i);
                            if(peakIntensity>mintpp)
                            {
              peakFreqPPM = getPeakFreqPPM(i);
              peakFreqHz = getPeakFreqHz(i);
              if (peakFreqHz >= maxpsh)
              {
                 maxpsp = peakFreqPPM;
                 maxpsh = peakFreqHz;
              }
   }
              }
/*Flips negative to choose most upfield peak of multiplet*/
for (i=0; i<numPeaks; i++)
   {
                            peakIntensity = getPeakIntensity(i);
                            if(peakIntensity>mintpp)
                            {
              peakFreqPPM = getPeakFreqPPM(i);
peakppmneg=peakFreqPPM*m;
maxpspneg=maxpsp*m;
              if (peakppmneg >= maxpspneg)
              {
                 minpsp = peakFreqPPM;
              }
   }
              }
   freePeakList();
/*writes centre of multiplet into text document*/
ppmdif=maxpsp-minpsp;
cent=minpsp+ppmdif*0.5;
   fprintf(fac,"%f \n",cent);
/*Extract LUT*/
STOREPAR("f2p",f2plut)
STOREPAR("f1p",f1plut)
PP
 numPeaks = readPeakList(PROCPATH(0));
maxips=0.0;
   maxpsh=0.0;
   for (i=0; i<numPeaks; i++)
   {
              peakIntensity = getPeakIntensity(i);
              peakFreqHz = getPeakFreqHz(i);
              peakFreqPPM = getPeakFreqPPM(i);
              if (peakIntensity > maxips)
              {
                 maxips = peakIntensity;
                 maxpsh = peakFreqHz;
                 maxpsp = peakFreqPPM;
              }
   }
   /*Pick most downfield side of multiplet*/
   mintpp=maxips*ppsens;
maxpsp=0.0;
   for (i=0; i<numPeaks; i++)
   {
                            peakIntensity = getPeakIntensity(i);
```

```
                    if(peakIntensity>mintpp)
                    {
          peakFreqPPM = getPeakFreqPPM(i);
          peakFreqHz = getPeakFreqHz(i);
          if (peakFreqHz >= maxpsh)
          {
            maxpsp = peakFreqPPM;
            maxpsh = peakFreqHz;
          }
  }
          }
/*Flips negative to choose most upfield peak of multiplet*/
for (i=0; i<numPeaks; i++)
  {
                    peakIntensity = getPeakIntensity(i);
                    if(peakIntensity>mintpp)
                    {
          peakFreqPPM = getPeakFreqPPM(i);
peakppmneg=peakFreqPPM*m;
maxpspneg=maxpsp*m;
          if (peakppmneg >= maxpspneg)
          {
            minpsp = peakFreqPPM;
          }
  }
          }
  freePeakList();
/*writes centre of multiplet into text document*/
ppmdif=maxpsp-minpsp;
cent=minpsp+ppmdif*0.5;
  fprintf(flut,"%f \n",cent);
/*Extract NHS*/
STOREPAR("f2p",f2pnhs)
STOREPAR("f1p",f1pnhs)
PP
 numPeaks = readPeakList(PROCPATH(0));
maxips=0.0;
  maxpsh=0.0;
  for (i=0; i<numPeaks; i++)
  {
          peakIntensity = getPeakIntensity(i);
          peakFreqHz = getPeakFreqHz(i);
          peakFreqPPM = getPeakFreqPPM(i);
          if (peakIntensity > maxips)
          {
            maxips = peakIntensity;
            maxpsh = peakFreqHz;
            maxpsp = peakFreqPPM;
          }
  }
  /*Pick most downfield side of multiplet*/
  mintpp=maxips*ppsens;
maxpsp=0.0;
  for (i=0; i<numPeaks; i++)
  {
                    peakIntensity = getPeakIntensity(i);
                    if(peakIntensity>mintpp)
                    {
          peakFreqPPM = getPeakFreqPPM(i);
          peakFreqHz = getPeakFreqHz(i);
          if (peakFreqHz >= maxpsh)
          {
            maxpsp = peakFreqPPM;
            maxpsh = peakFreqHz;
          }
  }
          }
/*Flips negative to choose most upfield peak of multiplet*/
for (i=0; i<numPeaks; i++)
  {
                    peakIntensity = getPeakIntensity(i);
                    if(peakIntensity>mintpp)
                    {
          peakFreqPPM = getPeakFreqPPM(i);
peakppmneg=peakFreqPPM*m;
maxpspneg=maxpsp*m;
          if (peakppmneg >= maxpspneg)
          {
            minpsp = peakFreqPPM;
          }
  }
          }
  freePeakList();
/*writes centre of multiplet into text document*/
```

```
ppmdif=maxpsp-minpsp;
cent=minpsp+ppmdif*0.5;
   fprintf(fnhs,"%f \n",cent);
/*Extract GLY*/
STOREPAR("f2p",f2pgly)
STOREPAR("f1p",f1pgly)
PP
 numPeaks = readPeakList(PROCPATH(0));
maxips=0.0;
   maxpsh=0.0;
   for (i=0; i<numPeaks; i++)
   {
              peakIntensity = getPeakIntensity(i);
              peakFreqHz = getPeakFreqHz(i);
              peakFreqPPM = getPeakFreqPPM(i);
              if (peakIntensity > maxips)
              {
                 maxips = peakIntensity;
                 maxpsh = peakFreqHz;
                 maxpsp = peakFreqPPM;
              }
   }
   /*Pick most downfield side of multiplet*/
   mintpp=maxips*ppsens;
maxpsp=0.0;
   for (i=0; i<numPeaks; i++)
   {
                            peakIntensity = getPeakIntensity(i);
                            if(peakIntensity>mintpp)
                            {
              peakFreqPPM = getPeakFreqPPM(i);
              peakFreqHz = getPeakFreqHz(i);
              if (peakFreqHz >= maxpsh)
              {
                 maxpsp = peakFreqPPM;
                 maxpsh = peakFreqHz;
              }
   }
              }
/*Flips negative to choose most upfield peak of multiplet*/
for (i=0; i<numPeaks; i++)
   {
                            peakIntensity = getPeakIntensity(i);
                            if(peakIntensity>mintpp)
                            {
              peakFreqPPM = getPeakFreqPPM(i);
peakppmneg=peakFreqPPM*m;
maxpspneg=maxpsp*m;
              if (peakppmneg >= maxpspneg)
              {
                 minpsp = peakFreqPPM;
              }
   }
              }
   freePeakList();
/*writes centre of multiplet into text document*/
ppmdif=maxpsp-minpsp;
cent=minpsp+ppmdif*0.5;
   fprintf(fgly,"%f \n",cent);
/*Extract MENH*/
STOREPAR("f2p",f2pmenh)
STOREPAR("f1p",f1pmenh)
PP
 numPeaks = readPeakList(PROCPATH(0));
maxips=0.0;
   maxpsh=0.0;
   for (i=0; i<numPeaks; i++)
   {
              peakIntensity = getPeakIntensity(i);
              peakFreqHz = getPeakFreqHz(i);
              peakFreqPPM = getPeakFreqPPM(i);
              if (peakIntensity > maxips)
              {
                 maxips = peakIntensity;
                 maxpsh = peakFreqHz;
                 maxpsp = peakFreqPPM;
              }
   }
   /*Pick most downfield side of multiplet*/
   mintpp=maxips*ppsens;
maxpsp=0.0;
   for (i=0; i<numPeaks; i++)
   {
                            peakIntensity = getPeakIntensity(i);
```

```
                        if(peakIntensity>mintpp)
                        {
            peakFreqPPM = getPeakFreqPPM(i);
            peakFreqHz = getPeakFreqHz(i);
            if (peakFreqHz >= maxpsh)
            {
               maxpsp = peakFreqPPM;
               maxpsh = peakFreqHz;
            }
   }
            }
/*Flips negative to choose most upfield peak of multiplet*/
for (i=0; i<numPeaks; i++)
   {
                        peakIntensity = getPeakIntensity(i);
                        if(peakIntensity>mintpp)
                        {
            peakFreqPPM = getPeakFreqPPM(i);
peakppmneg=peakFreqPPM*m;
maxpspneg=maxpsp*m;
            if (peakppmneg >= maxpspneg)
            {
               minpsp = peakFreqPPM;
            }
   }
            }
   freePeakList();
/*writes centre of multiplet into text document*/
ppmdif=maxpsp-minpsp;
cent=minpsp+ppmdif*0.5;
   fprintf(fmenh,"%f \n",cent);
/*Now advance row counter and repeat for the next row*/
v++;
RPROCNO(phpno)
SETCURDATA
}
END
fclose(fdss);
fclose(fdca);
fclose(fdss);
fclose(fform);
fclose(fac);
fclose(flut);
fclose(fnhs);
fclose(fgly);
fclose(fmenh);
QUIT
```

14.3 Pulse programs for CSI experiments (Bruker Topspin)

14.3.1 Pulse sequence with perfect echo water suppression

^{1}H chemical shift images were acquired using a gradient phase encoding sequence based on Trigo-Mouriño *et al*.[54] The sequence incorporated the perfect echo Watergate sequence of Adams *et al.*[49] incorporating the double echo W5 pulse train. Experiments were acquired with a vertical window (cnst0) of 2.6 cm. d1 was set as 0.1 s.

```
;!!!This version is for running unlocked with sweep etc. disabled
;!!!For running locked, delete the (UN)BLKGRAMP and insert the (UN)BLKGRAD
;!!!statements as in the standard 1D water suppression sequences
;Modified from: "Probing spatial distribution of alignment by deuterium NMR imaging"
;Chem. Eur. J., 9, 2013, 7013-7019. DOI: 10.1002/chem.201300254
;2D sequence for z-imaging preserving chemical shift
;Original sequence written by Christian Merle, Martin Koos
;Modified to be on 1H with Perfect Echo Watergate component for water suppression
;Watergate component taken from: "Perfecting WATERGATE: clean proton NMR spectra from aqueous solution"
;Adams RW, Holroyd CM, Aguilar JA, Nilsson M, Morris GA,  Chem. Comm., 49 (2013), 358-360. DOI: 10.1039/C2CC37579F
;itself based on: water suppression using watergate W5 pulse sequence with gradients using double echo;
;M. Liu, X. Mao, C. He, H. Huang, J.K. Nicholson & J.C. Lindon, J. Magn. Reson. 132, 125 - 129 (1998)
;Set 1 SW to Z-range in mm (see cnst0) to get 1 Hz/mm scale in indirect dimension
;Make cnst0 bigger than actual sample size to avoid folding artefacts.
;Keep gpz6 at 100% and adjust cnst3 to get p30 to an acceptable length according to instrument (ca. 150-300 us)
;This pulse program is not fully tested and comes without warranty.
;Check the sequence and your parameters carefully before use.
;Matthew Wallace, 9/2022 (University of East Anglia, matthew.wallace@uea.ac.uk)
;1H-Version
;$CLASS=HighRes
;$DIM=2D
;$TYPE=
;$SUBTYPE=
;$COMMENT=
prosol relations=<triple>
#include <Avance.incl>
```

```
#include <Grad.incl>
#include <Delay.incl>
"cnst2= 0.8914027" ; integralfactor of gradient shape SMSQ10.32
"cnst4= 267.52220" ; * 10^6 /Ts = gamma1H
"p30=(td1/cnst0)*(1/(cnst1*cnst2*cnst3))*(1/cnst4)*(2*3.14159265/1000)*0.5 s"
"l1=td1-1"
lgrad r1d = l1
"acqt0=0"
"l1=td1-1"
"d19=1/(2*cnst19)"
"DELTA1=p30+d16"

1 ze
2 30m
100m
3 30m
d1
;spoil gradient from previous
 50u UNBLKGRAMP
p19:gp3
d16
;start of Perfect echo water suppression
20u pl1:f1
  p1 ph1
50u
p16:gp1
 d16 pl18:f1
DELTA1
  p27*0.087 ph3
  d19*2
  p27*0.206 ph3
  d19*2
  p27*0.413 ph3
  d19*2
  p27*0.778 ph3
  d19*2
  p27*1.491 ph3
  d19*2
  p27*1.491 ph4
  d19*2
  p27*0.778 ph4
  d19*2
  p27*0.413 ph4
  d19*2
  p27*0.206 ph4
  d19*2
  p27*0.087 ph4
DELTA1
p16:gp1
d16 pl1:f1
50u
50u
  p1 ph10
50u
p16:gp2
d16
DELTA1
50u
  p27*0.087 ph5
  d19*2
  p27*0.206 ph5
  d19*2
  p27*0.413 ph5
  d19*2
  p27*0.778 ph5
  d19*2
  p27*1.491 ph5
  d19*2
  p27*1.491 ph6
  d19*2
  p27*0.778 ph6
  d19*2
  p27*0.413 ph6
  d19*2
  p27*0.206 ph6
  d19*2
  p27*0.087 ph6
50u
p30:gp6*r1d*cnst3
d16
p16:gp2
d16 BLKGRAMP
go=2 ph31
30m
100m wr #0 if #0 zd igrad r1d
lo to 3 times l1
goto 5
; run last increment:
4 30m
100m
5 30m
d1
;spoil gradient from previous
50u UNBLKGRAMP
p19:gp3
d16
  20u pl1:f1
  p1 ph1
```

```
50u
  p16:gp1
  d16 pl18:f1
DELTA1
  p27*0.087 ph3
  d19*2
  p27*0.206 ph3
  d19*2
  p27*0.413 ph3
  d19*2
  p27*0.778 ph3
  d19*2
  p27*1.491 ph3
  d19*2
  p27*1.491 ph4
  d19*2
  p27*0.778 ph4
  d19*2
  p27*0.413 ph4
  d19*2
  p27*0.206 ph4
  d19*2
  p27*0.087 ph4
DELTA1
  p16:gp1
d16 pl1:f1
 50u
50u
  p1 ph10
  50u
  p16:gp2
  d16
DELTA1
50u
  p27*0.087 ph5
  d19*2
  p27*0.206 ph5
  d19*2
  p27*0.413 ph5
  d19*2
  p27*0.778 ph5
  d19*2
  p27*1.491 ph5
  d19*2
  p27*1.491 ph6
  d19*2
  p27*0.778 ph6
  d19*2
  p27*0.413 ph6
  d19*2
  p27*0.206 ph6
  d19*2
  p27*0.087 ph6
50u
p30:gp6*r1d*cnst3
d16
  p16:gp2
  d16 BLKGRAMP
go=4 ph31
30m
100m wr #0 if #0 zd
exit
ph1=0 2
ph3=0 0 1 1 2 2 3 3
ph4=2 2 3 3 0 0 1 1
ph5=0 0 0 0 0 0 0 0 1 1 1 1 1 1 1 1
    2 2 2 2 2 2 2 2 3 3 3 3 3 3 3 3
ph6=2 2 2 2 2 2 2 2 3 3 3 3 3 3 3 3
    0 0 0 0 0 0 0 0 1 1 1 1 1 1 1 1
ph10=1 3
ph31=0 2 2 0 0 2 2 0 2 0 0 2 2 0 0 2

;cnst0 : z-Range in cm
;cnst1 : GCC (G/mm) from Gradpar
;cnst3 : set to get GP of sufficient length
;cnst19 : Distance to next null of WATERGATE sequence (in Hz)
;pl1 : f1 channel - power level for pulse (default)
;pl18: f1 channel - power level for 3-9-19-pulse (watergate)
;p1 : f1 channel - 90 degree high power pulse
;p16 : watergate gradient pulse  [1000u]
;p19 : Spoil gradient pulse [1000u]
;p27: f1 channel -  90 degree pulse at pl18
;gpz6: 100% phase encoding gradient
;d12: delay for power switching                    [20 usec]
;d16: standard eddy delay [200u]
;d1 :  relaxation delay (in addition to 160 ms delay)
;d19: delay for binomial water suppression
;     d19 = (1/(2*d)), d = distance of next null (in Hz)
;ns: 8 * n, total number of scans: NS * TD0
;ds: 16
;td1: number of experiments
;FnMODE: QF
;use gradient ratio:    gp 1 : gp 2
;                       34 :   22
;for z-only gradients:
;gpz1: 34%
;gpz2: 22%
;gpz3: 50%
```

```
;use gradient files:
;gpnam1: SMSQ10.100
;gpnam2: SMSQ10.100
;gpnam3: SMSQ10.100
;gpnam6: SMSQ10.32
;$Id: phaseenc,v 1.1 2011/08/10 15:12:45 ber Exp $
```

14.3.2 Pulse sequence for analysis of biphasic samples

```
;!!!This version is for running unlocked with sweep etc. disabled
;For analysis of biphasic samples (which generally won't lock or shim)
;Modified from: "Probing spatial distribution of alignment by deuterium NMR imaging"
;Chem. Eur. J., 9, 2013, 7013-7019. DOI: 10.1002/chem.201300254
;2D sequence for z-imaging preserving chemical shift
;Original sequence written by Christian Merle, Martin Koos
;Modified to be on 1H with spin echo
;Set 1 SW to Z-range in mm (see cnst0) to get 1 Hz/mm scale in indirect dimension
;Make cnst0 bigger than actual sample size to avoid folding artefacts.
;Keep gpz6 at 100% and adjust cnst3 to get p30 to an acceptable length according to instrument (ca. 150-300 us)
;This pulse program is not fully tested and comes without warranty.
;Check the sequence and your parameters carefully before use.
;Matthew Wallace, 9/2022 (University of East Anglia, matthew.wallace@uea.ac.uk)
;1H-Version
;$CLASS=HighRes
;$DIM=2D
;$TYPE=
;$SUBTYPE=
;$COMMENT=
prosol relations=<triple>
#include <Avance.incl>
#include <Grad.incl>
#include <Delay.incl>
"cnst2= 0.8914027" ; integralfactor of gradient shape SMSQ10.32
"cnst4= 267.52220" ; * 10^6 /Ts = gamma1H
"p30=(td1/cnst0)*(1/(cnst1*cnst2*cnst3))*(1/cnst4)*(2*3.14159265/1000)*0.5 s"
"l1=td1-1"
lgrad r1d = l1
"acqt0=0"
"l1=td1-1"
"p2=p1*2"
"DELTA1=d6+p30+5u+d16"

1 ze
2 30m
3 d1
 50u UNBLKGRAMP
  p1 ph1
DELTA1
p2 ph2
d6
p30:gp6*r1d*cnst3
5u
d16 BLKGRAMP
go=2 ph31
30m wr #0 if #0 zd igrad r1d
lo to 3 times l1
goto 5
; run last increment:
4 30m
5 d1
50u UNBLKGRAMP
p1 ph1
DELTA1
p2 ph2
d6
p30:gp6*r1d*cnst3
5u
d16 BLKGRAMP
go=4 ph31
30m wr #0 if #0 zd
exit
ph1=0 0 2 2 1 1 3 3
ph2=1 3 1 3 0 2 0 2
ph31=0 0 2 2 1 1 3 3

;cnst0 : z-Range in cm
;cnst1 : GCC (G/mm) from Gradpar
;cnst3 : set to get GP of sufficient length
;pl1 : f1 channel - power level for pulse (default)
;p1 : f1 channel - 90 degree high power pulse
;gpz6: 100% phase encoding gradient
;d16: standard eddy delay [200u]
;d1 :  relaxation delay
;d6 : pre GP delay [10u]
;ns: 2*n
;ds: 1*m
;td1: number of experiments
;FnMODE: QF
;gpnam6: SMSQ10.32
;$Id: phaseenc,v 1.1 2011/08/10 15:12:45 ber Exp $
```
